# Beliefs and misperceptions about naloxone and overdose among U.S. laypersons: a cross-sectional study

**DOI:** 10.1186/s12889-022-13298-3

**Published:** 2022-05-10

**Authors:** Jon Agley, Yunyu Xiao, Lori Eldridge, Beth Meyerson, Lilian Golzarri-Arroyo

**Affiliations:** 1 Prevention Insights, Department of Applied Health Science, School of Public Health Bloomington, Indiana University Bloomington, 809 E. 9th St., Bloomington, IN 47405 USA; 2 Department of Population Health Sciences, Weill Cornell Medicine, New York, NY USA; 3 College of Health and Human Performance, East Carolina University, Greenville, NC USA; 4 Southwest Institute for Research On Women, College of Social & Behavioral Sciences, University of Arizona, Tucson, AZ USA; 5 Biostatistics Consulting Center, School of Public Health Bloomington, Indiana University Bloomington, Bloomington, IN USA

**Keywords:** Naloxone, Overdose, Misinformation, Trust in science, Opioids, Opioid epidemic

## Abstract

**Background:**

Overdose education and naloxone distribution (OEND) to laypersons are key approaches to reduce the incidence of opioid-involved overdoses. While some research has examined attitudes toward OEND, especially among pharmacists and first responders, our understanding of what laypersons believe about overdose and naloxone is surprisingly limited. Further, some scholars have expressed concerns about the prevalence of non-evidence-based beliefs about overdose and naloxone. We designed this study to analyze the prevalence, nature, and context of beliefs about naloxone and overdose among U.S. laypersons.

**Methods:**

We conducted a cross-sectional study (*n* = 702) using Prolific.co (representative of the U.S. population by age, gender, and race). Primary outcomes were the believability of six statements about overdose/naloxone on a seven-point Likert-type scale. Five statements were unsupported, and one was supported, by current scientific evidence. We used latent profile analysis to classify participants into belief groups, then used regression to study correlates of profile classification.

**Results:**

Believability of the statements (7: extremely believable) ranged from m = 5.57 (SD = 1.38) for a scientifically supported idea (trained bystanders can reverse overdose with naloxone), to m = 3.33 (SD = 1.83) for a statement claiming opioid users can get high on naloxone. Participants were classified into three latent belief profiles: Profile 1 (most aligned with current evidence; *n* = 246), Profile 2 (moderately aligned; *n* = 351), and Profile 3 (least aligned, *n* = 105). Compared to Profile 1, several covariates were associated with categorization into Profiles 2 and 3, including lower trust in science (RRR = 0.36, 95%CI = 0.24–0.54; RRR = 0.21, 95%CI = 0.12–0.36, respectively), conservative political orientation (RRR = 1.41, 95%CI = 1.23–1.63; 3:RRR = 1.62, 95%CI = 1.35–1.95, respectively), and never being trained about naloxone (Profile 3: RRR = 3.37, 95%CI = 1.16–9.77).

**Conclusions:**

Preliminary evidence suggests some U.S. laypersons simultaneously believe that bystander overdose prevention with naloxone can prevent overdose and one or more scientifically unsupported claims about naloxone/overdose. Categorization into clusters displaying such belief patterns was associated with low trust in science, conservative political orientation, and not having been trained about naloxone.

**Preregistration:**

This cross-sectional study was preregistered prior to any data collection using the Open Science Framework: https://osf.io/c6ufv

**Supplementary Information:**

The online version contains supplementary material available at 10.1186/s12889-022-13298-3.

## Background

### Naloxone and the overdose death epidemic

For the 12-month period ending in May 2021, the United States (U.S.) reported 97,516 overdose deaths, the majority of which involved opioids [1]. One important component of a comprehensive national response to the overdose death epidemic is education on and distribution of naloxone [2], an opioid receptor antagonist that is used to reverse the effects of opioids, thereby preventing overdose death [3]. Research studies and expert analysis consistently have affirmed the value of naloxone availability and training as an overdose mitigation approach [4–7].

### Attitudes toward expanded naloxone access

Some researchers recently have expressed concern that misinformation about naloxone may hamper its distribution or use [8]. However, the nature and prevalence of such misinformation in the U.S. remains unclear, and studies thereof often are intermixed with broader concepts of support for or objection to expanded access. A 2010 summary of objections to take-home naloxone [9] highlighted both policy-level opposition from the early 2000s and instances of controversy in the news media regarding access or rumors about naloxone. Examples of the latter [10, 11] have continued to appear intermittently in national and local media in the past decade.

A few papers have examined lay support for naloxone access (or, conversely, opposition thereto). One study found associations between Just World Belief (“people getting what they deserve and deserving what they get”), individualism, and concern about naloxone access expansion [12]. Another survey found correlations between opposition to nonprescription naloxone and a variety of factors including social dominance orientation (support for inequality between different groups), endorsing authoritarian ideas, and perceiving that opioid users present a threat to the nation [13]. At the same time, support in those studies for take-home naloxone was relatively high. In a different study examining layperson perception of community pharmacist dispensing of naloxone, roughly 2/3 of respondents were comfortable with such an approach, but those who were not often cited “promoting drug abuse and misuse” or “promoting reckless behavior” as reasons for discomfort [14]. Similar concerns have been elicited from law enforcement officers, more than 80% of whom in one recent study indicated that naloxone “gives people who use drugs an excuse to continue doing drugs,” though many respondents also indicated willingness and ability to use naloxone and interface with drug treatment programs [15].

Though such studies provide helpful context, we do not currently have a clear sense of the ways in which laypersons think about overdose and naloxone, nor do we know the prevalence of such beliefs or whether they co-occur. At the same time, despite proliferation of state-level support for naloxone distribution (e.g., third-party prescribing) [16], the combination of anecdotal examples in the news and other studies permits inference that at least some people hold beliefs about overdose and naloxone that either do not correspond with existing scientific evidence or are misinformed.

### Characterizing layperson beliefs about overdose and naloxone

To better understand layperson beliefs, we designed a study to examine how believable a national sample of respondents found statements about naloxone and overdose to be. The study examined beliefs across four conceptual domains.

The first domain was risk compensation beliefs – the idea that people who use opioids will use more opioids or be less likely to seek treatment if they have access to naloxone [17]. While there may be anecdotal exceptions, such beliefs do not align with extant evidence about population-level effects, which fairly strongly indicates that naloxone education and distribution are not associated with increased opioid use [18–21] or reduced risk perceptions for heroin use [22].

The second domain was beliefs about overdose inevitability – the idea that people who experience nonfatal overdose once will *usually* overdose again and will *usually* die of an overdose within the year. Here, we emphasize our inclusion of the term *usually *as a normative claim about the most likely outcome. In contrast to such beliefs, current evidence indicates that while risk of mortality and morbidity is substantively elevated following a nonfatal overdose, the preponderance of that risk is not attributable to a subsequent overdose (fatal or nonfatal), though subsequent overdoses can and do occur in a percentage of people, and an index overdose is a significant risk factor for a repeated overdose [23–26]. Risk of a repeated overdose also appears to be higher among those with diagnoses of anxiety, depression, or substance use disorders prior to overdose [27].

The third domain was believability of misinformation – in this case, the idea that take-home naloxone can be used to get high, which is not possible [3]. Finally, the fourth domain was related to the efficacy of layperson naloxone – the idea that training and provision of naloxone is associated with bystander prevention of community overdose. Research suggests that layperson naloxone training and distribution is feasible [28], and a large-scale observation study found that it was associated with reduced community deaths from overdose [29].

Our approach to understanding these beliefs was informed by our prior work on beliefs about COVID-19 [30, 31]. There, we found that reported believability of statements about COVID-19 clustered. There was a latent group of people that found a scientifically supported statement to be believable while finding misinformed or unsupported narratives to be unbelievable, and several groups that reported believing narratives that were either misinformed or unlikely to be true, while also *not* rejecting a scientifically supported statement. We hypothesized that beliefs about naloxone and overdose might cluster similarly. Further, trust in science was very strongly associated with COVID-19 belief profiles, but it was less clear whether that would be the case for naloxone and overdose, or whether other factors, such as knowledge and prior experiences, would offer more explanatory power.

Considering all these factors, we believe that examining beliefs about naloxone and overdose in a rigorous manner can potentially support important research and policy initiatives. Thus, this study tested three preregistered hypotheses (1, 2, and 2a) and one exploratory aim (3), included here verbatim: [32].

### Study hypotheses

- 1) There will be some prevalence of [unsupported beliefs] about naloxone; however, we are agnostic as to the degree of prevalence, except that it will be non-zero.
- 2) Latent profile analysis (LPA) of beliefs about naloxone, using the prespecified criteria to determine the number of profiles, will identify at least two latent profiles of study participants.
  - 2a) The largest latent profile will strongly endorse the true statement about naloxone (> 5) while generally finding all other statements to be unbelievable (< 3).
- 3) We will conduct an exploratory multivariate regression model to contrast each of the latent profiles. All variables indicated in the "Variables" section will be included in the model. Significance testing will be two-sided and contrast odds compared to the most populated latent profile.

## Methods

### Data collection

Participants were recruited on December 2–3, 2021, through the online research platform Prolific, as a nationally representative sample of the U.S. by race, gender, and age [33]. They were required to be age 18 or older and to reside in the U.S. Those who indicated interest in the study were provided a link to QualtricsXM, where the survey was hosted. After indicating agreement to participate (digital study information sheet), participants proceeded to the first section of the survey where they completed demographic items and were screened for possible VPN/bot use, inattention, and explicit dishonesty using validated approaches [34]. Sequentially, participants were asked to complete sociodemographic items (including quality control checks, per above), complete several attitude and belief items, rate believability of statements about naloxone, then complete the Trust in Science and Scientists inventory. Participants who fully completed the study and submitted it for payment to Prolific received $1.65 USD.

We aimed to recruit 700 individuals. We based our planned sample size on the intention to correctly compute the number of classes for our sample. Distances between classes could not be known in advance for this study, but prior research indicated that a separation of Cohen’s d = 0.8 with 10 indicators was feasible with a sample of 500 individuals [35]; thus, with 6 indicators, we aimed to recruit 700 individuals.

In total, 736 individuals signed up to participate; 29 failed quality checks, and five timed out or quit the survey before reaching the questions about naloxone. Those 34 individuals were excluded from participation and resampled. One participant quit the survey after the naloxone questions but before the end, and another fully completed the survey but did not submit to Prolific for compensation. Both of those individuals were retained. Thus, the final analytic sample was 702 participants.

### Study instrument

#### Narratives about naloxone

Participants were asked to indicate the believability [1 = Extremely unbelievable to 7 = Extremely believable] of six different statements about naloxone and overdose using the question formatting developed in our prior research [30, 31]. Items 1 and 2 were from the Naloxone-Related Risk Compensation Beliefs scale [17], items 3 and 4 were declarative statements disagreeing in principle with findings from research on opioid overdose [23–26], item 5 reflected an impossibility (e.g., misinformation), and item 6 was a declarative statement agreeing in principle with research on naloxone training and access [28, 29].

- (1) Opioid users will use more opioids if they know they have access to naloxone.
- (2) Opioid users will be less likely to seek out treatment if they have access to naloxone.
- (3) People who overdose once on opioids usually will overdose again.
- (4) People who experience a non-fatal opioid overdose usually die of another overdose within the next 12 months.
- (5) It is risky to provide take-home naloxone to opioid users because they can use it to get high.
- (6) If trained and provided with naloxone, bystanders can effectively prevent overdoses in the community.

### Covariates

The following variables were used as covariates, and are provided alongside rationale where appropriate:

- (1) Standardized questions for age, sex, ethnicity, and education level.
- (2) Profession (“Do you work in any of the following fields?”).
- (3) Rurality (“Please identify the category that best describes your primary location of residence”), given prior research suggesting community rurality may impact use of naloxone for some providers [36].
- (4) Religious commitment (“Please describe your level of religious commitment [this refers to any belief system]”) from 1 (low) to 10 (high), from our prior misinformation research [30, 31].
- (5) Political orientation (“Please describe your political orientation”) from 1 (liberal) to 10 (conservative), from our prior misinformation research [30, 31].
- (6) Political party orientation (“Regarding your political orientation, would you consider yourself to be…”) [Republican, Democrat, Other], based on recent research indicating face mask perceptions were more strongly associated with political party than general political orientation [37].
- (7) A set of items associated with accepting take-home naloxone in the emergency department from Kestler et al. [38] (injection drug use in the last 6 months, heroin use in the last 6 months, previously having received naloxone for an overdose, having witnessed an overdose of another, use of harm reduction services).
- (8) Training about prescription drug abuse (“In the past, have you received educational training about prescription drug abuse?”), wording from Panther et al. [39]
- (9) Training about naloxone (“In the past, have you received educational training about naloxone or Narcan?”), wording adapted from Panther et al. [39]
- (10) Trust in science and scientists, a 21-item scale developed by Nadelson et al. [40]

### Statistical analyses

Initial data cleaning, scale computation (i.e., trust in science), and descriptive statistics were completed in SPSS v.28 (IBM Corporation). Then, latent profile analysis (LPA) was conducted using Mplus version 8.3 (Muthen & Muthen, Los Angeles, CA). LPA is a person-centered analysis approach aimed at identifying meaningful response patterns among participants [41]. We used the methodology for LPA described in our prior work [30] as well as in our preregistration document [32]. This included examination of the conceptual meaning and fit indices (AIC, BIC, adjusted BIC, entropy, and Vuong-Lo-Mendel-Rubin Likelihood Ratio Test to examine k versus k-1 classes). Models that failed to converge or that included small class sizes (< 1%) were not considered.

Finally, multivariate multinomial logistic regression was conducted using STATA v.17 (StataCorp LLC, College Station, TX) to identify factors associated with belonging to latent profiles identified by the LPA analysis. All variables except having received harm reduction services from a program were included in the model. Significance testing was two-sided and at the 5% significance level, though meeting such a criterion was not taken as a definitive indicator of the importance of any given finding [42].

## Results

### Descriptive statistics

Sociodemographic characteristics and other covariates for the full study sample are provided in Table 1. The mean believability scores for the two risk compensation statements about (1) using more opioids or (2) being less likely to seek treatment (3.51 and 3.76, respectively) indicated low-moderate believability overall. The two statements about overdose inevitability were perceived to be more believable, with mean scores of 4.87 and 4.35, respectively. Very few participants found the statement that (3) “people who overdose once on opioids usually will overdose again” to be very unbelievable (47 individuals selected ‘1’ or ‘2’). In contrast, there was more ambivalence about the claim that (4) people who non-fatally overdose will die of another overdose within 12 months.

**Table 1:** Descriptive Statistics

Variable	N	%	Mean	SD
Gender				
* Male*	341	48.6	-	-
* Female*	346	49.3	-	-
* Non-binary*	13	1.9	-	-
* Transgender*	2	0.3	-	-
Hispanic, Latino, or Spanish Origin	57	8.1	-	-
Race				
* White*	519	73.9	-	-
* Black or African American*	93	13.2	-	-
* American Indian or Alaska Native*	4	0.6	-	-
* Asian*	51	7.3	-	-
* Multiple races*	27	3.8	-	-
* Other*	8	1.1	-	-
Age (years)	-	-	32.6	12.3
Education Level				
* No diploma (did not finish high school)*	9	1.3	-	-
* High school graduate, GED, or equivalent*	98	14.0	-	-
* Some college, no degree*	169	24.1	-	-
* Associate degree or bachelor’s degree*	333	47.4	-	-
* Master’s degree*	78	11.1	-	-
* Professional or doctoral degree*	15	2.1	-	-
Residence				
* A large city (*> *250,000 people)*	183	26.1	-	-
* A midsized city (100,000 to 249,999 people)*	129	18.4	-	-
* A large town (25,000 to 99,999 people*)	144	20.5	-	-
* A small town (2,500 to 24,999 people*)	113	16.1	-	-
* A suburb of a large city*	87	12.4	-	-
* A rural area (non-farm*)	38	5.4	-	-
* A rural area (farm)*	8	1.1	-	-
Religious Commitment (1: Low to 10: High)	-	-	3.58	3.0
Political Orientation (1: Liberal to 10: Conservative)	-	-	3.76	2.5
Political Party				
* Republican*	112	16.0	-	-
* Democrat*	374	53.3	-	-
* Other*	215	30.6	-	-
Area of Employment				
* Medicine (e.g., physician, nurse, physician assistant)*	47	6.7	-	-
* Behavioral health (e.g., social worker, counselor, psychologist)*	20	2.8	-	-
* First responder (e.g., law enforcement, fire, EMS)*	7	1.0	-	-
* Pharmacy (e.g., pharmacist, pharmacy technician)*	6	0.9	-	-
* Community service (e.g., parks and recreation, librarian, bus driver, or another public-facing role)*	32	4.6	-	-
* Currently unemployed*	193	27.5	-	-
* None of the above*	396	56.4	-	-
Received prior training about prescription drug abuse (1: Yes)	189	26.9	-	-
Received educational training about naloxone (Narcan) (1: Yes)	106	15.1	-	-
Use of opioids in the past month (non-medical)				
* Never*	679	96.7	-	-
* Once or twice*	15	2.1	-	-
* Monthly*	1	0.1	-	-
* Weekly*	3	0.4	-	-
* Daily or almost daily*	3	0.4	-	-
Use of any drug by injection in the past month (non-medical)				
* No, never*	698	99.4	-	-
* Yes, at least once in the past 6 months*	3	0.4	-	-
Ever received naloxone to reverse an overdose (1: Yes)	5	0.7	-	-
Ever personally witnessed an opioid overdose (1: Yes)	59	8.4	-	-
Ever received services from a harm reduction program (1: Yes)	1	0.1	-	-
Trust in Science (1: Low to 5: High)	-	-	3.81	0.61
Opioid users will use more opioids if they know they have access to naloxone^a^	-	-	3.51	1.73
Opioid users will be less likely to seek out treatment if they have access to naloxone^a^	-	-	3.76	1.73
People who overdose once on opioids usually will overdose again^a^	-	-	4.87	1.44
People who experience a non-fatal opioid overdose usually die of another overdose within the next 12 months^a^	-	-	4.35	1.33
It is risky to provide take-home naloxone to opioid users because they can use it to get high*	-	-	3.33	1.83
If trained and provided with naloxone, bystanders can effectively prevent overdoses in the community^a^	-	-	5.57	1.38

^a^All such items have responses ranging from (1: Extremely unbelievable to 7: Extremely believable)

The definitively incorrect statement about (5) getting high on take-home naloxone was the least believable statement, with a mean of 3.33, but was still perceived to be very believable (score of 6 or 7) by 100 participants. Finally, the statement about (6) bystander prevention of overdose with naloxone was the most believable statement (mean of 5.57), with only 24 participants finding it very unbelievable (‘1’ or ‘2’).

### Latent profile analysis

**Fig. 1:**
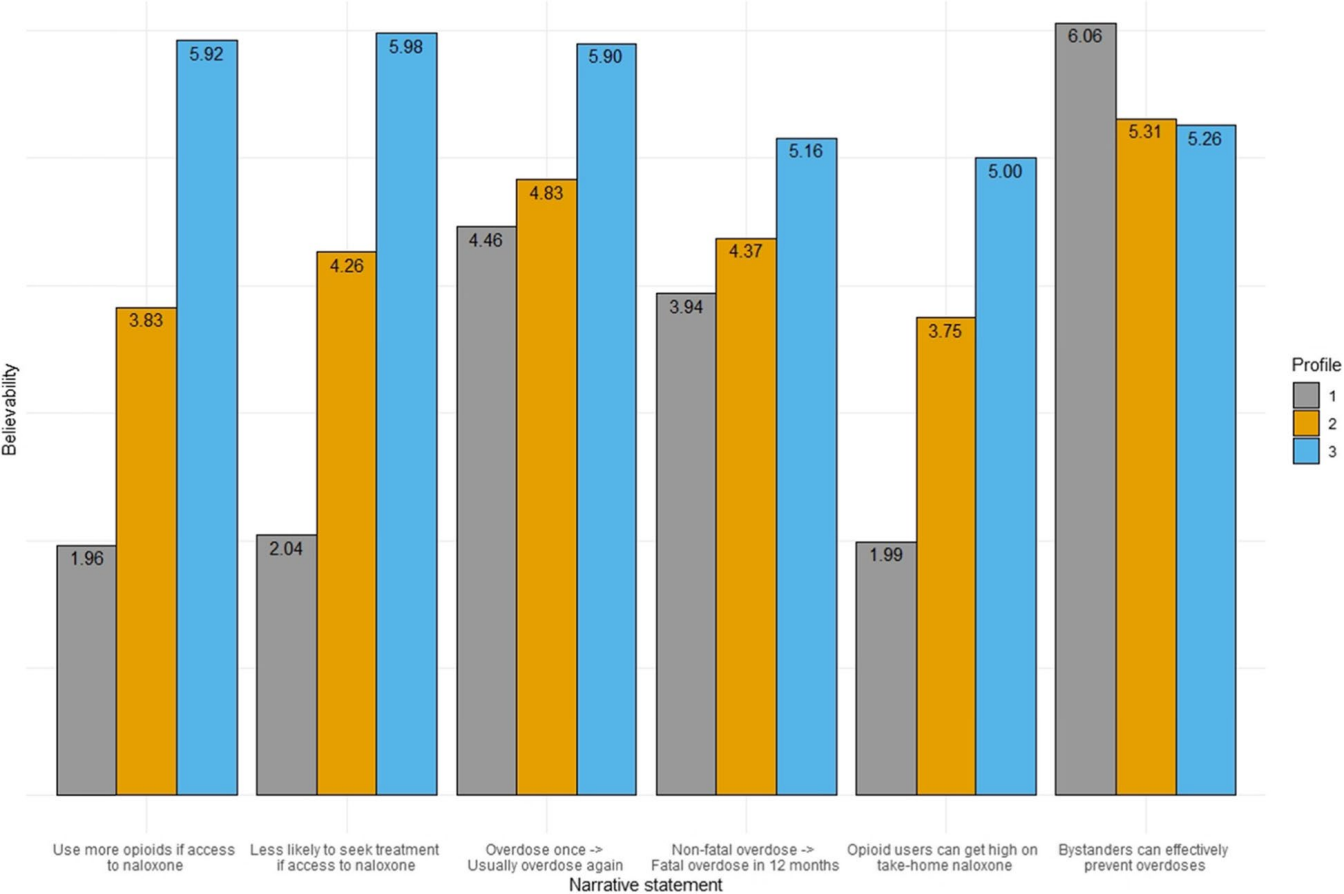
Narrative Believability by Latent Profile

Based on model fit parameters and empirical evidence, we selected a 3-class model. Fig. 1 shows the mean believability of the naloxone and overdose narrative statements across the profiles, and Table 2 displays the numeric values. Profiles were differentiated by the degree to which their beliefs aligned with currently available scientific evidence.

**Table 2:** Narrative Believability by Latent Profile

Variable	Profile 1	Profile 2	Profile 3
	(*n* = 246)	(*n* = 351)	(*n* = 105)
Opioid users will use more opioids if they know they have access to naloxone^a^	1.96	3.83	5.92
Opioid users will be less likely to seek out treatment if they have access to naloxone^a^	2.04	4.26	5.98
People who overdose once on opioids usually will overdose again^a^	4.46	4.83	5.00
People who experience a non-fatal opioid overdose usually die of another overdose within the next 12 months^a^	3.94	4.37	5.16
It is risky to provide take-home naloxone to opioid users because they can use it to get high^a^	1.99	3.75	5.00
If trained and provided with naloxone, bystanders can effectively prevent overdoses in the community^a^	6.06	5.31	5.26

^a^All items have responses ranging from (1: Extremely unbelievable to 7: Extremely believable)

Profile 1 (“*More Aligned*”; *n* = 246, 35.0%) tended not to believe the risk compensation statements about naloxone access resulting in more opioid use (mean = 1.96) or reduced likelihood of seeking treatment (mean = 2.04), or the statement that opioid users can get high on take-home naloxone (mean = 1.99). Members reported moderate believability in overdose inevitability, that opioid users who overdose once will usually overdose again (mean = 4.46) and that non-fatal overdose usually precedes fatal overdose within 12 months (mean = 3.94). They reported very high believability for the statement that trained, provisioned bystanders can effectively prevent overdoses (mean = 6.06).

Profile 2 (“*Moderately Aligned*”; *n* = 351, 50.0%) reported moderate believability for most statements, with believability means ranging from 3.75 (getting high on take-home naloxone) to 4.83 (users who overdose once will usually overdose again). Members also reported high believability that bystanders can effectively prevent overdoses (mean = 5.31).

Profile 3 (“*Less Aligned*”; *n* = 105, 15.0%) reported high or very high believability for all six narrative statements, with scores ranging from 5.00 (getting high on take-home naloxone) to 5.98 (users less likely to seek treatment if they have access to naloxone).

### Regression analysis

Table 3 shows the adjusted relative risk ratio (RRR) of a participant being categorized into Profile 2 and/or Profile 3 compared to Profile 1 – in other words, the RRR values indicate the likelihood of being *moderately aligned* (Profile 2) or *less aligned* (Profile 3) with current scientific evidence rather than being *more aligned* (Profile 1). Links to full output and model fit parameters are provided.

**Table 3:** Logistic Regression Output (Profile 1 = Referent)

**Likelihood of Being Classified into Profile 2**				
**Variable**	**RRR**	**SE**	***p***	**95% CI**
Gender	-	-	-	-
* Male (ref)*	-	-	-	-
* Female*	1.36	0.30	.155	0.89 – 2.08
* Non-binary*	0.40	0.32	.251	0.09 – 1.90
* Transgender*	2.44	3.73	.559	0.12 – 48.77
Hispanic, Latino, or Spanish Origin (yes = ref)	0.63	0.27	.276	0.28 – 1.45
Race	-	-	-	-
* White (ref)*	-	-	-	-
* Black or African American*	1.28	0.41	.206	0.20 – 1.42
American Indian or Alaska Native*	-	-	-	-
* Asian*	1.03	0.39	.947	0.49 – 2.16
* Multiple races*	0.53	0.27	.206	0.20 – 1.42
* Other*	1.40	1.71	.781	0.13 – 15.36
Age (years)	0.99	0.01	.362	0.98 – 1.01
Education Level	-	-	-	-
* No diploma (did not finish high school)*	0.67	0.64	.674	0.10 – 4.36
* High school graduate, GED, or equivalent*	1.44	0.50	.287	0.73 – 2.83
* Some college, no degree*	1.19	0.30	.506	0.72 – 1.96
* Associate degree or bachelor’s degree (ref)*	-	-	-	-
* Master’s degree*	0.80	0.25	.475	0.43 – 1.49
* Professional or doctoral degree*	0.94	0.61	.919	0.26 – 3.36
Residence	-	-	-	-
* A large city (*> *250,000 people) (ref)*	-	-	-	-
* A midsized city (100,000 to 249,999 people)*	0.67	0.64	.674	0.10 – 4.36
* A large town (25,000 to 99,999 people*)	1.91	0.57	.030	1.06 – 3.45
* A small town (2,500 to 24,999 people*)	0.89	0.28	.702	0.48 – 1.64
* A suburb of a large city*	1.44	0.50	.295	0.73 – 2.83
* A rural area (non-farm*)	0.82	0.39	.678	0.33 – 2.07
* A rural area (farm)*	0.40	0.41	.377	0.05 – 3.06
Religious Commitment (1: Low to 10: High)	1.05	0.04	.226	0.97 – 1.13
Political Orientation (1: Liberal to 10: Conservative)	1.41	0.10	< .001	1.23 – 1.63
Political Party	-	-	-	-
* Republican (ref)*	-	-	-	-
* Democrat*	1.30	0.62	.575	0.52 – 3.29
* Other*	1.59	0.68	.284	0.68 – 3.68
Area of Employment	-	-	-	-
* Medicine (e.g., physician, nurse, physician assistant) (ref)*	-	-	-	-
* Behavioral health (e.g., social worker, counselor, psychologist)*	0.79	0.55	.732	0.20 – 3.12
* First responder (e.g., law enforcement, fire, EMS)*	1.25	1.63	.866	0.10 – 16.07
* Pharmacy (e.g., pharmacist, pharmacy technician)*	1.26	1.36	.831	0.15 – 10.47
* Community service (e.g., parks and recreation, librarian, bus driver, or another public-facing role)*	1.02	0.62	.980	0.31 – 3.33
* Currently unemployed*	0.70	0.31	.428	0.29 – 1.68
* None of the above*	0.88	0.41	.782	0.35 – 2.18
Received prior training about prescription drug abuse (yes = ref)	1.14	0.33	.649	0.64 – 2.03
Received educational training about naloxone (Narcan) (yes = ref)	1.92	0.69	.069	0.95 – 3.87
Use of opioids in the past month (non-medical)	-	-	-	-
* Never (ref)*	-	-	-	-
* Once or twice*	0.95	0.72	.947	0.22 – 4.18
Monthly*	-	-	-	-
* Weekly*	0.41	0.58	.532	0.03 – 6.60
Daily or almost daily*	-	-	-	-
Use of any drug by injection in the past month (non-medical)	-	-	-	-
* No, never (ref)*	-	-	-	-
* Yes, at least once in the past 6 months*	2.04	5.49	.792	0.01 – 401.28
Ever received naloxone to reverse an overdose (yes = ref)	3.20	4.12	.367	0.26 – 39.87
Ever personally witnessed an opioid overdose (yes = ref)	0.96	0.38	.917	0.44 – 2.07
Trust in Science (1: Low to 5: High)	0.36	0.08	< .001	0.24 – 0.54
**Likelihood of Being Classified into Profile 3**				
**Variable**	**RRR**	**SE**	***p***	**95% CI**
Gender	-	-	-	-
* Male (ref)*	-	-	-	-
* Female*	1.76	0.52	.059	0.98 – 3.15
* Non-binary*	0.83	1.01	.876	0.08 – 9.04
* Transgender*	0.00	0.01	.996	-
Hispanic, Latino, or Spanish Origin (yes = ref)	0.24	0.12	.005	0.09 – 0.66
Race	-	-	-	-
* White (ref)*	-	-	-	-
* Black or African American*	1.04	0.46	.936	0.44 – 2.45
American Indian or Alaska Native*	-	-	-	-
* Asian*	1.09	0.58	.871	0.39 – 3.08
* Multiple races*	0.65	0.48	.560	0.16 – 2.73
* Other*	1.83	2.50	.657	0.13 – 26.46
Age (years)	0.98	0.01	.218	0.96 – 1.01
Education Level	-	-	-	-
* No diploma (did not finish high school)*	0.93	1.11	.948	0.09 – 9.78
* High school graduate, GED, or equivalent*	3.32	1.43	.005	1.43 – 7.71
* Some college, no degree*	1.88	0.66	.071	0.95 – 3.73
* Associate degree or bachelor’s degree (ref)*	-	-	-	-
* Master’s degree*	1.34	0.64	.532	0.53 – 3.40
* Professional or doctoral degree*	0.61	0.72	.676	0.06 – 6.04
Residence	-	-	-	-
* A large city (*> *250,000 people) (ref)*	-	-	-	-
* A midsized city (100,000 to 249,999 people)*	1.28	0.53	.550	0.57 – 2.87
* A large town (25,000 to 99,999 people*)	1.76	0.75	.185	0.76 – 4.04
* A small town (2,500 to 24,999 people*)	1.09	0.48	.848	0.46 – 2.59
* A suburb of a large city*	1.25	0.62	.648	0.48 – 3.28
* A rural area (non-farm*)	1.02	0.67	.974	0.28 – 3.69
* A rural area (farm)*	1.12	1.42	.930	0.93 – 13.39
Religious Commitment (1: Low to 10: High)	1.01	0.05	.899	0.91 – 1.12
Political Orientation (1: Liberal to 10: Conservative)	1.39	0.73	< .001	1.35 – 1.95
Political Party	-	-	-	-
* Republican (ref)*	-	-	-	-
* Democrat*	2.25	1.34	.172	0.70 – 7.23
* Other*	1.39	0.73	.537	0.49 – 3.91
Area of Employment	-	-	-	-
* Medicine (e.g., physician, nurse, physician assistant) (ref)*	-	-	-	-
* Behavioral health (e.g., social worker, counselor, psychologist)*	0.59	0.58	.589	0.09 – 4.01
* First responder (e.g., law enforcement, fire, EMS)*	2.48	3.73	.547	0.13 – 47.26
* Pharmacy (e.g., pharmacist, pharmacy technician)*	-	-	-	-
* Community service (e.g., parks and recreation, librarian, bus driver, or another public-facing role)*	0.40	0.36	.312	0.07 – 2.37
* Currently unemployed*	0.54	0.33	.309	0.17 – 1.76
* None of the above*				
Received prior training about prescription drug abuse (yes = ref)	1.02	0.40	.968	0.47 – 2.21
Received educational training about naloxone (Narcan) (yes = ref)	3.37	1.83	.025	1.16 – 9.77
Use of opioids in the past month (non-medical)	-	-	-	-
* Never (ref)*	-	-	-	-
* Once or twice*	4.79	3.99	.060	0.94 – 24.52
* Monthly*^a^	-	-	-	-
* Weekly*^a^	-	-	-	-
* Daily or almost daily*^a^	-	-	-	-
Use of any drug by injection in the past month (non-medical)	-	-	-	-
* No, never (ref)*	-	-	-	-
* Yes, at least once in the past 6 months*^a^	-	-	-	-
Ever received naloxone to reverse an overdose (yes = ref)^a^	-	-	-	-
Ever personally witnessed an opioid overdose (yes = ref)	1.14	0.65	.816	0.37 – 3.50
Trust in Science (1: Low to 5: High)	0.21	0.06	< .001	0.12 – 0.36

^a^Insufficient responses within this category to generate parameter estimates

Only two variables were clearly associated with being categorized into both Profile 2 and Profile 3 rather than Profile 1. First, conservative political orientation was associated with higher likelihood of belonging to a *moderately aligned* or *less aligned* latent profile. For each one-point movement toward conservative political orientation on a 10-point scale, participants were 1.41 times (*95%CI*: 1.23–1.63) more likely to be categorized as *moderately aligned* and 1.62 times (*95%CI*: 1.35–1.95) more likely to be categorized as *less aligned* with current scientific evidence, versus being *more aligned*. Second, higher trust in science was associated with lower likelihood of belonging to a *moderately aligned* or *less aligned* latent profile. For each one-point movement toward increased trust in science and scientists on a five-point scale, participants were 2.78 times (*95%CI*: 1.84–4.18) less likely to be categorized as *moderately aligned* and 4.76 times (*95%CI*: 2.78–8.33) less likely to be categorized as *less aligned* with current scientific evidence, versus being *more aligned*.

One additional variable was associated only with belonging to Profile 2 (*moderately aligned*) compared to Profile 1 (*more aligned*). Individuals living in a large town were 1.91 times (*95%CI*: 1.06–3.45) more likely than those living in a large city to be categorized as *moderately aligned* with current scientific evidence versus being *more aligned*.

In addition, three variables were associated only with belonging to Profile 3 (*less aligned*) compared to Profile 1 (*more aligned*). High school graduates or those with a GED were 3.32 times (*95%CI*: 1.43–7.71) more likely than those with a bachelor’s or associate degree to be *less aligned* with current scientific evidence, participants not identifying as Hispanic, Latino, or of Spanish origin were 4.17 times (*95%CI*: 1.52–11.11) more likely to be *less aligned* with current scientific evidence, and those who had not previously received training about naloxone were 3.37 times (*95%CI*: 1.16–9.77) more likely to be *less aligned* with current scientific evidence.

## Discussion

The believability of different narratives around naloxone and overdose varied widely among US laypersons. Consistent with our hypothesis, multiple latent profiles were identifiable using the narrative believability scores, though our expectations about profile sizes and belief patterns were not validated.

### Belief that bystander overdose prevention works

All three latent profiles reported high to very high believability for the statement that trained bystanders can prevent overdose with naloxone. This is consistent with much of the national messaging on naloxone from the past several years (e.g., the U.S. Surgeon General’s Advisory on Naloxone and Opioid Overdose) [43] as well as studies on such programs [28, 29]. However, we cannot assume that people who believe bystander overdose prevention with naloxone is effective will always support such a program, since some profiles simultaneously endorsed less supportive beliefs.

### Interpreting latent profiles

While the overall sample displayed at least some uniformity in perceptions of bystander overdose prevention and repeated overdose, the latent profiles were most clearly separated by differences in believability of statements proposing (a) unsupported ideas about risk compensation and (b) misinformation about getting high on naloxone (see Fig. 1).

Regression analysis found that profiles *moderately aligned* and *less aligned* with current evidence were strongly associated with both lower trust in science and greater political conservatism, though not with political party affiliation. This association between trust in science and belief in unsupported statements mirrors what we observed regarding COVID-19 [30, 31].

We now question whether these variables may approximate exposure to certain kinds of information about naloxone. One direction for future research would investigate the possibility of a continued-influence effect (CIE) [44] affecting unsupported statements about naloxone. While CIE, as referenced, pertains to retracted misinformation, such a finding may also be applicable when studies advancing one theory are more visible to laypersons than studies disagreeing with that theory. We note, for example, popular articles that are retracted often receive more popular attention than the retractions [45]. In the case of naloxone, a substantial public debate emerged over a study of the ‘moral hazard’ of naloxone [46]. The 2018 pre-print igniting the debate has been mentioned in 60 news articles (though sometimes for tangential topics, like face masks for COVID-19) and 1,503 tweets [47], whereas a systematic review with different conclusions, published November 2021, has not yet been mentioned in any news media captured by PlumX, and has been mentioned in 164 tweets [21].

Consistent with a recent systematic review of reviews finding improved knowledge and attitudes associated with OEND programs [2], having received training about naloxone was associated with reduced likelihood of being categorized in Profile 3, and possibly associated with reduced likelihood of being in Profile 2 (though *p* = 0.069). One promising direction for research would be to study the potential for OEND programs to attenuate belief in unsupported statements about naloxone and overdose, but such research might also benefit from modifying OEND programs to explicitly address data on repeated overdose (for reasons described subsequently).

There were several individual findings in the regression model (ethnicity, education, and residence) for which we do not have working theories. We encourage exploration of these findings using our data but do not propose hypotheses thereabout. Such exploration is potentially important given evidence of differential overdose mortality risk by subgroups such as race and ethnicity [48].

### Inevitability of repeated overdoses

We were surprised to find that most participants, regardless of latent profile, found statements about overdose inevitability to be at least moderately believable. Research does indicate that there is a meaningful, increased risk of overdose in the year following a nonfatal opioid overdose. Estimated percentages of repeated overdose from studies with varying, but robust, methodologies include 18.9% (within a year; national longitudinal cohort of Medicaid patients ages 18–64) [23], 14.9% (within 2.5 years, retrospective cohort of patients ages 18–64 in a Western PA health system) [27], and 7% (within a median of 299 days; commercially insured patients ages 18–64 who were undergoing long-term opioid therapy for non-cancer pain) [25]. It is clear that a nonfatal opioid overdose should be treated as a significant risk factor for subsequent harm. However, that concept is separate from the belief that someone who nonfatally overdoses *usually* will overdose again, which is contradicted by data. It is especially notable that even Profile 1 (*More Aligned*), members of which generally reflected current scientific evidence, indicated moderate believability for statements about overdose inevitability. We encourage further research about this observation and question whether some people who are aware of acute risks of overdose may conflate the concepts of “likely to overdose” and “more likely to overdose than someone who has never overdosed before.”

## Limitations

We note several limitations to this work. First, while some narrative statements about overdose and naloxone were from established scales [17], others were based on research findings, and were proposed using approaches from prior studies [30, 31], but had not been validated, per se. Second, as with most research, our models are subject to omitted variable bias [49]. Third, some factors that we thought might be important covariates (such as personal experience with a syringe service program) were extremely uncommon in our sample (< 1%), and while we still controlled for that information, we could not establish clear model parameters for those variables. Fourth, our cross-sectional design does not allow strong inference of causality.

Finally, while Prolific can produce a nationally representative US sample by race, gender, and age [33], some questions remain about generalizability. The sample distribution of political party identification was skewed toward Democrats, whereas the national US population distribution on the Gallup Poll for Quarter 4, 2021 (the quarter when the study was conducted) showed an even split at 28%/28% for Democrats and Republicans [50]. This may have affected the aggregate level of believability for some statements about naloxone and overdose, though the clustering observed through the LPA analysis would not likely be substantively affected. In addition, the sample was still crowdsourced, meaning it was limited to members of the Prolific panel which may, for example, overrepresent those with regular access to the Internet. At the same time, research on crowdsourced samples using a more common platform, Mechanical Turk (MTurk), has suggested that such samples may effectively produce representative data for some populations [51] and characteristics [52]. When quality control approaches are utilized, data from MTurk can broadly mirror the distribution of population-level data for risk assessments like the US Alcohol Use Disorders Identification Test [34, 53]. In addition, scholars have successfully replicated ‘in-person’ experimental research using MTurk [54]. Thus, it is reasonable to infer some level of generalizability, especially given the nationally representative draw of race, gender, and age cross-sections.

## Conclusions

Within the bounds of the noted limitations as well as a reasonable interpretation of results from a single cross-sectional study, we pose several ideas for consideration. Persons interested in advancing naloxone policy should consider that for two latent profiles representing more than half the sample, laypersons believed that OEND programs work *and* that there are meaningful social ‘costs’ (i.e., unsupported ideas about risk compensation). It is important to recognize the possibility of these ideas simultaneously influencing a person’s support for OEND programs in their own community but adding weight in opposite directions. Further, statements about the inevitability of repeated overdose were surprisingly believable to laypersons regardless of their belief in other unsupported statements. While the relationship of such beliefs to support/opposition to layperson naloxone access should be studied further, individuals developing messaging and training on naloxone and overdose might consider whether proactively to provide evidence-based messaging on those topics as prophylaxis. More broadly, the similarity in belief profiles between naloxone and COVID-19, as well as the consistent association between profile membership and trust in science, suggests the possibility that trust in science is a topic-invariant variable of interest in understanding belief in unsupported statements in general. We strongly encourage more research in this area, particularly before making any definitive claims.

## Supplementary Information

**Additional file 1.** (DOCX 13 kb)

**Additional file 2.** (DOCX 13 kb)

**Additional file 3.** (DOCX 3180 kb)

**Additional file 4.** (PDF 182 kb)

## Data Availability

All code and outputs are included in this published article as additional files. Raw study data are available through the Open Science Framework Storage System (https://osf.io/2d856/). Cleaned study data are also available through the Open Science Framework Storage System (https://osf.io/ma2v6/).

## Abbreviations

AIC: Akaike information criterion
BIC: Bayesian information criterion
CIE: Continued-influence effect
LPA: Latent profile analysis
MTurk: Amazon’s Mechanical Turk
OEND: Overdose education, naloxone distribution
USD: United States dollar
VPN: Virtual private network
