# Supplementary material for: Beliefs and misperceptions about naloxone and overdose among U.S. laypersons: a cross-sectional study
Source: BMC Public Health. 2022 May 10;22:924. doi: 10.1186/s12889-022-13298-3 (PMC9086153; doi:10.1186/s12889-022-13298-3)
Supplement: Supplementary file 1 — Additional file 1. (DOCX 13 kb) [file 12889_2022_13298_MOESM1_ESM.docx]

***Remove cases that were rejected due to quality checks.

Alter Type RejectAttention1 (f8.2).

Alter Type RejectHonesty (f8.2).

Alter Type RejectVPN (f8.2).

Alter Type RejectAttention2 (f8.2).

Execute.

Select if (RejectAttention1 ne 1) and (RejectHonesty ne 1) and (RejectVPN ne 1) and (RejectAttention2 ne 1).

Execute.

***Remove people who failed quality checks but then immediately quit (closed browser).

Select If (Latveria=1).

Select If (Trust1_1_3 = 3).

Execute.

***Remove cases that never entered the survey after the SIS.

Select If ProlificID ne "".

Execute.

***Remove cases that timed out or quit before the naloxone questions.

Select if not Sysmis(Narratives_1).

Execute.

**Create a single race variable from the different questions.

Compute Race=0.

If (Race_1=1) and Sysmis(Race_2) and Sysmis(Race_3) and Sysmis(Race_4) and Sysmis(Race_5) and Sysmis(Race_6) Race=1.

If (Race_2=1) and Sysmis(Race_1) and Sysmis(Race_3) and Sysmis(Race_4) and Sysmis(Race_5) and Sysmis(Race_6) Race=2.

If (Race_3=1) and Sysmis(Race_1) and Sysmis(Race_2) and Sysmis(Race_4) and Sysmis(Race_5) and Sysmis(Race_6) Race=3.

If (Race_4=1) and Sysmis(Race_1) and Sysmis(Race_2) and Sysmis(Race_3) and Sysmis(Race_5) and Sysmis(Race_6) Race=4.

If (Race_5=1) and Sysmis(Race_1) and Sysmis(Race_2) and Sysmis(Race_3) and Sysmis(Race_4) and Sysmis(Race_6) Race=5.

If (Race_6=1) and Sysmis(Race_1) and Sysmis(Race_2) and Sysmis(Race_3) and Sysmis(Race_4) and Sysmis(Race_5) Race=6.

Execute.

Value Labels Race

'0' Multiple races

'1' White

'2' Black or African American

'3' American Indian or Alaska Native

'4' Asian

'5' Native Hawaiian or Pacific Islander

'6' Other.

Execute.

**Compute Trust in Science Variable.

*Recode variable per Nadelson et al.

Recode Trust1_1_1 Trust1_1_2 Trust1_1_4 Trust1_1_5 Trust1_1_7 Trust1_1_9 Trust1_2_3 Trust1_2_7 Trust1_2_8 Trust1_2_9 Trust1_2_10 Trust1_2_11 (5=1) (4=2) (2=4) (1=5).

Execute.

Compute Trust=((Trust1_1_1+Trust1_1_2)+Sum(Trust1_1_4 to Trust1_2_11))/21.

Execute.
