# Supplementary material for: Beliefs and misperceptions about naloxone and overdose among U.S. laypersons: a cross-sectional study
Source: BMC Public Health. 2022 May 10;22:924. doi: 10.1186/s12889-022-13298-3 (PMC9086153; doi:10.1186/s12889-022-13298-3)
Supplement: Supplementary file 2 — Additional file 2. (DOCX 13 kb) [file 12889_2022_13298_MOESM2_ESM.docx]

**Deviations from Proposed Plan**

Modification One: In our exploratory aim (#3) about the regression model, we indicated that we would include all variables from the “Variables” section in the model. However, the variable about having received services from a harm reduction program was nearly a constant, with only one person indicating they had done so. Inclusion of this variable in the model prevented convergence and so we eliminated it from the analyses.

Modification Two: Also, in our exploratory aim (#3), we proposed contrasting the profiles against the most populated profile; in this case, that would be Profile 2. However, that aim was inexpertly written, as we used language that presumed that hypothesis 2a would be upheld, which it was not. Our intention was to contrast the profile most strongly reporting a scientific belief pattern (Profile 1) against the others, but in this study, that profile was *not* the largest.
