# Supplementary material for: Beliefs and misperceptions about naloxone and overdose among U.S. laypersons: a cross-sectional study
Source: BMC Public Health. 2022 May 10;22:924. doi: 10.1186/s12889-022-13298-3 (PMC9086153; doi:10.1186/s12889-022-13298-3)
Supplement: Supplementary file 3 — Additional file 3. (DOCX 3180 kb) [file 12889_2022_13298_MOESM3_ESM.docx]

Mplus VERSION 8.3

MUTHEN & MUTHEN

01/06/2022 10:40 AM

INPUT INSTRUCTIONS

Data:

File is ~\Naloxone data for LPA adjusted_clean.csv ;

Variable:

Names are

id Narratives_1 Narratives_2 Narratives_3 Narratives_4 Narratives_5

Narratives_6 Religious Political Party Gender Ethnicity Race Age Education;

Missing are all (9999) ;

Usevariables are Narratives_1 Narratives_2 Narratives_3 Narratives_4 Narratives_5

Narratives_6;

IDVARIABLE is id;

Missing are all (99) ;

CLASSES = c(3);

Analysis:

TYPE=MIXTURE ;

ESTIMATOR = MLR;

STARTS = 1000 250;

STITERATIONS = 500;

LRTSTARTS = 2 1 50 10;

MODEl:

%OVERALL%

[ Narratives_1 Narratives_2 Narratives_3 Narratives_4 Narratives_5

Narratives_6 ];

Narratives_1 Narratives_2 Narratives_3 Narratives_4 Narratives_5

Narratives_6 (Var1-Var6);

OUTPUT: TECH1 TECH4 TECH8 TECH11 TECH14;

SAVEDATA:

FILE IS LPA_MISINFO_3_unadjusted.dat;

SAVE = CPROBABILITIES;

Plot:

type = plot3;

series =

Narratives_1 Narratives_2 Narratives_3 Narratives_4 Narratives_5

Narratives_6 (*);

*** WARNING in VARIABLE command

Note that only the first 8 characters of variable names are used in the output.

Shorten variable names to avoid any confusion.

*** WARNING in MODEL command

There are more equality/parameter labels given than there are parameters.

Some labels will not be used.

NARRATIVES_6 (VAR1-VAR6)

*** WARNING in MODEL command

All variables are uncorrelated with all other variables within class.

Check that this is what is intended.

*** WARNING in PLOT command

Note that only the first 8 characters of variable names are used in plots.

If variable names are not unique within the first 8 characters, problems

may occur.

4 WARNING(S) FOUND IN THE INPUT INSTRUCTIONS

SUMMARY OF ANALYSIS

Number of groups 1

Number of observations 702

Number of dependent variables 6

Number of independent variables 0

Number of continuous latent variables 0

Number of categorical latent variables 1

Observed dependent variables

Continuous

NARRATIVES NARRATIVES NARRATIVES NARRATIVES NARRATIVES NARRATIVES

Categorical latent variables

C

Variables with special functions

ID variable ID

Estimator MLR

Information matrix OBSERVED

Optimization Specifications for the Quasi-Newton Algorithm for

Continuous Outcomes

Maximum number of iterations 100

Convergence criterion 0.100D-05

Optimization Specifications for the EM Algorithm

Maximum number of iterations 500

Convergence criteria

Loglikelihood change 0.100D-06

Relative loglikelihood change 0.100D-06

Derivative 0.100D-05

Optimization Specifications for the M step of the EM Algorithm for

Categorical Latent variables

Number of M step iterations 1

M step convergence criterion 0.100D-05

Basis for M step termination ITERATION

Optimization Specifications for the M step of the EM Algorithm for

Censored, Binary or Ordered Categorical (Ordinal), Unordered

Categorical (Nominal) and Count Outcomes

Number of M step iterations 1

M step convergence criterion 0.100D-05

Basis for M step termination ITERATION

Maximum value for logit thresholds 15

Minimum value for logit thresholds -15

Minimum expected cell size for chi-square 0.100D-01

Maximum number of iterations for H1 2000

Convergence criterion for H1 0.100D-03

Optimization algorithm EMA

Random Starts Specifications

Number of initial stage random starts 1000

Number of final stage optimizations 250

Number of initial stage iterations 500

Initial stage convergence criterion 0.100D+01

Random starts scale 0.500D+01

Random seed for generating random starts 0

Input data file(s)

~\Naloxone data for LPA adjusted_clean.csv

Input data format FREE

SUMMARY OF DATA

Number of missing data patterns 1

Number of y missing data patterns 1

Number of u missing data patterns 0

COVARIANCE COVERAGE OF DATA

Minimum covariance coverage value 0.100

PROPORTION OF DATA PRESENT FOR Y

Covariance Coverage

NARRATIV NARRATIV NARRATIV NARRATIV NARRATIV

________ ________ ________ ________ ________

NARRATIV 1.000

NARRATIV 1.000 1.000

NARRATIV 1.000 1.000 1.000

NARRATIV 1.000 1.000 1.000 1.000

NARRATIV 1.000 1.000 1.000 1.000 1.000

NARRATIV 1.000 1.000 1.000 1.000 1.000

Covariance Coverage

NARRATIV

________

NARRATIV 1.000

UNIVARIATE SAMPLE STATISTICS

UNIVARIATE HIGHER-ORDER MOMENT DESCRIPTIVE STATISTICS

Variable/ Mean/ Skewness/ Minimum/ % with Percentiles

Sample Size Variance Kurtosis Maximum Min/Max 20%/60% 40%/80% Median

NARRATIVES_1 3.507 0.305 1.000 13.11% 2.000 3.000 3.000

702.000 3.002 -0.854 7.000 6.27% 4.000 5.000

NARRATIVES_2 3.758 0.051 1.000 10.97% 2.000 3.000 4.000

702.000 2.993 -0.995 7.000 5.98% 4.000 5.000

NARRATIVES_3 4.872 -0.477 1.000 2.42% 4.000 5.000 5.000

702.000 2.066 -0.031 7.000 14.25% 5.000 6.000

NARRATIVES_4 4.346 -0.223 1.000 2.42% 3.000 4.000 4.000

702.000 1.770 -0.067 7.000 4.99% 5.000 5.000

NARRATIVES_5 3.328 0.298 1.000 21.94% 1.000 2.000 3.000

702.000 3.354 -1.005 7.000 5.41% 4.000 5.000

NARRATIVES_6 5.566 -0.930 1.000 1.14% 4.000 5.000 6.000

702.000 1.904 0.494 7.000 31.48% 6.000 7.000

RANDOM STARTS RESULTS RANKED FROM THE BEST TO THE WORST LOGLIKELIHOOD VALUES

Final stage loglikelihood values at local maxima, seeds, and initial stage start numbers:

-7428.905 86698 893

-7428.905 461687 833

-7428.905 857799 315

-7428.905 213532 503

-7428.905 462228 298

-7428.905 467561 819

-7428.905 954914 911

-7428.905 957392 79

-7428.905 49293 707

-7428.905 301428 885

-7428.905 311214 64

-7428.905 737601 891

-7428.905 794236 127

-7428.905 49910 829

-7428.905 30098 209

-7428.905 22075 659

-7428.905 369602 146

-7428.905 1548 384

-7428.905 641794 591

-7428.905 300697 820

-7428.905 722748 346

-7428.905 46437 153

-7428.905 700846 867

-7428.905 59963 670

-7428.905 814975 129

-7428.905 783110 72

-7428.905 193847 354

-7428.905 846194 93

-7428.905 58507 793

-7428.905 631413 439

-7428.905 772131 407

-7428.905 995875 547

-7428.905 209031 632

-7428.905 216565 474

-7428.905 188498 258

-7428.905 436892 565

-7428.905 297518 166

-7428.905 928287 197

-7428.905 358074 560

-7428.905 717754 180

-7428.905 484687 306

-7428.905 668003 647

-7428.905 662983 666

-7428.905 148918 682

-7428.905 937588 293

-7428.905 570681 777

-7428.905 848590 694

-7428.905 15715 274

-7428.905 206099 363

-7428.905 520865 763

-7428.905 830392 35

-7428.905 194143 510

-7428.905 820977 776

-7428.905 84013 598

-7428.905 484501 163

-7428.905 119513 821

-7428.905 790059 469

-7428.905 677720 681

-7428.905 926283 269

-7428.905 765586 709

-7428.905 312587 775

-7428.905 979450 913

-7428.905 436460 89

-7428.905 395754 388

-7428.905 57226 208

-7428.905 408713 450

-7428.905 714455 476

-7428.905 948615 140

-7428.905 548245 818

-7428.905 121425 511

-7428.905 22089 143

-7428.905 888905 444

-7428.905 443442 380

-7428.905 996231 310

-7428.905 98068 998

-7428.905 692169 308

-7428.905 645052 910

-7428.905 130541 896

-7428.905 165853 105

-7428.905 602797 336

-7428.905 152496 123

-7428.905 392407 221

-7428.905 264901 634

-7428.905 977800 853

-7428.905 878749 728

-7428.905 887676 22

-7428.905 425982 485

-7428.905 569833 85

-7428.905 370957 554

-7428.905 749635 420

-7428.905 263049 454

-7428.905 85462 51

-7428.905 804660 260

-7428.905 760850 739

-7428.905 65952 933

-7428.905 781489 627

-7428.905 889774 954

-7428.905 539389 544

-7428.905 552272 654

-7428.905 782179 835

-7428.905 427006 839

-7428.905 650371 14

-7428.905 712531 631

-7428.905 379729 512

-7428.905 423661 875

-7428.905 109946 771

-7428.905 775881 778

-7428.905 926797 406

-7428.905 326091 759

-7428.905 545108 667

-7428.905 848356 602

-7428.905 506113 895

-7428.905 331681 549

-7428.905 420910 928

-7428.905 972873 157

-7428.905 137888 901

-7428.905 284384 600

-7428.905 40327 971

-7428.905 618000 190

-7428.905 884242 957

-7428.905 193042 316

-7428.905 569131 26

-7428.905 923437 398

-7428.905 179022 687

-7428.905 17359 227

-7428.905 478341 731

-7428.905 850545 357

-7428.905 931558 898

-7428.905 783102 433

-7428.905 945065 255

-7428.905 302046 863

-7428.905 247224 94

-7428.905 840031 276

-7428.905 424223 900

-7428.905 72344 897

-7428.905 496762 870

-7428.905 930323 267

-7428.905 65651 214

-7428.905 321390 133

-7428.905 396795 323

-7428.905 645664 39

-7428.905 479273 156

-7428.905 298201 903

-7428.905 788796 145

-7428.905 392766 331

-7428.905 92091 649

-7428.905 313407 132

-7428.905 384199 882

-7428.905 252949 487

-7428.905 79945 395

-7428.905 490123 995

-7428.905 432148 30

-7428.905 588699 828

-7428.905 123985 248

-7428.905 140442 500

-7428.905 569338 755

-7428.905 422103 62

-7428.905 995913 787

-7428.905 264521 929

-7428.905 267983 228

-7428.905 539751 459

-7428.905 745972 521

-7428.905 190339 102

-7428.905 642386 662

-7428.905 21132 351

-7428.905 96941 890

-7428.905 679615 942

-7428.905 735928 916

-7428.905 88437 761

-7428.905 195353 225

-7428.905 376411 473

-7428.905 847088 750

-7428.905 574942 558

-7428.905 327475 518

-7428.905 68985 17

-7428.905 74518 927

-7428.905 922596 456

-7428.905 25127 107

-7428.905 185071 370

-7428.905 350608 334

-7428.905 371246 101

-7428.905 810594 845

-7428.905 177175 851

-7428.905 876943 650

-7428.905 971853 402

-7428.905 97158 205

-7428.905 995249 525

-7428.905 638977 643

-7428.905 36714 201

-7428.905 440395 917

-7428.905 459221 652

-7428.905 793487 595

-7428.905 501995 791

-7428.905 77571 980

-7428.905 307843 664

-7428.905 94573 983

-7428.905 760878 249

-7428.905 107446 12

-7428.905 319575 499

-7428.905 263221 447

-7428.905 728038 177

-7428.905 648035 836

-7428.905 188640 451

-7428.905 848969 173

-7428.905 405371 569

-7428.905 67009 564

-7428.905 399508 415

-7428.905 59674 240

-7428.905 78862 529

-7428.905 442072 625

-7428.905 902278 21

-7428.905 161421 519

-7428.905 696773 80

-7428.905 724519 333

-7428.905 595153 230

-7428.905 349562 359

-7428.905 699834 723

-7428.905 507154 387

-7428.905 134830 780

-7428.905 301180 236

-7428.905 816435 914

-7428.905 248742 556

-7428.905 605358 321

-7428.905 278692 342

-7428.905 876056 1000

-7428.905 871722 355

-7428.905 965639 463

-7428.905 830292 527

-7428.905 576726 280

-7428.905 82357 339

-7428.905 392751 480

-7428.905 197223 332

-7428.905 496710 386

-7428.905 922042 492

-7428.905 792389 746

-7428.905 589483 950

-7428.905 308582 752

-7428.905 377584 630

-7428.905 887580 493

-7428.905 414284 158

-7428.905 347222 533

-7428.905 243346 930

-7428.905 418686 338

-7428.905 435506 988

-7428.905 853195 431

-7428.905 97300 640

-7428.905 746978 410

-7428.905 314084 81

-7428.905 416463 467

-7428.905 588923 606

THE BEST LOGLIKELIHOOD VALUE HAS BEEN REPLICATED. RERUN WITH AT LEAST TWICE THE

RANDOM STARTS TO CHECK THAT THE BEST LOGLIKELIHOOD IS STILL OBTAINED AND REPLICATED.

THE MODEL ESTIMATION TERMINATED NORMALLY

MODEL FIT INFORMATION

Number of Free Parameters 26

Loglikelihood

H0 Value -7428.905

H0 Scaling Correction Factor 1.1933

for MLR

Information Criteria

Akaike (AIC) 14909.810

Bayesian (BIC) 15028.212

Sample-Size Adjusted BIC 14945.657

(n* = (n + 2) / 24)

FINAL CLASS COUNTS AND PROPORTIONS FOR THE LATENT CLASSES

BASED ON THE ESTIMATED MODEL

Latent

Classes

1 247.24131 0.35220

2 341.28348 0.48616

3 113.47521 0.16165

FINAL CLASS COUNTS AND PROPORTIONS FOR THE LATENT CLASSES

BASED ON ESTIMATED POSTERIOR PROBABILITIES

Latent

Classes

1 247.24131 0.35220

2 341.28348 0.48616

3 113.47521 0.16165

FINAL CLASS COUNTS AND PROPORTIONS FOR THE LATENT CLASSES

BASED ON THEIR MOST LIKELY LATENT CLASS MEMBERSHIP

Class Counts and Proportions

Latent

Classes

1 246 0.35043

2 351 0.50000

3 105 0.14957

CLASSIFICATION QUALITY

Entropy 0.772

Average Latent Class Probabilities for Most Likely Latent Class Membership (Row)

by Latent Class (Column)

1 2 3

1 0.917 0.083 0.000

2 0.062 0.880 0.059

3 0.000 0.115 0.885

Classification Probabilities for the Most Likely Latent Class Membership (Column)

by Latent Class (Row)

1 2 3

1 0.912 0.088 0.000

2 0.060 0.905 0.035

3 0.000 0.181 0.819

Logits for the Classification Probabilities for the Most Likely Latent Class Membership (Column)

by Latent Class (Row)

1 2 3

1 13.724 11.381 0.000

2 0.525 3.241 0.000

3 -11.130 -1.508 0.000

MODEL RESULTS

Two-Tailed

Estimate S.E. Est./S.E. P-Value

Latent Class 1

Means

NARRATIVES 1.955 0.114 17.139 0.000

NARRATIVES 2.039 0.099 20.667 0.000

NARRATIVES 4.460 0.112 39.652 0.000

NARRATIVES 3.938 0.108 36.588 0.000

NARRATIVES 1.986 0.119 16.683 0.000

NARRATIVES 6.061 0.096 62.902 0.000

Variances

NARRATIVES 1.164 0.146 7.991 0.000

NARRATIVES 1.028 0.174 5.917 0.000

NARRATIVES 1.833 0.102 17.914 0.000

NARRATIVES 1.606 0.090 17.863 0.000

NARRATIVES 2.186 0.149 14.664 0.000

NARRATIVES 1.770 0.117 15.144 0.000

Latent Class 2

Means

NARRATIVES 3.830 0.189 20.214 0.000

NARRATIVES 4.264 0.159 26.885 0.000

NARRATIVES 4.827 0.090 53.894 0.000

NARRATIVES 4.373 0.074 59.432 0.000

NARRATIVES 3.745 0.155 24.177 0.000

NARRATIVES 5.307 0.081 65.232 0.000

Variances

NARRATIVES 1.164 0.146 7.991 0.000

NARRATIVES 1.028 0.174 5.917 0.000

NARRATIVES 1.833 0.102 17.914 0.000

NARRATIVES 1.606 0.090 17.863 0.000

NARRATIVES 2.186 0.149 14.664 0.000

NARRATIVES 1.770 0.117 15.144 0.000

Latent Class 3

Means

NARRATIVES 5.917 0.191 30.962 0.000

NARRATIVES 5.982 0.336 17.794 0.000

NARRATIVES 5.904 0.212 27.894 0.000

NARRATIVES 5.155 0.203 25.450 0.000

NARRATIVES 4.995 0.230 21.678 0.000

NARRATIVES 5.264 0.196 26.818 0.000

Variances

NARRATIVES 1.164 0.146 7.991 0.000

NARRATIVES 1.028 0.174 5.917 0.000

NARRATIVES 1.833 0.102 17.914 0.000

NARRATIVES 1.606 0.090 17.863 0.000

NARRATIVES 2.186 0.149 14.664 0.000

NARRATIVES 1.770 0.117 15.144 0.000

Categorical Latent Variables

Means

C#1 0.779 0.346 2.248 0.025

C#2 1.101 0.335 3.288 0.001

QUALITY OF NUMERICAL RESULTS

Condition Number for the Information Matrix 0.857E-02

(ratio of smallest to largest eigenvalue)

TECHNICAL 1 OUTPUT

PARAMETER SPECIFICATION FOR LATENT CLASS 1

NU

NARRATIV NARRATIV NARRATIV NARRATIV NARRATIV

________ ________ ________ ________ ________

1 2 3 4 5

NU

NARRATIV

________

6

THETA

NARRATIV NARRATIV NARRATIV NARRATIV NARRATIV

________ ________ ________ ________ ________

NARRATIV 7

NARRATIV 0 8

NARRATIV 0 0 9

NARRATIV 0 0 0 10

NARRATIV 0 0 0 0 11

NARRATIV 0 0 0 0 0

THETA

NARRATIV

________

NARRATIV 12

PARAMETER SPECIFICATION FOR LATENT CLASS 2

NU

NARRATIV NARRATIV NARRATIV NARRATIV NARRATIV

________ ________ ________ ________ ________

13 14 15 16 17

NU

NARRATIV

________

18

THETA

NARRATIV NARRATIV NARRATIV NARRATIV NARRATIV

________ ________ ________ ________ ________

NARRATIV 7

NARRATIV 0 8

NARRATIV 0 0 9

NARRATIV 0 0 0 10

NARRATIV 0 0 0 0 11

NARRATIV 0 0 0 0 0

THETA

NARRATIV

________

NARRATIV 12

PARAMETER SPECIFICATION FOR LATENT CLASS 3

NU

NARRATIV NARRATIV NARRATIV NARRATIV NARRATIV

________ ________ ________ ________ ________

19 20 21 22 23

NU

NARRATIV

________

24

THETA

NARRATIV NARRATIV NARRATIV NARRATIV NARRATIV

________ ________ ________ ________ ________

NARRATIV 7

NARRATIV 0 8

NARRATIV 0 0 9

NARRATIV 0 0 0 10

NARRATIV 0 0 0 0 11

NARRATIV 0 0 0 0 0

THETA

NARRATIV

________

NARRATIV 12

PARAMETER SPECIFICATION FOR LATENT CLASS REGRESSION MODEL PART

ALPHA(C)

C#1 C#2 C#3

________ ________ ________

25 26 0

STARTING VALUES FOR LATENT CLASS 1

NU

NARRATIV NARRATIV NARRATIV NARRATIV NARRATIV

________ ________ ________ ________ ________

1.774 2.028 3.434 3.016 1.496

NU

NARRATIV

________

4.186

THETA

NARRATIV NARRATIV NARRATIV NARRATIV NARRATIV

________ ________ ________ ________ ________

NARRATIV 1.501

NARRATIV 0.000 1.496

NARRATIV 0.000 0.000 1.033

NARRATIV 0.000 0.000 0.000 0.885

NARRATIV 0.000 0.000 0.000 0.000 1.677

NARRATIV 0.000 0.000 0.000 0.000 0.000

THETA

NARRATIV

________

NARRATIV 0.952

STARTING VALUES FOR LATENT CLASS 2

NU

NARRATIV NARRATIV NARRATIV NARRATIV NARRATIV

________ ________ ________ ________ ________

3.507 3.758 4.872 4.346 3.328

NU

NARRATIV

________

5.566

THETA

NARRATIV NARRATIV NARRATIV NARRATIV NARRATIV

________ ________ ________ ________ ________

NARRATIV 1.501

NARRATIV 0.000 1.496

NARRATIV 0.000 0.000 1.033

NARRATIV 0.000 0.000 0.000 0.885

NARRATIV 0.000 0.000 0.000 0.000 1.677

NARRATIV 0.000 0.000 0.000 0.000 0.000

THETA

NARRATIV

________

NARRATIV 0.952

STARTING VALUES FOR LATENT CLASS 3

NU

NARRATIV NARRATIV NARRATIV NARRATIV NARRATIV

________ ________ ________ ________ ________

5.240 5.488 6.309 5.677 5.159

NU

NARRATIV

________

6.945

THETA

NARRATIV NARRATIV NARRATIV NARRATIV NARRATIV

________ ________ ________ ________ ________

NARRATIV 1.501

NARRATIV 0.000 1.496

NARRATIV 0.000 0.000 1.033

NARRATIV 0.000 0.000 0.000 0.885

NARRATIV 0.000 0.000 0.000 0.000 1.677

NARRATIV 0.000 0.000 0.000 0.000 0.000

THETA

NARRATIV

________

NARRATIV 0.952

STARTING VALUES FOR LATENT CLASS REGRESSION MODEL PART

ALPHA(C)

C#1 C#2 C#3

________ ________ ________

0.000 0.000 0.000

TECHNICAL 4 OUTPUT

TECHNICAL 11 OUTPUT

Random Starts Specifications for the k-1 Class Analysis Model

Number of initial stage random starts 1000

Number of final stage optimizations 250

VUONG-LO-MENDELL-RUBIN LIKELIHOOD RATIO TEST FOR 2 (H0) VERSUS 3 CLASSES

H0 Loglikelihood Value -7523.620

2 Times the Loglikelihood Difference 189.430

Difference in the Number of Parameters 7

Mean 23.118

Standard Deviation 29.837

P-Value 0.0013

LO-MENDELL-RUBIN ADJUSTED LRT TEST

Value 185.389

P-Value 0.0014

TECHNICAL 14 OUTPUT

Random Starts Specifications for the k-1 Class Analysis Model

Number of initial stage random starts 1000

Number of final stage optimizations 250

Random Starts Specification for the k-1 Class Model for Generated Data

Number of initial stage random starts 2

Number of final stage optimizations 1

Random Starts Specification for the k Class Model for Generated Data

Number of initial stage random starts 50

Number of final stage optimizations 10

Number of bootstrap draws requested Varies

PARAMETRIC BOOTSTRAPPED LIKELIHOOD RATIO TEST FOR 2 (H0) VERSUS 3 CLASSES

H0 Loglikelihood Value -7523.620

2 Times the Loglikelihood Difference 189.430

Difference in the Number of Parameters 7

Approximate P-Value 0.0000

Successful Bootstrap Draws 5

TECHNICAL 8 OUTPUT

INITIAL STAGE ITERATIONS

TECHNICAL 8 OUTPUT FOR UNPERTURBED STARTING VALUE SET

ITER LOGLIKELIHOOD ABS CHANGE REL CHANGE CLASS COUNTS ALGORITHM

1 -0.81831785D+04 0.0000000 0.0000000 135.589 436.745 EM

129.665

2 -0.75496613D+04 633.5172367 0.0774170 140.246 427.806 EM

133.948

3 -0.75057149D+04 43.9464199 0.0058210 148.532 411.670 EM

141.797

4 -0.74829979D+04 22.7169514 0.0030266 159.902 393.137 EM

148.961

5 -0.74630231D+04 19.9748380 0.0026694 173.523 373.963 EM

154.515

6 -0.74472659D+04 15.7571649 0.0021114 186.312 357.789 EM

157.898

7 -0.74388452D+04 8.4206756 0.0011307 196.515 346.114 EM

159.371

8 -0.74351447D+04 3.7005688 0.0004975 204.228 338.230 EM

159.542

9 -0.74334291D+04 1.7156245 0.0002307 210.042 333.063 EM

158.894

10 -0.74325197D+04 0.9093342 0.0001223 214.486 329.771 EM

157.743

TECHNICAL 8 OUTPUT FOR STARTING VALUE SET 1

ITER LOGLIKELIHOOD ABS CHANGE REL CHANGE CLASS COUNTS ALGORITHM

1 -0.20699169D+05 0.0000000 0.0000000 445.579 228.247 EM

28.174

2 -0.75869903D+04 ************ 0.6334640 423.197 246.583 EM

32.220

3 -0.75363770D+04 50.6133372 0.0066711 398.636 265.127 EM

38.237

4 -0.75187157D+04 17.6612495 0.0023435 377.342 281.327 EM

43.331

5 -0.75095228D+04 9.1929883 0.0012227 359.335 294.992 EM

47.673

6 -0.75038545D+04 5.6682037 0.0007548 344.103 306.384 EM

51.514

7 -0.75001462D+04 3.7083971 0.0004942 331.175 315.882 EM

54.943

8 -0.74975780D+04 2.5681104 0.0003424 320.124 323.851 EM

58.025

9 -0.74956896D+04 1.8883973 0.0002519 310.580 330.590 EM

60.830

10 -0.74942291D+04 1.4605879 0.0001949 302.247 336.321 EM

63.432

11 -0.74930587D+04 1.1703171 0.0001562 294.892 341.211 EM

65.897

12 -0.74921001D+04 0.9586863 0.0001279 288.340 345.383 EM

68.277

TECHNICAL 8 OUTPUT FOR STARTING VALUE SET 2

ITER LOGLIKELIHOOD ABS CHANGE REL CHANGE CLASS COUNTS ALGORITHM

1 -0.19155871D+05 0.0000000 0.0000000 2.001 429.008 EM

270.991

2 -0.76284626D+04 ************ 0.6017689 3.237 409.959 EM

288.804

3 -0.75372452D+04 91.2173904 0.0119575 3.813 391.975 EM

306.212

4 -0.75194595D+04 17.7857370 0.0023597 4.173 378.221 EM

319.606

5 -0.75146313D+04 4.8281697 0.0006421 4.395 367.570 EM

330.035

6 -0.75122348D+04 2.3965735 0.0003189 4.558 359.341 EM

338.100

7 -0.75108605D+04 1.3742923 0.0001829 4.701 353.035 EM

344.264

8 -0.75100466D+04 0.8139058 0.0001084 4.839 348.227 EM

348.934

TECHNICAL 8 OUTPUT FOR STARTING VALUE SET 3

ITER LOGLIKELIHOOD ABS CHANGE REL CHANGE CLASS COUNTS ALGORITHM

1 -0.18315063D+05 0.0000000 0.0000000 4.770 658.734 EM

38.496

2 -0.77942131D+04 ************ 0.5744370 10.297 639.674 EM

52.029

3 -0.76866667D+04 107.5463936 0.0137982 22.853 598.433 EM

80.714

4 -0.76054427D+04 81.2239424 0.0105669 36.971 557.800 EM

107.229

5 -0.75657078D+04 39.7349404 0.0052245 49.284 523.192 EM

129.523

6 -0.75430205D+04 22.6872754 0.0029987 59.910 493.659 EM

148.431

7 -0.75271152D+04 15.9052851 0.0021086 69.519 467.671 EM

164.810

8 -0.75146052D+04 12.5100116 0.0016620 78.638 444.199 EM

179.164

9 -0.75042105D+04 10.3947323 0.0013833 87.596 422.835 EM

191.568

10 -0.74955257D+04 8.6847792 0.0011573 96.528 403.643 EM

201.829

11 -0.74883600D+04 7.1657105 0.0009560 105.476 386.780 EM

209.744

12 -0.74823525D+04 6.0074975 0.0008022 114.538 372.195 EM

215.267

13 -0.74768987D+04 5.4537662 0.0007289 123.941 359.566 EM

218.492

14 -0.74712657D+04 5.6329863 0.0007534 133.973 348.454 EM

219.572

15 -0.74648793D+04 6.3864397 0.0008548 144.689 338.631 EM

218.680

16 -0.74579324D+04 6.9468665 0.0009306 155.558 330.369 EM

216.073

17 -0.74515929D+04 6.3395871 0.0008500 165.710 324.083 EM

212.207

18 -0.74467677D+04 4.8251547 0.0006475 174.606 319.727 EM

207.668

19 -0.74433619D+04 3.4058015 0.0004574 182.162 316.906 EM

202.932

20 -0.74409280D+04 2.4338978 0.0003270 188.521 315.209 EM

198.270

21 -0.74391152D+04 1.8127913 0.0002436 193.882 314.315 EM

193.803

22 -0.74377098D+04 1.4053910 0.0001889 198.434 313.995 EM

189.571

23 -0.74365849D+04 1.1249518 0.0001512 202.336 314.084 EM

185.580

24 -0.74356620D+04 0.9228319 0.0001241 205.715 314.461 EM

181.824

TECHNICAL 8 OUTPUT FOR STARTING VALUE SET 4

ITER LOGLIKELIHOOD ABS CHANGE REL CHANGE CLASS COUNTS ALGORITHM

1 -0.22467696D+05 0.0000000 0.0000000 418.083 265.832 EM

18.085

2 -0.76485131D+04 ************ 0.6595773 403.006 279.021 EM

19.973

3 -0.75506590D+04 97.8541242 0.0127939 381.846 294.660 EM

25.494

4 -0.75223913D+04 28.2677110 0.0037437 361.017 309.108 EM

31.874

5 -0.75088551D+04 13.5362441 0.0017995 343.382 321.324 EM

37.293

6 -0.75015811D+04 7.2739206 0.0009687 329.196 331.000 EM

41.804

7 -0.74976574D+04 3.9237836 0.0005231 317.794 338.424 EM

45.783

8 -0.74954064D+04 2.2509181 0.0003002 308.477 344.087 EM

49.436

9 -0.74939591D+04 1.4473368 0.0001931 300.688 348.440 EM

52.872

10 -0.74929179D+04 1.0412103 0.0001389 294.020 351.832 EM

56.148

11 -0.74921053D+04 0.8126019 0.0001084 288.186 354.514 EM

59.300

TECHNICAL 8 OUTPUT FOR STARTING VALUE SET 5

ITER LOGLIKELIHOOD ABS CHANGE REL CHANGE CLASS COUNTS ALGORITHM

1 -0.18153585D+05 0.0000000 0.0000000 427.291 227.146 EM

47.563

2 -0.76468438D+04 ************ 0.5787695 417.307 238.117 EM

46.576

3 -0.75473941D+04 99.4496883 0.0130053 402.559 251.217 EM

48.223

4 -0.75309737D+04 16.4204177 0.0021756 389.114 263.873 EM

49.012

5 -0.75212214D+04 9.7523418 0.0012950 376.688 276.381 EM

48.931

6 -0.75123464D+04 8.8750085 0.0011800 364.896 289.396 EM

47.708

7 -0.75037170D+04 8.6293997 0.0011487 353.581 301.916 EM

46.503

8 -0.74922357D+04 11.4812898 0.0015301 342.610 311.281 EM

48.109

9 -0.74745552D+04 17.6804488 0.0023598 331.634 316.765 EM

53.602

10 -0.74576303D+04 16.9249083 0.0022643 320.927 320.964 EM

60.109

11 -0.74479323D+04 9.6979893 0.0013004 311.463 324.810 EM

65.727

12 -0.74430782D+04 4.8541521 0.0006517 303.514 328.019 EM

70.467

13 -0.74401303D+04 2.9478532 0.0003961 296.777 330.602 EM

74.621

14 -0.74379579D+04 2.1723931 0.0002920 290.904 332.762 EM

78.334

15 -0.74362127D+04 1.7452078 0.0002346 285.667 334.659 EM

81.674

16 -0.74347647D+04 1.4480606 0.0001947 280.939 336.374 EM

84.687

17 -0.74335503D+04 1.2143475 0.0001633 276.658 337.936 EM

87.406

18 -0.74325347D+04 1.0156086 0.0001366 272.800 339.341 EM

89.859

19 -0.74316973D+04 0.8374400 0.0001127 269.358 340.572 EM

92.070

TECHNICAL 8 OUTPUT FOR STARTING VALUE SET 6

ITER LOGLIKELIHOOD ABS CHANGE REL CHANGE CLASS COUNTS ALGORITHM

1 -0.17532212D+05 0.0000000 0.0000000 8.234 633.447 EM

60.319

2 -0.77150327D+04 9817.1797958 0.5599510 5.530 608.933 EM

87.537

3 -0.76643871D+04 50.6455944 0.0065645 5.938 578.924 EM

117.138

4 -0.76265657D+04 37.8213902 0.0049347 6.329 550.628 EM

145.043

5 -0.76016649D+04 24.9007755 0.0032650 5.992 524.727 EM

171.281

6 -0.75827130D+04 18.9519148 0.0024931 5.556 500.270 EM

196.174

7 -0.75667000D+04 16.0130169 0.0021118 5.223 477.219 EM

219.558

8 -0.75533517D+04 13.3483549 0.0017641 5.015 455.864 EM

241.121

9 -0.75425667D+04 10.7849442 0.0014278 4.908 436.346 EM

260.746

10 -0.75339338D+04 8.6328666 0.0011446 4.870 418.663 EM

278.467

11 -0.75270025D+04 6.9313287 0.0009200 4.876 402.845 EM

294.279

12 -0.75215030D+04 5.4995237 0.0007306 4.912 388.993 EM

308.096

13 -0.75173044D+04 4.1985687 0.0005582 4.966 377.202 EM

319.832

14 -0.75142738D+04 3.0306278 0.0004032 5.032 367.456 EM

329.512

15 -0.75122086D+04 2.0652340 0.0002748 5.105 359.601 EM

337.294

16 -0.75108672D+04 1.3413223 0.0001786 5.182 353.392 EM

343.426

17 -0.75100263D+04 0.8409396 0.0001120 5.258 348.547 EM

348.195

TECHNICAL 8 OUTPUT FOR STARTING VALUE SET 7

ITER LOGLIKELIHOOD ABS CHANGE REL CHANGE CLASS COUNTS ALGORITHM

1 -0.22521388D+05 0.0000000 0.0000000 124.741 465.582 EM

111.677

2 -0.75988424D+04 ************ 0.6625944 135.056 437.699 EM

129.245

3 -0.75205601D+04 78.2823034 0.0103019 148.093 412.133 EM

141.774

4 -0.74853813D+04 35.1788070 0.0046777 161.420 390.649 EM

149.931

5 -0.74630210D+04 22.3602988 0.0029872 175.233 371.558 EM

155.209

6 -0.74472494D+04 15.7715842 0.0021133 187.697 356.149 EM

158.154

7 -0.74388445D+04 8.4049137 0.0011286 197.582 345.106 EM

159.312

8 -0.74350854D+04 3.7591358 0.0005053 205.070 337.634 EM

159.295

9 -0.74333446D+04 1.7407154 0.0002341 210.729 332.728 EM

158.543

10 -0.74324369D+04 0.9077433 0.0001221 215.060 329.603 EM

157.336

TECHNICAL 8 OUTPUT FOR STARTING VALUE SET 8

ITER LOGLIKELIHOOD ABS CHANGE REL CHANGE CLASS COUNTS ALGORITHM

1 -0.16972634D+05 0.0000000 0.0000000 490.919 61.151 EM

149.930

2 -0.76173762D+04 9355.2578039 0.5511966 472.607 66.343 EM

163.050

3 -0.75638908D+04 53.4853215 0.0070215 451.925 66.831 EM

183.243

4 -0.75448907D+04 19.0001380 0.0025120 433.125 66.250 EM

202.625

5 -0.75326681D+04 12.2226141 0.0016200 416.146 66.075 EM

219.779

6 -0.75224791D+04 10.1890132 0.0013526 401.054 66.862 EM

234.085

7 -0.75142990D+04 8.1800846 0.0010874 388.153 68.497 EM

245.349

8 -0.75088051D+04 5.4938761 0.0007311 377.606 70.537 EM

253.857

9 -0.75055704D+04 3.2347221 0.0004308 369.211 72.732 EM

260.057

10 -0.75036550D+04 1.9153642 0.0002552 362.581 75.029 EM

264.390

11 -0.75024311D+04 1.2239314 0.0001631 357.334 77.431 EM

267.235

12 -0.75015794D+04 0.8516813 0.0001135 353.149 79.961 EM

268.890

TECHNICAL 8 OUTPUT FOR STARTING VALUE SET 9

ITER LOGLIKELIHOOD ABS CHANGE REL CHANGE CLASS COUNTS ALGORITHM

1 -0.12860146D+05 0.0000000 0.0000000 0.987 687.577 EM

13.435

2 -0.77829119D+04 5077.2344510 0.3948038 8.992 668.763 EM

24.245

3 -0.77308912D+04 52.0206363 0.0066840 21.457 643.260 EM

37.284

4 -0.76788165D+04 52.0747210 0.0067359 35.917 608.568 EM

57.515

5 -0.76188225D+04 59.9940006 0.0078129 50.648 568.792 EM

82.560

6 -0.75744203D+04 44.4021748 0.0058280 64.469 531.919 EM

105.612

7 -0.75475279D+04 26.8924360 0.0035504 77.144 499.638 EM

125.218

8 -0.75286700D+04 18.8578828 0.0024986 89.116 471.056 EM

141.828

9 -0.75134805D+04 15.1895490 0.0020176 100.850 445.227 EM

155.923

10 -0.75002737D+04 13.2067721 0.0017577 112.640 421.617 EM

167.743

11 -0.74882831D+04 11.9905548 0.0015987 124.775 399.976 EM

177.250

12 -0.74767960D+04 11.4871731 0.0015340 137.617 380.132 EM

184.251

13 -0.74651836D+04 11.6123944 0.0015531 151.126 362.306 EM

188.567

14 -0.74542713D+04 10.9122563 0.0014618 164.184 347.566 EM

190.250

15 -0.74462545D+04 8.0167887 0.0010755 175.481 336.716 EM

189.803

16 -0.74414898D+04 4.7647716 0.0006399 184.650 329.347 EM

188.002

17 -0.74387390D+04 2.7508048 0.0003697 191.965 324.555 EM

185.480

18 -0.74370132D+04 1.7257061 0.0002320 197.830 321.552 EM

182.618

19 -0.74358182D+04 1.1950486 0.0001607 202.597 319.777 EM

179.626

20 -0.74349230D+04 0.8952305 0.0001204 206.533 318.846 EM

176.620

TECHNICAL 8 OUTPUT FOR STARTING VALUE SET 10

ITER LOGLIKELIHOOD ABS CHANGE REL CHANGE CLASS COUNTS ALGORITHM

1 -0.17234873D+05 0.0000000 0.0000000 200.453 381.816 EM

119.731

2 -0.76299396D+04 9604.9332879 0.5572964 213.931 360.447 EM

127.621

3 -0.75889678D+04 40.9718820 0.0053699 226.144 342.979 EM

132.878

4 -0.75714495D+04 17.5182532 0.0023084 239.506 327.251 EM

135.243

5 -0.75540017D+04 17.4477700 0.0023044 254.521 312.961 EM

134.518

6 -0.75373009D+04 16.7008757 0.0022109 269.183 302.038 EM

130.779

7 -0.75256906D+04 11.6102778 0.0015404 282.063 294.888 EM

125.049

8 -0.75185982D+04 7.0923862 0.0009424 292.894 290.426 EM

118.680

9 -0.75136432D+04 4.9550355 0.0006590 301.865 287.396 EM

112.739

10 -0.75095371D+04 4.1060309 0.0005465 309.253 284.774 EM

107.973

11 -0.75060557D+04 3.4814444 0.0004636 315.265 281.959 EM

104.776

12 -0.75033552D+04 2.7005198 0.0003598 320.040 278.802 EM

103.158

13 -0.75014245D+04 1.9306639 0.0002573 323.709 275.390 EM

102.901

14 -0.75000580D+04 1.3664756 0.0001822 326.420 271.840 EM

103.740

15 -0.74990347D+04 1.0233557 0.0001364 328.337 268.223 EM

105.440

16 -0.74981990D+04 0.8356433 0.0001114 329.613 264.573 EM

107.814

TECHNICAL 8 OUTPUT FOR STARTING VALUE SET 11

ITER LOGLIKELIHOOD ABS CHANGE REL CHANGE CLASS COUNTS ALGORITHM

1 -0.24385766D+05 0.0000000 0.0000000 356.686 78.523 EM

266.791

2 -0.75404962D+04 ************ 0.6907829 357.291 77.036 EM

267.673

3 -0.74947487D+04 45.7474779 0.0060669 351.546 80.020 EM

270.434

4 -0.74779031D+04 16.8456105 0.0022477 343.307 82.683 EM

276.010

5 -0.74655053D+04 12.3978392 0.0016579 333.630 84.451 EM

283.919

6 -0.74557650D+04 9.7402799 0.0013047 323.523 85.696 EM

292.781

7 -0.74482772D+04 7.4878352 0.0010043 313.813 86.858 EM

301.330

8 -0.74428945D+04 5.3827118 0.0007227 304.943 88.070 EM

308.987

9 -0.74392976D+04 3.5968410 0.0004833 297.097 89.365 EM

315.538

10 -0.74368935D+04 2.4040733 0.0003232 290.272 90.760 EM

320.968

11 -0.74351783D+04 1.7152863 0.0002306 284.354 92.235 EM

325.411

12 -0.74338605D+04 1.3177888 0.0001772 279.199 93.747 EM

329.055

13 -0.74327957D+04 1.0647443 0.0001432 274.683 95.250 EM

332.067

14 -0.74319167D+04 0.8790426 0.0001183 270.723 96.708 EM

334.569

TECHNICAL 8 OUTPUT FOR STARTING VALUE SET 12

ITER LOGLIKELIHOOD ABS CHANGE REL CHANGE CLASS COUNTS ALGORITHM

1 -0.15463647D+05 0.0000000 0.0000000 45.019 618.095 EM

38.886

2 -0.77340524D+04 7729.5940784 0.4998558 54.822 601.547 EM

45.631

3 -0.76805557D+04 53.4967109 0.0069170 72.403 577.888 EM

51.709

4 -0.76539404D+04 26.6152778 0.0034653 89.842 554.381 EM

57.777

5 -0.76325050D+04 21.4354633 0.0028006 107.796 527.932 EM

66.273

6 -0.75996486D+04 32.8564081 0.0043048 125.793 496.114 EM

80.093

7 -0.75493380D+04 50.3105344 0.0066201 142.622 462.007 EM

97.372

8 -0.75060514D+04 43.2866898 0.0057338 158.697 431.221 EM

112.082

9 -0.74764924D+04 29.5589086 0.0039380 175.437 403.874 EM

122.690

10 -0.74533124D+04 23.1800025 0.0031004 191.047 381.038 EM

129.916

11 -0.74399814D+04 13.3309931 0.0017886 203.333 364.272 EM

134.395

12 -0.74343164D+04 5.6650330 0.0007614 212.309 352.793 EM

136.898

13 -0.74319667D+04 2.3497342 0.0003161 218.773 345.145 EM

138.083

14 -0.74309095D+04 1.0572294 0.0001423 223.456 340.137 EM

138.407

15 -0.74303878D+04 0.5216722 0.0000702 226.898 336.921 EM

138.181

TECHNICAL 8 OUTPUT FOR STARTING VALUE SET 13

ITER LOGLIKELIHOOD ABS CHANGE REL CHANGE CLASS COUNTS ALGORITHM

1 -0.19869606D+05 0.0000000 0.0000000 141.920 483.455 EM

76.625

2 -0.75432998D+04 ************ 0.6203599 162.886 457.653 EM

81.461

3 -0.75128409D+04 30.4588231 0.0040379 176.397 435.598 EM

90.006

4 -0.75002335D+04 12.6074285 0.0016781 185.837 418.396 EM

97.766

5 -0.74935404D+04 6.6930784 0.0008924 192.335 405.391 EM

104.274

6 -0.74899098D+04 3.6305817 0.0004845 196.661 395.630 EM

109.709

7 -0.74878643D+04 2.0454959 0.0002731 199.404 388.267 EM

114.328

8 -0.74866493D+04 1.2149998 0.0001623 200.998 382.658 EM

118.344

9 -0.74858868D+04 0.7625220 0.0001019 201.753 378.336 EM

121.911

TECHNICAL 8 OUTPUT FOR STARTING VALUE SET 14

ITER LOGLIKELIHOOD ABS CHANGE REL CHANGE CLASS COUNTS ALGORITHM

1 -0.19713021D+05 0.0000000 0.0000000 17.250 617.670 EM

67.079

2 -0.77241199D+04 ************ 0.6081717 15.092 588.796 EM

98.111

3 -0.76470061D+04 77.1138488 0.0099835 16.092 556.512 EM

129.395

4 -0.76133716D+04 33.6344513 0.0043984 16.743 527.336 EM

157.920

5 -0.75901561D+04 23.2154957 0.0030493 17.672 499.741 EM

184.587

6 -0.75705852D+04 19.5709567 0.0025785 19.238 473.436 EM

209.326

7 -0.75538310D+04 16.7541866 0.0022131 21.495 448.806 EM

231.699

8 -0.75399601D+04 13.8708696 0.0018363 24.420 426.267 EM

251.313

9 -0.75288288D+04 11.1313558 0.0014763 28.006 406.085 EM

267.909

10 -0.75200940D+04 8.7347414 0.0011602 32.248 388.421 EM

281.331

11 -0.75133820D+04 6.7119989 0.0008925 37.110 373.312 EM

291.578

12 -0.75082987D+04 5.0833055 0.0006766 42.524 360.628 EM

298.849

13 -0.75044440D+04 3.8547140 0.0005134 48.393 350.112 EM

303.495

14 -0.75014663D+04 2.9776984 0.0003968 54.621 341.454 EM

305.924

15 -0.74990888D+04 2.3775138 0.0003169 61.137 334.341 EM

306.522

16 -0.74970978D+04 1.9909887 0.0002655 67.911 328.481 EM

305.609

17 -0.74953240D+04 1.7737923 0.0002366 74.957 323.608 EM

303.435

18 -0.74936293D+04 1.6947435 0.0002261 82.332 319.486 EM

300.183

19 -0.74918961D+04 1.7331343 0.0002313 90.126 315.906 EM

295.968

20 -0.74900185D+04 1.8776813 0.0002506 98.460 312.683 EM

290.856

21 -0.74878948D+04 2.1236738 0.0002835 107.475 309.649 EM

284.876

22 -0.74854300D+04 2.4647933 0.0003292 117.321 306.643 EM

278.037

23 -0.74825498D+04 2.8801568 0.0003848 128.126 303.509 EM

270.365

24 -0.74792250D+04 3.3248897 0.0004444 139.953 300.108 EM

261.939

25 -0.74754874D+04 3.7375883 0.0004997 152.757 296.332 EM

252.911

26 -0.74714223D+04 4.0650168 0.0005438 166.380 292.125 EM

243.495

27 -0.74671395D+04 4.2828353 0.0005732 180.598 287.481 EM

233.921

28 -0.74627408D+04 4.3987227 0.0005891 195.179 282.424 EM

224.397

29 -0.74583024D+04 4.4384008 0.0005947 209.922 277.001 EM

215.077

30 -0.74538810D+04 4.4214386 0.0005928 224.641 271.286 EM

206.074

31 -0.74495441D+04 4.3368350 0.0005818 239.124 265.396 EM

197.481

32 -0.74454090D+04 4.1350829 0.0005551 253.082 259.513 EM

189.405

33 -0.74416514D+04 3.7576277 0.0005047 266.151 253.875 EM

181.973

34 -0.74384570D+04 3.1944275 0.0004293 277.961 248.738 EM

175.301

35 -0.74359404D+04 2.5165447 0.0003383 288.235 244.308 EM

169.457

36 -0.74323993D+04 3.5411704 0.0004762 313.722 234.894 FS

153.384

37 -0.74313158D+04 1.0834546 0.0001458 319.010 234.498 FS

148.492

38 -0.74306490D+04 0.6668555 0.0000897 321.176 234.354 EM

146.470

TECHNICAL 8 OUTPUT FOR STARTING VALUE SET 15

ITER LOGLIKELIHOOD ABS CHANGE REL CHANGE CLASS COUNTS ALGORITHM

1 -0.18774224D+05 0.0000000 0.0000000 391.808 32.438 EM

277.754

2 -0.76015969D+04 ************ 0.5951046 395.274 22.771 EM

283.954

3 -0.75458368D+04 55.7601030 0.0073353 385.874 21.736 EM

294.390

4 -0.75327903D+04 13.0465036 0.0017290 372.636 23.640 EM

305.723

5 -0.75246942D+04 8.0960645 0.0010748 357.890 27.725 EM

316.384

6 -0.75163639D+04 8.3303020 0.0011071 342.520 33.612 EM

325.868

7 -0.75075471D+04 8.8168826 0.0011730 328.187 39.786 EM

334.027

8 -0.75007730D+04 6.7740533 0.0009023 316.312 44.931 EM

340.758

9 -0.74967729D+04 4.0001455 0.0005333 306.740 49.148 EM

346.112

10 -0.74944687D+04 2.3041908 0.0003074 298.836 52.868 EM

350.296

11 -0.74930305D+04 1.4381668 0.0001919 292.124 56.326 EM

353.550

12 -0.74920424D+04 0.9880939 0.0001319 286.291 59.621 EM

356.088

TECHNICAL 8 OUTPUT FOR STARTING VALUE SET 16

ITER LOGLIKELIHOOD ABS CHANGE REL CHANGE CLASS COUNTS ALGORITHM

1 -0.19934786D+05 0.0000000 0.0000000 556.582 59.128 EM

86.290

2 -0.76531396D+04 ************ 0.6160912 523.934 64.385 EM

113.681

3 -0.75630389D+04 90.1006852 0.0117730 491.072 74.080 EM

136.848

4 -0.75323747D+04 30.6641971 0.0040545 465.825 81.856 EM

154.319

5 -0.75172490D+04 15.1257370 0.0020081 443.902 89.395 EM

168.703

6 -0.75056188D+04 11.6302291 0.0015471 423.479 97.503 EM

181.018

7 -0.74956914D+04 9.9273280 0.0013227 404.463 106.236 EM

191.301

8 -0.74871215D+04 8.5699113 0.0011433 387.173 115.510 EM

199.317

9 -0.74794980D+04 7.6234861 0.0010182 371.707 125.386 EM

204.907

10 -0.74720819D+04 7.4161896 0.0009915 357.873 136.067 EM

208.059

11 -0.74641850D+04 7.8968955 0.0010569 345.609 147.511 EM

208.880

12 -0.74561303D+04 8.0546352 0.0010791 335.411 158.960 EM

207.629

13 -0.74493816D+04 6.7487677 0.0009051 327.789 169.378 EM

204.833

14 -0.74446897D+04 4.6919002 0.0006298 322.566 178.269 EM

201.165

15 -0.74415916D+04 3.0980492 0.0004161 319.183 185.666 EM

197.151

16 -0.74394590D+04 2.1326031 0.0002866 317.114 191.797 EM

193.089

17 -0.74378976D+04 1.5614316 0.0002099 315.971 196.910 EM

189.119

18 -0.74366938D+04 1.2037310 0.0001618 315.478 201.219 EM

185.303

19 -0.74357298D+04 0.9640717 0.0001296 315.443 204.892 EM

181.665

TECHNICAL 8 OUTPUT FOR STARTING VALUE SET 17

ITER LOGLIKELIHOOD ABS CHANGE REL CHANGE CLASS COUNTS ALGORITHM

1 -0.27777024D+05 0.0000000 0.0000000 267.167 298.904 EM

135.930

2 -0.75640474D+04 ************ 0.7276869 249.236 298.648 EM

154.116

3 -0.74788159D+04 85.2315215 0.0112680 253.603 290.950 EM

157.447

4 -0.74529652D+04 25.8506583 0.0034565 267.204 281.887 EM

152.909

5 -0.74427714D+04 10.1937651 0.0013677 281.686 273.599 EM

146.715

6 -0.74370036D+04 5.7678491 0.0007750 294.381 266.469 EM

141.150

7 -0.74335637D+04 3.4398295 0.0004625 304.711 260.583 EM

136.706

8 -0.74316036D+04 1.9601330 0.0002637 312.778 255.914 EM

133.308

9 -0.74305217D+04 1.0819097 0.0001456 318.924 252.333 EM

130.743

10 -0.74299261D+04 0.5956140 0.0000802 323.544 249.662 EM

128.794

TECHNICAL 8 OUTPUT FOR STARTING VALUE SET 18

ITER LOGLIKELIHOOD ABS CHANGE REL CHANGE CLASS COUNTS ALGORITHM

1 -0.17720553D+05 0.0000000 0.0000000 615.439 86.555 EM

0.006

2 -0.76918826D+04 ************ 0.5659344 591.576 110.386 EM

0.038

3 -0.76492862D+04 42.5963872 0.0055378 564.978 136.712 EM

0.310

4 -0.76210762D+04 28.2099875 0.0036879 538.109 162.829 EM

1.062

5 -0.75984931D+04 22.5830547 0.0029632 512.000 188.109 EM

1.891

6 -0.75796135D+04 18.8796358 0.0024847 487.519 211.814 EM

2.667

7 -0.75645391D+04 15.0744022 0.0019888 465.368 233.359 EM

3.274

8 -0.75529050D+04 11.6341073 0.0015380 445.565 252.595 EM

3.840

9 -0.75439440D+04 8.9610245 0.0011864 427.942 269.756 EM

4.302

10 -0.75370127D+04 6.9312222 0.0009188 412.309 285.039 EM

4.653

11 -0.75315548D+04 5.4579589 0.0007242 398.554 298.481 EM

4.965

12 -0.75272663D+04 4.2884731 0.0005694 386.664 310.061 EM

5.275

13 -0.75239965D+04 3.2698216 0.0004344 376.621 319.799 EM

5.579

14 -0.75216203D+04 2.3762183 0.0003158 368.333 327.809 EM

5.858

15 -0.75199817D+04 1.6385406 0.0002178 361.626 334.275 EM

6.099

16 -0.75189004D+04 1.0813354 0.0001438 356.278 339.417 EM

6.305

17 -0.75182085D+04 0.6918944 0.0000920 352.056 343.460 EM

6.484

TECHNICAL 8 OUTPUT FOR STARTING VALUE SET 19

ITER LOGLIKELIHOOD ABS CHANGE REL CHANGE CLASS COUNTS ALGORITHM

1 -0.23058612D+05 0.0000000 0.0000000 326.493 350.439 EM

25.069

2 -0.75417629D+04 ************ 0.6729308 331.016 350.203 EM

20.781

3 -0.75231035D+04 18.6594715 0.0024742 332.479 347.293 EM

22.228

4 -0.75156439D+04 7.4595396 0.0009916 333.171 343.806 EM

25.023

5 -0.75110286D+04 4.6153089 0.0006141 333.765 340.600 EM

27.634

6 -0.75085742D+04 2.4544324 0.0003268 334.352 337.738 EM

29.910

7 -0.75071100D+04 1.4641634 0.0001950 334.857 335.037 EM

32.107

8 -0.75060853D+04 1.0247361 0.0001365 335.232 332.443 EM

34.325

9 -0.75053216D+04 0.7637056 0.0001017 335.471 329.984 EM

36.545

TECHNICAL 8 OUTPUT FOR STARTING VALUE SET 20

ITER LOGLIKELIHOOD ABS CHANGE REL CHANGE CLASS COUNTS ALGORITHM

1 -0.12050867D+05 0.0000000 0.0000000 0.135 21.752 EM

680.112

2 -0.77894096D+04 4261.4572148 0.3536225 0.767 41.838 EM

659.395

3 -0.77191763D+04 70.2333361 0.0090165 2.352 69.205 EM

630.443

4 -0.76749804D+04 44.1959144 0.0057255 2.885 97.633 EM

601.482

5 -0.76422255D+04 32.7548739 0.0042677 3.228 125.562 EM

573.211

6 -0.76166913D+04 25.5342057 0.0033412 3.445 152.473 EM

546.082

7 -0.75957479D+04 20.9434259 0.0027497 3.583 178.212 EM

520.204

8 -0.75779885D+04 17.7593152 0.0023381 3.679 202.636 EM

495.685

9 -0.75629000D+04 15.0885554 0.0019911 3.759 225.421 EM

472.819

10 -0.75504443D+04 12.4557399 0.0016470 3.839 246.310 EM

451.851

11 -0.75404115D+04 10.0327705 0.0013288 3.923 265.279 EM

432.798

12 -0.75323448D+04 8.0666676 0.0010698 4.010 282.395 EM

415.594

13 -0.75258445D+04 6.5002738 0.0008630 4.099 297.641 EM

400.260

14 -0.75207036D+04 5.1409903 0.0006831 4.188 310.911 EM

386.901

15 -0.75168140D+04 3.8895839 0.0005172 4.276 322.127 EM

375.597

16 -0.75140342D+04 2.7797588 0.0003698 4.364 331.333 EM

366.303

17 -0.75121516D+04 1.8825754 0.0002505 4.453 338.705 EM

358.841

18 -0.75109275D+04 1.2240891 0.0001629 4.549 344.498 EM

352.953

19 -0.75101500D+04 0.7775642 0.0001035 4.656 348.986 EM

348.358

TECHNICAL 8 OUTPUT FOR STARTING VALUE SET 21

ITER LOGLIKELIHOOD ABS CHANGE REL CHANGE CLASS COUNTS ALGORITHM

1 -0.26059757D+05 0.0000000 0.0000000 334.656 290.654 EM

76.690

2 -0.75882732D+04 ************ 0.7088126 333.460 275.150 EM

93.390

3 -0.74969467D+04 91.3265606 0.0120352 326.059 273.783 EM

102.158

4 -0.74654893D+04 31.4573107 0.0041960 317.833 279.411 EM

104.756

5 -0.74533798D+04 12.1095439 0.0016221 309.759 287.307 EM

104.934

6 -0.74459323D+04 7.4475226 0.0009992 301.844 295.655 EM

104.502

7 -0.74406248D+04 5.3074560 0.0007128 294.263 303.756 EM

103.981

8 -0.74370837D+04 3.5411270 0.0004759 287.312 311.075 EM

103.613

9 -0.74347922D+04 2.2914864 0.0003081 281.160 317.330 EM

103.511

10 -0.74332603D+04 1.5319476 0.0002061 275.821 322.505 EM

103.674

11 -0.74321679D+04 1.0924091 0.0001470 271.227 326.728 EM

104.045

12 -0.74313425D+04 0.8253707 0.0001111 267.292 330.156 EM

104.552

TECHNICAL 8 OUTPUT FOR STARTING VALUE SET 22

ITER LOGLIKELIHOOD ABS CHANGE REL CHANGE CLASS COUNTS ALGORITHM

1 -0.20879096D+05 0.0000000 0.0000000 291.212 367.185 EM

43.603

2 -0.77209710D+04 ************ 0.6302057 278.795 347.379 EM

75.826

3 -0.76322146D+04 88.7563173 0.0114955 265.628 329.718 EM

106.654

4 -0.75699231D+04 62.2915472 0.0081617 253.085 317.086 EM

131.829

5 -0.75096026D+04 60.3204945 0.0079684 247.162 307.968 EM

146.870

6 -0.74695277D+04 40.0749496 0.0053365 251.267 299.768 EM

150.965

7 -0.74531537D+04 16.3739554 0.0021921 261.107 292.103 EM

148.790

8 -0.74456612D+04 7.4925292 0.0010053 272.518 284.881 EM

144.601

9 -0.74406116D+04 5.0495187 0.0006782 283.725 278.057 EM

140.219

10 -0.74368997D+04 3.7119615 0.0004989 293.947 271.765 EM

136.288

11 -0.74342745D+04 2.6251976 0.0003530 302.810 266.180 EM

133.011

12 -0.74325031D+04 1.7714329 0.0002383 310.205 261.401 EM

130.394

13 -0.74313371D+04 1.1659193 0.0001569 316.208 257.438 EM

128.353

14 -0.74305714D+04 0.7657304 0.0001030 320.992 254.234 EM

126.774

TECHNICAL 8 OUTPUT FOR STARTING VALUE SET 23

ITER LOGLIKELIHOOD ABS CHANGE REL CHANGE CLASS COUNTS ALGORITHM

1 -0.21796787D+05 0.0000000 0.0000000 148.290 136.524 EM

417.186

2 -0.76603806D+04 ************ 0.6485546 177.684 128.114 EM

396.202

3 -0.75957853D+04 64.5952958 0.0084324 204.261 128.326 EM

369.413

4 -0.75632350D+04 32.5503066 0.0042853 229.870 128.621 EM

343.509

5 -0.75371185D+04 26.1165494 0.0034531 252.616 127.931 EM

321.453

6 -0.75190860D+04 18.0324545 0.0023925 271.207 126.974 EM

303.819

7 -0.75083460D+04 10.7400361 0.0014284 285.644 126.524 EM

289.832

8 -0.75023177D+04 6.0283107 0.0008029 296.470 126.846 EM

278.684

9 -0.74989419D+04 3.3757334 0.0004500 304.430 127.894 EM

269.676

10 -0.74969420D+04 1.9999296 0.0002667 310.236 129.531 EM

262.233

11 -0.74956374D+04 1.3045988 0.0001740 314.463 131.620 EM

255.917

12 -0.74946841D+04 0.9533052 0.0001272 317.539 134.056 EM

250.405

TECHNICAL 8 OUTPUT FOR STARTING VALUE SET 24

ITER LOGLIKELIHOOD ABS CHANGE REL CHANGE CLASS COUNTS ALGORITHM

1 -0.20182717D+05 0.0000000 0.0000000 207.156 121.197 EM

373.647

2 -0.75629788D+04 ************ 0.6252745 231.730 117.880 EM

352.390

3 -0.75106093D+04 52.3694634 0.0069244 243.423 128.817 EM

329.760

4 -0.74938993D+04 16.7100209 0.0022249 247.381 144.807 EM

309.812

5 -0.74841422D+04 9.7570777 0.0013020 247.266 163.029 EM

291.705

6 -0.74757072D+04 8.4350017 0.0011270 245.098 182.187 EM

274.715

7 -0.74677320D+04 7.9751690 0.0010668 241.873 201.371 EM

258.757

8 -0.74603855D+04 7.3464910 0.0009838 238.122 219.847 EM

244.031

9 -0.74539016D+04 6.4839556 0.0008691 234.208 237.059 EM

230.732

10 -0.74484119D+04 5.4897131 0.0007365 230.448 252.603 EM

218.949

11 -0.74439685D+04 4.4433777 0.0005966 227.114 266.211 EM

208.675

12 -0.74405480D+04 3.4204570 0.0004595 224.403 277.762 EM

199.835

13 -0.74380378D+04 2.5102152 0.0003374 222.407 287.296 EM

192.297

14 -0.74362552D+04 1.7825916 0.0002397 221.120 294.985 EM

185.895

15 -0.74349994D+04 1.2557800 0.0001689 220.463 301.094 EM

180.443

16 -0.74340980D+04 0.9014647 0.0001212 220.324 305.912 EM

175.764

TECHNICAL 8 OUTPUT FOR STARTING VALUE SET 25

ITER LOGLIKELIHOOD ABS CHANGE REL CHANGE CLASS COUNTS ALGORITHM

1 -0.15034392D+05 0.0000000 0.0000000 514.712 66.797 EM

120.491

2 -0.77151051D+04 7319.2872191 0.4868363 495.872 76.049 EM

130.079

3 -0.76438250D+04 71.2800573 0.0092390 478.424 84.877 EM

138.699

4 -0.75988998D+04 44.9251668 0.0058773 467.778 90.619 EM

143.604

5 -0.75545082D+04 44.3916566 0.0058419 456.228 95.921 EM

149.851

6 -0.75247665D+04 29.7416915 0.0039369 441.003 102.464 EM

158.533

7 -0.75072638D+04 17.5026628 0.0023260 423.270 110.673 EM

168.056

8 -0.74943229D+04 12.9409761 0.0017238 404.629 120.339 EM

177.032

9 -0.74830082D+04 11.3146210 0.0015098 386.302 131.272 EM

184.426

10 -0.74720437D+04 10.9645851 0.0014653 368.964 143.437 EM

189.599

11 -0.74609548D+04 11.0888323 0.0014840 353.447 156.283 EM

192.270

12 -0.74511614D+04 9.7933833 0.0013126 341.031 168.383 EM

192.586

13 -0.74445046D+04 6.6568682 0.0008934 332.166 178.664 EM

191.171

14 -0.74406158D+04 3.8887854 0.0005224 326.249 186.993 EM

188.758

15 -0.74382923D+04 2.3235238 0.0003123 322.454 193.684 EM

185.862

16 -0.74367658D+04 1.5264731 0.0002052 320.128 199.101 EM

182.771

17 -0.74356678D+04 1.0979853 0.0001476 318.815 203.547 EM

179.637

18 -0.74348229D+04 0.8448957 0.0001136 318.202 207.255 EM

176.543

TECHNICAL 8 OUTPUT FOR STARTING VALUE SET 26

ITER LOGLIKELIHOOD ABS CHANGE REL CHANGE CLASS COUNTS ALGORITHM

1 -0.17317795D+05 0.0000000 0.0000000 27.116 106.065 EM

568.818

2 -0.76628003D+04 9654.9943044 0.5575187 28.491 134.648 EM

538.862

3 -0.76025091D+04 60.2911964 0.0078680 33.910 163.048 EM

505.043

4 -0.75713209D+04 31.1882401 0.0041024 36.648 190.539 EM

474.813

5 -0.75420386D+04 29.2822846 0.0038675 39.760 216.147 EM

446.093

6 -0.75110898D+04 30.9487786 0.0041035 46.896 237.083 EM

418.020

7 -0.74754557D+04 35.6340856 0.0047442 58.667 251.516 EM

391.817

8 -0.74486669D+04 26.7888200 0.0035836 69.776 259.371 EM

372.853

9 -0.74371785D+04 11.4884623 0.0015424 77.609 262.671 EM

361.720

10 -0.74333325D+04 3.8459669 0.0005171 83.105 263.516 EM

355.379

11 -0.74317688D+04 1.5637093 0.0002104 87.216 263.114 EM

351.670

12 -0.74309320D+04 0.8367756 0.0001126 90.443 262.110 EM

349.448

TECHNICAL 8 OUTPUT FOR STARTING VALUE SET 27

ITER LOGLIKELIHOOD ABS CHANGE REL CHANGE CLASS COUNTS ALGORITHM

1 -0.13836634D+05 0.0000000 0.0000000 371.536 99.903 EM

230.561

2 -0.77550103D+04 6081.6234551 0.4395306 348.316 110.248 EM

243.437

3 -0.76541066D+04 100.9036309 0.0130114 315.538 139.198 EM

247.264

4 -0.75819200D+04 72.1866228 0.0094311 289.516 169.519 EM

242.965

5 -0.75248204D+04 57.0996269 0.0075310 273.272 193.734 EM

234.995

6 -0.74871631D+04 37.6573040 0.0050044 266.766 209.617 EM

225.617

7 -0.74654354D+04 21.7276266 0.0029020 268.232 218.036 EM

215.732

8 -0.74528922D+04 12.5432561 0.0016802 274.575 221.535 EM

205.889

9 -0.74449617D+04 7.9304423 0.0010641 282.822 222.521 EM

196.658

10 -0.74397841D+04 5.1776988 0.0006955 290.992 222.501 EM

188.506

11 -0.74365490D+04 3.2350766 0.0004348 298.133 222.261 EM

181.606

12 -0.74346227D+04 1.9262586 0.0002590 303.982 222.150 EM

175.868

13 -0.74334793D+04 1.1434645 0.0001538 308.621 222.289 EM

171.091

14 -0.74327631D+04 0.7161738 0.0000963 312.259 222.679 EM

167.061

TECHNICAL 8 OUTPUT FOR STARTING VALUE SET 28

ITER LOGLIKELIHOOD ABS CHANGE REL CHANGE CLASS COUNTS ALGORITHM

1 -0.20488719D+05 0.0000000 0.0000000 75.266 336.957 EM

289.777

2 -0.75037657D+04 ************ 0.6337611 73.995 332.648 EM

295.357

3 -0.74867808D+04 16.9848881 0.0022635 73.947 326.304 EM

301.749

4 -0.74839502D+04 2.8305580 0.0003781 74.284 320.534 EM

307.182

5 -0.74828572D+04 1.0930313 0.0001461 74.729 315.659 EM

311.612

6 -0.74822935D+04 0.5637202 0.0000753 75.182 311.605 EM

315.213

TECHNICAL 8 OUTPUT FOR STARTING VALUE SET 29

ITER LOGLIKELIHOOD ABS CHANGE REL CHANGE CLASS COUNTS ALGORITHM

1 -0.29105365D+05 0.0000000 0.0000000 261.731 144.731 EM

295.538

2 -0.76683434D+04 ************ 0.7365316 265.128 157.753 EM

279.120

3 -0.76030975D+04 65.2458378 0.0085085 265.404 175.536 EM

261.061

4 -0.75767670D+04 26.3305372 0.0034631 264.344 195.025 EM

242.631

5 -0.75536845D+04 23.0824722 0.0030465 262.808 214.311 EM

224.880

6 -0.75319729D+04 21.7116383 0.0028743 260.238 230.308 EM

211.454

7 -0.75143204D+04 17.6524426 0.0023437 256.038 240.797 EM

205.165

8 -0.75002462D+04 14.0742303 0.0018730 250.340 244.715 EM

206.944

9 -0.74873649D+04 12.8813202 0.0017175 243.814 241.929 EM

216.257

10 -0.74738272D+04 13.5376425 0.0018081 237.228 233.419 EM

231.353

11 -0.74601102D+04 13.7170606 0.0018353 231.310 221.727 EM

248.963

12 -0.74488958D+04 11.2144009 0.0015032 226.606 209.919 EM

265.475

13 -0.74418570D+04 7.0387529 0.0009449 223.280 199.687 EM

279.033

14 -0.74380334D+04 3.8236171 0.0005138 221.188 191.309 EM

289.503

15 -0.74359193D+04 2.1140629 0.0002842 220.075 184.503 EM

297.422

16 -0.74346382D+04 1.2811857 0.0001723 219.692 178.909 EM

303.399

17 -0.74337844D+04 0.8537794 0.0001148 219.834 174.223 EM

307.944

TECHNICAL 8 OUTPUT FOR STARTING VALUE SET 30

ITER LOGLIKELIHOOD ABS CHANGE REL CHANGE CLASS COUNTS ALGORITHM

1 -0.26281194D+05 0.0000000 0.0000000 411.970 126.113 EM

163.917

2 -0.77929443D+04 ************ 0.7034783 423.756 102.210 EM

176.034

3 -0.76505363D+04 142.4080178 0.0182740 415.478 99.870 EM

186.653

4 -0.75578337D+04 92.7026058 0.0121171 397.613 113.900 EM

190.486

5 -0.75111380D+04 46.6956815 0.0061784 376.845 135.001 EM

190.154

6 -0.74901991D+04 20.9388867 0.0027877 357.883 156.780 EM

187.337

7 -0.74778129D+04 12.3862574 0.0016537 341.861 177.428 EM

182.710

8 -0.74688582D+04 8.9546946 0.0011975 328.279 196.783 EM

176.938

9 -0.74615602D+04 7.2979598 0.0009771 316.390 214.998 EM

170.612

10 -0.74551989D+04 6.3612809 0.0008525 305.680 232.165 EM

164.156

11 -0.74495464D+04 5.6525686 0.0007582 295.885 248.248 EM

157.867

12 -0.74445904D+04 4.9559456 0.0006653 286.927 263.096 EM

151.977

13 -0.74404100D+04 4.1803684 0.0005615 278.842 276.481 EM

146.677

14 -0.74370814D+04 3.3286048 0.0004474 271.710 288.192 EM

142.098

15 -0.74345935D+04 2.4879036 0.0003345 265.586 298.125 EM

138.289

16 -0.74328294D+04 1.7641151 0.0002373 260.467 306.321 EM

135.212

17 -0.74316188D+04 1.2106207 0.0001629 256.292 312.940 EM

132.767

18 -0.74307996D+04 0.8191855 0.0001102 252.960 318.206 EM

130.834

TECHNICAL 8 OUTPUT FOR STARTING VALUE SET 31

ITER LOGLIKELIHOOD ABS CHANGE REL CHANGE CLASS COUNTS ALGORITHM

1 -0.17676382D+05 0.0000000 0.0000000 55.129 505.783 EM

141.088

2 -0.77391923D+04 9937.1893426 0.5621733 77.810 475.108 EM

149.083

3 -0.75989151D+04 140.2772281 0.0181256 108.467 428.726 EM

164.807

4 -0.75190673D+04 79.8477116 0.0105078 135.611 390.547 EM

175.842

5 -0.74787887D+04 40.2786495 0.0053569 157.604 363.212 EM

181.184

6 -0.74562817D+04 22.5069862 0.0030094 174.021 345.808 EM

182.172

7 -0.74447878D+04 11.4939239 0.0015415 185.754 335.526 EM

180.721

8 -0.74392298D+04 5.5580276 0.0007466 194.323 329.350 EM

178.328

9 -0.74364209D+04 2.8088168 0.0003776 200.790 325.554 EM

175.656

10 -0.74348821D+04 1.5388357 0.0002069 205.814 323.249 EM

172.937

11 -0.74339374D+04 0.9446825 0.0001271 209.818 321.930 EM

170.251

TECHNICAL 8 OUTPUT FOR STARTING VALUE SET 32

ITER LOGLIKELIHOOD ABS CHANGE REL CHANGE CLASS COUNTS ALGORITHM

1 -0.21674178D+05 0.0000000 0.0000000 549.307 146.539 EM

6.154

2 -0.76932406D+04 ************ 0.6450504 528.051 168.026 EM

5.923

3 -0.76056076D+04 87.6329280 0.0113909 501.556 193.145 EM

7.299

4 -0.75758167D+04 29.7909309 0.0039170 476.147 216.286 EM

9.567

5 -0.75543852D+04 21.4314939 0.0028289 451.183 237.323 EM

13.494

6 -0.75296671D+04 24.7181290 0.0032720 426.805 255.398 EM

19.797

7 -0.75098216D+04 19.8454460 0.0026356 405.712 269.773 EM

26.515

8 -0.74992988D+04 10.5228044 0.0014012 388.551 281.073 EM

32.376

9 -0.74935513D+04 5.7474788 0.0007664 374.406 290.184 EM

37.410

10 -0.74899653D+04 3.5860197 0.0004785 362.554 297.687 EM

41.759

11 -0.74875475D+04 2.4177954 0.0003228 352.541 303.926 EM

45.533

12 -0.74858564D+04 1.6911118 0.0002259 344.047 309.133 EM

48.820

13 -0.74846496D+04 1.2068509 0.0001612 336.823 313.484 EM

51.693

14 -0.74837763D+04 0.8732265 0.0001167 330.662 317.124 EM

54.214

TECHNICAL 8 OUTPUT FOR STARTING VALUE SET 33

ITER LOGLIKELIHOOD ABS CHANGE REL CHANGE CLASS COUNTS ALGORITHM

1 -0.22114730D+05 0.0000000 0.0000000 5.422 239.566 EM

457.012

2 -0.77497865D+04 ************ 0.6495645 3.647 250.362 EM

447.991

3 -0.76538575D+04 95.9289258 0.0123783 3.506 271.434 EM

427.060

4 -0.75664716D+04 87.3859279 0.0114172 3.918 292.153 EM

405.929

5 -0.75329636D+04 33.5080338 0.0044285 4.511 307.499 EM

389.990

6 -0.75250616D+04 7.9019940 0.0010490 5.054 319.079 EM

377.867

7 -0.75217496D+04 3.3119731 0.0004401 5.519 327.978 EM

368.504

8 -0.75198724D+04 1.8771799 0.0002496 5.889 334.818 EM

361.293

9 -0.75187689D+04 1.1035469 0.0001468 6.175 340.064 EM

355.761

10 -0.75181107D+04 0.6581499 0.0000875 6.401 344.081 EM

351.519

TECHNICAL 8 OUTPUT FOR STARTING VALUE SET 34

ITER LOGLIKELIHOOD ABS CHANGE REL CHANGE CLASS COUNTS ALGORITHM

1 -0.19913326D+05 0.0000000 0.0000000 204.079 486.526 EM

11.394

2 -0.75786254D+04 ************ 0.6194194 235.884 455.069 EM

11.047

3 -0.75501686D+04 28.4567906 0.0037549 260.408 429.059 EM

12.533

4 -0.75352823D+04 14.8862364 0.0019716 278.262 408.665 EM

15.073

5 -0.75265698D+04 8.7125447 0.0011562 290.576 393.084 EM

18.339

6 -0.75214605D+04 5.1093322 0.0006788 298.572 381.345 EM

22.083

7 -0.75184046D+04 3.0558249 0.0004063 303.399 372.473 EM

26.128

8 -0.75164483D+04 1.9563479 0.0002602 305.991 365.622 EM

30.387

9 -0.75150438D+04 1.4044425 0.0001868 307.031 360.128 EM

34.841

10 -0.75138991D+04 1.1447447 0.0001523 306.997 355.504 EM

39.498

11 -0.75128684D+04 1.0307112 0.0001372 306.219 351.413 EM

44.368

12 -0.75118851D+04 0.9832497 0.0001309 304.921 347.632 EM

49.447

TECHNICAL 8 OUTPUT FOR STARTING VALUE SET 35

ITER LOGLIKELIHOOD ABS CHANGE REL CHANGE CLASS COUNTS ALGORITHM

1 -0.16654632D+05 0.0000000 0.0000000 7.227 678.458 EM

16.316

2 -0.77667271D+04 8887.9047272 0.5336596 14.455 655.804 EM

31.741

3 -0.77001840D+04 66.5431860 0.0085677 22.245 628.411 EM

51.345

4 -0.76607317D+04 39.4522779 0.0051236 28.586 601.779 EM

71.635

5 -0.76328208D+04 27.9108517 0.0036434 34.426 575.783 EM

91.791

6 -0.76103120D+04 22.5088089 0.0029490 40.276 550.216 EM

111.508

7 -0.75907938D+04 19.5182068 0.0025647 46.392 524.938 EM

130.670

8 -0.75730233D+04 17.7705437 0.0023411 52.827 500.059 EM

149.114

9 -0.75567251D+04 16.2981480 0.0021521 59.439 476.077 EM

166.484

10 -0.75423326D+04 14.3925611 0.0019046 66.019 453.690 EM

182.291

11 -0.75303498D+04 11.9827695 0.0015887 72.487 433.435 EM

196.078

12 -0.75208573D+04 9.4924861 0.0012606 78.860 415.592 EM

207.548

13 -0.75136038D+04 7.2535190 0.0009645 85.129 400.268 EM

216.602

14 -0.75082327D+04 5.3710932 0.0007148 91.258 387.430 EM

223.311

15 -0.75043430D+04 3.8896843 0.0005181 97.238 376.875 EM

227.888

16 -0.75015184D+04 2.8246198 0.0003764 103.100 368.267 EM

230.633

17 -0.74993935D+04 2.1249286 0.0002833 108.898 361.228 EM

231.874

18 -0.74977007D+04 1.6927408 0.0002257 114.671 355.409 EM

231.920

19 -0.74962692D+04 1.4315597 0.0001909 120.438 350.530 EM

231.032

20 -0.74949965D+04 1.2727021 0.0001698 126.196 346.380 EM

229.424

21 -0.74938183D+04 1.1781992 0.0001572 131.932 342.807 EM

227.261

22 -0.74926837D+04 1.1345845 0.0001514 137.639 339.690 EM

224.671

23 -0.74915372D+04 1.1465154 0.0001530 143.326 336.926 EM

221.748

24 -0.74903038D+04 1.2333752 0.0001646 149.034 334.405 EM

218.561

25 -0.74888737D+04 1.4301277 0.0001909 154.837 332.004 EM

215.159

26 -0.74870835D+04 1.7902069 0.0002390 160.864 329.561 EM

211.575

27 -0.74847041D+04 2.3793977 0.0003178 167.317 326.867 EM

207.816

28 -0.74814668D+04 3.2372367 0.0004325 174.488 323.661 EM

203.850

29 -0.74771735D+04 4.2933162 0.0005739 182.737 319.662 EM

199.601

30 -0.74718800D+04 5.2934553 0.0007079 192.393 314.652 EM

194.955

31 -0.74660076D+04 5.8724048 0.0007859 203.612 308.586 EM

189.802

32 -0.74601615D+04 5.8461816 0.0007830 216.239 301.651 EM

184.111

33 -0.74547302D+04 5.4312508 0.0007280 229.817 294.182 EM

178.001

34 -0.74497715D+04 4.9587053 0.0006652 243.769 286.531 EM

171.699

35 -0.74390337D+04 10.7377774 0.0014414 300.269 257.318 FS

144.414

36 -0.74360558D+04 2.9778949 0.0004003 311.032 250.166 FS

140.802

37 -0.74315886D+04 4.4672362 0.0006008 315.289 248.125 EM

138.586

38 -0.74307504D+04 0.8382274 0.0001128 319.194 246.185 EM

136.621

TECHNICAL 8 OUTPUT FOR STARTING VALUE SET 36

ITER LOGLIKELIHOOD ABS CHANGE REL CHANGE CLASS COUNTS ALGORITHM

1 -0.23071557D+05 0.0000000 0.0000000 196.962 276.707 EM

228.331

2 -0.75920791D+04 ************ 0.6709334 220.938 247.621 EM

233.440

3 -0.75037983D+04 88.2807982 0.0116280 236.321 234.965 EM

230.714

4 -0.74756765D+04 28.1218922 0.0037477 243.323 236.360 EM

222.318

5 -0.74616961D+04 13.9803482 0.0018701 244.933 245.404 EM

211.662

6 -0.74523019D+04 9.3942305 0.0012590 243.578 257.594 EM

200.829

7 -0.74452442D+04 7.0577230 0.0009471 240.889 270.301 EM

190.810

8 -0.74400181D+04 5.2260071 0.0007019 237.889 282.037 EM

182.073

9 -0.74364280D+04 3.5901391 0.0004825 235.166 292.081 EM

174.753

10 -0.74341547D+04 2.2732847 0.0003057 233.000 300.253 EM

168.747

11 -0.74327890D+04 1.3656978 0.0001837 231.465 306.691 EM

163.844

12 -0.74319744D+04 0.8145789 0.0001096 230.515 311.675 EM

159.810

TECHNICAL 8 OUTPUT FOR STARTING VALUE SET 37

ITER LOGLIKELIHOOD ABS CHANGE REL CHANGE CLASS COUNTS ALGORITHM

1 -0.17049907D+05 0.0000000 0.0000000 161.289 329.077 EM

211.634

2 -0.76331302D+04 9416.7766521 0.5523066 156.848 318.279 EM

226.873

3 -0.75705961D+04 62.5340977 0.0081925 155.684 303.032 EM

243.283

4 -0.75346886D+04 35.9075581 0.0047430 153.678 288.491 EM

259.831

5 -0.75140794D+04 20.6091717 0.0027352 150.853 276.665 EM

274.482

6 -0.75042753D+04 9.8040434 0.0013048 148.355 267.318 EM

286.328

7 -0.74996124D+04 4.6629714 0.0006214 146.706 259.911 EM

295.384

8 -0.74971910D+04 2.4214050 0.0003229 145.922 253.932 EM

302.146

9 -0.74957620D+04 1.4289745 0.0001906 145.855 248.953 EM

307.191

10 -0.74947891D+04 0.9728476 0.0001298 146.352 244.655 EM

310.993

TECHNICAL 8 OUTPUT FOR STARTING VALUE SET 38

ITER LOGLIKELIHOOD ABS CHANGE REL CHANGE CLASS COUNTS ALGORITHM

1 -0.20258112D+05 0.0000000 0.0000000 277.415 373.847 EM

50.738

2 -0.76015716D+04 ************ 0.6247641 293.243 368.473 EM

40.285

3 -0.75369774D+04 64.5941341 0.0084975 301.119 362.517 EM

38.364

4 -0.75234441D+04 13.5333346 0.0017956 305.286 355.969 EM

40.744

5 -0.75174914D+04 5.9526561 0.0007912 307.356 348.892 EM

45.751

6 -0.75135432D+04 3.9482663 0.0005252 308.024 341.257 EM

52.719

7 -0.75099691D+04 3.5740714 0.0004757 307.667 332.808 EM

61.525

8 -0.75059753D+04 3.9937715 0.0005318 306.532 323.289 EM

72.179

9 -0.75012690D+04 4.7063545 0.0006270 304.810 312.630 EM

84.560

10 -0.74959540D+04 5.3149819 0.0007085 302.642 301.031 EM

98.326

11 -0.74903709D+04 5.5831452 0.0007448 300.102 288.869 EM

113.029

12 -0.74848469D+04 5.5239471 0.0007375 297.189 276.510 EM

128.301

13 -0.74795118D+04 5.3351293 0.0007128 293.851 264.216 EM

143.933

14 -0.74743243D+04 5.1874881 0.0006936 290.022 252.168 EM

159.810

15 -0.74692171D+04 5.1072147 0.0006833 285.664 240.515 EM

175.821

16 -0.74641795D+04 5.0376077 0.0006744 280.792 229.376 EM

191.833

17 -0.74592424D+04 4.9370355 0.0006614 275.472 218.824 EM

207.703

18 -0.74544462D+04 4.7962386 0.0006430 269.813 208.904 EM

223.283

19 -0.74498452D+04 4.6010311 0.0006172 263.962 199.648 EM

238.391

20 -0.74455384D+04 4.3067525 0.0005781 258.122 191.105 EM

252.773

21 -0.74416814D+04 3.8570570 0.0005180 252.545 183.352 EM

266.104

22 -0.74384411D+04 3.2402719 0.0004354 247.489 176.460 EM

278.051

23 -0.74359153D+04 2.5258106 0.0003396 243.159 170.467 EM

288.374

24 -0.74340829D+04 1.8323539 0.0002464 239.662 165.344 EM

296.994

25 -0.74328251D+04 1.2577821 0.0001692 237.004 161.004 EM

303.993

26 -0.74319864D+04 0.8387071 0.0001128 235.108 157.328 EM

309.564

TECHNICAL 8 OUTPUT FOR STARTING VALUE SET 39

ITER LOGLIKELIHOOD ABS CHANGE REL CHANGE CLASS COUNTS ALGORITHM

1 -0.20202296D+05 0.0000000 0.0000000 208.981 461.514 EM

31.505

2 -0.77562028D+04 ************ 0.6160732 188.530 453.859 EM

59.611

3 -0.76555583D+04 100.6445474 0.0129760 180.368 434.927 EM

86.705

4 -0.75889086D+04 66.6496326 0.0087060 181.328 411.245 EM

109.427

5 -0.75417077D+04 47.2009280 0.0062197 187.814 387.784 EM

126.402

6 -0.75113299D+04 30.3778186 0.0040280 196.741 367.947 EM

137.312

7 -0.74924709D+04 18.8590112 0.0025107 207.047 351.961 EM

142.992

8 -0.74804336D+04 12.0373033 0.0016066 218.042 339.068 EM

144.890

9 -0.74714973D+04 8.9362321 0.0011946 229.270 328.335 EM

144.395

10 -0.74636659D+04 7.8314098 0.0010482 240.728 318.816 EM

142.457

11 -0.74564351D+04 7.2308300 0.0009688 252.545 309.854 EM

139.602

12 -0.74499427D+04 6.4923517 0.0008707 264.650 301.171 EM

136.178

13 -0.74443900D+04 5.5527915 0.0007453 276.625 292.818 EM

132.557

14 -0.74399105D+04 4.4794370 0.0006017 287.865 285.017 EM

129.119

15 -0.74365419D+04 3.3685798 0.0004528 297.859 277.996 EM

126.145

16 -0.74341658D+04 2.3761334 0.0003195 306.355 271.887 EM

123.759

17 -0.74325483D+04 1.6174923 0.0002176 313.348 266.707 EM

121.946

18 -0.74314497D+04 1.0986145 0.0001478 318.988 262.395 EM

120.617

19 -0.74306901D+04 0.7596034 0.0001022 323.483 258.854 EM

119.663

TECHNICAL 8 OUTPUT FOR STARTING VALUE SET 40

ITER LOGLIKELIHOOD ABS CHANGE REL CHANGE CLASS COUNTS ALGORITHM

1 -0.20387143D+05 0.0000000 0.0000000 632.371 1.161 EM

68.468

2 -0.78154893D+04 ************ 0.6166462 620.896 0.629 EM

80.475

3 -0.77522453D+04 63.2440434 0.0080921 599.378 0.942 EM

101.680

4 -0.76799357D+04 72.3095787 0.0093276 570.827 4.378 EM

126.795

5 -0.76212479D+04 58.6877830 0.0076417 537.453 12.455 EM

152.091

6 -0.75851077D+04 36.1402116 0.0047420 505.167 20.458 EM

176.375

7 -0.75613794D+04 23.7282750 0.0031283 474.769 28.315 EM

198.915

8 -0.75433290D+04 18.0504107 0.0023872 446.583 36.172 EM

219.245

9 -0.75296330D+04 13.6960089 0.0018156 421.394 43.609 EM

236.997

10 -0.75197984D+04 9.8346352 0.0013061 399.724 50.229 EM

252.048

11 -0.75130987D+04 6.6996740 0.0008909 381.554 55.863 EM

264.583

12 -0.75086443D+04 4.4544296 0.0005929 366.466 60.536 EM

274.998

13 -0.75056389D+04 3.0054020 0.0004003 353.879 64.364 EM

283.758

14 -0.75034991D+04 2.1397130 0.0002851 343.213 67.490 EM

291.297

15 -0.75018445D+04 1.6546794 0.0002205 333.969 70.050 EM

297.981

16 -0.75004422D+04 1.4022794 0.0001869 325.749 72.162 EM

304.089

17 -0.74991575D+04 1.2847169 0.0001713 318.250 73.931 EM

309.820

18 -0.74979191D+04 1.2384114 0.0001651 311.253 75.449 EM

315.299

19 -0.74966980D+04 1.2210736 0.0001629 304.614 76.797 EM

320.589

20 -0.74954935D+04 1.2044348 0.0001607 298.249 78.048 EM

325.703

21 -0.74943226D+04 1.1709037 0.0001562 292.124 79.262 EM

330.615

22 -0.74932104D+04 1.1122773 0.0001484 286.234 80.491 EM

335.275

23 -0.74921819D+04 1.0284859 0.0001373 280.598 81.776 EM

339.626

24 -0.74912562D+04 0.9256297 0.0001235 275.238 83.145 EM

343.617

TECHNICAL 8 OUTPUT FOR STARTING VALUE SET 41

ITER LOGLIKELIHOOD ABS CHANGE REL CHANGE CLASS COUNTS ALGORITHM

1 -0.24898751D+05 0.0000000 0.0000000 280.386 152.764 EM

268.850

2 -0.75423126D+04 ************ 0.6970807 265.802 147.913 EM

288.285

3 -0.75128205D+04 29.4921060 0.0039102 251.492 145.600 EM

304.908

4 -0.74995470D+04 13.2735101 0.0017668 239.812 143.899 EM

318.289

5 -0.74917553D+04 7.7916576 0.0010390 230.967 142.293 EM

328.740

6 -0.74871789D+04 4.5764098 0.0006109 224.652 140.746 EM

336.602

7 -0.74846276D+04 2.5512781 0.0003408 220.389 139.332 EM

342.279

8 -0.74832844D+04 1.3432635 0.0001795 217.685 138.085 EM

346.230

9 -0.74826058D+04 0.6785397 0.0000907 216.115 136.990 EM

348.895

TECHNICAL 8 OUTPUT FOR STARTING VALUE SET 42

ITER LOGLIKELIHOOD ABS CHANGE REL CHANGE CLASS COUNTS ALGORITHM

1 -0.15740324D+05 0.0000000 0.0000000 165.238 0.024 EM

536.739

2 -0.77525463D+04 7987.7772806 0.5074722 173.888 0.162 EM

527.950

3 -0.77011192D+04 51.4271355 0.0066336 187.105 1.981 EM

512.914

4 -0.76520828D+04 49.0364040 0.0063674 200.740 10.423 EM

490.837

5 -0.75874997D+04 64.5831000 0.0084399 214.355 22.052 EM

465.592

6 -0.75375992D+04 49.9004191 0.0065767 226.219 32.717 EM

443.064

7 -0.75134264D+04 24.1728538 0.0032070 235.692 41.438 EM

424.870

8 -0.75026106D+04 10.8157821 0.0014395 242.723 48.478 EM

410.799

9 -0.74971400D+04 5.4706437 0.0007292 247.485 54.312 EM

400.204

10 -0.74940692D+04 3.0707267 0.0004096 250.386 59.336 EM

392.277

11 -0.74921880D+04 1.8811968 0.0002510 251.872 63.812 EM

386.315

12 -0.74909461D+04 1.2419721 0.0001658 252.314 67.898 EM

381.787

13 -0.74900723D+04 0.8737232 0.0001166 251.992 71.693 EM

378.315

TECHNICAL 8 OUTPUT FOR STARTING VALUE SET 43

ITER LOGLIKELIHOOD ABS CHANGE REL CHANGE CLASS COUNTS ALGORITHM

1 -0.20556598D+05 0.0000000 0.0000000 290.051 312.164 EM

99.785

2 -0.77203061D+04 ************ 0.6244366 273.715 302.509 EM

125.776

3 -0.76164508D+04 103.8553867 0.0134522 259.480 289.534 EM

152.986

4 -0.75754976D+04 40.9531925 0.0053769 247.068 277.649 EM

177.283

5 -0.75523244D+04 23.1731671 0.0030590 235.222 267.391 EM

199.387

6 -0.75369892D+04 15.3352022 0.0020305 223.998 258.259 EM

219.743

7 -0.75257910D+04 11.1982177 0.0014858 213.697 249.846 EM

238.457

8 -0.75170923D+04 8.6986721 0.0011558 204.508 241.972 EM

255.520

9 -0.75101740D+04 6.9182906 0.0009203 196.467 234.602 EM

270.931

10 -0.75046221D+04 5.5519355 0.0007393 189.509 227.744 EM

284.746

11 -0.75001201D+04 4.5020123 0.0005999 183.546 221.392 EM

297.062

12 -0.74964146D+04 3.7054316 0.0004940 178.489 215.516 EM

307.995

13 -0.74933230D+04 3.0916647 0.0004124 174.266 210.087 EM

317.647

14 -0.74907371D+04 2.5858796 0.0003451 170.820 205.095 EM

326.085

15 -0.74886119D+04 2.1251394 0.0002837 168.102 200.555 EM

333.343

16 -0.74869332D+04 1.6787008 0.0002242 166.069 196.487 EM

339.444

17 -0.74856780D+04 1.2552080 0.0001677 164.670 192.897 EM

344.433

18 -0.74847926D+04 0.8854481 0.0001183 163.842 189.759 EM

348.399

TECHNICAL 8 OUTPUT FOR STARTING VALUE SET 44

ITER LOGLIKELIHOOD ABS CHANGE REL CHANGE CLASS COUNTS ALGORITHM

1 -0.21447006D+05 0.0000000 0.0000000 173.330 296.653 EM

232.016

2 -0.75250019D+04 ************ 0.6491351 190.478 282.435 EM

229.087

3 -0.74742276D+04 50.7743022 0.0067474 201.495 276.440 EM

224.065

4 -0.74575507D+04 16.6768412 0.0022312 207.378 278.018 EM

216.604

5 -0.74482300D+04 9.3207532 0.0012498 210.362 283.471 EM

208.167

6 -0.74423831D+04 5.8468958 0.0007850 211.946 290.041 EM

200.013

7 -0.74387619D+04 3.6211569 0.0004866 212.997 296.249 EM

192.754

8 -0.74365524D+04 2.2095608 0.0002970 213.938 301.547 EM

186.515

9 -0.74351733D+04 1.3790698 0.0001854 214.937 305.867 EM

181.196

10 -0.74342547D+04 0.9186270 0.0001236 216.039 309.332 EM

176.628

TECHNICAL 8 OUTPUT FOR STARTING VALUE SET 45

ITER LOGLIKELIHOOD ABS CHANGE REL CHANGE CLASS COUNTS ALGORITHM

1 -0.16953660D+05 0.0000000 0.0000000 151.458 74.263 EM

476.279

2 -0.76614364D+04 9292.2240191 0.5480954 154.916 80.888 EM

466.197

3 -0.75976328D+04 63.8036020 0.0083279 156.698 99.792 EM

445.510

4 -0.75334260D+04 64.2068024 0.0084509 157.594 120.597 EM

423.809

5 -0.75002211D+04 33.2048524 0.0044077 161.084 133.685 EM

407.232

6 -0.74901965D+04 10.0246827 0.0013366 165.161 141.304 EM

395.535

7 -0.74868399D+04 3.3565304 0.0004481 168.339 146.492 EM

387.169

8 -0.74853431D+04 1.4968796 0.0001999 170.491 150.502 EM

381.007

9 -0.74845287D+04 0.8143647 0.0001088 171.829 153.814 EM

376.357

TECHNICAL 8 OUTPUT FOR STARTING VALUE SET 46

ITER LOGLIKELIHOOD ABS CHANGE REL CHANGE CLASS COUNTS ALGORITHM

1 -0.26910991D+05 0.0000000 0.0000000 8.393 175.502 EM

518.104

2 -0.77851073D+04 ************ 0.7107090 8.004 185.247 EM

508.750

3 -0.77209190D+04 64.1882849 0.0082450 7.956 200.081 EM

493.962

4 -0.76642583D+04 56.6607240 0.0073386 8.303 211.647 EM

482.050

5 -0.76050489D+04 59.2093955 0.0077254 10.596 223.871 EM

467.532

6 -0.75564736D+04 48.5752206 0.0063872 17.095 238.892 EM

446.014

7 -0.75218141D+04 34.6595655 0.0045867 25.196 253.388 EM

423.415

8 -0.75052113D+04 16.6027715 0.0022073 32.108 265.734 EM

404.158

9 -0.74972101D+04 8.0012427 0.0010661 37.872 276.148 EM

387.980

10 -0.74924939D+04 4.7161910 0.0006291 42.724 284.952 EM

374.324

11 -0.74893730D+04 3.1208325 0.0004165 46.849 292.413 EM

362.738

12 -0.74872038D+04 2.1691997 0.0002896 50.382 298.744 EM

352.874

13 -0.74856592D+04 1.5446522 0.0002063 53.425 304.120 EM

344.455

14 -0.74845421D+04 1.1170994 0.0001492 56.055 308.691 EM

337.255

15 -0.74837235D+04 0.8185526 0.0001094 58.336 312.583 EM

331.080

TECHNICAL 8 OUTPUT FOR STARTING VALUE SET 47

ITER LOGLIKELIHOOD ABS CHANGE REL CHANGE CLASS COUNTS ALGORITHM

1 -0.14205139D+05 0.0000000 0.0000000 649.860 0.004 EM

52.137

2 -0.77149627D+04 6490.1765457 0.4568893 623.997 0.002 EM

78.001

3 -0.76773150D+04 37.6477177 0.0048798 595.819 0.003 EM

106.179

4 -0.76474376D+04 29.8773551 0.0038916 567.852 0.004 EM

134.144

5 -0.76231000D+04 24.3376634 0.0031825 540.891 0.006 EM

161.103

6 -0.76028425D+04 20.2574630 0.0026574 515.206 0.010 EM

186.784

7 -0.75856850D+04 17.1575302 0.0022567 491.051 0.020 EM

210.929

8 -0.75713315D+04 14.3534533 0.0018922 468.777 0.047 EM

233.176

9 -0.75597084D+04 11.6231035 0.0015351 448.572 0.115 EM

253.314

10 -0.75504568D+04 9.2516243 0.0012238 430.386 0.258 EM

271.356

11 -0.75430677D+04 7.3890665 0.0009786 414.147 0.477 EM

287.376

12 -0.75372223D+04 5.8454433 0.0007749 399.878 0.702 EM

301.421

13 -0.75327596D+04 4.4627067 0.0005921 387.639 0.881 EM

313.480

14 -0.75295036D+04 3.2559886 0.0004322 377.422 1.021 EM

323.557

15 -0.75272399D+04 2.2636920 0.0003006 369.107 1.140 EM

331.754

16 -0.75257350D+04 1.5048572 0.0001999 362.477 1.249 EM

338.274

17 -0.75247695D+04 0.9655142 0.0001283 357.271 1.356 EM

343.374

TECHNICAL 8 OUTPUT FOR STARTING VALUE SET 48

ITER LOGLIKELIHOOD ABS CHANGE REL CHANGE CLASS COUNTS ALGORITHM

1 -0.25721176D+05 0.0000000 0.0000000 10.122 617.613 EM

74.265

2 -0.77471215D+04 ************ 0.6988038 17.466 588.602 EM

95.931

3 -0.76401889D+04 106.9325939 0.0138029 32.115 544.051 EM

125.834

4 -0.75860694D+04 54.1194633 0.0070835 48.025 501.972 EM

152.002

5 -0.75560797D+04 29.9897267 0.0039533 61.807 465.741 EM

174.452

6 -0.75368844D+04 19.1953343 0.0025404 73.655 435.000 EM

193.345

7 -0.75233028D+04 13.5815999 0.0018020 84.619 408.632 EM

208.748

8 -0.75125309D+04 10.7718938 0.0014318 95.870 385.548 EM

220.582

9 -0.75022215D+04 10.3093342 0.0013723 108.515 364.909 EM

228.577

10 -0.74904052D+04 11.8163829 0.0015751 122.829 346.814 EM

232.357

11 -0.74771675D+04 13.2376311 0.0017673 137.818 332.291 EM

231.891

12 -0.74646324D+04 12.5350773 0.0016764 151.689 322.384 EM

227.926

13 -0.74552254D+04 9.4070042 0.0012602 163.241 316.850 EM

221.908

14 -0.74491111D+04 6.1143253 0.0008201 172.606 314.089 EM

215.305

15 -0.74450535D+04 4.0575789 0.0005447 180.299 312.759 EM

208.942

16 -0.74421956D+04 2.8579012 0.0003839 186.723 312.186 EM

203.091

17 -0.74400892D+04 2.1064520 0.0002830 192.152 312.074 EM

197.774

18 -0.74384792D+04 1.6099575 0.0002164 196.787 312.280 EM

192.933

19 -0.74372090D+04 1.2702038 0.0001708 200.782 312.720 EM

188.498

20 -0.74361795D+04 1.0295023 0.0001384 204.258 313.333 EM

184.409

21 -0.74353269D+04 0.8526401 0.0001147 207.314 314.070 EM

180.616

TECHNICAL 8 OUTPUT FOR STARTING VALUE SET 49

ITER LOGLIKELIHOOD ABS CHANGE REL CHANGE CLASS COUNTS ALGORITHM

1 -0.26059805D+05 0.0000000 0.0000000 258.689 280.690 EM

162.621

2 -0.76331556D+04 ************ 0.7070908 238.680 279.624 EM

183.696

3 -0.75400028D+04 93.1527828 0.0122037 221.601 275.291 EM

205.108

4 -0.75041723D+04 35.8304728 0.0047521 212.797 269.015 EM

220.187

5 -0.74874038D+04 16.7685557 0.0022346 211.699 260.828 EM

229.474

6 -0.74765090D+04 10.8947708 0.0014551 216.543 250.877 EM

234.580

7 -0.74674123D+04 9.0966835 0.0012167 225.698 239.621 EM

236.681

8 -0.74593145D+04 8.0978315 0.0010844 237.596 227.831 EM

236.574

9 -0.74522556D+04 7.0588695 0.0009463 250.660 216.337 EM

235.002

10 -0.74463479D+04 5.9077830 0.0007928 263.566 205.765 EM

232.669

11 -0.74417023D+04 4.6455465 0.0006239 275.386 196.447 EM

230.166

12 -0.74383110D+04 3.3913507 0.0004557 285.612 188.467 EM

227.921

13 -0.74359929D+04 2.3180287 0.0003116 294.082 181.743 EM

226.175

14 -0.74344700D+04 1.5228768 0.0002048 300.889 176.107 EM

225.004

15 -0.74334716D+04 0.9984165 0.0001343 306.258 171.363 EM

224.379

TECHNICAL 8 OUTPUT FOR STARTING VALUE SET 50

ITER LOGLIKELIHOOD ABS CHANGE REL CHANGE CLASS COUNTS ALGORITHM

1 -0.13442655D+05 0.0000000 0.0000000 3.297 493.894 EM

204.809

2 -0.77512567D+04 5691.3984384 0.4233835 5.703 491.984 EM

204.314

3 -0.77266833D+04 24.5733910 0.0031702 7.300 483.031 EM

211.669

4 -0.76963432D+04 30.3401540 0.0039267 8.004 465.029 EM

228.967

5 -0.76345828D+04 61.7603632 0.0080246 8.471 439.583 EM

253.947

6 -0.75671637D+04 67.4190604 0.0088307 8.382 414.900 EM

278.719

7 -0.75335105D+04 33.6532067 0.0044473 7.880 395.855 EM

298.265

8 -0.75217363D+04 11.7742270 0.0015629 7.370 381.054 EM

313.576

9 -0.75161018D+04 5.6345581 0.0007491 6.969 369.415 EM

325.616

10 -0.75129232D+04 3.1785643 0.0004229 6.664 360.385 EM

334.951

11 -0.75111143D+04 1.8089239 0.0002408 6.432 353.488 EM

342.080

12 -0.75100913D+04 1.0229738 0.0001362 6.254 348.276 EM

347.469

13 -0.75095128D+04 0.5785307 0.0000770 6.119 344.360 EM

351.521

TECHNICAL 8 OUTPUT FOR STARTING VALUE SET 51

ITER LOGLIKELIHOOD ABS CHANGE REL CHANGE CLASS COUNTS ALGORITHM

1 -0.17351393D+05 0.0000000 0.0000000 36.578 652.882 EM

12.540

2 -0.77895173D+04 9561.8756169 0.5510725 29.634 640.085 EM

32.280

3 -0.77325763D+04 56.9410134 0.0073100 29.463 615.020 EM

57.516

4 -0.76858530D+04 46.7232802 0.0060424 31.892 585.329 EM

84.779

5 -0.76474311D+04 38.4219842 0.0049991 36.055 554.314 EM

111.631

6 -0.76157802D+04 31.6508381 0.0041388 41.732 523.082 EM

137.186

7 -0.75883663D+04 27.4139202 0.0035996 48.792 492.601 EM

160.607

8 -0.75642493D+04 24.1169697 0.0031782 57.112 463.875 EM

181.012

9 -0.75435153D+04 20.7340317 0.0027411 66.603 437.747 EM

197.650

10 -0.75267201D+04 16.7951669 0.0022264 77.063 414.775 EM

210.162

11 -0.75141018D+04 12.6183593 0.0016765 88.050 395.262 EM

218.689

12 -0.75051208D+04 8.9809699 0.0011952 99.115 379.167 EM

223.718

13 -0.74987644D+04 6.3563555 0.0008469 110.017 366.094 EM

225.889

14 -0.74939668D+04 4.7976882 0.0006398 120.732 355.420 EM

225.848

15 -0.74898417D+04 4.1250376 0.0005504 131.396 346.465 EM

224.139

16 -0.74857483D+04 4.0934067 0.0005465 142.255 338.583 EM

221.163

17 -0.74812879D+04 4.4604226 0.0005959 153.616 331.212 EM

217.172

18 -0.74763188D+04 4.9691225 0.0006642 165.772 323.913 EM

212.315

19 -0.74709700D+04 5.3487876 0.0007154 178.884 316.421 EM

206.695

20 -0.74655471D+04 5.4228875 0.0007259 192.882 308.676 EM

200.442

21 -0.74603165D+04 5.2305804 0.0007006 207.483 300.775 EM

193.742

22 -0.74553753D+04 4.9412443 0.0006623 222.326 292.862 EM

186.811

23 -0.74507136D+04 4.6617153 0.0006253 237.072 285.070 EM

179.858

24 -0.74463392D+04 4.3743551 0.0005871 251.392 277.527 EM

173.082

25 -0.74423448D+04 3.9944335 0.0005364 264.925 270.391 EM

166.684

26 -0.74388849D+04 3.4598552 0.0004649 277.301 263.849 EM

160.850

27 -0.74360897D+04 2.7952074 0.0003758 288.214 258.073 EM

155.713

28 -0.74339880D+04 2.1017294 0.0002826 297.501 253.175 EM

151.324

29 -0.74325009D+04 1.4870413 0.0002000 305.167 249.186 EM

147.647

30 -0.74314926D+04 1.0083451 0.0001357 311.344 246.060 EM

144.595

31 -0.74308242D+04 0.6683781 0.0000899 316.242 243.702 EM

142.056

TECHNICAL 8 OUTPUT FOR STARTING VALUE SET 52

ITER LOGLIKELIHOOD ABS CHANGE REL CHANGE CLASS COUNTS ALGORITHM

1 -0.20227989D+05 0.0000000 0.0000000 116.182 48.398 EM

537.420

2 -0.75861615D+04 ************ 0.6249671 139.384 62.398 EM

500.219

3 -0.75377229D+04 48.4386618 0.0063851 157.983 74.396 EM

469.622

4 -0.75160303D+04 21.6925429 0.0028779 172.151 84.285 EM

445.564

5 -0.75034633D+04 12.5670692 0.0016720 182.715 92.398 EM

426.887

6 -0.74961094D+04 7.3538731 0.0009801 190.374 99.062 EM

412.565

7 -0.74918051D+04 4.3043109 0.0005742 195.756 104.610 EM

401.635

8 -0.74892350D+04 2.5700842 0.0003431 199.399 109.331 EM

393.270

9 -0.74876468D+04 1.5881552 0.0002121 201.731 113.449 EM

386.820

10 -0.74866270D+04 1.0198160 0.0001362 203.078 117.121 EM

381.801

11 -0.74859473D+04 0.6797730 0.0000908 203.682 120.457 EM

377.861

TECHNICAL 8 OUTPUT FOR STARTING VALUE SET 53

ITER LOGLIKELIHOOD ABS CHANGE REL CHANGE CLASS COUNTS ALGORITHM

1 -0.19740126D+05 0.0000000 0.0000000 242.497 76.572 EM

382.931

2 -0.76457781D+04 ************ 0.6126783 258.436 61.848 EM

381.716

3 -0.75725528D+04 73.2253504 0.0095772 275.941 57.040 EM

369.020

4 -0.75381993D+04 34.3534664 0.0045366 291.331 55.708 EM

354.960

5 -0.75235510D+04 14.6482895 0.0019432 303.309 56.555 EM

342.136

6 -0.75147292D+04 8.8217964 0.0011726 312.168 58.498 EM

331.334

7 -0.75081291D+04 6.6001364 0.0008783 318.541 60.588 EM

322.871

8 -0.75028958D+04 5.2333051 0.0006970 322.872 62.874 EM

316.254

9 -0.74965460D+04 6.3498171 0.0008463 325.241 66.086 EM

310.673

10 -0.74859414D+04 10.6045859 0.0014146 325.221 70.441 EM

306.338

11 -0.74709759D+04 14.9654796 0.0019991 322.147 74.955 EM

304.898

12 -0.74566746D+04 14.3012551 0.0019142 316.146 78.295 EM

307.558

13 -0.74473363D+04 9.3383566 0.0012523 308.727 80.822 EM

312.450

14 -0.74418497D+04 5.4865946 0.0007367 301.254 83.044 EM

317.703

15 -0.74385698D+04 3.2799161 0.0004407 294.357 85.147 EM

322.497

16 -0.74364273D+04 2.1424465 0.0002880 288.210 87.205 EM

326.585

17 -0.74348606D+04 1.5666843 0.0002107 282.780 89.221 EM

329.999

18 -0.74336203D+04 1.2403916 0.0001668 277.981 91.170 EM

332.848

19 -0.74326001D+04 1.0201711 0.0001372 273.737 93.028 EM

335.235

20 -0.74317548D+04 0.8452740 0.0001137 269.995 94.774 EM

337.231

TECHNICAL 8 OUTPUT FOR STARTING VALUE SET 54

ITER LOGLIKELIHOOD ABS CHANGE REL CHANGE CLASS COUNTS ALGORITHM

1 -0.17220796D+05 0.0000000 0.0000000 21.392 61.985 EM

618.623

2 -0.77812132D+04 9439.5830114 0.5481502 35.809 53.676 EM

612.515

3 -0.77355433D+04 45.6698997 0.0058693 46.186 59.400 EM

596.415

4 -0.77036065D+04 31.9367775 0.0041286 53.185 74.027 EM

574.788

5 -0.76696371D+04 33.9693760 0.0044095 57.045 96.788 EM

548.167

6 -0.76294422D+04 40.1949089 0.0052408 59.373 125.838 EM

516.789

7 -0.75791057D+04 50.3364926 0.0065977 62.697 156.441 EM

482.862

8 -0.75357667D+04 43.3389852 0.0057182 67.960 180.655 EM

453.385

9 -0.75124821D+04 23.2846480 0.0030899 73.695 198.250 EM

430.055

10 -0.75000053D+04 12.4767378 0.0016608 78.777 211.149 EM

412.074

11 -0.74928710D+04 7.1343411 0.0009512 82.901 220.742 EM

398.357

12 -0.74886567D+04 4.2142514 0.0005624 86.137 228.007 EM

387.856

13 -0.74860759D+04 2.5808496 0.0003446 88.647 233.625 EM

379.728

14 -0.74844399D+04 1.6360298 0.0002185 90.589 238.056 EM

373.355

15 -0.74833765D+04 1.0634156 0.0001421 92.090 241.611 EM

368.299

16 -0.74826736D+04 0.7028722 0.0000939 93.245 244.506 EM

364.249

TECHNICAL 8 OUTPUT FOR STARTING VALUE SET 55

ITER LOGLIKELIHOOD ABS CHANGE REL CHANGE CLASS COUNTS ALGORITHM

1 -0.18406797D+05 0.0000000 0.0000000 107.738 95.946 EM

498.316

2 -0.76523266D+04 ************ 0.5842663 132.693 99.999 EM

469.308

3 -0.75639483D+04 88.3782509 0.0115492 148.795 109.698 EM

443.507

4 -0.75370152D+04 26.9331220 0.0035607 157.588 121.116 EM

423.296

5 -0.75192123D+04 17.8028674 0.0023621 162.325 131.886 EM

407.789

6 -0.75041122D+04 15.1001629 0.0020082 164.888 141.002 EM

396.110

7 -0.74934588D+04 10.6533423 0.0014197 166.448 148.170 EM

387.382

8 -0.74877958D+04 5.6629926 0.0007557 167.482 153.678 EM

380.840

9 -0.74852646D+04 2.5312688 0.0003381 168.127 157.990 EM

375.884

10 -0.74841450D+04 1.1195307 0.0001496 168.439 161.476 EM

372.086

11 -0.74836037D+04 0.5413500 0.0000723 168.469 164.381 EM

369.150

TECHNICAL 8 OUTPUT FOR STARTING VALUE SET 56

ITER LOGLIKELIHOOD ABS CHANGE REL CHANGE CLASS COUNTS ALGORITHM

1 -0.22991455D+05 0.0000000 0.0000000 349.205 326.931 EM

25.864

2 -0.77136458D+04 ************ 0.6644994 346.833 309.589 EM

45.578

3 -0.75722942D+04 141.3515686 0.0183249 337.895 303.197 EM

60.908

4 -0.74925778D+04 79.7164565 0.0105274 327.343 305.257 EM

69.399

5 -0.74616943D+04 30.8834950 0.0041219 318.064 310.029 EM

73.908

6 -0.74506492D+04 11.0450678 0.0014802 310.078 314.669 EM

77.253

7 -0.74442759D+04 6.3733230 0.0008554 302.867 318.907 EM

80.226

8 -0.74401122D+04 4.1637160 0.0005593 296.236 322.869 EM

82.894

9 -0.74374198D+04 2.6923462 0.0003619 290.192 326.483 EM

85.325

10 -0.74355667D+04 1.8531073 0.0002492 284.738 329.672 EM

87.590

11 -0.74341696D+04 1.3971520 0.0001879 279.843 332.435 EM

89.723

12 -0.74330473D+04 1.1222817 0.0001510 275.463 334.810 EM

91.727

13 -0.74321223D+04 0.9249565 0.0001244 271.565 336.836 EM

93.599

TECHNICAL 8 OUTPUT FOR STARTING VALUE SET 57

ITER LOGLIKELIHOOD ABS CHANGE REL CHANGE CLASS COUNTS ALGORITHM

1 -0.19743731D+05 0.0000000 0.0000000 21.908 54.206 EM

625.887

2 -0.77062691D+04 ************ 0.6096853 39.478 78.421 EM

584.101

3 -0.76290467D+04 77.2223433 0.0100207 55.817 105.883 EM

540.300

4 -0.75769840D+04 52.0626911 0.0068243 69.341 130.961 EM

501.698

5 -0.75415522D+04 35.4318549 0.0046762 80.541 151.859 EM

469.600

6 -0.75185094D+04 23.0427615 0.0030554 89.678 168.648 EM

443.674

7 -0.75040640D+04 14.4454339 0.0019213 96.950 181.787 EM

423.263

8 -0.74953312D+04 8.7327912 0.0011637 102.581 191.889 EM

407.531

9 -0.74901555D+04 5.1756859 0.0006905 106.850 199.634 EM

395.516

10 -0.74870615D+04 3.0940400 0.0004131 110.048 205.636 EM

386.316

11 -0.74851609D+04 1.9005216 0.0002538 112.426 210.369 EM

379.206

12 -0.74839601D+04 1.2008904 0.0001604 114.177 214.169 EM

373.654

13 -0.74831850D+04 0.7750162 0.0001036 115.449 217.276 EM

369.275

TECHNICAL 8 OUTPUT FOR STARTING VALUE SET 58

ITER LOGLIKELIHOOD ABS CHANGE REL CHANGE CLASS COUNTS ALGORITHM

1 -0.15611172D+05 0.0000000 0.0000000 101.661 18.666 EM

581.673

2 -0.78525861D+04 7758.5860488 0.4969893 100.097 18.304 EM

583.599

3 -0.77709102D+04 81.6759031 0.0104011 112.811 26.863 EM

562.325

4 -0.76831456D+04 87.7645491 0.0112940 129.308 38.943 EM

533.749

5 -0.76355012D+04 47.6443769 0.0062012 145.462 45.937 EM

510.601

6 -0.76119538D+04 23.5474543 0.0030839 162.549 49.492 EM

489.959

7 -0.75930092D+04 18.9445967 0.0024888 181.410 51.123 EM

469.467

8 -0.75723766D+04 20.6326432 0.0027173 202.684 51.261 EM

448.055

9 -0.75483863D+04 23.9902580 0.0031681 224.816 50.037 EM

427.147

10 -0.75273440D+04 21.0422571 0.0027876 244.575 47.882 EM

409.542

11 -0.75132079D+04 14.1361394 0.0018780 260.469 45.845 EM

395.686

12 -0.75017196D+04 11.4882840 0.0015291 272.308 45.969 EM

383.723

13 -0.74849653D+04 16.7542809 0.0022334 279.912 51.286 EM

370.802

14 -0.74608569D+04 24.1084364 0.0032209 282.866 60.608 EM

358.525

15 -0.74441887D+04 16.6681562 0.0022341 282.106 68.985 EM

350.909

16 -0.74375055D+04 6.6832605 0.0008978 279.576 75.123 EM

347.302

17 -0.74348897D+04 2.6157603 0.0003517 276.490 79.821 EM

345.689

18 -0.74333981D+04 1.4916465 0.0002006 273.322 83.649 EM

345.030

19 -0.74323257D+04 1.0723351 0.0001443 270.275 86.874 EM

344.852

20 -0.74315065D+04 0.8192036 0.0001102 267.457 89.642 EM

344.901

TECHNICAL 8 OUTPUT FOR STARTING VALUE SET 59

ITER LOGLIKELIHOOD ABS CHANGE REL CHANGE CLASS COUNTS ALGORITHM

1 -0.20404326D+05 0.0000000 0.0000000 398.431 242.029 EM

61.541

2 -0.75532688D+04 ************ 0.6298202 382.311 263.373 EM

56.316

3 -0.75204527D+04 32.8161604 0.0043446 367.724 277.855 EM

56.420

4 -0.75111044D+04 9.3483136 0.0012431 356.165 288.222 EM

57.613

5 -0.75065200D+04 4.5843821 0.0006103 347.548 295.621 EM

58.831

6 -0.75041399D+04 2.3801277 0.0003171 341.348 300.804 EM

59.848

7 -0.75028928D+04 1.2470947 0.0001662 336.948 304.389 EM

60.663

8 -0.75022240D+04 0.6687709 0.0000891 333.825 306.853 EM

61.321

TECHNICAL 8 OUTPUT FOR STARTING VALUE SET 60

ITER LOGLIKELIHOOD ABS CHANGE REL CHANGE CLASS COUNTS ALGORITHM

1 -0.17720907D+05 0.0000000 0.0000000 152.937 18.358 EM

530.705

2 -0.78285685D+04 9892.3389281 0.5582298 152.112 15.743 EM

534.145

3 -0.77222091D+04 106.3594075 0.0135861 157.505 37.273 EM

507.222

4 -0.76147568D+04 107.4523093 0.0139147 164.730 60.636 EM

476.635

5 -0.75629683D+04 51.7884890 0.0068011 176.394 78.057 EM

447.549

6 -0.75319133D+04 31.0549945 0.0041062 190.822 90.267 EM

420.911

7 -0.75112411D+04 20.6721883 0.0027446 206.421 98.057 EM

397.522

8 -0.74964111D+04 14.8300789 0.0019744 221.813 102.659 EM

377.528

9 -0.74847037D+04 11.7073989 0.0015617 236.321 105.011 EM

360.668

10 -0.74746353D+04 10.0683291 0.0013452 249.866 105.794 EM

346.340

11 -0.74654878D+04 9.1475744 0.0012238 262.550 105.602 EM

333.849

12 -0.74571063D+04 8.3814592 0.0011227 274.445 104.917 EM

322.638

13 -0.74497287D+04 7.3776071 0.0009893 285.550 104.052 EM

312.398

14 -0.74437752D+04 5.9534718 0.0007992 295.710 103.220 EM

303.070

15 -0.74394532D+04 4.3220534 0.0005806 304.648 102.605 EM

294.747

16 -0.74365311D+04 2.9220662 0.0003928 312.189 102.319 EM

287.492

17 -0.74345685D+04 1.9625692 0.0002639 318.375 102.366 EM

281.259

18 -0.74331896D+04 1.3789452 0.0001855 323.387 102.684 EM

275.929

19 -0.74321604D+04 1.0291520 0.0001385 327.441 103.192 EM

271.368

20 -0.74313575D+04 0.8029325 0.0001080 330.727 103.811 EM

267.462

TECHNICAL 8 OUTPUT FOR STARTING VALUE SET 61

ITER LOGLIKELIHOOD ABS CHANGE REL CHANGE CLASS COUNTS ALGORITHM

1 -0.13387411D+05 0.0000000 0.0000000 34.793 31.974 EM

635.232

2 -0.77663810D+04 5621.0297071 0.4198743 60.642 20.889 EM

620.469

3 -0.77213733D+04 45.0077001 0.0057952 86.339 18.610 EM

597.051

4 -0.76942258D+04 27.1474737 0.0035159 111.880 19.038 EM

571.082

5 -0.76694850D+04 24.7408485 0.0032155 137.701 20.905 EM

543.394

6 -0.76436207D+04 25.8642642 0.0033724 164.194 23.845 EM

513.962

7 -0.76138284D+04 29.7923442 0.0038977 191.958 27.492 EM

482.551

8 -0.75787514D+04 35.0770348 0.0046070 219.288 31.440 EM

451.273

9 -0.75467147D+04 32.0366498 0.0042272 242.495 35.421 EM

424.085

10 -0.75258085D+04 20.9062515 0.0027702 260.405 39.248 EM

402.347

11 -0.75141047D+04 11.7037675 0.0015552 273.612 42.756 EM

385.632

12 -0.75079021D+04 6.2026248 0.0008255 283.131 46.019 EM

372.850

13 -0.75044812D+04 3.4208638 0.0004556 289.957 49.233 EM

362.810

14 -0.75023350D+04 2.1461715 0.0002860 294.858 52.565 EM

354.578

15 -0.75007787D+04 1.5563495 0.0002074 298.364 56.120 EM

347.516

16 -0.74995066D+04 1.2720745 0.0001696 300.836 59.965 EM

341.200

17 -0.74983672D+04 1.1394503 0.0001519 302.518 64.142 EM

335.340

18 -0.74972738D+04 1.0933937 0.0001458 303.585 68.687 EM

329.728

19 -0.74961696D+04 1.1041300 0.0001473 304.159 73.637 EM

324.204

20 -0.74950115D+04 1.1581660 0.0001545 304.328 79.032 EM

318.640

21 -0.74937597D+04 1.2517843 0.0001670 304.151 84.924 EM

312.925

22 -0.74923717D+04 1.3880013 0.0001852 303.666 91.375 EM

306.958

23 -0.74907964D+04 1.5752439 0.0002102 302.893 98.467 EM

300.640

24 -0.74889707D+04 1.8257419 0.0002437 301.828 106.297 EM

293.874

25 -0.74868190D+04 2.1517012 0.0002873 300.452 114.980 EM

286.568

26 -0.74842629D+04 2.5561197 0.0003414 298.716 124.635 EM

278.649

27 -0.74812447D+04 3.0181951 0.0004033 296.552 135.358 EM

270.091

28 -0.74777593D+04 3.4854009 0.0004659 293.881 147.173 EM

260.946

29 -0.74738681D+04 3.8911314 0.0005204 290.648 159.992 EM

251.360

30 -0.74696785D+04 4.1896264 0.0005606 286.841 173.620 EM

241.539

31 -0.74653058D+04 4.3726807 0.0005854 282.485 187.816 EM

231.699

32 -0.74608458D+04 4.4600199 0.0005974 277.633 202.347 EM

222.020

33 -0.74563685D+04 4.4773651 0.0006001 272.361 217.006 EM

212.632

34 -0.74519365D+04 4.4319449 0.0005944 266.778 231.587 EM

203.635

35 -0.74407619D+04 11.1745732 0.0014996 247.619 293.286 FS

161.095

36 -0.74381074D+04 2.6545139 0.0003568 240.981 304.519 FS

156.500

37 -0.74322616D+04 5.8458660 0.0007859 239.305 309.616 EM

153.079

38 -0.74313945D+04 0.8670023 0.0001167 237.808 314.083 EM

150.109

TECHNICAL 8 OUTPUT FOR STARTING VALUE SET 62

ITER LOGLIKELIHOOD ABS CHANGE REL CHANGE CLASS COUNTS ALGORITHM

1 -0.13435849D+05 0.0000000 0.0000000 65.410 635.244 EM

1.346

2 -0.77888315D+04 5647.0172053 0.4202948 74.740 620.197 EM

7.062

3 -0.77353442D+04 53.4873138 0.0068672 99.587 587.995 EM

14.418

4 -0.76683658D+04 66.9783627 0.0086587 138.355 542.127 EM

21.518

5 -0.75906306D+04 77.7352584 0.0101371 177.973 494.745 EM

29.282

6 -0.75335209D+04 57.1096354 0.0075237 208.936 452.689 EM

40.374

7 -0.74867494D+04 46.7715222 0.0062085 230.900 416.052 EM

55.047

8 -0.74539136D+04 32.8358098 0.0043859 244.531 389.149 EM

68.320

9 -0.74389105D+04 15.0030470 0.0020128 252.006 372.415 EM

77.579

10 -0.74335501D+04 5.3604604 0.0007206 255.746 362.352 EM

83.901

11 -0.74314906D+04 2.0594588 0.0002770 257.346 356.212 EM

88.442

12 -0.74305614D+04 0.9292045 0.0001250 257.745 352.401 EM

91.854

TECHNICAL 8 OUTPUT FOR STARTING VALUE SET 63

ITER LOGLIKELIHOOD ABS CHANGE REL CHANGE CLASS COUNTS ALGORITHM

1 -0.23531479D+05 0.0000000 0.0000000 181.591 122.742 EM

397.667

2 -0.77876302D+04 ************ 0.6690548 159.143 148.844 EM

394.013

3 -0.76351141D+04 152.5160956 0.0195844 141.092 181.503 EM

379.405

4 -0.75668048D+04 68.3093380 0.0089467 132.016 209.000 EM

360.984

5 -0.75272632D+04 39.5415334 0.0052257 127.984 230.968 EM

343.047

6 -0.75059057D+04 21.3575434 0.0028374 128.046 246.722 EM

327.232

7 -0.74956325D+04 10.2732187 0.0013687 131.382 257.140 EM

313.478

8 -0.74899196D+04 5.7128994 0.0007622 137.214 263.703 EM

301.084

9 -0.74855783D+04 4.3412630 0.0005796 145.016 267.654 EM

289.329

10 -0.74813844D+04 4.1938812 0.0005603 154.503 269.746 EM

277.752

11 -0.74769363D+04 4.4481784 0.0005946 165.501 270.353 EM

266.146

12 -0.74721768D+04 4.7594024 0.0006365 177.823 269.653 EM

254.524

13 -0.74672065D+04 4.9703109 0.0006652 191.193 267.774 EM

243.034

14 -0.74621591D+04 5.0474813 0.0006760 205.276 264.858 EM

231.866

15 -0.74571466D+04 5.0124224 0.0006717 219.733 261.085 EM

221.181

16 -0.74522712D+04 4.8754158 0.0006538 234.222 256.678 EM

211.101

17 -0.74476607D+04 4.6105492 0.0006187 248.361 251.912 EM

201.726

18 -0.74434886D+04 4.1720380 0.0005602 261.724 247.116 EM

193.160

19 -0.74399418D+04 3.5468212 0.0004765 273.886 242.621 EM

185.493

20 -0.74371419D+04 2.7999590 0.0003763 284.522 238.701 EM

178.777

21 -0.74350865D+04 2.0553881 0.0002764 293.484 235.520 EM

172.997

22 -0.74336608D+04 1.4256414 0.0001917 300.806 233.121 EM

168.073

23 -0.74327004D+04 0.9603729 0.0001292 306.660 231.453 EM

163.886

TECHNICAL 8 OUTPUT FOR STARTING VALUE SET 64

ITER LOGLIKELIHOOD ABS CHANGE REL CHANGE CLASS COUNTS ALGORITHM

1 -0.21145371D+05 0.0000000 0.0000000 158.042 504.745 EM

39.213

2 -0.76680063D+04 ************ 0.6373671 166.562 484.302 EM

51.136

3 -0.75763849D+04 91.6214157 0.0119485 174.724 458.809 EM

68.467

4 -0.75306190D+04 45.7659194 0.0060406 183.538 435.609 EM

82.853

5 -0.74936771D+04 36.9418303 0.0049056 195.929 411.706 EM

94.366

6 -0.74600467D+04 33.6304106 0.0044878 209.659 389.282 EM

103.059

7 -0.74404489D+04 19.5978534 0.0026270 220.667 372.242 EM

109.090

8 -0.74331800D+04 7.2688682 0.0009769 228.414 360.556 EM

113.030

9 -0.74306228D+04 2.5571528 0.0003440 233.674 352.827 EM

115.499

10 -0.74296505D+04 0.9723814 0.0001309 237.211 347.806 EM

116.982

TECHNICAL 8 OUTPUT FOR STARTING VALUE SET 65

ITER LOGLIKELIHOOD ABS CHANGE REL CHANGE CLASS COUNTS ALGORITHM

1 -0.20239263D+05 0.0000000 0.0000000 367.588 264.619 EM

69.792

2 -0.75308120D+04 ************ 0.6279108 353.646 270.645 EM

77.709

3 -0.74882896D+04 42.5224028 0.0056465 341.539 277.597 EM

82.864

4 -0.74721142D+04 16.1753476 0.0021601 331.137 284.904 EM

85.959

5 -0.74611960D+04 10.9182697 0.0014612 321.781 292.361 EM

87.858

6 -0.74524929D+04 8.7030805 0.0011664 313.068 299.712 EM

89.220

7 -0.74456065D+04 6.8863631 0.0009240 304.883 306.738 EM

90.379

8 -0.74406671D+04 4.9394720 0.0006634 297.306 313.237 EM

91.457

9 -0.74374380D+04 3.2290092 0.0004340 290.478 318.968 EM

92.554

10 -0.74353324D+04 2.1056918 0.0002831 284.448 323.818 EM

93.733

11 -0.74338592D+04 1.4731306 0.0001981 279.169 327.840 EM

94.991

12 -0.74327429D+04 1.1162723 0.0001502 274.555 331.154 EM

96.291

13 -0.74318533D+04 0.8896304 0.0001197 270.529 333.882 EM

97.589

TECHNICAL 8 OUTPUT FOR STARTING VALUE SET 66

ITER LOGLIKELIHOOD ABS CHANGE REL CHANGE CLASS COUNTS ALGORITHM

1 -0.18739142D+05 0.0000000 0.0000000 21.945 306.883 EM

373.172

2 -0.77094554D+04 ************ 0.5885908 31.811 308.586 EM

361.602

3 -0.75722158D+04 137.2396572 0.0178015 37.383 311.063 EM

353.554

4 -0.75052191D+04 66.9967311 0.0088477 40.628 314.666 EM

346.706

5 -0.74886911D+04 16.5279526 0.0022022 43.446 318.993 EM

339.561

6 -0.74852884D+04 3.4027069 0.0004544 46.177 323.058 EM

332.765

7 -0.74839715D+04 1.3169077 0.0001759 48.755 326.392 EM

326.853

8 -0.74832107D+04 0.7607633 0.0001017 51.139 328.979 EM

321.882

TECHNICAL 8 OUTPUT FOR STARTING VALUE SET 67

ITER LOGLIKELIHOOD ABS CHANGE REL CHANGE CLASS COUNTS ALGORITHM

1 -0.22989973D+05 0.0000000 0.0000000 502.435 94.935 EM

104.630

2 -0.76164775D+04 ************ 0.6687044 481.065 103.643 EM

117.292

3 -0.75397709D+04 76.7066005 0.0100711 454.581 116.466 EM

130.953

4 -0.75093558D+04 30.4150766 0.0040340 431.146 128.556 EM

142.298

5 -0.74906624D+04 18.6934384 0.0024894 408.740 141.529 EM

151.731

6 -0.74735701D+04 17.0923114 0.0022818 386.783 156.064 EM

159.153

7 -0.74571295D+04 16.4405307 0.0021998 367.042 170.807 EM

164.151

8 -0.74451308D+04 11.9987032 0.0016090 351.777 183.511 EM

166.713

9 -0.74387994D+04 6.3314347 0.0008504 341.151 193.430 EM

167.419

10 -0.74357694D+04 3.0300356 0.0004073 334.098 200.967 EM

166.936

11 -0.74342056D+04 1.5637903 0.0002103 329.541 206.725 EM

165.734

12 -0.74332901D+04 0.9154438 0.0001231 326.689 211.200 EM

164.111

TECHNICAL 8 OUTPUT FOR STARTING VALUE SET 68

ITER LOGLIKELIHOOD ABS CHANGE REL CHANGE CLASS COUNTS ALGORITHM

1 -0.22963402D+05 0.0000000 0.0000000 255.192 338.863 EM

107.944

2 -0.77749807D+04 ************ 0.6614186 233.148 331.605 EM

137.247

3 -0.76150428D+04 159.9378265 0.0205708 213.674 320.392 EM

167.934

4 -0.75472496D+04 67.7932415 0.0089025 211.533 305.189 EM

185.278

5 -0.75006947D+04 46.5548967 0.0061685 221.452 290.152 EM

190.396

6 -0.74674934D+04 33.2013172 0.0044264 237.130 278.008 EM

186.862

7 -0.74507558D+04 16.7375493 0.0022414 253.189 268.860 EM

179.951

8 -0.74430010D+04 7.7547915 0.0010408 267.593 261.597 EM

172.810

9 -0.74385574D+04 4.4436211 0.0005970 280.062 255.540 EM

166.398

10 -0.74356047D+04 2.9527284 0.0003969 290.632 250.485 EM

160.883

11 -0.74335942D+04 2.0104995 0.0002704 299.389 246.381 EM

156.230

12 -0.74322598D+04 1.3343965 0.0001795 306.479 243.175 EM

152.346

13 -0.74313971D+04 0.8627300 0.0001161 312.114 240.780 EM

149.106

TECHNICAL 8 OUTPUT FOR STARTING VALUE SET 69

ITER LOGLIKELIHOOD ABS CHANGE REL CHANGE CLASS COUNTS ALGORITHM

1 -0.15037124D+05 0.0000000 0.0000000 39.508 480.662 EM

181.830

2 -0.76367796D+04 7400.3446850 0.4921383 36.114 447.996 EM

217.890

3 -0.75734781D+04 63.3015019 0.0082890 37.859 416.903 EM

247.238

4 -0.75430696D+04 30.4084463 0.0040151 41.522 390.218 EM

270.260

5 -0.75205922D+04 22.4774580 0.0029799 46.345 366.941 EM

288.715

6 -0.75009460D+04 19.6461184 0.0026123 51.066 348.259 EM

302.676

7 -0.74891901D+04 11.7559179 0.0015673 54.542 335.228 EM

312.230

8 -0.74847228D+04 4.4673614 0.0005965 56.886 326.472 EM

318.642

9 -0.74830933D+04 1.6294767 0.0002177 58.695 320.187 EM

323.118

10 -0.74823616D+04 0.7316971 0.0000978 60.268 315.384 EM

326.349

TECHNICAL 8 OUTPUT FOR STARTING VALUE SET 70

ITER LOGLIKELIHOOD ABS CHANGE REL CHANGE CLASS COUNTS ALGORITHM

1 -0.26293505D+05 0.0000000 0.0000000 19.315 609.800 EM

72.885

2 -0.77438137D+04 ************ 0.7054857 35.004 587.673 EM

79.323

3 -0.76672050D+04 76.6086542 0.0098929 49.468 556.800 EM

95.732

4 -0.76013756D+04 65.8294568 0.0085858 61.429 527.622 EM

112.949

5 -0.75597289D+04 41.6466789 0.0054788 71.572 501.361 EM

129.066

6 -0.75364733D+04 23.2556365 0.0030763 81.127 476.965 EM

143.908

7 -0.75205332D+04 15.9400901 0.0021151 90.849 453.616 EM

157.535

8 -0.75074986D+04 13.0345589 0.0017332 100.990 431.230 EM

169.779

9 -0.74960643D+04 11.4343437 0.0015231 111.559 410.174 EM

180.267

10 -0.74857950D+04 10.2692510 0.0013700 122.610 390.798 EM

188.591

11 -0.74761308D+04 9.6642182 0.0012910 134.365 373.181 EM

194.455

12 -0.74663557D+04 9.7751556 0.0013075 146.872 357.398 EM

197.730

13 -0.74566747D+04 9.6809930 0.0012966 159.358 344.117 EM

198.525

14 -0.74487949D+04 7.8797791 0.0010567 170.593 334.100 EM

197.307

15 -0.74436372D+04 5.1576896 0.0006924 179.996 327.192 EM

194.812

16 -0.74404771D+04 3.1600658 0.0004245 187.655 322.670 EM

191.675

17 -0.74384325D+04 2.0446009 0.0002748 193.884 319.835 EM

188.281

18 -0.74369955D+04 1.4370569 0.0001932 198.997 318.170 EM

184.834

19 -0.74359134D+04 1.0820812 0.0001455 203.250 317.315 EM

181.436

20 -0.74350569D+04 0.8564873 0.0001152 206.838 317.023 EM

178.139

TECHNICAL 8 OUTPUT FOR STARTING VALUE SET 71

ITER LOGLIKELIHOOD ABS CHANGE REL CHANGE CLASS COUNTS ALGORITHM

1 -0.16881336D+05 0.0000000 0.0000000 28.097 419.144 EM

254.759

2 -0.75825660D+04 9298.7695983 0.5508314 26.241 404.435 EM

271.324

3 -0.75243000D+04 58.2659571 0.0076842 26.660 391.415 EM

283.925

4 -0.75129187D+04 11.3813084 0.0015126 27.906 381.573 EM

292.521

5 -0.75089211D+04 3.9976630 0.0005321 29.615 374.065 EM

298.319

6 -0.75068502D+04 2.0708916 0.0002758 31.675 368.079 EM

302.247

7 -0.75054716D+04 1.3785579 0.0001836 34.054 363.007 EM

304.939

8 -0.75043529D+04 1.1187603 0.0001491 36.732 358.464 EM

306.805

9 -0.75033483D+04 1.0046009 0.0001339 39.680 354.220 EM

308.100

10 -0.75024099D+04 0.9383234 0.0001251 42.876 350.145 EM

308.979

TECHNICAL 8 OUTPUT FOR STARTING VALUE SET 72

ITER LOGLIKELIHOOD ABS CHANGE REL CHANGE CLASS COUNTS ALGORITHM

1 -0.18488701D+05 0.0000000 0.0000000 257.721 384.015 EM

60.263

2 -0.75205373D+04 ************ 0.5932360 271.197 359.639 EM

71.164

3 -0.74699270D+04 50.6102722 0.0067296 278.306 345.569 EM

78.125

4 -0.74498862D+04 20.0408035 0.0026829 280.088 339.240 EM

82.672

5 -0.74396395D+04 10.2467086 0.0013754 278.563 337.312 EM

86.125

6 -0.74347674D+04 4.8721202 0.0006549 275.461 337.737 EM

88.802

7 -0.74326380D+04 2.1293948 0.0002864 271.958 339.017 EM

91.026

8 -0.74315386D+04 1.0993980 0.0001479 268.613 340.388 EM

92.999

9 -0.74308364D+04 0.7021962 0.0000945 265.626 341.576 EM

94.798

TECHNICAL 8 OUTPUT FOR STARTING VALUE SET 73

ITER LOGLIKELIHOOD ABS CHANGE REL CHANGE CLASS COUNTS ALGORITHM

1 -0.19171059D+05 0.0000000 0.0000000 490.545 211.436 EM

0.019

2 -0.76749439D+04 ************ 0.5996599 481.106 220.827 EM

0.067

3 -0.76130381D+04 61.9057966 0.0080660 467.588 234.302 EM

0.110

4 -0.75745189D+04 38.5192324 0.0050596 450.599 251.268 EM

0.132

5 -0.75553308D+04 19.1881098 0.0025332 432.907 268.943 EM

0.150

6 -0.75451613D+04 10.1694780 0.0013460 416.195 285.631 EM

0.173

7 -0.75383868D+04 6.7745245 0.0008979 401.194 300.602 EM

0.204

8 -0.75334600D+04 4.9267897 0.0006536 388.239 313.514 EM

0.247

9 -0.75299273D+04 3.5326677 0.0004689 377.425 324.270 EM

0.305

10 -0.75274907D+04 2.4366212 0.0003236 368.656 332.950 EM

0.393

11 -0.75257869D+04 1.7038555 0.0002264 361.724 339.690 EM

0.586

12 -0.75238380D+04 1.9488980 0.0002590 356.414 344.488 EM

1.098

13 -0.75213635D+04 2.4744820 0.0003289 352.425 347.807 EM

1.768

14 -0.75201353D+04 1.2281556 0.0001633 349.385 350.092 EM

2.523

15 -0.75193649D+04 0.7704035 0.0001024 347.057 351.654 EM

3.289

TECHNICAL 8 OUTPUT FOR STARTING VALUE SET 74

ITER LOGLIKELIHOOD ABS CHANGE REL CHANGE CLASS COUNTS ALGORITHM

1 -0.18658983D+05 0.0000000 0.0000000 507.357 7.756 EM

186.887

2 -0.76503500D+04 ************ 0.5899911 478.748 4.602 EM

218.650

3 -0.75898863D+04 60.4637103 0.0079034 450.940 4.657 EM

246.403

4 -0.75607937D+04 29.0926269 0.0038331 425.525 7.183 EM

269.292

5 -0.75303314D+04 30.4623025 0.0040290 398.738 15.470 EM

287.792

6 -0.75043335D+04 25.9978454 0.0034524 378.121 22.807 EM

301.072

7 -0.74949210D+04 9.4124902 0.0012543 363.086 28.377 EM

310.537

8 -0.74901667D+04 4.7542945 0.0006343 351.373 33.091 EM

317.537

9 -0.74873739D+04 2.7928264 0.0003729 342.083 37.183 EM

322.734

10 -0.74856366D+04 1.7373217 0.0002320 334.635 40.779 EM

326.586

11 -0.74844981D+04 1.1384746 0.0001521 328.588 43.973 EM

329.439

12 -0.74837147D+04 0.7833817 0.0001047 323.609 46.835 EM

331.556

TECHNICAL 8 OUTPUT FOR STARTING VALUE SET 75

ITER LOGLIKELIHOOD ABS CHANGE REL CHANGE CLASS COUNTS ALGORITHM

1 -0.23037792D+05 0.0000000 0.0000000 339.886 20.418 EM

341.696

2 -0.75349553D+04 ************ 0.6729307 338.050 17.210 EM

346.740

3 -0.75218056D+04 13.1497058 0.0017452 336.309 16.848 EM

348.843

4 -0.75189610D+04 2.8446697 0.0003782 334.902 17.348 EM

349.751

5 -0.75179081D+04 1.0528995 0.0001400 333.778 18.031 EM

350.191

6 -0.75174771D+04 0.4309535 0.0000573 332.875 18.693 EM

350.432

TECHNICAL 8 OUTPUT FOR STARTING VALUE SET 76

ITER LOGLIKELIHOOD ABS CHANGE REL CHANGE CLASS COUNTS ALGORITHM

1 -0.20783839D+05 0.0000000 0.0000000 146.997 127.346 EM

427.656

2 -0.75779710D+04 ************ 0.6353912 172.222 122.358 EM

407.419

3 -0.75222210D+04 55.7499640 0.0073568 182.735 128.479 EM

390.786

4 -0.75015162D+04 20.7048291 0.0027525 186.275 136.561 EM

379.164

5 -0.74923745D+04 9.1417262 0.0012187 186.967 143.558 EM

371.475

6 -0.74879976D+04 4.3768497 0.0005842 186.444 149.064 EM

366.491

7 -0.74858010D+04 2.1966539 0.0002934 185.339 153.363 EM

363.298

8 -0.74846559D+04 1.1450472 0.0001530 183.932 156.792 EM

361.276

9 -0.74840296D+04 0.6263029 0.0000837 182.378 159.608 EM

360.014

TECHNICAL 8 OUTPUT FOR STARTING VALUE SET 77

ITER LOGLIKELIHOOD ABS CHANGE REL CHANGE CLASS COUNTS ALGORITHM

1 -0.17781697D+05 0.0000000 0.0000000 67.449 90.180 EM

544.371

2 -0.76999698D+04 ************ 0.5669722 65.453 118.840 EM

517.707

3 -0.75987887D+04 101.1811211 0.0131405 79.484 146.337 EM

476.179

4 -0.75460085D+04 52.7802427 0.0069459 96.662 167.794 EM

437.544

5 -0.75162649D+04 29.7435457 0.0039416 115.363 183.266 EM

403.371

6 -0.74902380D+04 26.0268612 0.0034627 135.550 192.792 EM

373.658

7 -0.74672158D+04 23.0222366 0.0030736 154.259 196.611 EM

351.130

8 -0.74520768D+04 15.1389999 0.0020274 168.904 196.154 EM

336.941

9 -0.74444309D+04 7.6458869 0.0010260 179.802 193.552 EM

328.646

10 -0.74405048D+04 3.9261498 0.0005274 188.094 190.198 EM

323.708

11 -0.74382039D+04 2.3008437 0.0003092 194.597 186.675 EM

320.729

12 -0.74366904D+04 1.5134810 0.0002035 199.823 183.189 EM

318.988

13 -0.74356028D+04 1.0876926 0.0001463 204.112 179.812 EM

318.076

14 -0.74347673D+04 0.8354873 0.0001124 207.697 176.571 EM

317.732

TECHNICAL 8 OUTPUT FOR STARTING VALUE SET 78

ITER LOGLIKELIHOOD ABS CHANGE REL CHANGE CLASS COUNTS ALGORITHM

1 -0.23368092D+05 0.0000000 0.0000000 27.572 446.048 EM

228.380

2 -0.75737904D+04 ************ 0.6758918 38.087 421.987 EM

241.926

3 -0.75101934D+04 63.5969882 0.0083970 45.058 402.976 EM

253.966

4 -0.74972216D+04 12.9717553 0.0017272 50.162 388.275 EM

263.563

5 -0.74921601D+04 5.0614698 0.0006751 54.436 375.874 EM

271.690

6 -0.74892727D+04 2.8874335 0.0003854 58.136 365.141 EM

278.722

7 -0.74873464D+04 1.9263150 0.0002572 61.351 355.815 EM

284.834

8 -0.74859846D+04 1.3617761 0.0001819 64.132 347.719 EM

290.149

9 -0.74849950D+04 0.9895658 0.0001322 66.525 340.695 EM

294.780

TECHNICAL 8 OUTPUT FOR STARTING VALUE SET 79

ITER LOGLIKELIHOOD ABS CHANGE REL CHANGE CLASS COUNTS ALGORITHM

1 -0.27371787D+05 0.0000000 0.0000000 190.191 487.674 EM

24.135

2 -0.77504412D+04 ************ 0.7168456 186.962 471.424 EM

43.614

3 -0.76307606D+04 119.6805722 0.0154418 192.590 446.847 EM

62.564

4 -0.75697166D+04 61.0440699 0.0079997 194.803 429.418 EM

77.779

5 -0.75281588D+04 41.5577046 0.0054900 198.305 413.945 EM

89.750

6 -0.74893728D+04 38.7860306 0.0051521 206.930 395.827 EM

99.243

7 -0.74550824D+04 34.2904487 0.0045785 217.635 378.032 EM

106.333

8 -0.74375581D+04 17.5242868 0.0023506 226.266 364.569 EM

111.166

9 -0.74319179D+04 5.6401339 0.0007583 232.307 355.404 EM

114.290

10 -0.74300949D+04 1.8230761 0.0002453 236.384 349.388 EM

116.228

11 -0.74294288D+04 0.6660711 0.0000896 239.111 345.516 EM

117.373

TECHNICAL 8 OUTPUT FOR STARTING VALUE SET 80

ITER LOGLIKELIHOOD ABS CHANGE REL CHANGE CLASS COUNTS ALGORITHM

1 -0.20489511D+05 0.0000000 0.0000000 30.099 62.634 EM

609.268

2 -0.77859910D+04 ************ 0.6200011 51.036 43.962 EM

607.002

3 -0.77340571D+04 51.9338889 0.0066702 72.910 38.583 EM

590.507

4 -0.77061431D+04 27.9140530 0.0036092 95.323 37.764 EM

568.913

5 -0.76822287D+04 23.9144195 0.0031033 118.420 38.946 EM

544.634

6 -0.76584945D+04 23.7342072 0.0030895 142.249 41.145 EM

518.607

7 -0.76327219D+04 25.7725865 0.0033652 167.466 43.970 EM

490.564

8 -0.76014966D+04 31.2252971 0.0040910 194.287 47.311 EM

460.402

9 -0.75664121D+04 35.0844530 0.0046155 219.315 51.402 EM

431.283

10 -0.75390276D+04 27.3845381 0.0036192 239.041 56.458 EM

406.501

11 -0.75225831D+04 16.4445022 0.0021812 253.256 62.099 EM

386.645

12 -0.75131857D+04 9.3974379 0.0012492 263.162 67.865 EM

370.973

13 -0.75074569D+04 5.7287720 0.0007625 270.186 73.585 EM

358.229

14 -0.75033528D+04 4.1041300 0.0005467 275.429 79.315 EM

347.256

15 -0.74999592D+04 3.3935161 0.0004523 279.531 85.195 EM

337.273

16 -0.74969876D+04 2.9716136 0.0003962 282.795 91.357 EM

327.848

17 -0.74943336D+04 2.6540156 0.0003540 285.333 97.925 EM

318.742

18 -0.74918714D+04 2.4622484 0.0003285 287.179 105.036 EM

309.785

19 -0.74894342D+04 2.4371899 0.0003253 288.346 112.837 EM

300.817

20 -0.74868504D+04 2.5837345 0.0003450 288.840 121.473 EM

291.687

21 -0.74839698D+04 2.8806779 0.0003848 288.654 131.077 EM

282.269

22 -0.74806862D+04 3.2835273 0.0004387 287.762 141.749 EM

272.490

23 -0.74769684D+04 3.7178755 0.0004970 286.121 153.516 EM

262.363

24 -0.74728706D+04 4.0977872 0.0005481 283.705 166.291 EM

252.004

25 -0.74685026D+04 4.3680098 0.0005845 280.529 179.878 EM

241.593

26 -0.74639806D+04 4.5219689 0.0006055 276.649 194.034 EM

231.317

27 -0.74593986D+04 4.5819475 0.0006139 272.152 208.521 EM

221.327

28 -0.74548298D+04 4.5688362 0.0006125 267.154 223.112 EM

211.734

29 -0.74503532D+04 4.4766380 0.0006005 261.813 237.559 EM

202.628

30 -0.74460908D+04 4.2623616 0.0005721 256.350 251.544 EM

194.106

31 -0.74422199D+04 3.8708608 0.0005199 251.036 264.682 EM

186.281

32 -0.74389285D+04 3.2914281 0.0004423 246.156 276.581 EM

179.263

33 -0.74363343D+04 2.5942066 0.0003487 241.939 286.950 EM

173.111

34 -0.74344332D+04 1.9011240 0.0002557 238.516 295.664 EM

167.820

35 -0.74319314D+04 2.5018068 0.0003365 232.817 315.651 FS

153.532

36 -0.74310834D+04 0.8480126 0.0001141 233.501 320.191 FS

148.308

TECHNICAL 8 OUTPUT FOR STARTING VALUE SET 81

ITER LOGLIKELIHOOD ABS CHANGE REL CHANGE CLASS COUNTS ALGORITHM

1 -0.22010434D+05 0.0000000 0.0000000 287.859 301.549 EM

112.593

2 -0.75137243D+04 ************ 0.6586290 292.379 295.079 EM

114.542

3 -0.74580509D+04 55.6734505 0.0074096 287.679 298.664 EM

115.657

4 -0.74388718D+04 19.1790673 0.0025716 279.906 307.275 EM

114.819

5 -0.74334850D+04 5.3867784 0.0007241 272.913 315.410 EM

113.677

6 -0.74316559D+04 1.8291146 0.0002461 267.400 321.739 EM

112.861

7 -0.74307499D+04 0.9059939 0.0001219 263.113 326.495 EM

112.392

TECHNICAL 8 OUTPUT FOR STARTING VALUE SET 82

ITER LOGLIKELIHOOD ABS CHANGE REL CHANGE CLASS COUNTS ALGORITHM

1 -0.24817017D+05 0.0000000 0.0000000 56.018 203.922 EM

442.060

2 -0.77769404D+04 ************ 0.6866287 77.101 190.452 EM

434.447

3 -0.76941235D+04 82.8168625 0.0106490 97.758 185.696 EM

418.546

4 -0.76615093D+04 32.6142866 0.0042389 118.865 184.450 EM

398.686

5 -0.76366845D+04 24.8247616 0.0032402 140.779 183.773 EM

377.448

6 -0.76159314D+04 20.7531037 0.0027176 162.021 182.706 EM

357.272

7 -0.75992470D+04 16.6843737 0.0021907 182.108 180.640 EM

339.252

8 -0.75849821D+04 14.2649538 0.0018772 201.627 177.340 EM

323.033

9 -0.75699808D+04 15.0012503 0.0019778 221.675 172.763 EM

307.562

10 -0.75513823D+04 18.5984623 0.0024569 242.202 167.025 EM

292.773

11 -0.75314991D+04 19.8832024 0.0026331 261.298 160.839 EM

279.864

12 -0.75160151D+04 15.4840815 0.0020559 277.502 155.397 EM

269.100

13 -0.75061752D+04 9.8398354 0.0013092 290.411 151.479 EM

260.109

14 -0.75004204D+04 5.7548008 0.0007667 300.224 149.189 EM

252.587

15 -0.74970984D+04 3.3220765 0.0004429 307.467 148.285 EM

246.248

16 -0.74950905D+04 2.0078184 0.0002678 312.729 148.463 EM

240.808

17 -0.74937575D+04 1.3330046 0.0001779 316.514 149.466 EM

236.020

18 -0.74927514D+04 1.0061224 0.0001343 319.203 151.110 EM

231.687

19 -0.74918755D+04 0.8759344 0.0001169 321.070 153.276 EM

227.653

TECHNICAL 8 OUTPUT FOR STARTING VALUE SET 83

ITER LOGLIKELIHOOD ABS CHANGE REL CHANGE CLASS COUNTS ALGORITHM

1 -0.21670958D+05 0.0000000 0.0000000 455.645 178.414 EM

67.941

2 -0.76973429D+04 ************ 0.6448084 436.383 170.562 EM

95.055

3 -0.76140974D+04 83.2455174 0.0108148 410.884 167.264 EM

123.853

4 -0.75727025D+04 41.3948441 0.0054366 386.347 166.012 EM

149.641

5 -0.75458717D+04 26.8307760 0.0035431 363.883 167.344 EM

170.773

6 -0.75252262D+04 20.6454883 0.0027360 343.148 172.103 EM

186.748

7 -0.75048379D+04 20.3883978 0.0027093 324.299 180.558 EM

197.143

8 -0.74815096D+04 23.3282705 0.0031084 310.027 190.723 EM

201.250

9 -0.74600030D+04 21.5066256 0.0028746 303.182 199.339 EM

199.478

10 -0.74463780D+04 13.6249068 0.0018264 302.811 204.978 EM

194.211

11 -0.74397515D+04 6.6265847 0.0008899 305.245 208.598 EM

188.157

12 -0.74365443D+04 3.2071655 0.0004311 308.201 211.210 EM

182.590

13 -0.74348729D+04 1.6713730 0.0002248 310.900 213.333 EM

177.767

14 -0.74339019D+04 0.9709959 0.0001306 313.189 215.205 EM

173.606

TECHNICAL 8 OUTPUT FOR STARTING VALUE SET 84

ITER LOGLIKELIHOOD ABS CHANGE REL CHANGE CLASS COUNTS ALGORITHM

1 -0.16238536D+05 0.0000000 0.0000000 0.000 21.183 EM

680.817

2 -0.77922529D+04 8446.2827895 0.5201382 0.002 39.720 EM

662.279

3 -0.77365626D+04 55.6903048 0.0071469 0.052 66.019 EM

635.929

4 -0.76873416D+04 49.2210322 0.0063621 1.287 94.496 EM

606.217

5 -0.76474726D+04 39.8689660 0.0051863 2.421 122.663 EM

576.916

6 -0.76195448D+04 27.9277970 0.0036519 2.902 149.716 EM

549.382

7 -0.75979591D+04 21.5856562 0.0028329 3.187 175.563 EM

523.250

8 -0.75798404D+04 18.1187876 0.0023847 3.372 200.114 EM

498.514

9 -0.75644542D+04 15.3861681 0.0020299 3.510 223.068 EM

475.422

10 -0.75517163D+04 12.7379190 0.0016839 3.630 244.143 EM

454.227

11 -0.75414444D+04 10.2718658 0.0013602 3.745 263.295 EM

434.961

12 -0.75331908D+04 8.2536331 0.0010944 3.858 280.592 EM

417.550

13 -0.75265355D+04 6.6552730 0.0008835 3.972 296.030 EM

401.998

14 -0.75212516D+04 5.2838638 0.0007020 4.082 309.511 EM

388.407

15 -0.75172281D+04 4.0235473 0.0005350 4.188 320.946 EM

376.866

16 -0.75143317D+04 2.8963878 0.0003853 4.287 330.365 EM

367.347

17 -0.75123575D+04 1.9742361 0.0002627 4.384 337.930 EM

359.686

18 -0.75110678D+04 1.2896410 0.0001717 4.482 343.889 EM

353.629

19 -0.75102470D+04 0.8208452 0.0001093 4.588 348.515 EM

348.897

TECHNICAL 8 OUTPUT FOR STARTING VALUE SET 85

ITER LOGLIKELIHOOD ABS CHANGE REL CHANGE CLASS COUNTS ALGORITHM

1 -0.20670160D+05 0.0000000 0.0000000 219.273 296.975 EM

185.752

2 -0.77894740D+04 ************ 0.6231537 241.569 284.284 EM

176.148

3 -0.75860209D+04 203.4531262 0.0261190 267.289 257.695 EM

177.016

4 -0.75099625D+04 76.0584130 0.0100261 284.218 241.430 EM

176.352

5 -0.74838013D+04 26.1611619 0.0034835 293.512 235.882 EM

172.606

6 -0.74691318D+04 14.6695488 0.0019602 296.483 237.547 EM

167.971

7 -0.74594315D+04 9.7003189 0.0012987 294.756 244.046 EM

163.198

8 -0.74521754D+04 7.2560135 0.0009727 290.060 253.714 EM

158.225

9 -0.74463375D+04 5.8379278 0.0007834 283.846 265.061 EM

153.093

10 -0.74415539D+04 4.7835936 0.0006424 277.162 276.763 EM

148.075

11 -0.74377572D+04 3.7967328 0.0005102 270.695 287.820 EM

143.485

12 -0.74349285D+04 2.8287328 0.0003803 264.859 297.612 EM

139.529

13 -0.74329528D+04 1.9756751 0.0002657 259.853 305.878 EM

136.269

14 -0.74316342D+04 1.3185500 0.0001774 255.723 312.621 EM

133.656

15 -0.74307728D+04 0.8613974 0.0001159 252.420 317.997 EM

131.582

TECHNICAL 8 OUTPUT FOR STARTING VALUE SET 86

ITER LOGLIKELIHOOD ABS CHANGE REL CHANGE CLASS COUNTS ALGORITHM

1 -0.23696975D+05 0.0000000 0.0000000 88.570 258.172 EM

355.258

2 -0.75555205D+04 ************ 0.6811610 86.744 275.183 EM

340.073

3 -0.75134695D+04 42.0509895 0.0055656 87.402 287.035 EM

327.563

4 -0.75047054D+04 8.7641093 0.0011665 88.823 295.432 EM

317.745

5 -0.75000771D+04 4.6282875 0.0006167 90.850 301.119 EM

310.031

6 -0.74968876D+04 3.1895018 0.0004253 93.604 304.699 EM

303.698

7 -0.74943244D+04 2.5631815 0.0003419 97.259 306.644 EM

298.097

8 -0.74920397D+04 2.2847428 0.0003049 101.982 307.314 EM

292.704

9 -0.74898254D+04 2.2142987 0.0002956 107.893 306.986 EM

287.121

10 -0.74875003D+04 2.3250774 0.0003104 115.073 305.863 EM

281.063

11 -0.74849000D+04 2.6002759 0.0003473 123.586 304.078 EM

274.336

12 -0.74819035D+04 2.9965426 0.0004003 133.470 301.689 EM

266.842

13 -0.74784633D+04 3.4401624 0.0004598 144.702 298.707 EM

258.591

14 -0.74746164D+04 3.8469323 0.0005144 157.149 295.138 EM

249.714

15 -0.74704603D+04 4.1560582 0.0005560 170.566 291.006 EM

240.428

16 -0.74661116D+04 4.3487215 0.0005821 184.664 286.361 EM

230.975

17 -0.74616708D+04 4.4408042 0.0005948 199.166 281.266 EM

221.568

18 -0.74572096D+04 4.4611532 0.0005979 213.841 275.792 EM

212.367

19 -0.74527845D+04 4.4251095 0.0005934 228.477 270.033 EM

203.490

20 -0.74484720D+04 4.3125598 0.0005787 242.834 264.127 EM

195.039

21 -0.74444042D+04 4.0677312 0.0005461 256.591 258.278 EM

187.131

22 -0.74407658D+04 3.6384727 0.0004888 269.370 252.734 EM

179.896

23 -0.74377317D+04 3.0340251 0.0004078 280.810 247.747 EM

173.443

24 -0.74353884D+04 2.3433092 0.0003151 290.670 243.505 EM

167.824

25 -0.74337006D+04 1.6878179 0.0002270 298.884 240.098 EM

163.017

26 -0.74325455D+04 1.1550907 0.0001554 305.543 237.517 EM

158.940

27 -0.74317744D+04 0.7711529 0.0001038 310.842 235.679 EM

155.479

TECHNICAL 8 OUTPUT FOR STARTING VALUE SET 87

ITER LOGLIKELIHOOD ABS CHANGE REL CHANGE CLASS COUNTS ALGORITHM

1 -0.15904120D+05 0.0000000 0.0000000 83.634 100.903 EM

517.464

2 -0.76387656D+04 8265.3539531 0.5196989 82.519 128.396 EM

491.086

3 -0.75881662D+04 50.5993471 0.0066240 86.060 154.515 EM

461.424

4 -0.75598750D+04 28.2911672 0.0037283 90.150 177.776 EM

434.074

5 -0.75412562D+04 18.6188082 0.0024628 93.805 198.053 EM

410.142

6 -0.75286195D+04 12.6367287 0.0016757 96.919 215.529 EM

389.552

7 -0.75198647D+04 8.7548469 0.0011629 99.518 230.464 EM

372.018

8 -0.75137442D+04 6.1204824 0.0008139 101.663 243.095 EM

357.241

9 -0.75094880D+04 4.2562249 0.0005665 103.438 253.640 EM

344.922

10 -0.75065793D+04 2.9086924 0.0003873 104.930 262.329 EM

334.740

11 -0.75046321D+04 1.9471707 0.0002594 106.225 269.411 EM

326.364

12 -0.75033468D+04 1.2852596 0.0001713 107.389 275.139 EM

319.472

13 -0.75025004D+04 0.8464687 0.0001128 108.477 279.747 EM

313.776

TECHNICAL 8 OUTPUT FOR STARTING VALUE SET 88

ITER LOGLIKELIHOOD ABS CHANGE REL CHANGE CLASS COUNTS ALGORITHM

1 -0.17508164D+05 0.0000000 0.0000000 369.822 49.531 EM

282.647

2 -0.75242889D+04 9983.8752984 0.5702411 364.312 53.881 EM

283.807

3 -0.75077375D+04 16.5513561 0.0021997 357.329 61.638 EM

283.033

4 -0.75020912D+04 5.6463396 0.0007521 350.682 70.323 EM

280.995

5 -0.74982717D+04 3.8194911 0.0005091 344.531 79.521 EM

277.948

6 -0.74948720D+04 3.3997418 0.0004534 338.757 89.228 EM

274.015

7 -0.74915042D+04 3.3677953 0.0004493 333.218 99.518 EM

269.264

8 -0.74880295D+04 3.4746186 0.0004638 327.773 110.514 EM

263.714

9 -0.74843690D+04 3.6605425 0.0004889 322.288 122.342 EM

257.370

10 -0.74804701D+04 3.8988676 0.0005209 316.656 135.079 EM

250.266

11 -0.74763206D+04 4.1495451 0.0005547 310.809 148.698 EM

242.494

12 -0.74719618D+04 4.3588140 0.0005830 304.732 163.050 EM

234.219

13 -0.74674771D+04 4.4846456 0.0006002 298.450 177.905 EM

225.644

14 -0.74629564D+04 4.5207179 0.0006054 292.009 193.017 EM

216.974

15 -0.74584641D+04 4.4923211 0.0006019 285.451 208.172 EM

208.378

16 -0.74540371D+04 4.4269761 0.0005936 278.821 223.194 EM

199.984

17 -0.74497153D+04 4.3217993 0.0005798 272.192 237.907 EM

191.901

18 -0.74455858D+04 4.1294805 0.0005543 265.690 252.067 EM

184.244

19 -0.74418025D+04 3.7833092 0.0005081 259.504 265.349 EM

177.148

20 -0.74385468D+04 3.2557524 0.0004375 253.853 277.401 EM

170.746

21 -0.74359464D+04 2.6004134 0.0003496 248.932 287.945 EM

165.124

22 -0.74340167D+04 1.9296375 0.0002595 244.855 296.848 EM

160.297

23 -0.74326687D+04 1.3480455 0.0001813 241.646 304.146 EM

156.208

24 -0.74317627D+04 0.9059581 0.0001219 239.246 309.995 EM

152.759

TECHNICAL 8 OUTPUT FOR STARTING VALUE SET 89

ITER LOGLIKELIHOOD ABS CHANGE REL CHANGE CLASS COUNTS ALGORITHM

1 -0.27207686D+05 0.0000000 0.0000000 440.439 145.889 EM

115.672

2 -0.76361072D+04 ************ 0.7193401 435.906 128.900 EM

137.194

3 -0.75706108D+04 65.4963842 0.0085772 420.224 130.445 EM

151.331

4 -0.75437688D+04 26.8420035 0.0035456 401.231 141.575 EM

159.194

5 -0.75257777D+04 17.9910696 0.0023849 381.459 158.561 EM

161.980

6 -0.75097262D+04 16.0514631 0.0021329 361.810 179.854 EM

160.336

7 -0.74918876D+04 17.8386742 0.0023754 342.887 204.018 EM

155.095

8 -0.74724424D+04 19.4451321 0.0025955 325.318 228.655 EM

148.028

9 -0.74559751D+04 16.4673044 0.0022037 309.844 251.151 EM

141.005

10 -0.74454845D+04 10.4906392 0.0014070 296.954 270.029 EM

135.017

11 -0.74395297D+04 5.9547788 0.0007998 286.560 285.142 EM

130.298

12 -0.74360141D+04 3.5156251 0.0004726 278.241 297.022 EM

126.738

13 -0.74337918D+04 2.2222893 0.0002989 271.561 306.314 EM

124.125

14 -0.74323200D+04 1.4717690 0.0001980 266.175 313.582 EM

122.244

15 -0.74313125D+04 1.0075255 0.0001356 261.824 319.272 EM

120.904

16 -0.74306046D+04 0.7078720 0.0000953 258.314 323.733 EM

119.953

TECHNICAL 8 OUTPUT FOR STARTING VALUE SET 90

ITER LOGLIKELIHOOD ABS CHANGE REL CHANGE CLASS COUNTS ALGORITHM

1 -0.16548226D+05 0.0000000 0.0000000 542.964 135.856 EM

23.180

2 -0.78205857D+04 8727.6402503 0.5274064 546.758 141.427 EM

13.815

3 -0.77254529D+04 95.1327286 0.0121644 521.535 168.711 EM

11.754

4 -0.76365783D+04 88.8745840 0.0115041 489.632 199.695 EM

12.673

5 -0.75784195D+04 58.1588358 0.0076158 461.853 224.385 EM

15.762

6 -0.75541843D+04 24.2352158 0.0031979 438.878 243.246 EM

19.875

7 -0.75404037D+04 13.7806247 0.0018242 419.055 258.894 EM

24.051

8 -0.75298817D+04 10.5219986 0.0013954 401.573 272.757 EM

27.670

9 -0.75215610D+04 8.3206903 0.0011050 386.358 284.870 EM

30.772

10 -0.75152338D+04 6.3271449 0.0008412 373.614 294.771 EM

33.615

11 -0.75107407D+04 4.4931756 0.0005979 363.395 302.272 EM

36.333

12 -0.75077418D+04 2.9988147 0.0003993 355.483 307.571 EM

38.946

13 -0.75058081D+04 1.9337230 0.0002576 349.492 311.072 EM

41.436

14 -0.75045636D+04 1.2445505 0.0001658 344.997 313.221 EM

43.782

15 -0.75037417D+04 0.8218389 0.0001095 341.619 314.413 EM

45.969

TECHNICAL 8 OUTPUT FOR STARTING VALUE SET 91

ITER LOGLIKELIHOOD ABS CHANGE REL CHANGE CLASS COUNTS ALGORITHM

1 -0.20493271D+05 0.0000000 0.0000000 344.338 315.315 EM

42.347

2 -0.77330526D+04 ************ 0.6226541 334.694 301.659 EM

65.647

3 -0.76583235D+04 74.7291454 0.0096636 326.036 283.348 EM

92.616

4 -0.76184083D+04 39.9151520 0.0052120 316.926 266.593 EM

118.480

5 -0.75921692D+04 26.2391144 0.0034442 306.969 252.168 EM

142.862

6 -0.75725899D+04 19.5793009 0.0025789 296.331 239.622 EM

166.047

7 -0.75566899D+04 15.9000493 0.0020997 285.178 228.464 EM

188.358

8 -0.75429979D+04 13.6919879 0.0018119 273.762 218.334 EM

209.904

9 -0.75309809D+04 12.0169503 0.0015931 262.421 209.069 EM

230.511

10 -0.75205719D+04 10.4090503 0.0013822 251.470 200.666 EM

249.864

11 -0.75117913D+04 8.7805400 0.0011675 241.138 193.204 EM

267.658

12 -0.75045988D+04 7.1925041 0.0009575 231.573 186.758 EM

283.669

13 -0.74988656D+04 5.7332332 0.0007640 222.867 181.351 EM

297.782

14 -0.74943915D+04 4.4740581 0.0005966 215.066 176.943 EM

309.991

15 -0.74909615D+04 3.4300837 0.0004577 208.181 173.460 EM

320.359

16 -0.74883958D+04 2.5656442 0.0003425 202.195 170.820 EM

328.985

17 -0.74865502D+04 1.8456479 0.0002465 197.061 168.938 EM

336.000

18 -0.74852858D+04 1.2643435 0.0001689 192.695 167.728 EM

341.577

19 -0.74844589D+04 0.8269273 0.0001105 188.990 167.090 EM

345.920

TECHNICAL 8 OUTPUT FOR STARTING VALUE SET 92

ITER LOGLIKELIHOOD ABS CHANGE REL CHANGE CLASS COUNTS ALGORITHM

1 -0.18556321D+05 0.0000000 0.0000000 111.335 232.334 EM

358.331

2 -0.77225233D+04 ************ 0.5838333 127.144 220.586 EM

354.270

3 -0.76460211D+04 76.5022276 0.0099064 143.463 211.760 EM

346.777

4 -0.76178919D+04 28.1291798 0.0036789 159.273 203.882 EM

338.845

5 -0.76011170D+04 16.7749524 0.0022020 175.206 196.381 EM

330.413

6 -0.75872780D+04 13.8390008 0.0018207 191.855 189.222 EM

320.924

7 -0.75722007D+04 15.0772601 0.0019872 210.042 182.265 EM

309.692

8 -0.75534304D+04 18.7703168 0.0024788 229.718 175.254 EM

297.028

9 -0.75331872D+04 20.2432451 0.0026800 248.933 168.316 EM

284.751

10 -0.75174416D+04 15.7455213 0.0020902 265.792 162.139 EM

274.069

11 -0.75076362D+04 9.8053959 0.0013044 279.586 157.265 EM

265.149

12 -0.75019142D+04 5.7220148 0.0007622 290.355 153.805 EM

257.839

13 -0.74985746D+04 3.3395977 0.0004452 298.545 151.606 EM

251.849

14 -0.74965535D+04 2.0211122 0.0002695 304.715 150.430 EM

246.855

15 -0.74952404D+04 1.3131183 0.0001752 309.371 150.058 EM

242.572

16 -0.74942960D+04 0.9444290 0.0001260 312.910 150.317 EM

238.774

TECHNICAL 8 OUTPUT FOR STARTING VALUE SET 93

ITER LOGLIKELIHOOD ABS CHANGE REL CHANGE CLASS COUNTS ALGORITHM

1 -0.24034200D+05 0.0000000 0.0000000 87.591 499.598 EM

114.812

2 -0.77012705D+04 ************ 0.6795703 66.627 493.617 EM

141.757

3 -0.76286743D+04 72.5961913 0.0094265 59.600 474.211 EM

168.189

4 -0.75940594D+04 34.6149255 0.0045375 58.762 451.181 EM

192.057

5 -0.75632769D+04 30.7825126 0.0040535 65.583 425.177 EM

211.239

6 -0.75320243D+04 31.2525884 0.0041321 80.468 398.293 EM

223.239

7 -0.75074897D+04 24.5345722 0.0032574 99.139 374.359 EM

228.502

8 -0.74922390D+04 15.2506850 0.0020314 118.164 354.934 EM

228.902

9 -0.74827027D+04 9.5363167 0.0012728 136.541 339.543 EM

225.915

10 -0.74758087D+04 6.8939947 0.0009213 154.238 327.065 EM

220.697

11 -0.74700379D+04 5.7708674 0.0007719 171.383 316.477 EM

214.140

12 -0.74647848D+04 5.2530708 0.0007032 188.054 307.068 EM

206.878

13 -0.74598411D+04 4.9436536 0.0006623 204.274 298.397 EM

199.329

14 -0.74551321D+04 4.7090094 0.0006312 220.031 290.213 EM

191.756

15 -0.74506311D+04 4.5010029 0.0006037 235.270 282.397 EM

184.333

16 -0.74463671D+04 4.2640239 0.0005723 249.851 274.940 EM

177.208

17 -0.74424469D+04 3.9201897 0.0005265 263.532 267.933 EM

170.535

18 -0.74390306D+04 3.4163412 0.0004590 276.004 261.530 EM

164.466

19 -0.74362531D+04 2.7774779 0.0003734 286.993 255.891 EM

159.117

20 -0.74341522D+04 2.1009453 0.0002825 296.348 251.125 EM

154.527

21 -0.74326585D+04 1.4936071 0.0002009 304.075 247.264 EM

150.662

22 -0.74316426D+04 1.0159663 0.0001367 310.306 244.262 EM

147.432

23 -0.74309676D+04 0.6749984 0.0000908 315.250 242.023 EM

144.727

TECHNICAL 8 OUTPUT FOR STARTING VALUE SET 94

ITER LOGLIKELIHOOD ABS CHANGE REL CHANGE CLASS COUNTS ALGORITHM

1 -0.24180782D+05 0.0000000 0.0000000 577.959 37.398 EM

86.642

2 -0.77238778D+04 ************ 0.6805778 567.562 25.354 EM

109.085

3 -0.76454620D+04 78.4157768 0.0101524 541.410 26.336 EM

134.254

4 -0.76089386D+04 36.5234172 0.0047771 513.527 33.209 EM

155.263

5 -0.75838703D+04 25.0682170 0.0032946 485.201 44.206 EM

172.593

6 -0.75613214D+04 22.5489602 0.0029733 456.700 59.154 EM

186.146

7 -0.75396311D+04 21.6903129 0.0028686 428.633 77.720 EM

195.647

8 -0.75193660D+04 20.2650281 0.0026878 402.232 98.626 EM

201.143

9 -0.75021264D+04 17.2395966 0.0022927 378.939 120.088 EM

202.972

10 -0.74888888D+04 13.2376126 0.0017645 359.461 140.808 EM

201.731

11 -0.74790892D+04 9.7996813 0.0013086 343.449 160.357 EM

198.194

12 -0.74714330D+04 7.6561097 0.0010237 330.030 178.834 EM

193.136

13 -0.74649403D+04 6.4927116 0.0008690 318.380 196.437 EM

187.184

14 -0.74591341D+04 5.8062467 0.0007778 307.923 213.296 EM

180.781

15 -0.74538207D+04 5.3134145 0.0007123 298.315 229.452 EM

174.232

16 -0.74489328D+04 4.8878418 0.0006557 289.387 244.846 EM

167.766

17 -0.74444932D+04 4.4396570 0.0005960 281.106 259.308 EM

161.585

18 -0.74406044D+04 3.8887337 0.0005224 273.539 272.577 EM

155.884

19 -0.74373901D+04 3.2143518 0.0004320 266.797 284.383 EM

150.821

20 -0.74349059D+04 2.4841454 0.0003340 260.973 294.544 EM

146.483

21 -0.74330998D+04 1.8061077 0.0002429 256.105 303.027 EM

142.868

22 -0.74318442D+04 1.2555934 0.0001689 252.159 309.935 EM

139.906

23 -0.74309945D+04 0.8497311 0.0001143 249.054 315.456 EM

137.489

TECHNICAL 8 OUTPUT FOR STARTING VALUE SET 95

ITER LOGLIKELIHOOD ABS CHANGE REL CHANGE CLASS COUNTS ALGORITHM

1 -0.15664306D+05 0.0000000 0.0000000 169.918 413.820 EM

118.262

2 -0.75654365D+04 8098.8699325 0.5170270 190.878 403.603 EM

107.518

3 -0.75189697D+04 46.4668730 0.0061420 198.821 392.825 EM

110.354

4 -0.75055661D+04 13.4035293 0.0017826 201.291 385.769 EM

114.940

5 -0.74982577D+04 7.3084361 0.0009737 201.587 381.338 EM

119.075

6 -0.74928668D+04 5.3908496 0.0007189 200.970 378.223 EM

122.808

7 -0.74890513D+04 3.8155675 0.0005092 200.037 375.573 EM

126.390

8 -0.74867223D+04 2.3289749 0.0003110 199.039 373.128 EM

129.833

9 -0.74854449D+04 1.2773743 0.0001706 198.011 370.914 EM

133.075

10 -0.74847666D+04 0.6782920 0.0000906 196.927 368.991 EM

136.082

TECHNICAL 8 OUTPUT FOR STARTING VALUE SET 96

ITER LOGLIKELIHOOD ABS CHANGE REL CHANGE CLASS COUNTS ALGORITHM

1 -0.23278707D+05 0.0000000 0.0000000 168.236 298.844 EM

234.920

2 -0.76164218D+04 ************ 0.6728159 151.313 300.691 EM

249.996

3 -0.75526591D+04 63.7627142 0.0083717 143.162 295.515 EM

263.323

4 -0.75237699D+04 28.8892084 0.0038250 140.742 288.591 EM

272.667

5 -0.75071253D+04 16.6445638 0.0022123 142.978 282.730 EM

276.292

6 -0.74955079D+04 11.6174680 0.0015475 149.240 278.602 EM

274.158

7 -0.74858271D+04 9.6807912 0.0012915 159.161 275.579 EM

267.260

8 -0.74775122D+04 8.3148633 0.0011107 171.986 272.835 EM

257.179

9 -0.74703465D+04 7.1656987 0.0009583 186.662 269.804 EM

245.534

10 -0.74639431D+04 6.4034670 0.0008572 202.244 266.205 EM

233.551

11 -0.74580521D+04 5.8909646 0.0007893 218.059 261.986 EM

221.955

12 -0.74526013D+04 5.4507795 0.0007309 233.624 257.265 EM

211.111

13 -0.74476348D+04 4.9665078 0.0006664 248.529 252.280 EM

201.191

14 -0.74432720D+04 4.3628254 0.0005858 262.370 247.344 EM

192.286

15 -0.74396511D+04 3.6208415 0.0004865 274.782 242.777 EM

184.441

16 -0.74368497D+04 2.8014319 0.0003766 285.505 238.839 EM

177.656

17 -0.74348289D+04 2.0207931 0.0002717 294.450 235.675 EM

171.875

18 -0.74334483D+04 1.3806310 0.0001857 301.702 233.311 EM

166.987

19 -0.74325293D+04 0.9189491 0.0001236 307.464 231.684 EM

162.853

TECHNICAL 8 OUTPUT FOR STARTING VALUE SET 97

ITER LOGLIKELIHOOD ABS CHANGE REL CHANGE CLASS COUNTS ALGORITHM

1 -0.26891735D+05 0.0000000 0.0000000 344.506 294.384 EM

63.110

2 -0.75767707D+04 ************ 0.7182491 346.414 302.339 EM

53.247

3 -0.75376750D+04 39.0957102 0.0051599 345.275 305.994 EM

50.731

4 -0.75265432D+04 11.1317583 0.0014768 343.361 307.877 EM

50.762

5 -0.75171317D+04 9.4114716 0.0012504 340.901 307.648 EM

53.450

6 -0.74998183D+04 17.3134467 0.0023032 336.742 306.389 EM

58.868

7 -0.74770393D+04 22.7790199 0.0030373 329.619 307.855 EM

64.526

8 -0.74602175D+04 16.8217998 0.0022498 320.630 312.545 EM

68.824

9 -0.74503785D+04 9.8390087 0.0013189 311.875 317.527 EM

72.597

10 -0.74441878D+04 6.1906176 0.0008309 304.063 321.768 EM

76.169

11 -0.74402402D+04 3.9476382 0.0005303 297.152 325.430 EM

79.418

12 -0.74376920D+04 2.5481886 0.0003425 291.017 328.631 EM

82.352

13 -0.74358792D+04 1.8128662 0.0002437 285.547 331.414 EM

85.038

14 -0.74344632D+04 1.4159240 0.0001904 280.650 333.831 EM

87.519

15 -0.74333016D+04 1.1616092 0.0001562 276.259 335.930 EM

89.811

16 -0.74323348D+04 0.9667823 0.0001301 272.336 337.742 EM

91.922

TECHNICAL 8 OUTPUT FOR STARTING VALUE SET 98

ITER LOGLIKELIHOOD ABS CHANGE REL CHANGE CLASS COUNTS ALGORITHM

1 -0.18845823D+05 0.0000000 0.0000000 6.296 114.491 EM

581.213

2 -0.77701242D+04 ************ 0.5877005 13.969 130.748 EM

557.283

3 -0.76484000D+04 121.7241924 0.0156657 26.018 160.400 EM

515.582

4 -0.75681199D+04 80.2801194 0.0104963 35.575 187.426 EM

478.998

5 -0.75322448D+04 35.8750597 0.0047403 42.364 210.069 EM

449.566

6 -0.75124093D+04 19.8355550 0.0026334 47.673 228.112 EM

426.214

7 -0.75005120D+04 11.8972212 0.0015837 51.982 241.888 EM

408.129

8 -0.74933972D+04 7.1148432 0.0009486 55.518 252.193 EM

394.289

9 -0.74890918D+04 4.3054019 0.0005746 58.448 259.888 EM

383.664

10 -0.74864087D+04 2.6831396 0.0003583 60.909 265.672 EM

375.419

11 -0.74846846D+04 1.7240767 0.0002303 63.006 270.055 EM

368.940

12 -0.74835502D+04 1.1343627 0.0001516 64.817 273.392 EM

363.791

13 -0.74827904D+04 0.7598429 0.0001015 66.400 275.939 EM

359.661

TECHNICAL 8 OUTPUT FOR STARTING VALUE SET 99

ITER LOGLIKELIHOOD ABS CHANGE REL CHANGE CLASS COUNTS ALGORITHM

1 -0.24446813D+05 0.0000000 0.0000000 644.193 36.526 EM

21.281

2 -0.77679004D+04 ************ 0.6822530 626.048 57.832 EM

18.121

3 -0.77007681D+04 67.1322887 0.0086423 598.262 85.260 EM

18.479

4 -0.76588396D+04 41.9285353 0.0054447 570.154 111.953 EM

19.892

5 -0.76291514D+04 29.6881998 0.0038763 543.410 136.902 EM

21.689

6 -0.76059835D+04 23.1678329 0.0030368 517.894 160.583 EM

23.523

7 -0.75865040D+04 19.4795548 0.0025611 493.464 183.266 EM

25.270

8 -0.75697610D+04 16.7429845 0.0022069 470.311 204.759 EM

26.930

9 -0.75555262D+04 14.2348340 0.0018805 448.612 224.860 EM

28.528

10 -0.75434357D+04 12.0904900 0.0016002 428.395 243.491 EM

30.114

11 -0.75330389D+04 10.3967722 0.0013783 409.793 260.432 EM

31.775

12 -0.75242200D+04 8.8188820 0.0011707 393.201 275.211 EM

33.588

13 -0.75171765D+04 7.0435554 0.0009361 379.072 287.361 EM

35.567

14 -0.75120158D+04 5.1606987 0.0006865 367.615 296.725 EM

37.660

15 -0.75085303D+04 3.4854613 0.0004640 358.696 303.512 EM

39.792

16 -0.75063043D+04 2.2260042 0.0002965 351.945 308.162 EM

41.893

17 -0.75049130D+04 1.3912687 0.0001853 346.913 311.172 EM

43.915

18 -0.75040316D+04 0.8813970 0.0001174 343.176 312.996 EM

45.829

TECHNICAL 8 OUTPUT FOR STARTING VALUE SET 100

ITER LOGLIKELIHOOD ABS CHANGE REL CHANGE CLASS COUNTS ALGORITHM

1 -0.13751249D+05 0.0000000 0.0000000 0.796 361.275 EM

339.929

2 -0.77039435D+04 6047.3060082 0.4397641 1.324 356.106 EM

344.571

3 -0.76681211D+04 35.8223334 0.0046499 2.073 353.138 EM

346.789

4 -0.76323207D+04 35.8004130 0.0046687 2.668 352.822 EM

346.510

5 -0.75826947D+04 49.6260581 0.0065021 2.983 353.198 EM

345.819

6 -0.75346376D+04 48.0570935 0.0063377 3.383 350.931 EM

347.685

7 -0.75145751D+04 20.0625026 0.0026627 3.865 347.220 EM

350.915

8 -0.75102553D+04 4.3197674 0.0005749 4.314 343.712 EM

353.974

9 -0.75093700D+04 0.8852582 0.0001179 4.652 340.972 EM

356.376

TECHNICAL 8 OUTPUT FOR STARTING VALUE SET 101

ITER LOGLIKELIHOOD ABS CHANGE REL CHANGE CLASS COUNTS ALGORITHM

1 -0.23912637D+05 0.0000000 0.0000000 351.866 120.356 EM

229.778

2 -0.76483570D+04 ************ 0.6801542 358.654 111.667 EM

231.679

3 -0.75391746D+04 109.1823903 0.0142753 353.486 120.517 EM

227.997

4 -0.75010355D+04 38.1390794 0.0050588 345.383 134.280 EM

222.337

5 -0.74866594D+04 14.3761279 0.0019166 336.830 149.493 EM

215.677

6 -0.74778971D+04 8.7622972 0.0011704 328.169 165.408 EM

208.423

7 -0.74708771D+04 7.0200086 0.0009388 319.410 181.672 EM

200.918

8 -0.74646504D+04 6.2266471 0.0008335 310.613 198.017 EM

193.370

9 -0.74589316D+04 5.7188421 0.0007661 301.891 214.213 EM

185.896

10 -0.74536318D+04 5.2997961 0.0007105 293.361 230.044 EM

178.595

11 -0.74487319D+04 4.8999343 0.0006574 285.137 245.285 EM

171.578

12 -0.74442782D+04 4.4537150 0.0005979 277.345 259.672 EM

164.982

13 -0.74403850D+04 3.8931587 0.0005230 270.147 272.892 EM

158.961

14 -0.74371810D+04 3.2039912 0.0004306 263.706 284.648 EM

153.646

15 -0.74347229D+04 2.4581351 0.0003305 258.152 294.746 EM

149.102

16 -0.74329553D+04 1.7675770 0.0002377 253.536 303.151 EM

145.313

17 -0.74317446D+04 1.2107337 0.0001629 249.834 309.967 EM

142.199

18 -0.74309390D+04 0.8055201 0.0001084 246.962 315.392 EM

139.646

TECHNICAL 8 OUTPUT FOR STARTING VALUE SET 102

ITER LOGLIKELIHOOD ABS CHANGE REL CHANGE CLASS COUNTS ALGORITHM

1 -0.19599767D+05 0.0000000 0.0000000 505.954 53.530 EM

142.516

2 -0.76287516D+04 ************ 0.6107734 482.277 46.039 EM

173.684

3 -0.75729465D+04 55.8050080 0.0073151 454.622 51.400 EM

195.977

4 -0.75444425D+04 28.5040335 0.0037639 427.271 64.809 EM

209.921

5 -0.75232596D+04 21.1828826 0.0028077 401.753 83.028 EM

217.219

6 -0.75062769D+04 16.9827633 0.0022574 379.434 103.118 EM

219.448

7 -0.74933699D+04 12.9069905 0.0017195 360.895 123.206 EM

217.898

8 -0.74837434D+04 9.6264846 0.0012847 345.640 142.679 EM

213.681

9 -0.74760682D+04 7.6751933 0.0010256 332.731 161.523 EM

207.746

10 -0.74694201D+04 6.6481386 0.0008893 321.372 179.791 EM

200.837

11 -0.74633889D+04 6.0311118 0.0008074 311.051 197.465 EM

193.483

12 -0.74578314D+04 5.5575602 0.0007446 301.479 214.482 EM

186.039

13 -0.74526939D+04 5.1374870 0.0006889 292.503 230.763 EM

178.734

14 -0.74479624D+04 4.7315422 0.0006349 284.071 246.195 EM

171.734

15 -0.74436801D+04 4.2822863 0.0005750 276.217 260.597 EM

165.187

16 -0.74399541D+04 3.7259899 0.0005006 269.045 273.718 EM

159.237

17 -0.74368999D+04 3.0542286 0.0004105 262.686 285.313 EM

154.001

18 -0.74345603D+04 2.3395724 0.0003146 257.238 295.232 EM

149.529

19 -0.74328746D+04 1.6856436 0.0002267 252.734 303.469 EM

145.797

20 -0.74317140D+04 1.1606143 0.0001561 249.136 310.144 EM

142.720

21 -0.74309364D+04 0.7776070 0.0001046 246.357 315.458 EM

140.186

TECHNICAL 8 OUTPUT FOR STARTING VALUE SET 103

ITER LOGLIKELIHOOD ABS CHANGE REL CHANGE CLASS COUNTS ALGORITHM

1 -0.23628671D+05 0.0000000 0.0000000 347.223 194.078 EM

160.699

2 -0.76616379D+04 ************ 0.6757483 340.574 177.315 EM

184.111

3 -0.75561768D+04 105.4611360 0.0137648 329.387 166.896 EM

205.717

4 -0.75131142D+04 43.0625940 0.0056990 318.515 165.786 EM

217.698

5 -0.74910312D+04 22.0829695 0.0029393 309.295 170.982 EM

221.723

6 -0.74788097D+04 12.2215483 0.0016315 301.944 179.653 EM

220.403

7 -0.74706503D+04 8.1593778 0.0010910 295.690 190.550 EM

215.760

8 -0.74643410D+04 6.3092448 0.0008445 289.846 202.888 EM

209.266

9 -0.74589238D+04 5.4172272 0.0007257 284.029 216.086 EM

201.885

10 -0.74539530D+04 4.9708472 0.0006664 278.065 229.741 EM

194.194

11 -0.74492788D+04 4.6741347 0.0006271 271.948 243.500 EM

186.552

12 -0.74449307D+04 4.3481251 0.0005837 265.808 256.970 EM

179.222

13 -0.74410470D+04 3.8836474 0.0005216 259.876 269.704 EM

172.420

14 -0.74377941D+04 3.2529576 0.0004372 254.416 281.272 EM

166.312

15 -0.74352693D+04 2.5247908 0.0003395 249.651 291.358 EM

160.991

16 -0.74334483D+04 1.8209657 0.0002449 245.709 299.829 EM

156.462

17 -0.74322086D+04 1.2397617 0.0001668 242.612 306.731 EM

152.657

18 -0.74313927D+04 0.8158642 0.0001098 240.299 312.235 EM

149.466

TECHNICAL 8 OUTPUT FOR STARTING VALUE SET 104

ITER LOGLIKELIHOOD ABS CHANGE REL CHANGE CLASS COUNTS ALGORITHM

1 -0.20127392D+05 0.0000000 0.0000000 664.544 27.140 EM

10.315

2 -0.78301443D+04 ************ 0.6109708 665.757 15.563 EM

20.680

3 -0.77891510D+04 40.9933643 0.0052353 655.947 15.436 EM

30.617

4 -0.77692187D+04 19.9322581 0.0025590 638.603 20.649 EM

42.748

5 -0.77292996D+04 39.9190876 0.0051381 610.379 31.646 EM

59.975

6 -0.76731914D+04 56.1082566 0.0072592 577.003 47.370 EM

77.628

7 -0.76312138D+04 41.9776179 0.0054707 545.279 64.943 EM

91.778

8 -0.76039727D+04 27.2410440 0.0035697 515.936 83.223 EM

102.841

9 -0.75823864D+04 21.5862709 0.0028388 488.224 102.479 EM

111.297

10 -0.75624983D+04 19.8880950 0.0026229 461.278 123.592 EM

117.130

11 -0.75419104D+04 20.5878991 0.0027224 434.090 147.659 EM

120.251

12 -0.75191064D+04 22.8040695 0.0030236 406.376 174.721 EM

120.903

13 -0.74957919D+04 23.3144700 0.0031007 379.802 202.457 EM

119.741

14 -0.74762256D+04 19.5663121 0.0026103 356.654 227.849 EM

117.497

15 -0.74622458D+04 13.9797911 0.0018699 337.726 249.490 EM

114.784

16 -0.74526126D+04 9.6331768 0.0012909 322.440 267.459 EM

112.101

17 -0.74458095D+04 6.8031520 0.0009129 309.956 282.244 EM

109.799

18 -0.74410168D+04 4.7926938 0.0006437 299.655 294.285 EM

108.060

19 -0.74377187D+04 3.2980342 0.0004432 291.113 303.970 EM

106.917

20 -0.74354594D+04 2.2593093 0.0003038 284.002 311.697 EM

106.300

21 -0.74338664D+04 1.5930053 0.0002142 278.045 317.857 EM

106.098

22 -0.74326881D+04 1.1783491 0.0001585 273.015 322.793 EM

106.193

23 -0.74317771D+04 0.9109780 0.0001226 268.740 326.777 EM

106.482

TECHNICAL 8 OUTPUT FOR STARTING VALUE SET 105

ITER LOGLIKELIHOOD ABS CHANGE REL CHANGE CLASS COUNTS ALGORITHM

1 -0.22170884D+05 0.0000000 0.0000000 527.307 84.986 EM

89.707

2 -0.76513523D+04 ************ 0.6548919 515.219 87.547 EM

99.234

3 -0.75891166D+04 62.2356278 0.0081339 493.778 97.987 EM

110.236

4 -0.75585180D+04 30.5986915 0.0040319 469.348 110.294 EM

122.358

5 -0.75389657D+04 19.5522894 0.0025868 445.221 122.831 EM

133.948

6 -0.75240849D+04 14.8807837 0.0019738 422.985 135.127 EM

143.888

7 -0.75124076D+04 11.6772824 0.0015520 403.465 146.826 EM

151.709

8 -0.75035189D+04 8.8886875 0.0011832 386.951 157.739 EM

157.310

9 -0.74968754D+04 6.6435257 0.0008854 373.264 167.909 EM

160.827

10 -0.74917116D+04 5.1638219 0.0006888 361.922 177.545 EM

162.533

11 -0.74872313D+04 4.4802903 0.0005980 352.320 186.931 EM

162.749

12 -0.74827215D+04 4.5098085 0.0006023 343.836 196.384 EM

161.780

13 -0.74776163D+04 5.1052024 0.0006823 335.889 206.244 EM

159.867

14 -0.74716475D+04 5.9687736 0.0007982 327.993 216.810 EM

157.197

15 -0.74649936D+04 6.6539120 0.0008906 319.845 228.246 EM

153.910

16 -0.74581871D+04 6.8065068 0.0009118 311.361 240.539 EM

150.101

17 -0.74517997D+04 6.3873394 0.0008564 302.668 253.451 EM

145.881

18 -0.74461862D+04 5.6135535 0.0007533 294.058 266.459 EM

141.483

19 -0.74414986D+04 4.6875708 0.0006295 285.881 278.882 EM

137.237

20 -0.74378063D+04 3.6922648 0.0004962 278.437 290.124 EM

133.439

21 -0.74350804D+04 2.7259023 0.0003665 271.911 299.829 EM

130.260

22 -0.74331686D+04 1.9118821 0.0002571 266.360 307.911 EM

127.729

23 -0.74318601D+04 1.3085093 0.0001760 261.745 314.475 EM

125.781

24 -0.74309652D+04 0.8948918 0.0001204 257.974 319.721 EM

124.304

TECHNICAL 8 OUTPUT FOR STARTING VALUE SET 106

ITER LOGLIKELIHOOD ABS CHANGE REL CHANGE CLASS COUNTS ALGORITHM

1 -0.13617375D+05 0.0000000 0.0000000 149.596 2.969 EM

549.435

2 -0.77420508D+04 5875.3246277 0.4314579 158.915 4.663 EM

538.422

3 -0.76933845D+04 48.6663034 0.0062860 171.719 8.395 EM

521.886

4 -0.76537860D+04 39.5985088 0.0051471 183.448 17.338 EM

501.213

5 -0.75968743D+04 56.9116261 0.0074357 193.176 34.709 EM

474.115

6 -0.75385790D+04 58.2953200 0.0076736 203.307 49.706 EM

448.987

7 -0.75120591D+04 26.5199386 0.0035179 213.061 59.649 EM

429.289

8 -0.75006714D+04 11.3876585 0.0015159 220.768 66.993 EM

414.239

9 -0.74950272D+04 5.6442245 0.0007525 226.272 72.808 EM

402.920

10 -0.74919728D+04 3.0544236 0.0004075 229.898 77.678 EM

394.424

11 -0.74901768D+04 1.7959275 0.0002397 232.059 81.945 EM

387.996

12 -0.74890355D+04 1.1413068 0.0001524 233.115 85.806 EM

383.079

13 -0.74882598D+04 0.7756891 0.0001036 233.347 89.377 EM

379.276

TECHNICAL 8 OUTPUT FOR STARTING VALUE SET 107

ITER LOGLIKELIHOOD ABS CHANGE REL CHANGE CLASS COUNTS ALGORITHM

1 -0.14980210D+05 0.0000000 0.0000000 109.118 581.769 EM

11.114

2 -0.77767025D+04 7203.5074997 0.4808683 123.748 553.324 EM

24.928

3 -0.76128683D+04 163.8341750 0.0210673 164.131 493.832 EM

44.037

4 -0.75079802D+04 104.8880798 0.0137777 195.078 444.768 EM

62.155

5 -0.74626998D+04 45.2804587 0.0060310 216.289 409.599 EM

76.112

6 -0.74426031D+04 20.0966471 0.0026929 230.071 386.164 EM

85.765

7 -0.74344617D+04 8.1414198 0.0010939 238.702 371.014 EM

92.283

8 -0.74312361D+04 3.2256040 0.0004339 243.956 361.325 EM

96.719

9 -0.74299546D+04 1.2814736 0.0001724 247.065 355.138 EM

99.798

10 -0.74294336D+04 0.5209682 0.0000701 248.842 351.165 EM

101.992

TECHNICAL 8 OUTPUT FOR STARTING VALUE SET 108

ITER LOGLIKELIHOOD ABS CHANGE REL CHANGE CLASS COUNTS ALGORITHM

1 -0.20786027D+05 0.0000000 0.0000000 633.216 62.055 EM

6.730

2 -0.77263497D+04 ************ 0.6282912 609.338 84.186 EM

8.476

3 -0.76685349D+04 57.8148186 0.0074828 582.275 109.621 EM

10.103

4 -0.76352617D+04 33.2732029 0.0043389 555.426 134.764 EM

11.810

5 -0.76107155D+04 24.5461697 0.0032148 529.018 159.560 EM

13.422

6 -0.75902806D+04 20.4348964 0.0026850 503.355 183.777 EM

14.868

7 -0.75727551D+04 17.5254908 0.0023089 478.909 206.884 EM

16.207

8 -0.75578991D+04 14.8559768 0.0019618 455.998 228.480 EM

17.522

9 -0.75453739D+04 12.5252359 0.0016572 434.727 248.366 EM

18.907

10 -0.75347372D+04 10.6366512 0.0014097 415.246 266.291 EM

20.463

11 -0.75258024D+04 8.9348058 0.0011858 397.917 281.812 EM

22.270

12 -0.75186474D+04 7.1550693 0.0009507 383.151 294.491 EM

24.358

13 -0.75132721D+04 5.3752631 0.0007149 371.126 304.159 EM

26.715

14 -0.75093420D+04 3.9300793 0.0005231 361.671 310.969 EM

29.360

15 -0.75061219D+04 3.2201350 0.0004288 354.338 315.200 EM

32.463

16 -0.75021719D+04 3.9499427 0.0005262 348.472 317.014 EM

36.514

17 -0.74946093D+04 7.5626254 0.0010081 343.124 316.806 EM

42.071

18 -0.74811086D+04 13.5006914 0.0018014 337.014 316.118 EM

48.868

19 -0.74654665D+04 15.6420973 0.0020909 329.324 317.301 EM

55.375

20 -0.74545135D+04 10.9530399 0.0014672 320.797 320.390 EM

60.813

21 -0.74480876D+04 6.4258576 0.0008620 312.644 323.686 EM

65.669

22 -0.74438529D+04 4.2347689 0.0005686 305.248 326.632 EM

70.119

23 -0.74408123D+04 3.0405363 0.0004085 298.599 329.237 EM

74.164

24 -0.74384961D+04 2.3162700 0.0003113 292.614 331.557 EM

77.829

25 -0.74366467D+04 1.8493636 0.0002486 287.208 333.640 EM

81.153

26 -0.74351246D+04 1.5221339 0.0002047 282.309 335.523 EM

84.168

27 -0.74338523D+04 1.2722296 0.0001711 277.870 337.227 EM

86.903

28 -0.74327869D+04 1.0654374 0.0001433 273.866 338.752 EM

89.382

29 -0.74319039D+04 0.8829971 0.0001188 270.287 340.088 EM

91.625

TECHNICAL 8 OUTPUT FOR STARTING VALUE SET 109

ITER LOGLIKELIHOOD ABS CHANGE REL CHANGE CLASS COUNTS ALGORITHM

1 -0.15334828D+05 0.0000000 0.0000000 4.692 689.925 EM

7.383

2 -0.78193275D+04 7515.5006507 0.4900936 15.624 682.247 EM

4.129

3 -0.77727784D+04 46.5491738 0.0059531 30.470 659.043 EM

12.487

4 -0.76974597D+04 75.3186497 0.0096901 47.177 621.800 EM

33.023

5 -0.76373194D+04 60.1403534 0.0078130 63.865 582.408 EM

55.727

6 -0.75925525D+04 44.7668922 0.0058616 79.655 544.481 EM

77.864

7 -0.75596921D+04 32.8603275 0.0043280 94.503 510.079 EM

97.418

8 -0.75350348D+04 24.6573389 0.0032617 108.808 479.021 EM

114.171

9 -0.75147115D+04 20.3232860 0.0026972 123.011 450.680 EM

128.310

10 -0.74964447D+04 18.2668144 0.0024308 137.767 424.240 EM

139.993

11 -0.74782860D+04 18.1587175 0.0024223 153.673 399.128 EM

149.199

12 -0.74601804D+04 18.1055964 0.0024211 169.710 376.579 EM

155.711

13 -0.74463637D+04 13.8166506 0.0018521 183.552 358.919 EM

159.529

14 -0.74389459D+04 7.4177863 0.0009962 194.284 346.504 EM

161.212

15 -0.74354687D+04 3.4771993 0.0004674 202.332 338.201 EM

161.468

16 -0.74337538D+04 1.7149635 0.0002306 208.384 332.786 EM

160.830

17 -0.74328077D+04 0.9460903 0.0001273 213.008 329.345 EM

159.647

TECHNICAL 8 OUTPUT FOR STARTING VALUE SET 110

ITER LOGLIKELIHOOD ABS CHANGE REL CHANGE CLASS COUNTS ALGORITHM

1 -0.22015594D+05 0.0000000 0.0000000 640.722 31.988 EM

29.291

2 -0.77555419D+04 ************ 0.6477251 623.428 29.371 EM

49.201

3 -0.76927358D+04 62.8060822 0.0080982 593.158 39.106 EM

69.736

4 -0.76474059D+04 45.3299033 0.0058926 561.236 54.256 EM

86.508

5 -0.76148236D+04 32.5823657 0.0042606 530.132 72.465 EM

99.403

6 -0.75881148D+04 26.7087696 0.0035075 499.891 92.976 EM

109.133

7 -0.75639661D+04 24.1487240 0.0031824 470.308 115.550 EM

116.142

8 -0.75411419D+04 22.8242085 0.0030175 441.306 139.878 EM

120.816

9 -0.75196040D+04 21.5378678 0.0028560 413.650 164.823 EM

123.527

10 -0.75005775D+04 19.0264566 0.0025302 388.736 188.713 EM

124.551

11 -0.74851268D+04 15.4507824 0.0020599 367.466 210.397 EM

124.137

12 -0.74729806D+04 12.1461967 0.0016227 349.687 229.696 EM

122.617

13 -0.74631259D+04 9.8546338 0.0013187 334.661 246.948 EM

120.391

14 -0.74548512D+04 8.2747062 0.0011087 321.664 262.489 EM

117.848

15 -0.74479823D+04 6.8689360 0.0009214 310.251 276.430 EM

115.319

16 -0.74425954D+04 5.3868389 0.0007233 300.247 288.668 EM

113.085

17 -0.74386669D+04 3.9285594 0.0005278 291.598 299.071 EM

111.331

18 -0.74359474D+04 2.7195320 0.0003656 284.227 307.659 EM

110.113

19 -0.74340820D+04 1.8653337 0.0002509 278.000 314.618 EM

109.382

20 -0.74327639D+04 1.3181354 0.0001773 272.749 320.212 EM

109.039

21 -0.74317893D+04 0.9745553 0.0001311 268.316 324.708 EM

108.976

TECHNICAL 8 OUTPUT FOR STARTING VALUE SET 111

ITER LOGLIKELIHOOD ABS CHANGE REL CHANGE CLASS COUNTS ALGORITHM

1 -0.20376754D+05 0.0000000 0.0000000 595.101 11.717 EM

95.182

2 -0.77334214D+04 ************ 0.6204783 571.951 20.365 EM

109.684

3 -0.76348076D+04 98.6138494 0.0127516 532.974 33.496 EM

135.530

4 -0.75830922D+04 51.7153408 0.0067736 496.257 45.432 EM

160.310

5 -0.75563220D+04 26.7702817 0.0035303 463.921 55.865 EM

182.214

6 -0.75388789D+04 17.4430360 0.0023084 435.831 64.810 EM

201.358

7 -0.75266857D+04 12.1932541 0.0016174 411.841 72.381 EM

217.778

8 -0.75181553D+04 8.5304045 0.0011334 391.667 78.827 EM

231.506

9 -0.75122574D+04 5.8979089 0.0007845 374.889 84.469 EM

242.642

10 -0.75081946D+04 4.0627964 0.0005408 360.989 89.648 EM

251.362

11 -0.75052983D+04 2.8962193 0.0003857 349.405 94.720 EM

257.875

12 -0.75029733D+04 2.3250751 0.0003098 339.576 100.077 EM

262.347

13 -0.75006078D+04 2.3655032 0.0003153 330.971 106.190 EM

264.839

14 -0.74974293D+04 3.1784918 0.0004238 323.161 113.585 EM

265.255

15 -0.74924748D+04 4.9544362 0.0006608 316.042 122.614 EM

263.344

16 -0.74851005D+04 7.3743894 0.0009842 310.004 133.114 EM

258.882

17 -0.74757303D+04 9.3701207 0.0012518 305.690 144.317 EM

251.993

18 -0.74659680D+04 9.7623379 0.0013059 303.655 155.010 EM

243.335

19 -0.74577422D+04 8.2257696 0.0011018 303.625 164.383 EM

233.993

20 -0.74516265D+04 6.1156770 0.0008200 304.623 172.398 EM

224.979

21 -0.74471784D+04 4.4481761 0.0005969 305.896 179.286 EM

216.817

22 -0.74439085D+04 3.2698986 0.0004391 307.136 185.250 EM

209.614

23 -0.74414642D+04 2.4442584 0.0003284 308.282 190.439 EM

203.279

24 -0.74395955D+04 1.8687500 0.0002511 309.361 194.972 EM

197.667

25 -0.74381286D+04 1.4668310 0.0001972 310.407 198.950 EM

192.643

26 -0.74369476D+04 1.1810106 0.0001588 311.444 202.461 EM

188.094

27 -0.74359760D+04 0.9716423 0.0001307 312.483 205.581 EM

183.937

TECHNICAL 8 OUTPUT FOR STARTING VALUE SET 112

ITER LOGLIKELIHOOD ABS CHANGE REL CHANGE CLASS COUNTS ALGORITHM

1 -0.21476030D+05 0.0000000 0.0000000 664.529 9.293 EM

28.178

2 -0.78655142D+04 ************ 0.6337538 675.552 7.803 EM

18.645

3 -0.78401154D+04 25.3988495 0.0032291 669.060 17.403 EM

15.536

4 -0.77865631D+04 53.5522319 0.0068305 646.410 40.286 EM

15.304

5 -0.77220528D+04 64.5103233 0.0082848 613.238 67.125 EM

21.638

6 -0.76371057D+04 84.9471042 0.0110006 570.644 93.791 EM

37.565

7 -0.75776285D+04 59.4772061 0.0077879 534.463 117.005 EM

50.532

8 -0.75510873D+04 26.5411912 0.0035026 504.225 136.779 EM

60.996

9 -0.75333054D+04 17.7819563 0.0023549 477.547 153.869 EM

70.584

10 -0.75194897D+04 13.8156345 0.0018339 453.326 168.832 EM

79.842

11 -0.75079615D+04 11.5281979 0.0015331 431.033 181.885 EM

89.083

12 -0.74981082D+04 9.8533453 0.0013124 410.669 192.902 EM

98.429

13 -0.74897400D+04 8.3681929 0.0011160 392.465 201.631 EM

107.904

14 -0.74825722D+04 7.1678276 0.0009570 376.489 207.917 EM

117.594

15 -0.74759982D+04 6.5739874 0.0008786 362.490 211.773 EM

127.737

16 -0.74692217D+04 6.7765269 0.0009064 350.110 213.312 EM

138.579

17 -0.74618039D+04 7.4177138 0.0009931 339.339 212.720 EM

149.941

18 -0.74544460D+04 7.3579684 0.0009861 330.667 210.342 EM

160.991

19 -0.74485000D+04 5.9459200 0.0007976 324.369 206.755 EM

170.876

20 -0.74443450D+04 4.1550946 0.0005578 320.132 202.577 EM

179.291

21 -0.74415043D+04 2.8406753 0.0003816 317.440 198.234 EM

186.325

22 -0.74394779D+04 2.0263425 0.0002723 315.851 193.952 EM

192.197

23 -0.74379559D+04 1.5219980 0.0002046 315.042 189.829 EM

197.129

24 -0.74367634D+04 1.1925145 0.0001603 314.785 185.902 EM

201.313

25 -0.74357989D+04 0.9645246 0.0001297 314.917 182.180 EM

204.903

TECHNICAL 8 OUTPUT FOR STARTING VALUE SET 113

ITER LOGLIKELIHOOD ABS CHANGE REL CHANGE CLASS COUNTS ALGORITHM

1 -0.26697245D+05 0.0000000 0.0000000 196.106 235.479 EM

270.416

2 -0.77595383D+04 ************ 0.7093506 200.227 239.449 EM

262.324

3 -0.76576722D+04 101.8660909 0.0131279 199.327 237.038 EM

265.634

4 -0.75981247D+04 59.5474411 0.0077762 196.018 229.176 EM

276.806

5 -0.75382213D+04 59.9034562 0.0078840 193.177 222.524 EM

286.299

6 -0.74924699D+04 45.7514111 0.0060693 192.385 218.026 EM

291.589

7 -0.74666690D+04 25.8008356 0.0034436 194.021 212.668 EM

295.311

8 -0.74516257D+04 15.0433507 0.0020147 196.900 205.877 EM

299.223

9 -0.74433107D+04 8.3150268 0.0011159 199.985 198.856 EM

303.159

10 -0.74391086D+04 4.2020338 0.0005645 202.959 192.499 EM

306.542

11 -0.74369072D+04 2.2014398 0.0002959 205.754 187.003 EM

309.243

12 -0.74355977D+04 1.3095369 0.0001761 208.352 182.257 EM

311.391

13 -0.74347017D+04 0.8959227 0.0001205 210.753 178.099 EM

313.148

TECHNICAL 8 OUTPUT FOR STARTING VALUE SET 114

ITER LOGLIKELIHOOD ABS CHANGE REL CHANGE CLASS COUNTS ALGORITHM

1 -0.18585139D+05 0.0000000 0.0000000 1.204 87.667 EM

613.129

2 -0.77988971D+04 ************ 0.5803692 7.947 91.142 EM

602.911

3 -0.77344038D+04 64.4933521 0.0082695 20.774 98.344 EM

582.882

4 -0.77037505D+04 30.6532254 0.0039632 34.940 103.682 EM

563.378

5 -0.76851726D+04 18.5779442 0.0024115 49.549 107.458 EM

544.994

6 -0.76707539D+04 14.4186754 0.0018762 65.073 110.241 EM

526.686

7 -0.76573737D+04 13.3801986 0.0017443 82.074 112.287 EM

507.639

8 -0.76439697D+04 13.4040475 0.0017505 100.564 113.712 EM

487.724

9 -0.76303632D+04 13.6064993 0.0017800 120.085 114.591 EM

467.325

10 -0.76166916D+04 13.6716324 0.0017917 140.146 114.984 EM

446.870

11 -0.76028544D+04 13.8371737 0.0018167 160.630 114.936 EM

426.435

12 -0.75881049D+04 14.7494287 0.0019400 181.862 114.430 EM

405.708

13 -0.75710917D+04 17.0132555 0.0022421 204.130 113.386 EM

384.483

14 -0.75517036D+04 19.3881053 0.0025608 226.434 111.808 EM

363.759

15 -0.75338760D+04 17.8275517 0.0023607 246.803 110.034 EM

345.162

16 -0.75210588D+04 12.8172335 0.0017013 264.197 108.582 EM

329.221

17 -0.75126430D+04 8.4158498 0.0011190 278.471 107.799 EM

315.730

18 -0.75071602D+04 5.4827653 0.0007298 289.853 107.790 EM

304.357

19 -0.75035620D+04 3.5982060 0.0004793 298.748 108.516 EM

294.735

20 -0.75011466D+04 2.4153448 0.0003219 305.614 109.881 EM

286.505

21 -0.74994596D+04 1.6869946 0.0002249 310.869 111.772 EM

279.359

22 -0.74982176D+04 1.2420317 0.0001656 314.867 114.085 EM

273.048

23 -0.74972469D+04 0.9707038 0.0001295 317.890 116.730 EM

267.380

TECHNICAL 8 OUTPUT FOR STARTING VALUE SET 115

ITER LOGLIKELIHOOD ABS CHANGE REL CHANGE CLASS COUNTS ALGORITHM

1 -0.14716269D+05 0.0000000 0.0000000 6.282 55.210 EM

640.508

2 -0.78196489D+04 6896.6205601 0.4686392 18.713 40.209 EM

643.078

3 -0.77750304D+04 44.6184974 0.0057059 33.100 37.332 EM

631.568

4 -0.77477164D+04 27.3139535 0.0035130 47.713 39.248 EM

615.039

5 -0.77261108D+04 21.6055805 0.0027886 62.425 43.752 EM

595.823

6 -0.77061893D+04 19.9215118 0.0025785 77.887 49.559 EM

574.554

7 -0.76857978D+04 20.3915632 0.0026461 94.394 55.944 EM

551.662

8 -0.76640357D+04 21.7620433 0.0028315 111.821 62.563 EM

527.616

9 -0.76398375D+04 24.1982396 0.0031574 130.576 69.344 EM

502.080

10 -0.76099646D+04 29.8728841 0.0039101 151.408 76.309 EM

474.282

11 -0.75738069D+04 36.1577313 0.0047514 172.060 83.496 EM

446.444

12 -0.75437202D+04 30.0866991 0.0039725 188.290 91.288 EM

422.422

13 -0.75261406D+04 17.5795890 0.0023304 199.215 99.285 EM

403.500

14 -0.75161535D+04 9.9871145 0.0013270 205.692 106.845 EM

389.462

15 -0.75096759D+04 6.4775232 0.0008618 208.669 113.768 EM

379.563

16 -0.75044732D+04 5.2027928 0.0006928 209.006 120.004 EM

372.990

17 -0.74994495D+04 5.0236164 0.0006694 207.595 125.433 EM

368.972

18 -0.74944719D+04 4.9776775 0.0006637 205.434 129.841 EM

366.725

19 -0.74901090D+04 4.3628827 0.0005821 203.441 133.068 EM

365.492

20 -0.74869276D+04 3.1814126 0.0004247 202.123 135.195 EM

364.682

21 -0.74849421D+04 1.9855034 0.0002652 201.542 136.502 EM

363.956

22 -0.74837972D+04 1.1448603 0.0001530 201.540 137.276 EM

363.184

23 -0.74831439D+04 0.6533387 0.0000873 201.941 137.711 EM

362.348

TECHNICAL 8 OUTPUT FOR STARTING VALUE SET 116

ITER LOGLIKELIHOOD ABS CHANGE REL CHANGE CLASS COUNTS ALGORITHM

1 -0.25666163D+05 0.0000000 0.0000000 173.192 425.032 EM

103.775

2 -0.77101338D+04 ************ 0.6995993 162.733 415.833 EM

123.434

3 -0.76130571D+04 97.0766805 0.0125908 160.473 398.621 EM

142.906

4 -0.75495987D+04 63.4584195 0.0083355 160.298 384.233 EM

157.470

5 -0.75085792D+04 41.0195255 0.0054333 161.823 373.299 EM

166.878

6 -0.74840696D+04 24.5096323 0.0032642 166.462 362.710 EM

172.828

7 -0.74654532D+04 18.6163682 0.0024875 174.578 351.227 EM

176.195

8 -0.74502490D+04 15.2041770 0.0020366 183.952 340.817 EM

177.230

9 -0.74411505D+04 9.0985534 0.0012212 192.287 333.191 EM

176.523

10 -0.74370503D+04 4.1002104 0.0005510 199.013 328.134 EM

174.853

11 -0.74351663D+04 1.8839936 0.0002533 204.356 324.920 EM

172.724

12 -0.74341206D+04 1.0456849 0.0001406 208.640 322.974 EM

170.386

13 -0.74334248D+04 0.6957453 0.0000936 212.132 321.900 EM

167.968

TECHNICAL 8 OUTPUT FOR STARTING VALUE SET 117

ITER LOGLIKELIHOOD ABS CHANGE REL CHANGE CLASS COUNTS ALGORITHM

1 -0.19859950D+05 0.0000000 0.0000000 30.732 542.887 EM

128.381

2 -0.77627312D+04 ************ 0.6091263 43.818 528.669 EM

129.513

3 -0.76434185D+04 119.3127639 0.0153699 54.568 500.673 EM

146.759

4 -0.75667405D+04 76.6779365 0.0100319 62.242 479.215 EM

160.543

5 -0.75332461D+04 33.4943787 0.0044265 68.760 460.361 EM

172.879

6 -0.75172968D+04 15.9493604 0.0021172 75.389 441.463 EM

185.148

7 -0.75064691D+04 10.8277112 0.0014404 82.420 422.784 EM

196.796

8 -0.74979922D+04 8.4768817 0.0011293 89.796 405.168 EM

207.036

9 -0.74912632D+04 6.7289546 0.0008974 97.391 389.280 EM

215.330

10 -0.74859046D+04 5.3586687 0.0007153 105.129 375.403 EM

221.468

11 -0.74814354D+04 4.4691718 0.0005970 113.055 363.471 EM

225.474

12 -0.74772989D+04 4.1365321 0.0005529 121.342 353.178 EM

227.481

13 -0.74729033D+04 4.3955199 0.0005878 130.248 344.117 EM

227.635

14 -0.74677070D+04 5.1963227 0.0006954 139.944 335.976 EM

226.080

15 -0.74615661D+04 6.1409605 0.0008223 150.200 328.808 EM

222.992

16 -0.74552075D+04 6.3586109 0.0008522 160.293 323.016 EM

218.691

17 -0.74497764D+04 5.4310346 0.0007285 169.508 318.821 EM

213.671

18 -0.74457058D+04 4.0706431 0.0005464 177.538 316.037 EM

208.425

19 -0.74427509D+04 2.9549141 0.0003969 184.393 314.333 EM

203.273

20 -0.74405590D+04 2.1918504 0.0002945 190.216 313.418 EM

198.365

21 -0.74388766D+04 1.6824001 0.0002261 195.176 313.075 EM

193.749

22 -0.74375446D+04 1.3320393 0.0001791 199.431 313.144 EM

189.426

23 -0.74364632D+04 1.0813566 0.0001454 203.112 313.508 EM

185.380

24 -0.74355678D+04 0.8953737 0.0001204 206.328 314.080 EM

181.592

TECHNICAL 8 OUTPUT FOR STARTING VALUE SET 118

ITER LOGLIKELIHOOD ABS CHANGE REL CHANGE CLASS COUNTS ALGORITHM

1 -0.22280674D+05 0.0000000 0.0000000 203.279 162.895 EM

335.826

2 -0.77845046D+04 ************ 0.6506163 218.592 156.034 EM

327.374

3 -0.76792259D+04 105.2787318 0.0135241 228.670 171.841 EM

301.489

4 -0.75973898D+04 81.8360674 0.0106568 232.470 198.771 EM

270.759

5 -0.75432237D+04 54.1661110 0.0071296 229.102 222.862 EM

250.036

6 -0.75188299D+04 24.3937503 0.0032339 221.111 241.633 EM

239.256

7 -0.75066814D+04 12.1485096 0.0016157 211.723 255.797 EM

234.480

8 -0.74993606D+04 7.3208345 0.0009752 202.605 266.134 EM

233.261

9 -0.74945000D+04 4.8606375 0.0006481 194.343 273.210 EM

234.446

10 -0.74910650D+04 3.4349975 0.0004583 187.087 277.455 EM

237.458

11 -0.74884172D+04 2.6477894 0.0003535 180.859 279.201 EM

241.940

12 -0.74860879D+04 2.3292825 0.0003111 175.690 278.690 EM

247.620

13 -0.74836888D+04 2.3990810 0.0003205 171.678 276.085 EM

254.237

14 -0.74808426D+04 2.8461761 0.0003803 169.012 271.489 EM

261.498

15 -0.74771345D+04 3.7081364 0.0004957 167.952 265.001 EM

269.047

16 -0.74721559D+04 4.9785849 0.0006658 168.714 256.813 EM

276.473

17 -0.74657988D+04 6.3571332 0.0008508 171.254 247.351 EM

283.395

18 -0.74587445D+04 7.0542338 0.0009449 175.119 237.333 EM

289.548

19 -0.74523135D+04 6.4310353 0.0008622 179.647 227.589 EM

294.764

20 -0.74473645D+04 4.9489889 0.0006641 184.308 218.712 EM

298.981

21 -0.74438475D+04 3.5170408 0.0004723 188.799 210.912 EM

302.289

22 -0.74413401D+04 2.5073538 0.0003368 192.986 204.127 EM

304.887

23 -0.74394779D+04 1.8622488 0.0002503 196.823 198.194 EM

306.983

24 -0.74380326D+04 1.4452687 0.0001943 200.310 192.946 EM

308.744

25 -0.74368715D+04 1.1611174 0.0001561 203.472 188.244 EM

310.284

26 -0.74359151D+04 0.9563776 0.0001286 206.339 183.983 EM

311.678

TECHNICAL 8 OUTPUT FOR STARTING VALUE SET 119

ITER LOGLIKELIHOOD ABS CHANGE REL CHANGE CLASS COUNTS ALGORITHM

1 -0.24216701D+05 0.0000000 0.0000000 535.101 3.805 EM

163.094

2 -0.78438465D+04 ************ 0.6760976 532.182 2.033 EM

167.785

3 -0.77842353D+04 59.6111550 0.0075997 515.214 2.137 EM

184.650

4 -0.77324195D+04 51.8158141 0.0066565 495.840 3.838 EM

202.322

5 -0.76811751D+04 51.2444524 0.0066272 479.266 7.598 EM

215.135

6 -0.76233819D+04 57.7931169 0.0075240 460.357 15.328 EM

226.315

7 -0.75653106D+04 58.0713789 0.0076175 433.579 28.313 EM

240.108

8 -0.75297054D+04 35.6051729 0.0047064 407.693 39.850 EM

254.457

9 -0.75161427D+04 13.5626397 0.0018012 386.963 47.823 EM

267.214

10 -0.75099014D+04 6.2412953 0.0008304 370.285 53.675 EM

278.041

11 -0.75061480D+04 3.7534188 0.0004998 356.574 58.229 EM

287.197

12 -0.75035902D+04 2.5578252 0.0003408 345.056 61.879 EM

295.065

13 -0.75016671D+04 1.9231327 0.0002563 335.141 64.856 EM

302.002

14 -0.75000772D+04 1.5898763 0.0002119 326.379 67.329 EM

308.292

15 -0.74986574D+04 1.4198081 0.0001893 318.438 69.427 EM

314.135

16 -0.74973269D+04 1.3304544 0.0001774 311.089 71.258 EM

319.653

17 -0.74960553D+04 1.2716171 0.0001696 304.184 72.912 EM

324.904

18 -0.74948417D+04 1.2136348 0.0001619 297.641 74.462 EM

329.897

19 -0.74937003D+04 1.1414292 0.0001523 291.423 75.970 EM

334.607

20 -0.74926495D+04 1.0507086 0.0001402 285.521 77.485 EM

338.994

21 -0.74917047D+04 0.9448370 0.0001261 279.940 79.043 EM

343.017

TECHNICAL 8 OUTPUT FOR STARTING VALUE SET 120

ITER LOGLIKELIHOOD ABS CHANGE REL CHANGE CLASS COUNTS ALGORITHM

1 -0.20040819D+05 0.0000000 0.0000000 133.155 478.790 EM

90.055

2 -0.76237579D+04 ************ 0.6195885 131.854 459.885 EM

110.261

3 -0.75750242D+04 48.7337057 0.0063923 130.622 443.269 EM

128.109

4 -0.75487970D+04 26.2271749 0.0034623 128.756 430.237 EM

143.007

5 -0.75266962D+04 22.1008375 0.0029277 128.693 417.553 EM

155.754

6 -0.75076951D+04 19.0010638 0.0025245 131.578 403.828 EM

166.595

7 -0.74924675D+04 15.2276557 0.0020283 137.290 389.257 EM

175.453

8 -0.74794895D+04 12.9780068 0.0017321 145.693 374.207 EM

182.100

9 -0.74669965D+04 12.4930060 0.0016703 156.418 359.287 EM

186.295

10 -0.74549765D+04 12.0199872 0.0016097 167.995 346.026 EM

187.978

11 -0.74459727D+04 9.0037635 0.0012078 178.509 335.941 EM

187.550

12 -0.74408578D+04 5.1149182 0.0006869 187.177 329.043 EM

185.780

13 -0.74381116D+04 2.7462138 0.0003691 194.123 324.560 EM

183.317

14 -0.74364738D+04 1.6378091 0.0002202 199.704 321.758 EM

180.539

15 -0.74353651D+04 1.1086529 0.0001491 204.244 320.112 EM

177.644

16 -0.74345403D+04 0.8247646 0.0001109 207.998 319.263 EM

174.739

TECHNICAL 8 OUTPUT FOR STARTING VALUE SET 121

ITER LOGLIKELIHOOD ABS CHANGE REL CHANGE CLASS COUNTS ALGORITHM

1 -0.25067742D+05 0.0000000 0.0000000 368.045 128.377 EM

205.578

2 -0.75862572D+04 ************ 0.6973697 352.857 132.439 EM

216.704

3 -0.75337255D+04 52.5317860 0.0069246 337.496 136.909 EM

227.595

4 -0.75186005D+04 15.1249848 0.0020076 324.716 139.787 EM

237.496

5 -0.75114763D+04 7.1241827 0.0009475 313.967 141.987 EM

246.046

6 -0.75069150D+04 4.5612923 0.0006072 304.885 144.196 EM

252.919

7 -0.75031631D+04 3.7519070 0.0004998 297.212 147.010 EM

257.778

8 -0.74990886D+04 4.0745023 0.0005430 290.759 151.015 EM

260.226

9 -0.74934144D+04 5.6742037 0.0007567 285.623 156.616 EM

259.761

10 -0.74850476D+04 8.3668055 0.0011166 282.451 163.634 EM

255.915

11 -0.74743100D+04 10.7375811 0.0014345 282.030 171.249 EM

248.722

12 -0.74632283D+04 11.0816598 0.0014826 284.607 178.314 EM

239.080

13 -0.74541987D+04 9.0296866 0.0012099 289.275 184.182 EM

228.544

14 -0.74478772D+04 6.3214377 0.0008480 294.435 189.021 EM

218.544

15 -0.74436190D+04 4.2581669 0.0005717 299.059 193.175 EM

209.767

16 -0.74407418D+04 2.8772862 0.0003865 302.844 196.863 EM

202.293

17 -0.74387487D+04 1.9930372 0.0002679 305.858 200.200 EM

195.943

18 -0.74373050D+04 1.4437178 0.0001941 308.275 203.243 EM

190.482

19 -0.74362033D+04 1.1016648 0.0001481 310.263 206.031 EM

185.705

20 -0.74353234D+04 0.8799179 0.0001183 311.953 208.591 EM

181.456

TECHNICAL 8 OUTPUT FOR STARTING VALUE SET 122

ITER LOGLIKELIHOOD ABS CHANGE REL CHANGE CLASS COUNTS ALGORITHM

1 -0.18286874D+05 0.0000000 0.0000000 136.280 271.942 EM

293.779

2 -0.77541664D+04 ************ 0.5759709 139.801 281.440 EM

280.759

3 -0.76031179D+04 151.0485156 0.0194797 139.676 295.518 EM

266.806

4 -0.75207291D+04 82.3888139 0.0108362 136.470 309.722 EM

255.807

5 -0.74945616D+04 26.1675452 0.0034794 133.443 321.481 EM

247.076

6 -0.74874294D+04 7.1321330 0.0009516 131.132 330.557 EM

240.311

7 -0.74844293D+04 3.0001064 0.0004007 129.322 337.141 EM

235.538

8 -0.74829775D+04 1.4518604 0.0001940 127.844 341.705 EM

232.451

9 -0.74822832D+04 0.6942755 0.0000928 126.599 344.780 EM

230.621

TECHNICAL 8 OUTPUT FOR STARTING VALUE SET 123

ITER LOGLIKELIHOOD ABS CHANGE REL CHANGE CLASS COUNTS ALGORITHM

1 -0.20084317D+05 0.0000000 0.0000000 586.556 86.543 EM

28.901

2 -0.77292355D+04 ************ 0.6151607 560.682 102.583 EM

38.735

3 -0.76260902D+04 103.1453064 0.0133448 518.648 121.243 EM

62.109

4 -0.75578698D+04 68.2203881 0.0089457 481.387 136.983 EM

83.630

5 -0.75207355D+04 37.1343207 0.0049133 450.557 151.377 EM

100.066

6 -0.74931739D+04 27.5616196 0.0036648 422.479 167.046 EM

112.474

7 -0.74669103D+04 26.2636346 0.0035050 396.509 183.794 EM

121.697

8 -0.74467655D+04 20.1447643 0.0026979 375.691 198.331 EM

127.978

9 -0.74368943D+04 9.8711635 0.0013256 360.916 209.251 EM

131.834

10 -0.74328880D+04 4.0063545 0.0005387 350.935 217.095 EM

133.970

11 -0.74311972D+04 1.6908181 0.0002275 344.334 222.703 EM

134.963

12 -0.74304245D+04 0.7726893 0.0001040 340.039 226.751 EM

135.210

TECHNICAL 8 OUTPUT FOR STARTING VALUE SET 124

ITER LOGLIKELIHOOD ABS CHANGE REL CHANGE CLASS COUNTS ALGORITHM

1 -0.23902730D+05 0.0000000 0.0000000 42.813 66.439 EM

592.748

2 -0.77713151D+04 ************ 0.6748775 65.214 61.636 EM

575.150

3 -0.76960332D+04 75.2819282 0.0096872 91.221 67.775 EM

543.004

4 -0.76551319D+04 40.9013239 0.0053146 115.272 76.740 EM

509.988

5 -0.76254173D+04 29.7145885 0.0038817 137.779 82.589 EM

481.632

6 -0.76064411D+04 18.9761640 0.0024885 159.358 84.853 EM

457.789

7 -0.75904346D+04 16.0065650 0.0021043 181.168 84.845 EM

435.988

8 -0.75727080D+04 17.7265444 0.0023354 203.987 83.286 EM

414.727

9 -0.75525318D+04 20.1762458 0.0026643 226.815 80.460 EM

394.725

10 -0.75344843D+04 18.0475111 0.0023896 247.522 76.755 EM

377.724

11 -0.75220981D+04 12.3861246 0.0016439 265.107 72.769 EM

364.125

12 -0.75142676D+04 7.8305168 0.0010410 279.531 68.973 EM

353.496

13 -0.75093279D+04 4.9397452 0.0006574 291.078 65.573 EM

345.349

14 -0.75062232D+04 3.1047161 0.0004134 300.162 62.602 EM

339.235

15 -0.75042747D+04 1.9484098 0.0002596 307.231 60.017 EM

334.753

16 -0.75030423D+04 1.2324299 0.0001642 312.691 57.751 EM

331.558

17 -0.75022497D+04 0.7926207 0.0001056 316.888 55.738 EM

329.374

TECHNICAL 8 OUTPUT FOR STARTING VALUE SET 125

ITER LOGLIKELIHOOD ABS CHANGE REL CHANGE CLASS COUNTS ALGORITHM

1 -0.18264013D+05 0.0000000 0.0000000 28.987 379.994 EM

293.018

2 -0.76005476D+04 ************ 0.5838512 29.420 375.997 EM

296.583

3 -0.75290103D+04 71.5372960 0.0094121 31.650 367.282 EM

303.068

4 -0.75111795D+04 17.8308331 0.0023683 35.249 357.527 EM

309.224

5 -0.75059868D+04 5.1927156 0.0006913 39.711 348.533 EM

313.756

6 -0.75032329D+04 2.7538381 0.0003669 44.690 340.834 EM

316.475

7 -0.75012034D+04 2.0295152 0.0002705 50.002 334.414 EM

317.585

8 -0.74995399D+04 1.6635389 0.0002218 55.556 329.095 EM

317.349

9 -0.74980889D+04 1.4510180 0.0001935 61.327 324.677 EM

315.996

10 -0.74967477D+04 1.3411719 0.0001789 67.328 320.971 EM

313.702

11 -0.74954372D+04 1.3105134 0.0001748 73.601 317.807 EM

310.592

12 -0.74940911D+04 1.3461264 0.0001796 80.209 315.040 EM

306.751

13 -0.74926479D+04 1.4431617 0.0001926 87.236 312.543 EM

302.221

14 -0.74910440D+04 1.6038841 0.0002141 94.783 310.204 EM

297.012

15 -0.74892082D+04 1.8358171 0.0002451 102.972 307.918 EM

291.110

16 -0.74870609D+04 2.1473396 0.0002867 111.938 305.581 EM

284.481

17 -0.74845223D+04 2.5385087 0.0003391 121.819 303.083 EM

277.098

18 -0.74815347D+04 2.9876171 0.0003992 132.723 300.308 EM

268.968

19 -0.74780900D+04 3.4447260 0.0004604 144.684 297.148 EM

260.168

20 -0.74742429D+04 3.8471161 0.0005145 157.619 293.524 EM

250.857

21 -0.74700940D+04 4.1488470 0.0005551 171.340 289.409 EM

241.251

22 -0.74657565D+04 4.3375297 0.0005807 185.612 284.813 EM

231.575

23 -0.74613267D+04 4.4297937 0.0005933 200.205 279.778 EM

222.017

24 -0.74568744D+04 4.4522679 0.0005967 214.920 274.364 EM

212.716

25 -0.74524580D+04 4.4164328 0.0005923 229.561 268.665 EM

203.773

26 -0.74481601D+04 4.2978771 0.0005767 243.890 262.825 EM

195.285

27 -0.74441210D+04 4.0391691 0.0005423 257.582 257.054 EM

187.364

28 -0.74405286D+04 3.5923865 0.0004826 270.257 251.607 EM

180.136

29 -0.74375534D+04 2.9752113 0.0003999 281.562 246.733 EM

173.705

30 -0.74352707D+04 2.2826463 0.0003069 291.274 242.612 EM

168.114

31 -0.74336351D+04 1.6356618 0.0002200 299.341 239.327 EM

163.333

32 -0.74325186D+04 1.1164962 0.0001502 305.867 236.858 EM

159.276

33 -0.74317727D+04 0.7458839 0.0001004 311.053 235.119 EM

155.827

TECHNICAL 8 OUTPUT FOR STARTING VALUE SET 126

ITER LOGLIKELIHOOD ABS CHANGE REL CHANGE CLASS COUNTS ALGORITHM

1 -0.15239603D+05 0.0000000 0.0000000 12.096 10.230 EM

679.674

2 -0.78498452D+04 7389.7579892 0.4849049 9.861 7.362 EM

684.777

3 -0.78215871D+04 28.2581122 0.0035998 14.009 16.645 EM

671.345

4 -0.77443891D+04 77.1979622 0.0098699 28.167 39.099 EM

634.735

5 -0.76496009D+04 94.7882623 0.0122396 46.408 64.145 EM

591.447

6 -0.75951887D+04 54.4121525 0.0071131 62.000 87.372 EM

552.628

7 -0.75629555D+04 32.2332374 0.0042439 75.729 107.750 EM

518.521

8 -0.75401482D+04 22.8072486 0.0030157 88.560 125.409 EM

488.030

9 -0.75220953D+04 18.0529408 0.0023942 101.073 140.559 EM

460.368

10 -0.75066866D+04 15.4087292 0.0020485 113.602 153.410 EM

434.988

11 -0.74927409D+04 13.9456566 0.0018578 126.497 164.012 EM

411.491

12 -0.74792654D+04 13.4755214 0.0017985 140.209 172.218 EM

389.573

13 -0.74655775D+04 13.6878689 0.0018301 154.635 177.779 EM

369.586

14 -0.74530538D+04 12.5237425 0.0016775 168.344 180.622 EM

353.033

15 -0.74444612D+04 8.5926217 0.0011529 179.889 181.191 EM

340.919

16 -0.74397474D+04 4.7137461 0.0006332 189.023 180.258 EM

332.719

17 -0.74372074D+04 2.5400359 0.0003414 196.157 178.463 EM

327.380

18 -0.74356967D+04 1.5107211 0.0002031 201.780 176.209 EM

324.011

19 -0.74346888D+04 1.0078306 0.0001355 206.286 173.728 EM

321.986

20 -0.74339502D+04 0.7385788 0.0000993 209.966 171.157 EM

320.877

TECHNICAL 8 OUTPUT FOR STARTING VALUE SET 127

ITER LOGLIKELIHOOD ABS CHANGE REL CHANGE CLASS COUNTS ALGORITHM

1 -0.17049503D+05 0.0000000 0.0000000 147.093 344.584 EM

210.323

2 -0.76590358D+04 9390.4672581 0.5507766 178.434 320.600 EM

202.966

3 -0.75930023D+04 66.0335115 0.0086216 204.056 302.707 EM

195.237

4 -0.75715225D+04 21.4797856 0.0028289 227.246 289.296 EM

185.458

5 -0.75506418D+04 20.8807046 0.0027578 249.520 277.486 EM

174.994

6 -0.75264798D+04 24.1619974 0.0032000 269.130 266.674 EM

166.196

7 -0.75027275D+04 23.7523771 0.0031558 284.029 258.521 EM

159.450

8 -0.74820345D+04 20.6929528 0.0027581 292.835 255.169 EM

153.997

9 -0.74663014D+04 15.7330523 0.0021028 295.455 257.272 EM

149.272

10 -0.74553361D+04 10.9653010 0.0014686 293.235 263.895 EM

144.870

11 -0.74475484D+04 7.7877913 0.0010446 288.100 273.393 EM

140.506

12 -0.74417681D+04 5.7802489 0.0007761 281.729 283.985 EM

136.286

13 -0.74375203D+04 4.2477613 0.0005708 275.251 294.252 EM

132.497

14 -0.74345651D+04 2.9552442 0.0003973 269.301 303.359 EM

129.340

15 -0.74326172D+04 1.9478809 0.0002620 264.158 310.991 EM

126.852

16 -0.74313664D+04 1.2508688 0.0001683 259.882 317.161 EM

124.957

17 -0.74305611D+04 0.8052414 0.0001084 256.419 322.045 EM

123.537

TECHNICAL 8 OUTPUT FOR STARTING VALUE SET 128

ITER LOGLIKELIHOOD ABS CHANGE REL CHANGE CLASS COUNTS ALGORITHM

1 -0.17871360D+05 0.0000000 0.0000000 3.869 398.069 EM

300.062

2 -0.77244827D+04 ************ 0.5677731 6.142 392.291 EM

303.567

3 -0.76870148D+04 37.4679648 0.0048505 8.643 385.610 EM

307.747

4 -0.76521041D+04 34.9106907 0.0045415 12.953 379.376 EM

309.671

5 -0.75956867D+04 56.4174014 0.0073728 20.257 373.176 EM

308.568

6 -0.75343800D+04 61.3066887 0.0080713 25.778 367.724 EM

308.497

7 -0.75020198D+04 32.3601404 0.0042950 30.014 360.026 EM

311.960

8 -0.74913675D+04 10.6523190 0.0014199 33.996 351.065 EM

316.939

9 -0.74876393D+04 3.7281681 0.0004977 37.744 342.617 EM

321.638

10 -0.74857350D+04 1.9043116 0.0002543 41.180 335.338 EM

325.481

11 -0.74845422D+04 1.1928833 0.0001594 44.292 329.249 EM

328.458

12 -0.74837323D+04 0.8098548 0.0001082 47.107 324.175 EM

330.718

TECHNICAL 8 OUTPUT FOR STARTING VALUE SET 129

ITER LOGLIKELIHOOD ABS CHANGE REL CHANGE CLASS COUNTS ALGORITHM

1 -0.16923232D+05 0.0000000 0.0000000 38.811 554.274 EM

108.915

2 -0.78067757D+04 9116.4566921 0.5386948 63.772 535.305 EM

102.923

3 -0.76943157D+04 112.4600449 0.0144054 100.384 484.563 EM

117.053

4 -0.75756305D+04 118.6851878 0.0154250 137.524 431.174 EM

133.303

5 -0.74943517D+04 81.2787705 0.0107290 166.304 391.873 EM

143.823

6 -0.74567113D+04 37.6404645 0.0050225 185.589 367.676 EM

148.735

7 -0.74418337D+04 14.8775977 0.0019952 198.411 352.981 EM

150.608

8 -0.74357399D+04 6.0938152 0.0008189 207.282 343.667 EM

151.051

9 -0.74330928D+04 2.6470707 0.0003560 213.624 337.651 EM

150.725

10 -0.74318702D+04 1.2225822 0.0001645 218.276 333.792 EM

149.932

11 -0.74312487D+04 0.6214995 0.0000836 221.776 331.379 EM

148.846

TECHNICAL 8 OUTPUT FOR STARTING VALUE SET 130

ITER LOGLIKELIHOOD ABS CHANGE REL CHANGE CLASS COUNTS ALGORITHM

1 -0.22518521D+05 0.0000000 0.0000000 368.516 309.311 EM

24.173

2 -0.77619300D+04 ************ 0.6553091 350.773 305.955 EM

45.272

3 -0.76844535D+04 77.4765693 0.0099816 332.565 299.814 EM

69.622

4 -0.76378586D+04 46.5948493 0.0060635 316.769 291.818 EM

93.413

5 -0.76081639D+04 29.6947516 0.0038878 303.881 282.600 EM

115.519

6 -0.75875266D+04 20.6372411 0.0027125 293.251 272.611 EM

136.138

7 -0.75715964D+04 15.9302407 0.0020995 284.406 261.764 EM

155.830

8 -0.75576757D+04 13.9207254 0.0018385 276.894 250.039 EM

175.067

9 -0.75444000D+04 13.2757091 0.0017566 270.463 237.721 EM

193.816

10 -0.75315395D+04 12.8604751 0.0017046 265.181 225.364 EM

211.455

11 -0.75196355D+04 11.9039529 0.0015805 261.342 213.537 EM

227.121

12 -0.75093303D+04 10.3052589 0.0013704 259.233 202.661 EM

240.106

13 -0.75009007D+04 8.4295426 0.0011225 258.982 193.040 EM

249.978

14 -0.74942518D+04 6.6489087 0.0008864 260.562 184.881 EM

256.557

15 -0.74889800D+04 5.2718663 0.0007035 263.825 178.294 EM

259.881

16 -0.74844231D+04 4.5568362 0.0006085 268.477 173.422 EM

260.101

17 -0.74798558D+04 4.5672912 0.0006102 274.064 170.503 EM

257.433

18 -0.74745817D+04 5.2741201 0.0007051 280.010 169.790 EM

252.200

19 -0.74680966D+04 6.4851134 0.0008676 285.761 171.334 EM

244.905

20 -0.74606330D+04 7.4635475 0.0009994 290.996 174.711 EM

236.293

21 -0.74534979D+04 7.1351070 0.0009564 295.601 179.101 EM

227.298

22 -0.74479582D+04 5.5397357 0.0007432 299.479 183.778 EM

218.743

23 -0.74441287D+04 3.8295466 0.0005142 302.608 188.333 EM

211.059

24 -0.74414897D+04 2.6389372 0.0003545 305.100 192.590 EM

204.311

25 -0.74395732D+04 1.9164782 0.0002575 307.124 196.490 EM

198.386

26 -0.74381023D+04 1.4709410 0.0001977 308.832 200.032 EM

193.136

27 -0.74369264D+04 1.1759116 0.0001581 310.333 203.239 EM

188.428

28 -0.74359601D+04 0.9662965 0.0001299 311.698 206.142 EM

184.159

TECHNICAL 8 OUTPUT FOR STARTING VALUE SET 131

ITER LOGLIKELIHOOD ABS CHANGE REL CHANGE CLASS COUNTS ALGORITHM

1 -0.21358460D+05 0.0000000 0.0000000 266.913 213.414 EM

221.673

2 -0.76637291D+04 ************ 0.6411853 256.077 231.226 EM

214.698

3 -0.75297480D+04 133.9811874 0.0174825 248.674 246.107 EM

207.219

4 -0.75135180D+04 16.2299951 0.0021554 243.490 255.729 EM

202.781

5 -0.75077941D+04 5.7238712 0.0007618 240.287 262.116 EM

199.597

6 -0.75021316D+04 5.6624596 0.0007542 239.108 265.257 EM

197.635

7 -0.74950118D+04 7.1198063 0.0009490 239.588 264.690 EM

197.722

8 -0.74860420D+04 8.9698425 0.0011968 240.839 260.071 EM

201.089

9 -0.74762751D+04 9.7668365 0.0013047 241.804 251.773 EM

208.423

10 -0.74671121D+04 9.1630099 0.0012256 241.735 240.996 EM

219.269

11 -0.74591463D+04 7.9658336 0.0010668 240.414 229.227 EM

232.359

12 -0.74523579D+04 6.7884096 0.0009101 238.082 217.668 EM

246.250

13 -0.74466708D+04 5.6871190 0.0007631 235.192 207.029 EM

259.780

14 -0.74421214D+04 4.5493597 0.0006109 232.221 197.628 EM

272.151

15 -0.74387117D+04 3.4097088 0.0004582 229.560 189.537 EM

282.903

16 -0.74363140D+04 2.3976745 0.0003223 227.444 182.683 EM

291.873

17 -0.74347014D+04 1.6126113 0.0002169 225.957 176.915 EM

299.129

18 -0.74336299D+04 1.0715048 0.0001441 225.070 172.050 EM

304.880

19 -0.74329007D+04 0.7291925 0.0000981 224.695 167.909 EM

309.396

TECHNICAL 8 OUTPUT FOR STARTING VALUE SET 132

ITER LOGLIKELIHOOD ABS CHANGE REL CHANGE CLASS COUNTS ALGORITHM

1 -0.23449735D+05 0.0000000 0.0000000 551.873 145.412 EM

4.715

2 -0.78478863D+04 ************ 0.6653316 559.537 139.290 EM

3.173

3 -0.78115892D+04 36.2971114 0.0046251 558.065 139.573 EM

4.363

4 -0.77703831D+04 41.2061281 0.0052750 546.592 143.159 EM

12.249

5 -0.77022170D+04 68.1661049 0.0087726 523.225 147.886 EM

30.890

6 -0.76443090D+04 57.9079871 0.0075184 494.569 154.147 EM

53.285

7 -0.75983891D+04 45.9198645 0.0060071 465.013 161.246 EM

75.741

8 -0.75628819D+04 35.5071803 0.0046730 437.734 168.721 EM

95.545

9 -0.75362014D+04 26.6805060 0.0035278 414.098 176.510 EM

111.392

10 -0.75168635D+04 19.3379353 0.0025660 394.035 184.809 EM

123.156

11 -0.75034195D+04 13.4439874 0.0017885 377.224 193.476 EM

131.301

12 -0.74939588D+04 9.4606722 0.0012608 363.315 202.301 EM

136.384

13 -0.74867669D+04 7.1918906 0.0009597 351.793 211.258 EM

138.950

14 -0.74804501D+04 6.3167996 0.0008437 341.962 220.489 EM

139.549

15 -0.74740002D+04 6.4499343 0.0008622 333.081 230.220 EM

138.699

16 -0.74670144D+04 6.9858017 0.0009347 324.537 240.605 EM

136.858

17 -0.74597156D+04 7.2988394 0.0009775 315.968 251.671 EM

134.361

18 -0.74526807D+04 7.0348334 0.0009430 307.288 263.299 EM

131.414

19 -0.74464734D+04 6.2073601 0.0008329 298.663 275.106 EM

128.231

20 -0.74414272D+04 5.0461866 0.0006777 290.430 286.444 EM

125.126

21 -0.74376271D+04 3.8000440 0.0005107 282.916 296.684 EM

122.400

22 -0.74349478D+04 2.6793063 0.0003602 276.319 305.466 EM

120.215

23 -0.74331265D+04 1.8212897 0.0002450 270.684 312.729 EM

118.586

24 -0.74318877D+04 1.2387961 0.0001667 265.956 318.605 EM

117.439

25 -0.74310240D+04 0.8637630 0.0001162 262.032 323.303 EM

116.665

TECHNICAL 8 OUTPUT FOR STARTING VALUE SET 133

ITER LOGLIKELIHOOD ABS CHANGE REL CHANGE CLASS COUNTS ALGORITHM

1 -0.23184966D+05 0.0000000 0.0000000 223.010 373.033 EM

105.958

2 -0.78232543D+04 ************ 0.6625721 239.207 346.519 EM

116.273

3 -0.76930791D+04 130.1751256 0.0166395 257.706 314.125 EM

130.170

4 -0.75708308D+04 122.2483809 0.0158907 270.792 290.783 EM

140.424

5 -0.74982959D+04 72.5348825 0.0095808 277.314 279.012 EM

145.674

6 -0.74681681D+04 30.1277910 0.0040180 279.627 275.494 EM

146.879

7 -0.74543871D+04 13.7809612 0.0018453 278.686 277.866 EM

145.448

8 -0.74459996D+04 8.3875309 0.0011252 275.498 283.920 EM

142.582

9 -0.74402272D+04 5.7724394 0.0007752 271.101 291.739 EM

139.160

10 -0.74362413D+04 3.9858928 0.0005357 266.358 299.822 EM

135.820

11 -0.74336013D+04 2.6400022 0.0003550 261.843 307.253 EM

132.905

12 -0.74319295D+04 1.6717880 0.0002249 257.863 313.619 EM

130.518

13 -0.74308929D+04 1.0365765 0.0001395 254.533 318.843 EM

128.624

14 -0.74302468D+04 0.6460865 0.0000869 251.846 323.020 EM

127.134

TECHNICAL 8 OUTPUT FOR STARTING VALUE SET 134

ITER LOGLIKELIHOOD ABS CHANGE REL CHANGE CLASS COUNTS ALGORITHM

1 -0.25569310D+05 0.0000000 0.0000000 71.795 347.024 EM

283.180

2 -0.77319847D+04 ************ 0.6976068 67.398 349.649 EM

284.953

3 -0.75800647D+04 151.9200236 0.0196483 70.860 339.691 EM

291.449

4 -0.75259518D+04 54.1129220 0.0071388 78.115 325.819 EM

298.066

5 -0.74990211D+04 26.9306117 0.0035784 85.183 315.347 EM

301.471

6 -0.74878694D+04 11.1517278 0.0014871 89.371 310.651 EM

301.978

7 -0.74847845D+04 3.0849149 0.0004120 91.746 308.654 EM

301.600

8 -0.74838011D+04 0.9834001 0.0001314 93.289 307.430 EM

301.280

TECHNICAL 8 OUTPUT FOR STARTING VALUE SET 135

ITER LOGLIKELIHOOD ABS CHANGE REL CHANGE CLASS COUNTS ALGORITHM

1 -0.16584845D+05 0.0000000 0.0000000 0.036 279.484 EM

422.480

2 -0.75785691D+04 9006.2761230 0.5430425 1.580 295.826 EM

404.594

3 -0.75310607D+04 47.5084002 0.0062688 2.379 310.832 EM

388.789

4 -0.75199807D+04 11.0800281 0.0014712 2.907 323.265 EM

375.828

5 -0.75148937D+04 5.0870067 0.0006765 3.394 333.148 EM

365.457

6 -0.75122026D+04 2.6911517 0.0003581 3.818 340.748 EM

357.434

7 -0.75107416D+04 1.4609768 0.0001945 4.162 346.474 EM

351.365

8 -0.75099568D+04 0.7847429 0.0001045 4.419 350.742 EM

346.839

TECHNICAL 8 OUTPUT FOR STARTING VALUE SET 136

ITER LOGLIKELIHOOD ABS CHANGE REL CHANGE CLASS COUNTS ALGORITHM

1 -0.25146056D+05 0.0000000 0.0000000 584.345 4.800 EM

112.854

2 -0.77273609D+04 ************ 0.6927009 570.371 2.183 EM

129.446

3 -0.76421134D+04 85.2474574 0.0110319 543.643 1.717 EM

156.640

4 -0.76079410D+04 34.1724307 0.0044716 517.985 1.811 EM

182.203

5 -0.75885222D+04 19.4188215 0.0025524 493.581 2.081 EM

206.338

6 -0.75730790D+04 15.4431404 0.0020351 470.736 2.367 EM

228.896

7 -0.75606034D+04 12.4756234 0.0016474 449.773 2.727 EM

249.500

8 -0.75505606D+04 10.0428137 0.0013283 430.468 3.476 EM

268.056

9 -0.75413459D+04 9.2147366 0.0012204 411.658 5.723 EM

284.619

10 -0.75289143D+04 12.4315693 0.0016485 391.745 11.152 EM

299.103

11 -0.75162960D+04 12.6183307 0.0016760 373.822 17.092 EM

311.085

12 -0.75095337D+04 6.7622645 0.0008997 359.339 22.002 EM

320.659

13 -0.75053699D+04 4.1637619 0.0005545 347.163 26.465 EM

328.372

14 -0.75023036D+04 3.0663763 0.0004086 336.618 30.733 EM

334.648

15 -0.74999104D+04 2.3932034 0.0003190 327.343 34.860 EM

339.798

16 -0.74979888D+04 1.9215568 0.0002562 319.095 38.850 EM

344.055

17 -0.74964195D+04 1.5692929 0.0002093 311.701 42.698 EM

347.600

18 -0.74951239D+04 1.2955921 0.0001728 305.026 46.403 EM

350.571

19 -0.74940451D+04 1.0788454 0.0001439 298.963 49.964 EM

353.074

20 -0.74931386D+04 0.9065141 0.0001210 293.423 53.390 EM

355.187

TECHNICAL 8 OUTPUT FOR STARTING VALUE SET 137

ITER LOGLIKELIHOOD ABS CHANGE REL CHANGE CLASS COUNTS ALGORITHM

1 -0.16144408D+05 0.0000000 0.0000000 323.375 326.866 EM

51.759

2 -0.75369723D+04 8607.4356452 0.5331528 316.101 329.170 EM

56.729

3 -0.74680357D+04 68.9366584 0.0091465 307.513 332.458 EM

62.029

4 -0.74490416D+04 18.9940615 0.0025434 299.462 335.262 EM

67.276

5 -0.74413529D+04 7.6886823 0.0010322 292.397 337.488 EM

72.116

6 -0.74375253D+04 3.8276458 0.0005144 286.265 339.390 EM

76.345

7 -0.74352970D+04 2.2283003 0.0002996 280.970 340.961 EM

80.069

8 -0.74337606D+04 1.5363188 0.0002066 276.397 342.217 EM

83.386

9 -0.74326089D+04 1.1517182 0.0001549 272.444 343.204 EM

86.351

10 -0.74317250D+04 0.8838878 0.0001189 269.036 343.963 EM

89.001

TECHNICAL 8 OUTPUT FOR STARTING VALUE SET 138

ITER LOGLIKELIHOOD ABS CHANGE REL CHANGE CLASS COUNTS ALGORITHM

1 -0.19276738D+05 0.0000000 0.0000000 354.540 86.483 EM

260.977

2 -0.78089561D+04 ************ 0.5949026 345.783 109.314 EM

246.903

3 -0.76549589D+04 153.9972110 0.0197206 328.998 143.690 EM

229.312

4 -0.75869178D+04 68.0410174 0.0088885 314.340 172.086 EM

215.574

5 -0.75549559D+04 31.9619189 0.0042128 301.513 195.965 EM

204.522

6 -0.75363788D+04 18.5770686 0.0024589 290.216 216.668 EM

195.116

7 -0.75246402D+04 11.7386139 0.0015576 280.163 234.776 EM

187.061

8 -0.75166536D+04 7.9866809 0.0010614 271.147 250.636 EM

180.217

9 -0.75108847D+04 5.7688477 0.0007675 263.052 264.580 EM

174.368

10 -0.75065021D+04 4.3825729 0.0005835 255.781 276.933 EM

169.286

11 -0.75030039D+04 3.4982688 0.0004660 249.220 287.979 EM

164.801

12 -0.75000688D+04 2.9351131 0.0003912 243.233 297.954 EM

160.814

13 -0.74974916D+04 2.5771783 0.0003436 237.681 307.033 EM

157.286

14 -0.74951547D+04 2.3369153 0.0003117 232.451 315.336 EM

154.213

15 -0.74930109D+04 2.1437481 0.0002860 227.472 322.921 EM

151.607

16 -0.74910646D+04 1.9462912 0.0002597 222.728 329.791 EM

149.481

17 -0.74893480D+04 1.7166660 0.0002292 218.244 335.917 EM

147.838

18 -0.74878964D+04 1.4515235 0.0001938 214.065 341.260 EM

146.676

19 -0.74867283D+04 1.1680882 0.0001560 210.227 345.797 EM

145.976

20 -0.74858340D+04 0.8943623 0.0001195 206.748 349.544 EM

145.707

TECHNICAL 8 OUTPUT FOR STARTING VALUE SET 139

ITER LOGLIKELIHOOD ABS CHANGE REL CHANGE CLASS COUNTS ALGORITHM

1 -0.19097010D+05 0.0000000 0.0000000 327.451 296.640 EM

77.910

2 -0.76902603D+04 ************ 0.5973055 323.342 276.855 EM

101.803

3 -0.76077854D+04 82.4749182 0.0107246 310.988 269.059 EM

121.953

4 -0.75790332D+04 28.7521914 0.0037793 295.619 267.491 EM

138.889

5 -0.75598658D+04 19.1673677 0.0025290 279.253 268.603 EM

154.144

6 -0.75402612D+04 19.6045931 0.0025932 263.164 271.403 EM

167.433

7 -0.75168770D+04 23.3842779 0.0031013 249.357 275.675 EM

176.967

8 -0.74889001D+04 27.9768378 0.0037219 239.928 281.166 EM

180.907

9 -0.74604283D+04 28.4718021 0.0038019 234.503 288.165 EM

179.331

10 -0.74422787D+04 18.1496241 0.0024328 231.300 295.880 EM

174.820

11 -0.74354080D+04 6.8707007 0.0009232 229.272 302.744 EM

169.984

12 -0.74331427D+04 2.2652931 0.0003047 228.093 308.191 EM

165.716

13 -0.74322358D+04 0.9068469 0.0001220 227.550 312.388 EM

162.063

TECHNICAL 8 OUTPUT FOR STARTING VALUE SET 140

ITER LOGLIKELIHOOD ABS CHANGE REL CHANGE CLASS COUNTS ALGORITHM

1 -0.21500689D+05 0.0000000 0.0000000 578.164 41.151 EM

82.685

2 -0.76271638D+04 ************ 0.6452596 551.088 47.667 EM

103.245

3 -0.75919832D+04 35.1806591 0.0046125 523.306 55.792 EM

122.903

4 -0.75709682D+04 21.0149922 0.0027681 496.857 64.105 EM

141.038

5 -0.75538045D+04 17.1637154 0.0022670 472.037 72.373 EM

157.590

6 -0.75391897D+04 14.6148250 0.0019348 449.184 80.522 EM

172.294

7 -0.75270761D+04 12.1135515 0.0016067 428.623 88.579 EM

184.799

8 -0.75174322D+04 9.6438851 0.0012812 410.619 96.523 EM

194.857

9 -0.75100808D+04 7.3514123 0.0009779 395.306 104.261 EM

202.432

10 -0.75046830D+04 5.3977939 0.0007187 382.599 111.715 EM

207.685

11 -0.75007787D+04 3.9043020 0.0005202 372.204 118.882 EM

210.914

12 -0.74978947D+04 2.8839935 0.0003845 363.713 125.811 EM

212.476

13 -0.74956469D+04 2.2477978 0.0002998 356.719 132.562 EM

212.720

14 -0.74937679D+04 1.8789683 0.0002507 350.872 139.183 EM

211.945

15 -0.74920751D+04 1.6928569 0.0002259 345.892 145.718 EM

210.390

16 -0.74904209D+04 1.6541471 0.0002208 341.548 152.217 EM

208.235

17 -0.74886488D+04 1.7721674 0.0002366 337.629 158.759 EM

205.612

18 -0.74865567D+04 2.0921118 0.0002794 333.908 165.476 EM

202.616

19 -0.74838793D+04 2.6773598 0.0003576 330.122 172.576 EM

199.301

20 -0.74803235D+04 3.5557724 0.0004751 325.968 180.361 EM

195.671

21 -0.74757017D+04 4.6218022 0.0006179 321.138 189.184 EM

191.679

22 -0.74701242D+04 5.5775602 0.0007461 315.412 199.342 EM

187.246

23 -0.74640693D+04 6.0548733 0.0008105 308.762 210.945 EM

182.293

24 -0.74581454D+04 5.9238715 0.0007937 301.379 223.805 EM

176.815

25 -0.74526999D+04 5.4455140 0.0007301 293.596 237.450 EM

170.954

26 -0.74477753D+04 4.9245750 0.0006608 285.758 251.287 EM

164.956

27 -0.74433723D+04 4.4030200 0.0005912 278.170 264.721 EM

159.109

28 -0.74395829D+04 3.7894091 0.0005091 271.101 277.217 EM

153.682

29 -0.74365217D+04 3.0611876 0.0004115 264.769 288.356 EM

148.875

30 -0.74342171D+04 2.3046706 0.0003099 259.313 297.906 EM

144.781

31 -0.74325833D+04 1.6337577 0.0002198 254.780 305.828 EM

141.392

32 -0.74314716D+04 1.1116473 0.0001496 251.137 312.237 EM

138.627

33 -0.74307316D+04 0.7400018 0.0000996 248.296 317.330 EM

136.375

TECHNICAL 8 OUTPUT FOR STARTING VALUE SET 141

ITER LOGLIKELIHOOD ABS CHANGE REL CHANGE CLASS COUNTS ALGORITHM

1 -0.18854494D+05 0.0000000 0.0000000 39.988 396.728 EM

265.284

2 -0.75503270D+04 ************ 0.5995476 39.475 384.275 EM

278.250

3 -0.75125072D+04 37.8197598 0.0050090 42.196 367.755 EM

292.049

4 -0.74958550D+04 16.6521595 0.0022166 45.457 353.445 EM

303.099

5 -0.74887154D+04 7.1396062 0.0009525 48.281 342.385 EM

311.334

6 -0.74855364D+04 3.1790603 0.0004245 50.729 333.813 EM

317.457

7 -0.74839294D+04 1.6069986 0.0002147 52.935 327.030 EM

322.035

8 -0.74830340D+04 0.8954007 0.0001196 54.958 321.574 EM

325.467

TECHNICAL 8 OUTPUT FOR STARTING VALUE SET 142

ITER LOGLIKELIHOOD ABS CHANGE REL CHANGE CLASS COUNTS ALGORITHM

1 -0.14616824D+05 0.0000000 0.0000000 6.469 660.037 EM

35.493

2 -0.78200041D+04 6796.8195203 0.4649998 6.744 655.006 EM

40.250

3 -0.77651714D+04 54.8327562 0.0070119 7.083 639.954 EM

54.963

4 -0.77454372D+04 19.7341738 0.0025414 7.519 624.496 EM

69.986

5 -0.77187385D+04 26.6987440 0.0034470 7.752 601.298 EM

92.950

6 -0.76696873D+04 49.0511568 0.0063548 7.581 571.697 EM

122.722

7 -0.76242549D+04 45.4323674 0.0059236 7.076 542.543 EM

152.380

8 -0.75962398D+04 28.0151489 0.0036745 6.470 515.855 EM

179.675

9 -0.75768960D+04 19.3437815 0.0025465 5.930 491.267 EM

204.803

10 -0.75614671D+04 15.4288962 0.0020363 5.525 468.582 EM

227.893

11 -0.75489508D+04 12.5163679 0.0016553 5.261 447.819 EM

248.921

12 -0.75389460D+04 10.0047093 0.0013253 5.110 428.952 EM

267.938

13 -0.75309571D+04 7.9889078 0.0010597 5.039 411.951 EM

285.009

14 -0.75245790D+04 6.3781871 0.0008469 5.023 396.872 EM

300.105

15 -0.75195988D+04 4.9801102 0.0006618 5.042 383.825 EM

313.134

16 -0.75158896D+04 3.7091948 0.0004933 5.083 372.864 EM

324.054

17 -0.75132839D+04 2.6057806 0.0003467 5.137 363.911 EM

332.952

18 -0.75115508D+04 1.7330455 0.0002307 5.200 356.765 EM

340.035

19 -0.75104470D+04 1.1038582 0.0001470 5.265 351.156 EM

345.579

20 -0.75097651D+04 0.6818406 0.0000908 5.329 346.802 EM

349.869

TECHNICAL 8 OUTPUT FOR STARTING VALUE SET 143

ITER LOGLIKELIHOOD ABS CHANGE REL CHANGE CLASS COUNTS ALGORITHM

1 -0.23724123D+05 0.0000000 0.0000000 77.278 616.794 EM

7.928

2 -0.77134326D+04 ************ 0.6748696 101.754 595.984 EM

4.263

3 -0.76813541D+04 32.0784915 0.0041588 128.225 566.925 EM

6.850

4 -0.76258894D+04 55.4647081 0.0072207 154.885 523.889 EM

23.225

5 -0.75601173D+04 65.7721313 0.0086248 179.912 481.179 EM

40.909

6 -0.75099406D+04 50.1766907 0.0066370 203.428 441.466 EM

57.106

7 -0.74683966D+04 41.5440275 0.0055319 222.422 408.764 EM

70.814

8 -0.74452784D+04 23.1181836 0.0030955 235.298 385.759 EM

80.943

9 -0.74356136D+04 9.6648297 0.0012981 243.272 370.739 EM

87.989

10 -0.74318146D+04 3.7989865 0.0005109 247.927 361.186 EM

92.887

11 -0.74303060D+04 1.5085602 0.0002030 250.487 355.154 EM

96.359

12 -0.74296792D+04 0.6268580 0.0000844 251.777 351.329 EM

98.893

TECHNICAL 8 OUTPUT FOR STARTING VALUE SET 144

ITER LOGLIKELIHOOD ABS CHANGE REL CHANGE CLASS COUNTS ALGORITHM

1 -0.28115393D+05 0.0000000 0.0000000 219.495 42.382 EM

440.123

2 -0.75847856D+04 ************ 0.7302266 232.193 48.936 EM

420.871

3 -0.75191715D+04 65.6141568 0.0086508 236.149 58.034 EM

407.817

4 -0.75043981D+04 14.7733224 0.0019648 238.466 63.563 EM

399.971

5 -0.74979651D+04 6.4330044 0.0008572 239.830 67.736 EM

394.434

6 -0.74939746D+04 3.9904823 0.0005322 240.538 71.682 EM

389.779

7 -0.74915296D+04 2.4449912 0.0003263 240.823 75.539 EM

385.639

8 -0.74900163D+04 1.5133316 0.0002020 240.733 79.265 EM

382.001

9 -0.74890354D+04 0.9809431 0.0001310 240.273 82.842 EM

378.884

TECHNICAL 8 OUTPUT FOR STARTING VALUE SET 145

ITER LOGLIKELIHOOD ABS CHANGE REL CHANGE CLASS COUNTS ALGORITHM

1 -0.21443376D+05 0.0000000 0.0000000 283.100 310.032 EM

108.868

2 -0.77417681D+04 ************ 0.6389669 290.004 306.705 EM

105.292

3 -0.76094111D+04 132.3570118 0.0170965 290.038 297.005 EM

114.957

4 -0.75361875D+04 73.2236103 0.0096228 283.636 296.982 EM

121.382

5 -0.74784448D+04 57.7427442 0.0076621 275.927 303.285 EM

122.788

6 -0.74447077D+04 33.7370694 0.0045112 269.581 310.837 EM

121.581

7 -0.74341831D+04 10.5245398 0.0014137 264.665 317.255 EM

120.081

8 -0.74314469D+04 2.7362382 0.0003681 260.732 322.336 EM

118.931

9 -0.74304443D+04 1.0026389 0.0001349 257.569 326.300 EM

118.132

10 -0.74299157D+04 0.5285881 0.0000711 255.030 329.390 EM

117.580

TECHNICAL 8 OUTPUT FOR STARTING VALUE SET 146

ITER LOGLIKELIHOOD ABS CHANGE REL CHANGE CLASS COUNTS ALGORITHM

1 -0.19454588D+05 0.0000000 0.0000000 381.728 190.319 EM

129.953

2 -0.77354499D+04 ************ 0.6023843 353.267 209.727 EM

139.007

3 -0.75706295D+04 164.8203704 0.0213071 321.905 236.994 EM

143.101

4 -0.75002180D+04 70.4114758 0.0093006 303.680 255.876 EM

142.444

5 -0.74684659D+04 31.7521397 0.0042335 295.968 265.819 EM

140.213

6 -0.74507138D+04 17.7521080 0.0023769 295.966 268.525 EM

137.509

7 -0.74410473D+04 9.6664478 0.0012974 300.731 266.887 EM

134.382

8 -0.74357043D+04 5.3429897 0.0007180 307.251 263.435 EM

131.314

9 -0.74327375D+04 2.9668215 0.0003990 313.659 259.651 EM

128.690

10 -0.74311274D+04 1.6101016 0.0002166 319.178 256.213 EM

126.609

11 -0.74302542D+04 0.8732099 0.0001175 323.638 253.350 EM

125.013

TECHNICAL 8 OUTPUT FOR STARTING VALUE SET 147

ITER LOGLIKELIHOOD ABS CHANGE REL CHANGE CLASS COUNTS ALGORITHM

1 -0.16055518D+05 0.0000000 0.0000000 3.425 58.523 EM

640.053

2 -0.77825418D+04 8272.9763387 0.5152731 3.441 70.176 EM

628.383

3 -0.77307949D+04 51.7468220 0.0066491 3.897 87.185 EM

610.918

4 -0.77001274D+04 30.6675272 0.0039669 4.413 106.048 EM

591.539

5 -0.76756865D+04 24.4408882 0.0031741 4.804 127.918 EM

569.278

6 -0.76512673D+04 24.4192128 0.0031814 5.099 152.314 EM

544.587

7 -0.76247610D+04 26.5063470 0.0034643 5.312 179.246 EM

517.442

8 -0.75934113D+04 31.3496266 0.0041116 5.471 208.309 EM

488.219

9 -0.75607181D+04 32.6932847 0.0043055 5.605 236.003 EM

460.392

10 -0.75376202D+04 23.0978063 0.0030550 5.728 259.232 EM

437.041

11 -0.75247049D+04 12.9153067 0.0017134 5.834 277.543 EM

418.623

12 -0.75175767D+04 7.1282112 0.0009473 5.916 291.462 EM

404.622

13 -0.75136469D+04 3.9298287 0.0005228 5.972 301.840 EM

394.189

14 -0.75114870D+04 2.1598535 0.0002875 6.003 309.530 EM

386.466

15 -0.75102931D+04 1.1939059 0.0001589 6.016 315.235 EM

380.749

16 -0.75096267D+04 0.6664335 0.0000887 6.014 319.484 EM

376.502

TECHNICAL 8 OUTPUT FOR STARTING VALUE SET 148

ITER LOGLIKELIHOOD ABS CHANGE REL CHANGE CLASS COUNTS ALGORITHM

1 -0.16713141D+05 0.0000000 0.0000000 556.266 52.033 EM

93.701

2 -0.77314615D+04 8981.6798833 0.5374023 544.680 48.815 EM

108.506

3 -0.76226983D+04 108.7632120 0.0140676 516.702 58.265 EM

127.033

4 -0.75618618D+04 60.8364514 0.0079810 492.810 65.798 EM

143.392

5 -0.75370727D+04 24.7891481 0.0032782 471.943 72.107 EM

157.950

6 -0.75220043D+04 15.0683196 0.0019992 451.613 78.828 EM

171.559

7 -0.75102442D+04 11.7601607 0.0015634 431.636 86.219 EM

184.145

8 -0.75004459D+04 9.7982343 0.0013046 412.578 94.186 EM

195.236

9 -0.74922845D+04 8.1614716 0.0010881 395.101 102.560 EM

204.339

10 -0.74855099D+04 6.7745270 0.0009042 379.602 111.237 EM

211.162

11 -0.74796425D+04 5.8674286 0.0007838 366.064 120.283 EM

215.653

12 -0.74739804D+04 5.6620844 0.0007570 354.165 129.928 EM

217.907

13 -0.74677988D+04 6.1816488 0.0008271 343.579 140.351 EM

218.071

14 -0.74608546D+04 6.9441307 0.0009299 334.380 151.274 EM

216.346

15 -0.74539849D+04 6.8697434 0.0009208 327.046 161.856 EM

213.098

16 -0.74484307D+04 5.5541796 0.0007451 321.775 171.333 EM

208.892

17 -0.74444759D+04 3.9548494 0.0005310 318.265 179.449 EM

204.286

18 -0.74417030D+04 2.7728258 0.0003725 316.074 186.281 EM

199.645

19 -0.74396844D+04 2.0186881 0.0002713 314.829 192.024 EM

195.146

20 -0.74381477D+04 1.5366434 0.0002065 314.259 196.878 EM

190.862

21 -0.74369341D+04 1.2136089 0.0001632 314.170 201.018 EM

186.812

22 -0.74359482D+04 0.9858992 0.0001326 314.421 204.586 EM

182.993

TECHNICAL 8 OUTPUT FOR STARTING VALUE SET 149

ITER LOGLIKELIHOOD ABS CHANGE REL CHANGE CLASS COUNTS ALGORITHM

1 -0.20556456D+05 0.0000000 0.0000000 154.686 62.567 EM

484.747

2 -0.78868265D+04 ************ 0.6163334 152.162 55.016 EM

494.822

3 -0.77808653D+04 105.9611759 0.0134352 161.745 68.277 EM

471.978

4 -0.76658759D+04 114.9894002 0.0147785 174.017 96.860 EM

431.123

5 -0.75826198D+04 83.2560823 0.0108606 179.737 121.908 EM

400.354

6 -0.75363557D+04 46.2641321 0.0061013 180.369 139.148 EM

382.483

7 -0.75051208D+04 31.2348340 0.0041446 181.042 150.082 EM

370.876

8 -0.74797039D+04 25.4169852 0.0033866 185.386 156.610 EM

360.004

9 -0.74569080D+04 22.7958397 0.0030477 193.087 159.896 EM

349.017

10 -0.74417901D+04 15.1179018 0.0020274 200.845 160.815 EM

340.341

11 -0.74355418D+04 6.2483468 0.0008396 207.179 160.396 EM

334.425

12 -0.74333228D+04 2.2189579 0.0002984 212.126 159.310 EM

330.564

13 -0.74323782D+04 0.9445980 0.0001271 216.001 157.868 EM

328.130

TECHNICAL 8 OUTPUT FOR STARTING VALUE SET 150

ITER LOGLIKELIHOOD ABS CHANGE REL CHANGE CLASS COUNTS ALGORITHM

1 -0.22340245D+05 0.0000000 0.0000000 212.607 442.043 EM

47.350

2 -0.76251013D+04 ************ 0.6586832 227.376 422.411 EM

52.213

3 -0.75611604D+04 63.9408820 0.0083856 244.208 404.094 EM

53.697

4 -0.75311562D+04 30.0041878 0.0039682 260.604 389.098 EM

52.299

5 -0.75149174D+04 16.2388850 0.0021562 274.526 378.131 EM

49.343

6 -0.75075064D+04 7.4109043 0.0009862 285.584 370.522 EM

45.894

7 -0.75036122D+04 3.8942293 0.0005187 294.050 365.505 EM

42.444

8 -0.75011400D+04 2.4722602 0.0003295 300.399 362.435 EM

39.167

9 -0.74993193D+04 1.8206359 0.0002427 305.089 360.785 EM

36.126

10 -0.74978008D+04 1.5184916 0.0002025 308.497 360.128 EM

33.376

11 -0.74964166D+04 1.3842459 0.0001846 310.915 360.091 EM

30.994

12 -0.74950914D+04 1.3251933 0.0001768 312.562 360.327 EM

29.110

13 -0.74937773D+04 1.3140892 0.0001753 313.592 360.452 EM

27.956

14 -0.74923056D+04 1.4717013 0.0001964 314.078 359.954 EM

27.968

15 -0.74897983D+04 2.5072583 0.0003346 313.954 357.996 EM

30.049

16 -0.74833370D+04 6.4613403 0.0008627 312.859 353.654 EM

35.487

17 -0.74704515D+04 12.8855181 0.0017219 310.164 347.849 EM

43.988

18 -0.74566840D+04 13.7675273 0.0018429 305.857 342.898 EM

53.245

19 -0.74472337D+04 9.4502913 0.0012674 300.696 340.101 EM

61.203

20 -0.74421083D+04 5.1253863 0.0006882 295.407 339.060 EM

67.533

21 -0.74390962D+04 3.0120974 0.0004047 290.331 338.955 EM

72.714

22 -0.74369560D+04 2.1401413 0.0002877 285.552 339.347 EM

77.101

23 -0.74352773D+04 1.6787013 0.0002257 281.090 340.017 EM

80.894

24 -0.74339144D+04 1.3628926 0.0001833 276.964 340.824 EM

84.212

25 -0.74328011D+04 1.1133082 0.0001498 273.199 341.665 EM

87.136

26 -0.74319000D+04 0.9011655 0.0001212 269.813 342.462 EM

89.725

TECHNICAL 8 OUTPUT FOR STARTING VALUE SET 151

ITER LOGLIKELIHOOD ABS CHANGE REL CHANGE CLASS COUNTS ALGORITHM

1 -0.26324722D+05 0.0000000 0.0000000 34.639 599.658 EM

67.702

2 -0.77020691D+04 ************ 0.7074207 25.164 578.093 EM

98.742

3 -0.76480082D+04 54.0608599 0.0070190 21.741 551.952 EM

128.307

4 -0.76181800D+04 29.8282913 0.0039001 19.896 526.305 EM

155.799

5 -0.75956603D+04 22.5196976 0.0029560 18.657 501.494 EM

181.849

6 -0.75769396D+04 18.7206582 0.0024647 17.886 477.646 EM

206.469

7 -0.75606204D+04 16.3191975 0.0021538 17.850 454.872 EM

229.278

8 -0.75463556D+04 14.2648480 0.0018867 18.868 433.344 EM

249.787

9 -0.75343609D+04 11.9946161 0.0015895 20.922 413.453 EM

267.624

10 -0.75248531D+04 9.5077999 0.0012619 23.760 395.654 EM

282.586

11 -0.75176481D+04 7.2050164 0.0009575 27.198 380.198 EM

294.605

12 -0.75122784D+04 5.3696814 0.0007143 31.156 367.076 EM

303.769

13 -0.75082636D+04 4.0148461 0.0005344 35.574 356.095 EM

310.332

14 -0.75052099D+04 3.0537295 0.0004067 40.380 346.979 EM

314.641

15 -0.75028265D+04 2.3834095 0.0003176 45.497 339.445 EM

317.058

16 -0.75009058D+04 1.9206949 0.0002560 50.864 333.228 EM

317.908

17 -0.74992941D+04 1.6116808 0.0002149 56.445 328.092 EM

317.463

18 -0.74978722D+04 1.4218322 0.0001896 62.237 323.826 EM

315.937

19 -0.74965460D+04 1.3262895 0.0001769 68.264 320.240 EM

313.496

20 -0.74952391D+04 1.3068856 0.0001743 74.572 317.171 EM

310.256

21 -0.74938866D+04 1.3524268 0.0001804 81.228 314.477 EM

306.295

22 -0.74924276D+04 1.4590896 0.0001947 88.316 312.034 EM

301.650

23 -0.74907976D+04 1.6300038 0.0002176 95.941 309.729 EM

296.329

24 -0.74889244D+04 1.8731812 0.0002501 104.227 307.461 EM

290.312

25 -0.74867277D+04 2.1966247 0.0002933 113.309 305.125 EM

283.566

26 -0.74841296D+04 2.5981947 0.0003470 123.324 302.611 EM

276.065

27 -0.74810772D+04 3.0523386 0.0004078 134.373 299.802 EM

267.826

28 -0.74775714D+04 3.5057804 0.0004686 146.474 296.591 EM

258.935

29 -0.74736751D+04 3.8962987 0.0005211 159.528 292.910 EM

249.562

30 -0.74694929D+04 4.1821905 0.0005596 173.338 288.734 EM

239.928

31 -0.74651370D+04 4.3559425 0.0005832 187.666 284.081 EM

230.253

32 -0.74607000D+04 4.4369757 0.0005944 202.286 278.994 EM

220.721

33 -0.74562487D+04 4.4512790 0.0005966 217.001 273.538 EM

211.461

34 -0.74518425D+04 4.4062558 0.0005909 231.613 267.813 EM

202.574

35 -0.74406874D+04 11.1551203 0.0014970 293.405 247.920 FS

160.674

36 -0.74379663D+04 2.7211184 0.0003657 304.679 241.269 FS

156.052

37 -0.74322386D+04 5.7276775 0.0007701 309.750 239.580 EM

152.670

38 -0.74313728D+04 0.8658024 0.0001165 314.204 238.066 EM

149.730

TECHNICAL 8 OUTPUT FOR STARTING VALUE SET 152

ITER LOGLIKELIHOOD ABS CHANGE REL CHANGE CLASS COUNTS ALGORITHM

1 -0.18147612D+05 0.0000000 0.0000000 44.407 595.191 EM

62.402

2 -0.77371416D+04 ************ 0.5736551 56.045 568.605 EM

77.350

3 -0.76160146D+04 121.1270182 0.0156553 68.441 531.881 EM

101.678

4 -0.75621552D+04 53.8593689 0.0070719 79.153 500.701 EM

122.146

5 -0.75351666D+04 26.9885975 0.0035689 89.481 473.707 EM

138.812

6 -0.75178294D+04 17.3372481 0.0023008 99.983 449.116 EM

152.901

7 -0.75039730D+04 13.8563792 0.0018431 110.875 426.237 EM

164.889

8 -0.74917330D+04 12.2400085 0.0016311 122.304 404.920 EM

174.777

9 -0.74802101D+04 11.5229461 0.0015381 134.546 385.103 EM

182.351

10 -0.74686246D+04 11.5854361 0.0015488 147.708 366.924 EM

187.368

11 -0.74572276D+04 11.3969913 0.0015260 160.938 351.317 EM

189.745

12 -0.74481455D+04 9.0821672 0.0012179 172.761 339.416 EM

189.824

13 -0.74425216D+04 5.6238350 0.0007551 182.488 331.174 EM

188.338

14 -0.74393185D+04 3.2030816 0.0004304 190.263 325.756 EM

185.981

15 -0.74373746D+04 1.9439117 0.0002613 196.479 322.319 EM

183.201

16 -0.74360679D+04 1.3067625 0.0001757 201.509 320.246 EM

180.245

17 -0.74351098D+04 0.9580937 0.0001288 205.643 319.108 EM

177.249

TECHNICAL 8 OUTPUT FOR STARTING VALUE SET 153

ITER LOGLIKELIHOOD ABS CHANGE REL CHANGE CLASS COUNTS ALGORITHM

1 -0.20617806D+05 0.0000000 0.0000000 287.748 263.840 EM

150.412

2 -0.76006414D+04 ************ 0.6313555 257.534 272.013 EM

172.453

3 -0.75006513D+04 99.9900908 0.0131555 241.949 279.378 EM

180.673

4 -0.74703855D+04 30.2657459 0.0040351 240.426 281.204 EM

180.369

5 -0.74574791D+04 12.9064517 0.0017277 247.028 279.159 EM

175.813

6 -0.74499384D+04 7.5406468 0.0010112 257.288 274.990 EM

169.722

7 -0.74442783D+04 5.6601277 0.0007598 268.799 269.793 EM

163.408

8 -0.74397843D+04 4.4939964 0.0006037 280.193 264.309 EM

157.498

9 -0.74363576D+04 3.4266628 0.0004606 290.637 259.070 EM

152.293

10 -0.74339157D+04 2.4419756 0.0003284 299.686 254.422 EM

147.892

11 -0.74322815D+04 1.6341345 0.0002198 307.198 250.537 EM

144.265

12 -0.74312343D+04 1.0472156 0.0001409 313.245 247.447 EM

141.308

13 -0.74305758D+04 0.6585268 0.0000886 318.017 245.089 EM

138.893

TECHNICAL 8 OUTPUT FOR STARTING VALUE SET 154

ITER LOGLIKELIHOOD ABS CHANGE REL CHANGE CLASS COUNTS ALGORITHM

1 -0.16827477D+05 0.0000000 0.0000000 0.603 1.549 EM

699.847

2 -0.78508515D+04 8976.6254223 0.5334505 1.288 2.137 EM

698.574

3 -0.78305341D+04 20.3174490 0.0025879 10.655 4.114 EM

687.230

4 -0.77869655D+04 43.5685580 0.0055639 24.015 8.755 EM

669.229

5 -0.77555785D+04 31.3870234 0.0040307 37.194 16.775 EM

648.031

6 -0.77278836D+04 27.6948757 0.0035710 49.546 29.361 EM

623.093

7 -0.76978633D+04 30.0202987 0.0038847 60.395 48.039 EM

593.567

8 -0.76601353D+04 37.7280732 0.0049011 69.709 73.253 EM

559.038

9 -0.76113325D+04 48.8027382 0.0063710 78.873 102.079 EM

521.048

10 -0.75622963D+04 49.0362165 0.0064425 89.203 126.842 EM

485.955

11 -0.75297926D+04 32.5036724 0.0042981 99.484 145.371 EM

457.146

12 -0.75106040D+04 19.1886596 0.0025484 108.342 159.361 EM

434.297

13 -0.74992352D+04 11.3687903 0.0015137 115.429 169.980 EM

416.591

14 -0.74925763D+04 6.6588906 0.0008879 120.875 178.069 EM

403.056

15 -0.74886587D+04 3.9175666 0.0005229 124.969 184.306 EM

392.725

16 -0.74862940D+04 2.3646922 0.0003158 128.002 189.213 EM

384.784

17 -0.74848196D+04 1.4743873 0.0001969 130.222 193.160 EM

378.618

18 -0.74838760D+04 0.9436592 0.0001261 131.818 196.401 EM

373.781

TECHNICAL 8 OUTPUT FOR STARTING VALUE SET 155

ITER LOGLIKELIHOOD ABS CHANGE REL CHANGE CLASS COUNTS ALGORITHM

1 -0.18520919D+05 0.0000000 0.0000000 463.186 78.077 EM

160.737

2 -0.76119585D+04 ************ 0.5890075 443.786 76.934 EM

181.280

3 -0.75574879D+04 54.4705838 0.0071559 424.440 80.992 EM

196.568

4 -0.75368138D+04 20.6740909 0.0027356 406.643 84.482 EM

210.875

5 -0.75199356D+04 16.8782326 0.0022394 389.543 87.509 EM

224.948

6 -0.75010768D+04 18.8587717 0.0025078 372.702 90.025 EM

239.273

7 -0.74825214D+04 18.5553583 0.0024737 356.277 91.479 EM

254.244

8 -0.74679294D+04 14.5920711 0.0019502 341.043 91.946 EM

269.011

9 -0.74572011D+04 10.7282867 0.0014366 327.589 92.059 EM

282.352

10 -0.74491527D+04 8.0484268 0.0010793 315.960 92.230 EM

293.810

11 -0.74433619D+04 5.7907165 0.0007774 305.981 92.591 EM

303.428

12 -0.74394617D+04 3.9002619 0.0005240 297.470 93.186 EM

311.344

13 -0.74368723D+04 2.5894116 0.0003481 290.235 94.015 EM

317.750

14 -0.74350714D+04 1.8008467 0.0002422 284.062 95.036 EM

322.902

15 -0.74337283D+04 1.3431625 0.0001807 278.753 96.182 EM

327.065

16 -0.74326671D+04 1.0612019 0.0001428 274.153 97.387 EM

330.459

17 -0.74318026D+04 0.8644515 0.0001163 270.153 98.599 EM

333.248

TECHNICAL 8 OUTPUT FOR STARTING VALUE SET 156

ITER LOGLIKELIHOOD ABS CHANGE REL CHANGE CLASS COUNTS ALGORITHM

1 -0.19610889D+05 0.0000000 0.0000000 327.115 182.634 EM

192.251

2 -0.78393224D+04 ************ 0.6002567 329.673 183.138 EM

189.189

3 -0.77488853D+04 90.4371102 0.0115363 329.125 185.613 EM

187.262

4 -0.76625642D+04 86.3210250 0.0111398 326.736 184.071 EM

191.192

5 -0.75669217D+04 95.6424960 0.0124818 326.516 178.184 EM

197.300

6 -0.75166151D+04 50.3066660 0.0066482 327.623 173.944 EM

200.433

7 -0.74992789D+04 17.3361959 0.0023064 328.583 172.053 EM

201.364

8 -0.74937015D+04 5.5773654 0.0007437 329.048 171.920 EM

201.032

9 -0.74912836D+04 2.4178989 0.0003227 329.052 173.068 EM

199.879

10 -0.74896748D+04 1.6088433 0.0002148 328.668 175.181 EM

198.151

11 -0.74881788D+04 1.4959524 0.0001997 327.924 178.089 EM

195.987

12 -0.74864660D+04 1.7128672 0.0002287 326.789 181.739 EM

193.472

13 -0.74842512D+04 2.2147169 0.0002958 325.159 186.185 EM

190.655

14 -0.74812116D+04 3.0396458 0.0004061 322.852 191.593 EM

187.555

15 -0.74770465D+04 4.1650578 0.0005567 319.623 198.234 EM

184.143

16 -0.74716820D+04 5.3645003 0.0007175 315.250 206.400 EM

180.350

17 -0.74654723D+04 6.2097185 0.0008311 309.648 216.268 EM

176.083

18 -0.74591166D+04 6.3556785 0.0008513 302.962 227.771 EM

171.267

19 -0.74532161D+04 5.9005909 0.0007911 295.545 240.495 EM

165.960

20 -0.74479670D+04 5.2490998 0.0007043 287.836 253.772 EM

160.392

21 -0.74433692D+04 4.5977397 0.0006173 280.246 266.871 EM

154.884

22 -0.74394773D+04 3.8919223 0.0005229 273.116 279.140 EM

149.743

23 -0.74363777D+04 3.0995595 0.0004166 266.706 290.096 EM

145.198

24 -0.74340737D+04 2.3039966 0.0003098 261.169 299.479 EM

141.352

25 -0.74324566D+04 1.6171486 0.0002175 256.554 307.250 EM

138.196

26 -0.74313623D+04 1.0942803 0.0001472 252.827 313.525 EM

135.649

27 -0.74306345D+04 0.7278165 0.0000979 249.897 318.506 EM

133.597

TECHNICAL 8 OUTPUT FOR STARTING VALUE SET 157

ITER LOGLIKELIHOOD ABS CHANGE REL CHANGE CLASS COUNTS ALGORITHM

1 -0.18617561D+05 0.0000000 0.0000000 225.798 466.696 EM

9.506

2 -0.78472736D+04 ************ 0.5785015 225.497 470.788 EM

5.715

3 -0.77681137D+04 79.1599010 0.0100876 232.141 462.690 EM

7.169

4 -0.76992138D+04 68.8998553 0.0088696 239.772 443.962 EM

18.266

5 -0.76325559D+04 66.6579623 0.0086578 243.132 425.879 EM

32.989

6 -0.75852603D+04 47.2955916 0.0061966 243.089 413.271 EM

45.640

7 -0.75355626D+04 49.6976288 0.0065519 246.180 398.445 EM

57.375

8 -0.74815862D+04 53.9764682 0.0071629 252.699 381.523 EM

67.778

9 -0.74475558D+04 34.0303877 0.0045486 258.212 367.645 EM

76.143

10 -0.74363628D+04 11.1929906 0.0015029 261.226 358.368 EM

82.406

11 -0.74328597D+04 3.5031016 0.0004711 262.185 352.757 EM

87.057

12 -0.74314004D+04 1.4593149 0.0001963 261.899 349.501 EM

90.600

13 -0.74306271D+04 0.7732515 0.0001041 260.974 347.639 EM

93.387

TECHNICAL 8 OUTPUT FOR STARTING VALUE SET 158

ITER LOGLIKELIHOOD ABS CHANGE REL CHANGE CLASS COUNTS ALGORITHM

1 -0.18376961D+05 0.0000000 0.0000000 55.142 629.451 EM

17.408

2 -0.76821569D+04 ************ 0.5819681 69.808 595.119 EM

37.073

3 -0.76157339D+04 66.4230605 0.0086464 86.398 555.896 EM

59.706

4 -0.75730677D+04 42.6662051 0.0056024 102.123 518.977 EM

80.900

5 -0.75425157D+04 30.5519590 0.0040343 117.188 486.011 EM

98.801

6 -0.75184887D+04 24.0270288 0.0031855 132.157 456.163 EM

113.680

7 -0.74969302D+04 21.5584492 0.0028674 147.985 428.156 EM

125.859

8 -0.74752024D+04 21.7278136 0.0028982 165.071 401.544 EM

135.385

9 -0.74549657D+04 20.2366853 0.0027072 181.332 378.568 EM

142.100

10 -0.74420221D+04 12.9436619 0.0017362 194.449 361.379 EM

146.172

11 -0.74359787D+04 6.0433645 0.0008121 204.210 349.539 EM

148.250

12 -0.74333004D+04 2.6783002 0.0003602 211.356 341.651 EM

148.993

13 -0.74320224D+04 1.2780196 0.0001719 216.626 336.497 EM

148.876

14 -0.74313480D+04 0.6744267 0.0000907 220.579 333.206 EM

148.215

TECHNICAL 8 OUTPUT FOR STARTING VALUE SET 159

ITER LOGLIKELIHOOD ABS CHANGE REL CHANGE CLASS COUNTS ALGORITHM

1 -0.23705097D+05 0.0000000 0.0000000 449.511 212.386 EM

40.103

2 -0.75641212D+04 ************ 0.6809074 424.982 224.059 EM

52.959

3 -0.75135821D+04 50.5391193 0.0066814 400.789 242.053 EM

59.158

4 -0.74933032D+04 20.2788694 0.0026990 379.171 259.936 EM

62.894

5 -0.74779717D+04 15.3314927 0.0020460 360.377 275.847 EM

65.777

6 -0.74656007D+04 12.3709886 0.0016543 344.368 289.255 EM

68.376

7 -0.74561485D+04 9.4521977 0.0012661 330.931 300.049 EM

71.021

8 -0.74493088D+04 6.8396647 0.0009173 319.693 308.511 EM

73.796

9 -0.74445173D+04 4.7915313 0.0006432 310.234 315.153 EM

76.612

10 -0.74411576D+04 3.3596686 0.0004513 302.188 320.431 EM

79.381

11 -0.74387038D+04 2.4538089 0.0003298 295.260 324.683 EM

82.058

12 -0.74368094D+04 1.8944398 0.0002547 289.215 328.168 EM

84.618

13 -0.74352790D+04 1.5304453 0.0002058 283.875 331.084 EM

87.040

14 -0.74340068D+04 1.2721873 0.0001711 279.119 333.569 EM

89.312

15 -0.74329362D+04 1.0705885 0.0001440 274.868 335.707 EM

91.425

16 -0.74320377D+04 0.8984894 0.0001209 271.081 337.542 EM

93.376

TECHNICAL 8 OUTPUT FOR STARTING VALUE SET 160

ITER LOGLIKELIHOOD ABS CHANGE REL CHANGE CLASS COUNTS ALGORITHM

1 -0.24151886D+05 0.0000000 0.0000000 49.263 122.268 EM

530.469

2 -0.75992039D+04 ************ 0.6853578 52.655 157.342 EM

492.003

3 -0.75410238D+04 58.1800016 0.0076561 58.620 183.927 EM

459.453

4 -0.75157932D+04 25.2306903 0.0033458 64.432 203.521 EM

434.047

5 -0.75019941D+04 13.7990650 0.0018360 69.387 218.072 EM

414.542

6 -0.74939865D+04 8.0075655 0.0010674 73.398 228.921 EM

399.681

7 -0.74892411D+04 4.7454405 0.0006332 76.574 237.096 EM

388.330

8 -0.74863554D+04 2.8857237 0.0003853 79.075 243.349 EM

379.576

9 -0.74845496D+04 1.8057852 0.0002412 81.048 248.207 EM

372.745

10 -0.74833924D+04 1.1572228 0.0001546 82.612 252.034 EM

367.354

11 -0.74826371D+04 0.7552169 0.0001009 83.859 255.085 EM

363.056

TECHNICAL 8 OUTPUT FOR STARTING VALUE SET 161

ITER LOGLIKELIHOOD ABS CHANGE REL CHANGE CLASS COUNTS ALGORITHM

1 -0.19725612D+05 0.0000000 0.0000000 431.395 248.311 EM

22.295

2 -0.77039139D+04 ************ 0.6094461 421.089 257.258 EM

23.653

3 -0.76186883D+04 85.2255937 0.0110626 402.446 267.825 EM

31.730

4 -0.75620553D+04 56.6329845 0.0074334 383.247 278.665 EM

40.088

5 -0.75142737D+04 47.7816489 0.0063186 368.913 286.488 EM

46.599

6 -0.74807162D+04 33.5574806 0.0044658 357.824 289.946 EM

54.230

7 -0.74565751D+04 24.1410423 0.0032271 349.664 289.552 EM

62.784

8 -0.74435717D+04 13.0034044 0.0017439 344.998 286.904 EM

70.097

9 -0.74379336D+04 5.6381169 0.0007574 342.982 283.370 EM

75.648

10 -0.74354439D+04 2.4897503 0.0003347 342.333 279.642 EM

80.025

11 -0.74339193D+04 1.5245828 0.0002050 342.347 275.990 EM

83.663

12 -0.74327759D+04 1.1434082 0.0001538 342.688 272.536 EM

86.777

13 -0.74318725D+04 0.9033370 0.0001215 343.160 269.357 EM

89.483

TECHNICAL 8 OUTPUT FOR STARTING VALUE SET 162

ITER LOGLIKELIHOOD ABS CHANGE REL CHANGE CLASS COUNTS ALGORITHM

1 -0.27906857D+05 0.0000000 0.0000000 523.768 156.602 EM

21.631

2 -0.77644830D+04 ************ 0.7217715 517.117 161.390 EM

23.492

3 -0.76378349D+04 126.6480925 0.0163112 483.407 178.919 EM

39.674

4 -0.75608108D+04 77.0241325 0.0100846 448.899 197.238 EM

55.863

5 -0.75333080D+04 27.5028125 0.0036375 421.382 213.748 EM

66.870

6 -0.75216044D+04 11.7035591 0.0015536 399.242 228.104 EM

74.654

7 -0.75145371D+04 7.0673271 0.0009396 381.119 240.148 EM

80.734

8 -0.75098615D+04 4.6756260 0.0006222 366.231 249.904 EM

85.866

9 -0.75067115D+04 3.1499567 0.0004194 353.968 257.533 EM

90.500

10 -0.75045024D+04 2.2090668 0.0002943 343.783 263.240 EM

94.977

11 -0.75027673D+04 1.7351541 0.0002312 335.179 267.197 EM

99.624

12 -0.75010675D+04 1.6997974 0.0002266 327.706 269.481 EM

104.813

13 -0.74988665D+04 2.2009728 0.0002934 320.968 270.034 EM

110.998

14 -0.74954034D+04 3.4631267 0.0004618 314.731 268.640 EM

118.628

15 -0.74898284D+04 5.5749820 0.0007438 309.143 264.994 EM

127.864

16 -0.74818620D+04 7.9664426 0.0010636 304.722 258.944 EM

138.335

17 -0.74723333D+04 9.5286478 0.0012736 302.103 250.787 EM

149.110

18 -0.74630760D+04 9.2572590 0.0012389 301.592 241.342 EM

159.066

19 -0.74556272D+04 7.4488034 0.0009981 302.574 231.711 EM

167.715

20 -0.74501097D+04 5.5175726 0.0007401 304.118 222.727 EM

175.155

21 -0.74460632D+04 4.0464669 0.0005431 305.676 214.727 EM

181.597

22 -0.74430713D+04 2.9918934 0.0004018 307.080 207.713 EM

187.207

23 -0.74408232D+04 2.2481142 0.0003020 308.334 201.556 EM

192.109

24 -0.74390934D+04 1.7298276 0.0002325 309.491 196.100 EM

196.409

25 -0.74377260D+04 1.3673401 0.0001838 310.596 191.207 EM

200.197

26 -0.74366179D+04 1.1081199 0.0001490 311.677 186.771 EM

203.553

27 -0.74357012D+04 0.9167069 0.0001233 312.746 182.710 EM

206.544

TECHNICAL 8 OUTPUT FOR STARTING VALUE SET 163

ITER LOGLIKELIHOOD ABS CHANGE REL CHANGE CLASS COUNTS ALGORITHM

1 -0.17663310D+05 0.0000000 0.0000000 532.318 126.123 EM

43.559

2 -0.76083272D+04 ************ 0.5692581 485.371 155.959 EM

60.671

3 -0.75112266D+04 97.1006392 0.0127624 441.015 182.747 EM

78.238

4 -0.74657888D+04 45.4378367 0.0060493 407.477 203.266 EM

91.258

5 -0.74438261D+04 21.9626495 0.0029418 384.379 217.722 EM

99.900

6 -0.74348555D+04 8.9706450 0.0012051 369.020 227.478 EM

105.502

7 -0.74313108D+04 3.5446526 0.0004768 358.953 233.950 EM

109.097

8 -0.74298917D+04 1.4191415 0.0001910 352.416 238.206 EM

111.378

9 -0.74293169D+04 0.5748032 0.0000774 348.197 240.996 EM

112.807

TECHNICAL 8 OUTPUT FOR STARTING VALUE SET 164

ITER LOGLIKELIHOOD ABS CHANGE REL CHANGE CLASS COUNTS ALGORITHM

1 -0.21208355D+05 0.0000000 0.0000000 82.787 296.910 EM

322.303

2 -0.75454997D+04 ************ 0.6442204 70.869 304.086 EM

327.045

3 -0.75154605D+04 30.0391434 0.0039811 69.193 300.698 EM

332.109

4 -0.75103961D+04 5.0643830 0.0006739 70.103 296.412 EM

335.485

5 -0.75082696D+04 2.1264879 0.0002831 71.823 293.166 EM

337.011

6 -0.75064289D+04 1.8407965 0.0002452 74.044 291.188 EM

336.768

7 -0.75042247D+04 2.2041676 0.0002936 76.924 290.291 EM

334.785

8 -0.75015515D+04 2.6731399 0.0003562 80.690 290.177 EM

331.133

9 -0.74986964D+04 2.8551869 0.0003806 85.390 290.553 EM

326.056

10 -0.74960111D+04 2.6852110 0.0003581 90.909 291.161 EM

319.931

11 -0.74936101D+04 2.4010005 0.0003203 97.139 291.771 EM

313.091

12 -0.74913795D+04 2.2306137 0.0002977 104.071 292.201 EM

305.728

13 -0.74891310D+04 2.2485411 0.0003002 111.770 292.320 EM

297.910

14 -0.74866900D+04 2.4409755 0.0003259 120.339 292.038 EM

289.623

15 -0.74839161D+04 2.7739586 0.0003705 129.890 291.278 EM

280.832

16 -0.74807158D+04 3.2002754 0.0004276 140.515 289.961 EM

271.523

17 -0.74770684D+04 3.6473414 0.0004876 152.240 288.008 EM

261.752

18 -0.74730349D+04 4.0335247 0.0005395 164.974 285.366 EM

251.660

19 -0.74687261D+04 4.3088073 0.0005766 178.524 282.027 EM

241.448

20 -0.74642568D+04 4.4693143 0.0005984 192.648 278.034 EM

231.319

21 -0.74597191D+04 4.5377139 0.0006079 207.109 273.457 EM

221.435

22 -0.74551836D+04 4.5355104 0.0006080 221.689 268.398 EM

211.913

23 -0.74507245D+04 4.4590857 0.0005981 236.150 263.003 EM

202.847

24 -0.74464569D+04 4.2675709 0.0005728 250.188 257.476 EM

194.336

25 -0.74425531D+04 3.9037779 0.0005242 263.425 252.080 EM

186.494

26 -0.74392042D+04 3.3489527 0.0004500 275.467 247.096 EM

179.437

27 -0.74365400D+04 2.6641717 0.0003581 286.007 242.758 EM

173.235

28 -0.74345716D+04 1.9684476 0.0002647 294.900 239.210 EM

167.890

29 -0.74332005D+04 1.3710983 0.0001844 302.181 236.483 EM

163.337

30 -0.74322773D+04 0.9231736 0.0001242 308.010 234.521 EM

159.469

TECHNICAL 8 OUTPUT FOR STARTING VALUE SET 165

ITER LOGLIKELIHOOD ABS CHANGE REL CHANGE CLASS COUNTS ALGORITHM

1 -0.14056525D+05 0.0000000 0.0000000 9.760 656.810 EM

35.430

2 -0.77885340D+04 6267.9914726 0.4459133 16.591 632.714 EM

52.696

3 -0.77122511D+04 76.2829554 0.0097943 28.982 590.429 EM

82.588

4 -0.76411388D+04 71.1122402 0.0092207 40.804 545.715 EM

115.482

5 -0.75864496D+04 54.6892405 0.0071572 51.031 505.585 EM

145.384

6 -0.75477916D+04 38.6579747 0.0050957 59.922 472.081 EM

169.997

7 -0.75225239D+04 25.2677135 0.0033477 67.422 444.962 EM

189.616

8 -0.75066107D+04 15.9131614 0.0021154 73.539 423.564 EM

204.897

9 -0.74969071D+04 9.7036420 0.0012927 78.395 407.036 EM

216.569

10 -0.74910890D+04 5.8180750 0.0007761 82.181 394.377 EM

225.442

11 -0.74875634D+04 3.5256531 0.0004706 85.113 384.640 EM

232.246

12 -0.74853679D+04 2.1954820 0.0002932 87.382 377.073 EM

237.546

13 -0.74839635D+04 1.4044260 0.0001876 89.140 371.122 EM

241.738

14 -0.74830472D+04 0.9162852 0.0001224 90.502 366.394 EM

245.104

TECHNICAL 8 OUTPUT FOR STARTING VALUE SET 166

ITER LOGLIKELIHOOD ABS CHANGE REL CHANGE CLASS COUNTS ALGORITHM

1 -0.24907926D+05 0.0000000 0.0000000 151.278 17.329 EM

533.393

2 -0.78425449D+04 ************ 0.6851386 131.126 25.620 EM

545.255

3 -0.77712561D+04 71.2888087 0.0090900 122.481 37.071 EM

542.448

4 -0.77458725D+04 25.3835262 0.0032663 122.742 44.564 EM

534.694

5 -0.77320966D+04 13.7759395 0.0017785 124.362 55.778 EM

521.859

6 -0.76999899D+04 32.1066510 0.0041524 125.255 76.422 EM

500.323

7 -0.76451691D+04 54.8208650 0.0071196 127.549 102.155 EM

472.296

8 -0.75929786D+04 52.1904858 0.0068266 133.135 125.766 EM

443.099

9 -0.75528935D+04 40.0850609 0.0052792 142.029 144.670 EM

415.301

10 -0.75240504D+04 28.8431116 0.0038188 152.661 158.133 EM

391.207

11 -0.75046226D+04 19.4278520 0.0025821 163.899 166.574 EM

371.527

12 -0.74917801D+04 12.8424817 0.0017113 175.397 170.868 EM

355.734

13 -0.74828844D+04 8.8956949 0.0011874 186.966 172.009 EM

343.025

14 -0.74757121D+04 7.1723431 0.0009585 198.681 170.904 EM

332.415

15 -0.74689909D+04 6.7211166 0.0008991 210.787 168.240 EM

322.973

16 -0.74624118D+04 6.5791259 0.0008809 223.483 164.479 EM

314.038

17 -0.74561671D+04 6.2446620 0.0008368 236.764 159.948 EM

305.288

18 -0.74504662D+04 5.7009106 0.0007646 250.355 154.960 EM

296.685

19 -0.74454027D+04 5.0635630 0.0006796 263.769 149.861 EM

288.370

20 -0.74410657D+04 4.3369935 0.0005825 276.440 144.995 EM

280.565

21 -0.74375616D+04 3.5040974 0.0004709 287.874 140.636 EM

273.490

22 -0.74349188D+04 2.6427616 0.0003553 297.764 136.935 EM

267.301

23 -0.74330398D+04 1.8790555 0.0002527 306.024 133.918 EM

262.057

24 -0.74317516D+04 1.2881833 0.0001733 312.745 131.521 EM

257.734

25 -0.74308812D+04 0.8703834 0.0001171 318.114 129.635 EM

254.251

TECHNICAL 8 OUTPUT FOR STARTING VALUE SET 167

ITER LOGLIKELIHOOD ABS CHANGE REL CHANGE CLASS COUNTS ALGORITHM

1 -0.14968045D+05 0.0000000 0.0000000 558.047 141.528 EM

2.426

2 -0.76613094D+04 7306.7355630 0.4881556 532.121 168.622 EM

1.257

3 -0.76191007D+04 42.2087239 0.0055093 501.264 199.358 EM

1.378

4 -0.75849959D+04 34.1048071 0.0044762 470.238 229.333 EM

2.429

5 -0.75554765D+04 29.5193859 0.0038918 441.751 254.959 EM

5.290

6 -0.75241886D+04 31.2878573 0.0041411 413.382 274.704 EM

13.914

7 -0.74896862D+04 34.5024735 0.0045855 389.756 287.275 EM

24.969

8 -0.74697442D+04 19.9420073 0.0026626 372.676 293.502 EM

35.822

9 -0.74581731D+04 11.5710747 0.0015491 360.382 295.454 EM

46.164

10 -0.74497860D+04 8.3870503 0.0011245 351.895 294.621 EM

55.484

11 -0.74438442D+04 5.9418795 0.0007976 346.733 292.006 EM

63.261

12 -0.74399880D+04 3.8562042 0.0005180 344.051 288.414 EM

69.535

13 -0.74374111D+04 2.5768901 0.0003464 342.899 284.432 EM

74.669

14 -0.74355198D+04 1.8912173 0.0002543 342.616 280.406 EM

78.978

15 -0.74340480D+04 1.4718033 0.0001979 342.801 276.535 EM

82.664

16 -0.74328783D+04 1.1697494 0.0001574 343.209 272.931 EM

85.860

17 -0.74319497D+04 0.9286199 0.0001249 343.686 269.659 EM

88.655

TECHNICAL 8 OUTPUT FOR STARTING VALUE SET 168

ITER LOGLIKELIHOOD ABS CHANGE REL CHANGE CLASS COUNTS ALGORITHM

1 -0.19881775D+05 0.0000000 0.0000000 519.868 60.601 EM

121.531

2 -0.77421072D+04 ************ 0.6105928 506.735 84.858 EM

110.407

3 -0.76462681D+04 95.8390921 0.0123789 479.355 115.172 EM

107.473

4 -0.76004532D+04 45.8148703 0.0059918 451.057 143.672 EM

107.271

5 -0.75719547D+04 28.4985665 0.0037496 424.905 169.429 EM

107.666

6 -0.75512173D+04 20.7373098 0.0027387 401.366 192.294 EM

108.340

7 -0.75360100D+04 15.2073286 0.0020139 380.470 212.374 EM

109.156

8 -0.75249438D+04 11.0662550 0.0014685 362.165 229.927 EM

109.908

9 -0.75169339D+04 8.0099056 0.0010644 346.348 245.215 EM

110.437

10 -0.75111695D+04 5.7643269 0.0007668 332.836 258.484 EM

110.680

11 -0.75070328D+04 4.1366877 0.0005507 321.359 269.994 EM

110.646

12 -0.75040384D+04 2.9944427 0.0003989 311.588 280.029 EM

110.382

13 -0.75018014D+04 2.2370227 0.0002981 303.174 288.880 EM

109.946

14 -0.75000250D+04 1.7763354 0.0002368 295.779 296.821 EM

109.400

15 -0.74984960D+04 1.5289943 0.0002039 289.111 304.086 EM

108.803

16 -0.74970774D+04 1.4186075 0.0001892 282.933 310.853 EM

108.215

17 -0.74956973D+04 1.3801772 0.0001841 277.072 317.233 EM

107.694

18 -0.74943342D+04 1.3630302 0.0001818 271.427 323.278 EM

107.295

19 -0.74930020D+04 1.3321964 0.0001778 265.954 328.983 EM

107.064

20 -0.74917334D+04 1.2686151 0.0001693 260.653 334.310 EM

107.037

21 -0.74905657D+04 1.1677053 0.0001559 255.557 339.204 EM

107.239

22 -0.74895294D+04 1.0363169 0.0001383 250.704 343.612 EM

107.684

23 -0.74886412D+04 0.8881723 0.0001186 246.130 347.495 EM

108.376

TECHNICAL 8 OUTPUT FOR STARTING VALUE SET 169

ITER LOGLIKELIHOOD ABS CHANGE REL CHANGE CLASS COUNTS ALGORITHM

1 -0.33000004D+05 0.0000000 0.0000000 241.061 326.547 EM

134.392

2 -0.77572454D+04 ************ 0.7649320 238.517 323.174 EM

140.310

3 -0.76221559D+04 135.0895184 0.0174146 232.966 309.396 EM

159.638

4 -0.75697574D+04 52.3984398 0.0068745 224.798 298.433 EM

178.769

5 -0.75494923D+04 20.2651240 0.0026771 215.741 288.238 EM

198.021

6 -0.75358181D+04 13.6741567 0.0018113 206.412 278.339 EM

217.250

7 -0.75241909D+04 11.6272547 0.0015429 197.168 269.071 EM

235.761

8 -0.75141199D+04 10.0709824 0.0013385 188.277 260.736 EM

252.987

9 -0.75056588D+04 8.4611259 0.0011260 179.922 253.515 EM

268.563

10 -0.74988405D+04 6.8183099 0.0009084 172.248 247.503 EM

282.249

11 -0.74936225D+04 5.2179657 0.0006958 165.382 242.726 EM

293.892

12 -0.74898981D+04 3.7244229 0.0004970 159.375 239.163 EM

303.462

13 -0.74874218D+04 2.4762912 0.0003306 154.164 236.722 EM

311.114

14 -0.74858381D+04 1.5837434 0.0002115 149.623 235.233 EM

317.144

15 -0.74848157D+04 1.0223765 0.0001366 145.624 234.489 EM

321.887

16 -0.74841220D+04 0.6937181 0.0000927 142.059 234.292 EM

325.650

TECHNICAL 8 OUTPUT FOR STARTING VALUE SET 170

ITER LOGLIKELIHOOD ABS CHANGE REL CHANGE CLASS COUNTS ALGORITHM

1 -0.21718402D+05 0.0000000 0.0000000 191.944 402.185 EM

107.871

2 -0.77165875D+04 ************ 0.6446982 178.496 394.294 EM

129.210

3 -0.76003348D+04 116.2526509 0.0150653 169.978 376.817 EM

155.205

4 -0.75666638D+04 33.6710690 0.0044302 164.293 359.144 EM

178.563

5 -0.75489760D+04 17.6877259 0.0023376 159.089 342.393 EM

200.518

6 -0.75352013D+04 13.7747709 0.0018247 154.350 326.615 EM

221.036

7 -0.75240925D+04 11.1088210 0.0014743 150.206 311.943 EM

239.851

8 -0.75153491D+04 8.7434021 0.0011621 146.662 298.553 EM

256.786

9 -0.75086482D+04 6.7008305 0.0008916 143.645 286.531 EM

271.824

10 -0.75035672D+04 5.0810105 0.0006767 141.072 275.839 EM

285.089

11 -0.74996722D+04 3.8950033 0.0005191 138.879 266.339 EM

296.782

12 -0.74966002D+04 3.0719677 0.0004096 137.031 257.854 EM

307.115

13 -0.74940928D+04 2.5074839 0.0003345 135.512 250.222 EM

316.266

14 -0.74919931D+04 2.0996444 0.0002802 134.320 243.320 EM

324.359

15 -0.74902236D+04 1.7695377 0.0002362 133.463 237.071 EM

331.466

16 -0.74887532D+04 1.4703490 0.0001963 132.947 231.431 EM

337.622

17 -0.74875666D+04 1.1866450 0.0001585 132.776 226.367 EM

342.857

18 -0.74866420D+04 0.9245486 0.0001235 132.942 221.845 EM

347.213

TECHNICAL 8 OUTPUT FOR STARTING VALUE SET 171

ITER LOGLIKELIHOOD ABS CHANGE REL CHANGE CLASS COUNTS ALGORITHM

1 -0.18311649D+05 0.0000000 0.0000000 6.858 547.429 EM

147.714

2 -0.76673620D+04 ************ 0.5812850 6.081 521.502 EM

174.417

3 -0.75919601D+04 75.4019345 0.0098341 5.687 495.374 EM

200.939

4 -0.75668713D+04 25.0887668 0.0033046 5.340 471.861 EM

224.799

5 -0.75516994D+04 15.1719572 0.0020051 5.124 450.325 EM

246.551

6 -0.75403614D+04 11.3379928 0.0015014 5.023 430.684 EM

266.293

7 -0.75315921D+04 8.7692311 0.0011630 4.996 413.009 EM

283.994

8 -0.75247882D+04 6.8039796 0.0009034 5.012 397.419 EM

299.569

9 -0.75196073D+04 5.1809023 0.0006885 5.052 384.027 EM

312.920

10 -0.75158251D+04 3.7821898 0.0005030 5.106 372.859 EM

324.035

11 -0.75132086D+04 2.6164749 0.0003481 5.166 363.799 EM

333.035

12 -0.75114885D+04 1.7200791 0.0002289 5.229 356.609 EM

340.161

13 -0.75104022D+04 1.0862767 0.0001446 5.293 350.993 EM

345.715

14 -0.75097354D+04 0.6668585 0.0000888 5.353 346.650 EM

349.997

TECHNICAL 8 OUTPUT FOR STARTING VALUE SET 172

ITER LOGLIKELIHOOD ABS CHANGE REL CHANGE CLASS COUNTS ALGORITHM

1 -0.11300069D+05 0.0000000 0.0000000 28.924 673.076 EM

0.000

2 -0.77903635D+04 3509.7050733 0.3105915 36.278 665.722 EM

0.000

3 -0.77802363D+04 10.1272387 0.0013000 45.881 656.119 EM

0.000

4 -0.77650940D+04 15.1422286 0.0019462 61.698 640.302 EM

0.000

5 -0.77259679D+04 39.1260993 0.0050387 87.550 614.450 EM

0.000

6 -0.76734507D+04 52.5172695 0.0067975 117.794 584.205 EM

0.001

7 -0.76368960D+04 36.5546578 0.0047638 146.707 555.145 EM

0.147

8 -0.76064662D+04 30.4298381 0.0039846 173.682 524.406 EM

3.912

9 -0.75650986D+04 41.3675453 0.0054385 197.830 491.608 EM

12.562

10 -0.75359171D+04 29.1815794 0.0038574 217.971 462.851 EM

21.177

11 -0.75181226D+04 17.7944197 0.0023613 234.531 438.291 EM

29.178

12 -0.75067603D+04 11.3623095 0.0015113 248.106 417.718 EM

36.176

13 -0.74994162D+04 7.3441391 0.0009783 259.321 400.515 EM

42.164

14 -0.74945199D+04 4.8962840 0.0006529 268.709 386.005 EM

47.286

15 -0.74911387D+04 3.3812175 0.0004512 276.665 373.650 EM

51.686

16 -0.74887392D+04 2.3994791 0.0003203 283.471 363.053 EM

55.476

17 -0.74870043D+04 1.7348996 0.0002317 289.337 353.921 EM

58.742

18 -0.74857310D+04 1.2733208 0.0001701 294.424 346.025 EM

61.550

19 -0.74847818D+04 0.9491459 0.0001268 298.864 339.176 EM

63.960

TECHNICAL 8 OUTPUT FOR STARTING VALUE SET 173

ITER LOGLIKELIHOOD ABS CHANGE REL CHANGE CLASS COUNTS ALGORITHM

1 -0.18874853D+05 0.0000000 0.0000000 600.892 62.793 EM

38.315

2 -0.76859820D+04 ************ 0.5927925 578.674 71.321 EM

52.005

3 -0.76388174D+04 47.1645329 0.0061364 552.906 86.720 EM

62.374

4 -0.76125628D+04 26.2546607 0.0034370 527.644 103.693 EM

70.663

5 -0.75922881D+04 20.2747114 0.0026633 503.439 120.828 EM

77.733

6 -0.75752121D+04 17.0759980 0.0022491 480.698 137.455 EM

83.847

7 -0.75604436D+04 14.7684735 0.0019496 459.616 153.031 EM

89.353

8 -0.75469685D+04 13.4750731 0.0017823 440.033 166.975 EM

94.992

9 -0.75337351D+04 13.2333689 0.0017535 421.811 178.700 EM

101.489

10 -0.75210814D+04 12.6537362 0.0016796 405.271 187.814 EM

108.915

11 -0.75108578D+04 10.2236460 0.0013593 390.993 194.304 EM

116.703

12 -0.75039173D+04 6.9404164 0.0009241 379.161 198.451 EM

124.388

13 -0.74993977D+04 4.5196751 0.0006023 369.470 200.668 EM

131.862

14 -0.74962113D+04 3.1863466 0.0004249 361.455 201.382 EM

139.164

15 -0.74936906D+04 2.5206988 0.0003363 354.696 200.951 EM

146.353

16 -0.74914661D+04 2.2245279 0.0002969 348.858 199.651 EM

153.491

17 -0.74892868D+04 2.1793151 0.0002909 343.666 197.687 EM

160.647

18 -0.74869104D+04 2.3764178 0.0003173 338.865 195.203 EM

167.932

19 -0.74840477D+04 2.8627134 0.0003824 334.179 192.294 EM

175.527

20 -0.74803780D+04 3.6696184 0.0004903 329.294 188.998 EM

183.708

21 -0.74756767D+04 4.7013236 0.0006285 323.891 185.299 EM

192.810

22 -0.74700127D+04 5.6640537 0.0007577 317.730 181.150 EM

203.120

23 -0.74638318D+04 6.1808665 0.0008274 310.750 176.496 EM

214.754

24 -0.74577465D+04 6.0852452 0.0008153 303.103 171.327 EM

227.571

25 -0.74521525D+04 5.5940705 0.0007501 295.096 165.766 EM

241.137

26 -0.74471358D+04 5.0166741 0.0006732 287.076 160.066 EM

254.858

27 -0.74427159D+04 4.4198650 0.0005935 279.363 154.525 EM

268.111

28 -0.74389834D+04 3.7325030 0.0005015 272.231 149.423 EM

280.346

29 -0.74360290D+04 2.9544454 0.0003972 265.887 144.952 EM

291.161

30 -0.74338438D+04 2.1851755 0.0002939 260.451 141.189 EM

300.360

31 -0.74323125D+04 1.5312734 0.0002060 255.947 138.109 EM

307.944

32 -0.74312747D+04 1.0378164 0.0001396 252.325 135.622 EM

314.054

33 -0.74305821D+04 0.6926058 0.0000932 249.488 133.614 EM

318.898

TECHNICAL 8 OUTPUT FOR STARTING VALUE SET 174

ITER LOGLIKELIHOOD ABS CHANGE REL CHANGE CLASS COUNTS ALGORITHM

1 -0.22317407D+05 0.0000000 0.0000000 388.754 306.949 EM

6.297

2 -0.77583986D+04 ************ 0.6523611 369.060 314.461 EM

18.479

3 -0.75892365D+04 169.1620739 0.0218037 351.671 317.977 EM

32.352

4 -0.75096849D+04 79.5516596 0.0104822 342.185 317.143 EM

42.672

5 -0.74866131D+04 23.0717738 0.0030723 337.436 314.517 EM

50.046

6 -0.74743560D+04 12.2571378 0.0016372 335.020 310.735 EM

56.245

7 -0.74634539D+04 10.9020374 0.0014586 333.551 306.341 EM

62.108

8 -0.74536446D+04 9.8093680 0.0013143 332.470 301.801 EM

67.728

9 -0.74459841D+04 7.6604906 0.0010278 331.907 297.246 EM

72.847

10 -0.74408934D+04 5.0906434 0.0006837 332.145 292.629 EM

77.226

11 -0.74377905D+04 3.1029321 0.0004170 333.084 288.004 EM

80.912

12 -0.74357865D+04 2.0039623 0.0002694 334.432 283.490 EM

84.079

13 -0.74343258D+04 1.4607105 0.0001964 335.942 279.194 EM

86.864

14 -0.74331665D+04 1.1593631 0.0001559 337.458 275.189 EM

89.353

15 -0.74322158D+04 0.9506402 0.0001279 338.883 271.526 EM

91.592

TECHNICAL 8 OUTPUT FOR STARTING VALUE SET 175

ITER LOGLIKELIHOOD ABS CHANGE REL CHANGE CLASS COUNTS ALGORITHM

1 -0.19407204D+05 0.0000000 0.0000000 115.735 585.220 EM

1.045

2 -0.77044114D+04 ************ 0.6030128 142.578 557.838 EM

1.584

3 -0.76497212D+04 54.6902736 0.0070986 171.686 528.418 EM

1.896

4 -0.76156079D+04 34.1132410 0.0044594 201.621 498.324 EM

2.056

5 -0.75808906D+04 34.7173655 0.0045587 231.172 468.684 EM

2.143

6 -0.75524317D+04 28.4588735 0.0037540 256.849 442.936 EM

2.215

7 -0.75354703D+04 16.9613935 0.0022458 277.392 422.312 EM

2.297

8 -0.75261586D+04 9.3117039 0.0012357 293.036 406.570 EM

2.394

9 -0.75211243D+04 5.0342473 0.0006689 304.630 394.870 EM

2.500

10 -0.75184172D+04 2.7071736 0.0003599 313.149 386.245 EM

2.606

11 -0.75169442D+04 1.4730155 0.0001959 319.419 379.881 EM

2.700

12 -0.75161293D+04 0.8148109 0.0001084 324.059 375.167 EM

2.774

TECHNICAL 8 OUTPUT FOR STARTING VALUE SET 176

ITER LOGLIKELIHOOD ABS CHANGE REL CHANGE CLASS COUNTS ALGORITHM

1 -0.18062107D+05 0.0000000 0.0000000 478.838 160.895 EM

62.267

2 -0.78130883D+04 ************ 0.5674321 486.671 157.453 EM

57.876

3 -0.76650992D+04 147.9890837 0.0189412 470.047 178.896 EM

53.057

4 -0.75863259D+04 78.7733833 0.0102769 451.520 200.980 EM

49.501

5 -0.75612804D+04 25.0454329 0.0033014 432.716 219.391 EM

49.893

6 -0.75482369D+04 13.0435452 0.0017250 414.250 234.876 EM

52.874

7 -0.75383975D+04 9.8393539 0.0013035 396.564 247.512 EM

57.924

8 -0.75298272D+04 8.5703178 0.0011369 379.799 256.987 EM

65.215

9 -0.75215529D+04 8.2743411 0.0010989 363.981 262.682 EM

75.337

10 -0.75126866D+04 8.8663044 0.0011788 349.041 263.900 EM

89.059

11 -0.75024243D+04 10.2622345 0.0013660 334.870 260.357 EM

106.774

12 -0.74908089D+04 11.6154221 0.0015482 321.483 252.796 EM

127.721

13 -0.74793524D+04 11.4564643 0.0015294 309.133 242.829 EM

150.037

14 -0.74697292D+04 9.6232280 0.0012866 298.076 232.010 EM

171.914

15 -0.74622888D+04 7.4404389 0.0009961 288.300 221.323 EM

192.377

16 -0.74564299D+04 5.8589275 0.0007851 279.582 211.216 EM

211.203

17 -0.74514862D+04 4.9436357 0.0006630 271.673 201.823 EM

228.504

18 -0.74470896D+04 4.3966289 0.0005900 264.425 193.169 EM

244.406

19 -0.74431536D+04 3.9360096 0.0005285 257.816 185.281 EM

258.904

20 -0.74397514D+04 3.4022253 0.0004571 251.922 178.205 EM

271.872

21 -0.74369833D+04 2.7680779 0.0003721 246.852 171.985 EM

283.164

22 -0.74348801D+04 2.1031932 0.0002828 242.680 166.615 EM

292.706

23 -0.74333766D+04 1.5034749 0.0002022 239.413 162.038 EM

300.549

24 -0.74323464D+04 1.0301644 0.0001386 236.989 158.155 EM

306.856

25 -0.74316527D+04 0.6937440 0.0000933 235.297 154.848 EM

311.855

TECHNICAL 8 OUTPUT FOR STARTING VALUE SET 177

ITER LOGLIKELIHOOD ABS CHANGE REL CHANGE CLASS COUNTS ALGORITHM

1 -0.21429859D+05 0.0000000 0.0000000 174.173 339.154 EM

188.674

2 -0.76338268D+04 ************ 0.6437762 197.982 318.703 EM

185.315

3 -0.75832363D+04 50.5905628 0.0066272 219.179 297.014 EM

185.807

4 -0.75623820D+04 20.8542341 0.0027500 240.323 277.071 EM

184.606

5 -0.75428333D+04 19.5487383 0.0025850 260.370 260.452 EM

181.178

6 -0.75255783D+04 17.2550228 0.0022876 277.797 247.625 EM

176.577

7 -0.75122178D+04 13.3605138 0.0017753 291.928 237.559 EM

172.514

8 -0.75025788D+04 9.6389970 0.0012831 302.649 229.198 EM

170.153

9 -0.74960855D+04 6.4932962 0.0008655 310.239 221.991 EM

169.770

10 -0.74918063D+04 4.2791757 0.0005709 315.216 215.666 EM

171.118

11 -0.74886952D+04 3.1111252 0.0004153 318.106 210.040 EM

173.854

12 -0.74858726D+04 2.8226055 0.0003769 319.293 204.942 EM

177.765

13 -0.74826620D+04 3.2105910 0.0004289 318.970 200.197 EM

182.834

14 -0.74785780D+04 4.0839452 0.0005458 317.159 195.609 EM

189.232

15 -0.74734257D+04 5.1523255 0.0006889 313.812 190.970 EM

197.218

16 -0.74674449D+04 5.9807951 0.0008003 308.953 186.066 EM

206.981

17 -0.74612531D+04 6.1917857 0.0008292 302.793 180.733 EM

218.474

18 -0.74554328D+04 5.8203040 0.0007801 295.736 174.961 EM

231.303

19 -0.74501830D+04 5.2498406 0.0007042 288.245 168.924 EM

244.831

20 -0.74454789D+04 4.7040983 0.0006314 280.729 162.895 EM

258.376

21 -0.74413486D+04 4.1302360 0.0005547 273.528 157.160 EM

271.311

22 -0.74379041D+04 3.4445505 0.0004629 266.920 151.964 EM

283.116

23 -0.74352250D+04 2.6790612 0.0003602 261.106 147.454 EM

293.440

24 -0.74332786D+04 1.9463991 0.0002618 256.191 143.671 EM

302.138

25 -0.74319366D+04 1.3419909 0.0001805 252.185 140.564 EM

309.251

26 -0.74310405D+04 0.8961706 0.0001206 249.025 138.032 EM

314.944

TECHNICAL 8 OUTPUT FOR STARTING VALUE SET 178

ITER LOGLIKELIHOOD ABS CHANGE REL CHANGE CLASS COUNTS ALGORITHM

1 -0.27746748D+05 0.0000000 0.0000000 158.633 310.997 EM

232.370

2 -0.76021084D+04 ************ 0.7260180 162.991 281.458 EM

257.550

3 -0.75218249D+04 80.2835152 0.0105607 160.795 263.873 EM

277.332

4 -0.75017534D+04 20.0715039 0.0026684 155.782 252.889 EM

293.329

5 -0.74927175D+04 9.0358490 0.0012045 150.496 245.331 EM

306.173

6 -0.74878826D+04 4.8348761 0.0006453 145.808 239.927 EM

316.265

7 -0.74852061D+04 2.6765329 0.0003574 141.862 236.163 EM

323.975

8 -0.74837497D+04 1.4564384 0.0001946 138.562 233.690 EM

329.748

9 -0.74829570D+04 0.7927049 0.0001059 135.771 232.192 EM

334.037

TECHNICAL 8 OUTPUT FOR STARTING VALUE SET 179

ITER LOGLIKELIHOOD ABS CHANGE REL CHANGE CLASS COUNTS ALGORITHM

1 -0.18409193D+05 0.0000000 0.0000000 661.179 38.432 EM

2.389

2 -0.78396856D+04 ************ 0.5741429 660.386 36.008 EM

5.606

3 -0.78020046D+04 37.6809917 0.0048064 642.520 40.734 EM

18.746

4 -0.77384842D+04 63.5204038 0.0081415 609.445 50.543 EM

42.012

5 -0.76747366D+04 63.7475932 0.0082377 567.980 64.511 EM

69.509

6 -0.76189659D+04 55.7706774 0.0072668 523.796 81.597 EM

96.607

7 -0.75720569D+04 46.9090302 0.0061569 478.532 102.705 EM

120.763

8 -0.75262727D+04 45.7842068 0.0060465 433.639 128.546 EM

139.815

9 -0.74830502D+04 43.2225160 0.0057429 395.274 154.480 EM

152.246

10 -0.74546333D+04 28.4168431 0.0037975 368.957 174.507 EM

158.535

11 -0.74421862D+04 12.4470848 0.0016697 352.475 188.490 EM

161.035

12 -0.74369587D+04 5.2275177 0.0007024 342.002 198.383 EM

161.615

13 -0.74344911D+04 2.4676098 0.0003318 335.262 205.580 EM

161.158

14 -0.74332015D+04 1.2896219 0.0001735 330.954 210.952 EM

160.093

15 -0.74324507D+04 0.7507857 0.0001010 328.270 215.065 EM

158.665

TECHNICAL 8 OUTPUT FOR STARTING VALUE SET 180

ITER LOGLIKELIHOOD ABS CHANGE REL CHANGE CLASS COUNTS ALGORITHM

1 -0.18041628D+05 0.0000000 0.0000000 122.219 350.063 EM

229.718

2 -0.76733847D+04 ************ 0.5746845 173.574 325.665 EM

202.761

3 -0.75893948D+04 83.9899251 0.0109456 212.228 299.744 EM

190.028

4 -0.75496368D+04 39.7579867 0.0052386 242.107 277.413 EM

182.480

5 -0.75226308D+04 27.0060340 0.0035771 265.152 259.970 EM

176.877

6 -0.75024667D+04 20.1640814 0.0026805 281.796 248.226 EM

171.978

7 -0.74876184D+04 14.8482746 0.0019791 292.326 242.153 EM

167.521

8 -0.74760956D+04 11.5228409 0.0015389 297.351 241.251 EM

163.398

9 -0.74661672D+04 9.9284115 0.0013280 297.740 244.876 EM

159.384

10 -0.74573749D+04 8.7923058 0.0011776 294.603 252.168 EM

155.229

11 -0.74499544D+04 7.4204327 0.0009950 289.165 262.084 EM

150.751

12 -0.74440431D+04 5.9113113 0.0007935 282.604 273.302 EM

146.094

13 -0.74394776D+04 4.5655492 0.0006133 275.860 284.524 EM

141.617

14 -0.74360872D+04 3.3903829 0.0004557 269.550 294.813 EM

137.637

15 -0.74337050D+04 2.3822158 0.0003204 264.007 303.681 EM

134.312

16 -0.74321089D+04 1.5960396 0.0002147 259.347 311.005 EM

131.647

17 -0.74310658D+04 1.0431052 0.0001404 255.557 316.886 EM

129.558

18 -0.74303866D+04 0.6791704 0.0000914 252.549 321.525 EM

127.925

TECHNICAL 8 OUTPUT FOR STARTING VALUE SET 181

ITER LOGLIKELIHOOD ABS CHANGE REL CHANGE CLASS COUNTS ALGORITHM

1 -0.13036734D+05 0.0000000 0.0000000 112.729 475.539 EM

113.733

2 -0.76218995D+04 5414.8344558 0.4153521 149.388 426.785 EM

125.827

3 -0.74868905D+04 135.0090160 0.0177133 175.533 387.266 EM

139.201

4 -0.74488331D+04 38.0573630 0.0050832 191.842 365.368 EM

144.791

5 -0.74381048D+04 10.7283243 0.0014403 202.854 352.174 EM

146.972

6 -0.74340183D+04 4.0865477 0.0005494 210.685 343.666 EM

147.649

7 -0.74322256D+04 1.7926562 0.0002411 216.390 338.112 EM

147.499

8 -0.74313573D+04 0.8683329 0.0001168 220.625 334.531 EM

146.844

TECHNICAL 8 OUTPUT FOR STARTING VALUE SET 182

ITER LOGLIKELIHOOD ABS CHANGE REL CHANGE CLASS COUNTS ALGORITHM

1 -0.16141104D+05 0.0000000 0.0000000 37.897 546.261 EM

117.842

2 -0.76150687D+04 8526.0351200 0.5282188 49.859 515.198 EM

136.943

3 -0.75482810D+04 66.7876235 0.0087705 60.272 484.930 EM

156.798

4 -0.75254679D+04 22.8131128 0.0030223 69.256 459.876 EM

172.868

5 -0.75126842D+04 12.7836838 0.0016987 77.629 437.814 EM

186.557

6 -0.75028903D+04 9.7939092 0.0013036 85.833 417.838 EM

198.330

7 -0.74949508D+04 7.9395319 0.0010582 94.028 399.915 EM

208.057

8 -0.74885505D+04 6.4003367 0.0008540 102.251 384.191 EM

215.558

9 -0.74833068D+04 5.2436435 0.0007002 110.561 370.637 EM

220.802

10 -0.74786773D+04 4.6294857 0.0006186 119.124 358.982 EM

223.893

11 -0.74740314D+04 4.6459741 0.0006212 128.207 348.810 EM

224.984

12 -0.74687501D+04 5.2812132 0.0007066 138.032 339.743 EM

224.225

13 -0.74625457D+04 6.2044355 0.0008307 148.464 331.743 EM

221.792

14 -0.74559978D+04 6.5479327 0.0008774 158.823 325.192 EM

217.985

15 -0.74502974D+04 5.7003793 0.0007645 168.335 320.371 EM

213.293

16 -0.74460150D+04 4.2824140 0.0005748 176.631 317.124 EM

208.245

17 -0.74429344D+04 3.0806221 0.0004137 183.700 315.091 EM

203.209

18 -0.74406737D+04 2.2606579 0.0003037 189.686 313.946 EM

198.369

19 -0.74389532D+04 1.7205109 0.0002312 194.769 313.439 EM

193.793

20 -0.74375991D+04 1.3541055 0.0001820 199.114 313.392 EM

189.494

21 -0.74365042D+04 1.0948563 0.0001472 202.863 313.674 EM

185.463

22 -0.74356001D+04 0.9041178 0.0001216 206.129 314.188 EM

181.682

TECHNICAL 8 OUTPUT FOR STARTING VALUE SET 183

ITER LOGLIKELIHOOD ABS CHANGE REL CHANGE CLASS COUNTS ALGORITHM

1 -0.24577300D+05 0.0000000 0.0000000 64.833 548.764 EM

88.403

2 -0.76882232D+04 ************ 0.6871819 55.894 523.701 EM

122.405

3 -0.75984807D+04 89.7424650 0.0116727 67.741 484.469 EM

149.790

4 -0.75361717D+04 62.3089830 0.0082002 80.609 451.294 EM

170.097

5 -0.75105101D+04 25.6616665 0.0034051 89.223 428.086 EM

184.691

6 -0.74994683D+04 11.0418024 0.0014702 96.965 408.907 EM

196.128

7 -0.74913413D+04 8.1269456 0.0010837 104.954 391.947 EM

205.099

8 -0.74846909D+04 6.6504438 0.0008878 113.325 377.017 EM

211.657

9 -0.74788815D+04 5.8093806 0.0007762 122.187 363.953 EM

215.860

10 -0.74731726D+04 5.7088613 0.0007633 131.762 352.402 EM

217.836

11 -0.74668586D+04 6.3140326 0.0008449 142.177 342.082 EM

217.741

12 -0.74598199D+04 7.0386991 0.0009427 153.045 333.163 EM

215.792

13 -0.74530468D+04 6.7730867 0.0009079 163.468 326.149 EM

212.383

14 -0.74477184D+04 5.3284292 0.0007149 172.731 321.170 EM

208.100

15 -0.74439658D+04 3.7525666 0.0005039 180.633 317.887 EM

203.481

16 -0.74413312D+04 2.6346510 0.0003539 187.280 315.859 EM

198.861

17 -0.74394026D+04 1.9286025 0.0002592 192.870 314.730 EM

194.400

18 -0.74379264D+04 1.4761324 0.0001984 197.600 314.243 EM

190.157

19 -0.74367553D+04 1.1711176 0.0001575 201.640 314.212 EM

186.148

20 -0.74358005D+04 0.9547901 0.0001284 205.128 314.504 EM

182.369

TECHNICAL 8 OUTPUT FOR STARTING VALUE SET 184

ITER LOGLIKELIHOOD ABS CHANGE REL CHANGE CLASS COUNTS ALGORITHM

1 -0.20143039D+05 0.0000000 0.0000000 499.239 66.510 EM

136.252

2 -0.78092311D+04 ************ 0.6123112 494.739 77.059 EM

130.202

3 -0.76541984D+04 155.0326361 0.0198525 464.112 86.148 EM

151.740

4 -0.75424233D+04 111.7751146 0.0146031 438.579 93.138 EM

170.284

5 -0.75074922D+04 34.9311406 0.0046313 419.675 100.969 EM

181.356

6 -0.74953029D+04 12.1892596 0.0016236 405.702 107.678 EM

188.620

7 -0.74901775D+04 5.1254370 0.0006838 395.509 113.057 EM

193.434

8 -0.74877881D+04 2.3893161 0.0003190 388.009 117.490 EM

196.500

9 -0.74865212D+04 1.2669349 0.0001692 382.394 121.290 EM

198.316

10 -0.74857669D+04 0.7543465 0.0001008 378.115 124.653 EM

199.232

TECHNICAL 8 OUTPUT FOR STARTING VALUE SET 185

ITER LOGLIKELIHOOD ABS CHANGE REL CHANGE CLASS COUNTS ALGORITHM

1 -0.21212110D+05 0.0000000 0.0000000 136.790 536.156 EM

29.055

2 -0.76218815D+04 ************ 0.6406825 158.646 510.335 EM

33.018

3 -0.75814083D+04 40.4732051 0.0053101 183.795 484.083 EM

34.123

4 -0.75602867D+04 21.1216143 0.0027860 207.979 459.303 EM

34.718

5 -0.75424577D+04 17.8289659 0.0023582 230.868 435.390 EM

35.743

6 -0.75244871D+04 17.9705629 0.0023826 252.393 411.632 EM

37.976

7 -0.75048789D+04 19.6081807 0.0026059 271.961 388.197 EM

41.841

8 -0.74852016D+04 19.6773458 0.0026219 288.547 366.520 EM

46.933

9 -0.74691145D+04 16.0871331 0.0021492 301.339 348.213 EM

52.448

10 -0.74582288D+04 10.8857059 0.0014574 310.561 333.652 EM

57.786

11 -0.74512880D+04 6.9407340 0.0009306 317.087 322.151 EM

62.761

12 -0.74465666D+04 4.7214640 0.0006336 321.825 312.820 EM

67.355

13 -0.74430871D+04 3.4794784 0.0004673 325.445 304.982 EM

71.572

14 -0.74403899D+04 2.6971572 0.0003624 328.371 298.203 EM

75.426

15 -0.74382321D+04 2.1577837 0.0002900 330.850 292.212 EM

78.937

16 -0.74364651D+04 1.7670756 0.0002376 333.028 286.840 EM

82.132

17 -0.74349926D+04 1.4724660 0.0001980 334.981 281.983 EM

85.036

18 -0.74337537D+04 1.2389211 0.0001666 336.744 277.582 EM

87.674

19 -0.74327116D+04 1.0420651 0.0001402 338.324 273.610 EM

90.066

20 -0.74318451D+04 0.8664663 0.0001166 339.712 270.055 EM

92.233

TECHNICAL 8 OUTPUT FOR STARTING VALUE SET 186

ITER LOGLIKELIHOOD ABS CHANGE REL CHANGE CLASS COUNTS ALGORITHM

1 -0.17472586D+05 0.0000000 0.0000000 8.610 198.891 EM

494.500

2 -0.76785203D+04 9794.0653376 0.5605390 7.822 208.417 EM

485.761

3 -0.76165729D+04 61.9474001 0.0080676 7.925 227.066 EM

467.009

4 -0.75768914D+04 39.6814812 0.0052099 8.986 248.035 EM

444.979

5 -0.75453774D+04 31.5139314 0.0041592 12.914 263.989 EM

425.098

6 -0.75211435D+04 24.2339923 0.0032118 20.877 271.995 EM

409.128

7 -0.75066716D+04 14.4718584 0.0019242 27.825 276.862 EM

397.313

8 -0.75007381D+04 5.9335009 0.0007904 33.450 279.684 EM

388.866

9 -0.74975858D+04 3.1522663 0.0004203 38.539 280.646 EM

382.815

10 -0.74955449D+04 2.0409638 0.0002722 43.265 280.290 EM

378.445

11 -0.74940978D+04 1.4470464 0.0001931 47.679 279.058 EM

375.263

12 -0.74930077D+04 1.0901037 0.0001455 51.822 277.253 EM

372.926

13 -0.74921479D+04 0.8597697 0.0001147 55.731 275.073 EM

371.196

TECHNICAL 8 OUTPUT FOR STARTING VALUE SET 187

ITER LOGLIKELIHOOD ABS CHANGE REL CHANGE CLASS COUNTS ALGORITHM

1 -0.19629804D+05 0.0000000 0.0000000 623.923 71.625 EM

6.452

2 -0.78056036D+04 ************ 0.6023596 616.697 74.101 EM

11.202

3 -0.77088659D+04 96.7376818 0.0123934 589.218 95.475 EM

17.306

4 -0.76463774D+04 62.4885209 0.0081061 560.419 120.579 EM

21.002

5 -0.76091407D+04 37.2367180 0.0048699 533.491 145.197 EM

23.312

6 -0.75831950D+04 25.9456810 0.0034098 508.319 168.546 EM

25.135

7 -0.75646680D+04 18.5270067 0.0024432 484.646 190.603 EM

26.751

8 -0.75501733D+04 14.4946574 0.0019161 462.597 211.106 EM

28.297

9 -0.75384134D+04 11.7599268 0.0015576 442.415 229.826 EM

29.759

10 -0.75290044D+04 9.4089693 0.0012481 424.124 246.789 EM

31.087

11 -0.75214564D+04 7.5480271 0.0010025 407.661 262.104 EM

32.235

12 -0.75153814D+04 6.0749757 0.0008077 393.045 275.813 EM

33.142

13 -0.75105900D+04 4.7913965 0.0006375 380.349 287.906 EM

33.746

14 -0.75069506D+04 3.6394139 0.0004846 369.581 298.407 EM

34.012

15 -0.75042805D+04 2.6700815 0.0003557 360.630 307.433 EM

33.936

16 -0.75023489D+04 1.9316397 0.0002574 353.284 315.179 EM

33.537

17 -0.75009253D+04 1.4236239 0.0001898 347.284 321.876 EM

32.840

18 -0.74998141D+04 1.1111305 0.0001481 342.374 327.745 EM

31.881

19 -0.74988655D+04 0.9485928 0.0001265 338.327 332.971 EM

30.702

TECHNICAL 8 OUTPUT FOR STARTING VALUE SET 188

ITER LOGLIKELIHOOD ABS CHANGE REL CHANGE CLASS COUNTS ALGORITHM

1 -0.26115219D+05 0.0000000 0.0000000 1.603 240.661 EM

459.736

2 -0.76416771D+04 ************ 0.7073861 1.733 255.863 EM

444.404

3 -0.75725001D+04 69.1769670 0.0090526 2.360 273.560 EM

426.080

4 -0.75375819D+04 34.9182264 0.0046112 3.449 288.705 EM

409.846

5 -0.75235397D+04 14.0422383 0.0018630 5.130 299.658 EM

397.211

6 -0.75161656D+04 7.3740842 0.0009801 6.727 307.137 EM

388.136

7 -0.75118140D+04 4.3516005 0.0005790 7.530 312.668 EM

381.802

8 -0.75101447D+04 1.6692554 0.0002222 7.719 316.990 EM

377.291

9 -0.75095449D+04 0.5998599 0.0000799 7.714 320.314 EM

373.972

TECHNICAL 8 OUTPUT FOR STARTING VALUE SET 189

ITER LOGLIKELIHOOD ABS CHANGE REL CHANGE CLASS COUNTS ALGORITHM

1 -0.29620215D+05 0.0000000 0.0000000 323.039 211.412 EM

167.550

2 -0.76125767D+04 ************ 0.7429939 319.992 185.701 EM

196.307

3 -0.75545002D+04 58.0764385 0.0076290 308.058 174.260 EM

219.681

4 -0.75346011D+04 19.8991754 0.0026341 293.038 169.704 EM

239.258

5 -0.75216217D+04 12.9793588 0.0017226 277.640 168.462 EM

255.898

6 -0.75122741D+04 9.3476554 0.0012428 263.123 169.084 EM

269.793

7 -0.75055987D+04 6.6753602 0.0008886 250.017 170.975 EM

281.008

8 -0.75009372D+04 4.6615232 0.0006211 238.418 173.870 EM

289.712

9 -0.74977103D+04 3.2268639 0.0004302 228.183 177.605 EM

296.212

10 -0.74954343D+04 2.2759619 0.0003036 219.077 182.023 EM

300.899

11 -0.74937551D+04 1.6792430 0.0002240 210.870 186.968 EM

304.162

12 -0.74924425D+04 1.3126440 0.0001752 203.373 192.285 EM

306.342

13 -0.74913589D+04 1.0836106 0.0001446 196.447 197.837 EM

307.716

14 -0.74904260D+04 0.9328107 0.0001245 189.996 203.512 EM

308.493

TECHNICAL 8 OUTPUT FOR STARTING VALUE SET 190

ITER LOGLIKELIHOOD ABS CHANGE REL CHANGE CLASS COUNTS ALGORITHM

1 -0.11658758D+05 0.0000000 0.0000000 164.352 490.437 EM

47.211

2 -0.75605613D+04 4098.1965013 0.3515123 198.243 440.262 EM

63.495

3 -0.74793623D+04 81.1990007 0.0107398 221.851 402.932 EM

77.217

4 -0.74492424D+04 30.1198964 0.0040271 235.959 379.175 EM

86.866

5 -0.74366458D+04 12.5965803 0.0016910 243.877 364.855 EM

93.269

6 -0.74319240D+04 4.7218559 0.0006349 248.122 356.413 EM

97.465

7 -0.74302052D+04 1.7187968 0.0002313 250.285 351.383 EM

100.331

8 -0.74295465D+04 0.6586836 0.0000886 251.277 348.346 EM

102.376

TECHNICAL 8 OUTPUT FOR STARTING VALUE SET 191

ITER LOGLIKELIHOOD ABS CHANGE REL CHANGE CLASS COUNTS ALGORITHM

1 -0.23139869D+05 0.0000000 0.0000000 23.068 535.421 EM

143.511

2 -0.76503865D+04 ************ 0.6693851 14.679 516.932 EM

170.389

3 -0.76015096D+04 48.8769501 0.0063888 12.511 493.301 EM

196.188

4 -0.75798815D+04 21.6281094 0.0028452 12.319 470.018 EM

219.663

5 -0.75644062D+04 15.4752247 0.0020416 13.171 448.029 EM

240.800

6 -0.75524239D+04 11.9823465 0.0015840 14.663 427.750 EM

259.587

7 -0.75430777D+04 9.3462115 0.0012375 16.543 409.285 EM

276.172

8 -0.75357042D+04 7.3734717 0.0009775 18.677 392.665 EM

290.659

9 -0.75298704D+04 5.8338246 0.0007742 21.030 377.957 EM

303.013

10 -0.75253366D+04 4.5337555 0.0006021 23.618 365.215 EM

313.167

11 -0.75219095D+04 3.4271687 0.0004554 26.459 354.389 EM

321.153

12 -0.75193645D+04 2.5449615 0.0003383 29.553 345.304 EM

327.143

13 -0.75174666D+04 1.8978514 0.0002524 32.876 337.712 EM

331.411

14 -0.75160136D+04 1.4530401 0.0001933 36.386 331.352 EM

334.262

15 -0.75148548D+04 1.1588508 0.0001542 40.035 325.985 EM

335.981

16 -0.75138863D+04 0.9684353 0.0001289 43.776 321.413 EM

336.810

TECHNICAL 8 OUTPUT FOR STARTING VALUE SET 192

ITER LOGLIKELIHOOD ABS CHANGE REL CHANGE CLASS COUNTS ALGORITHM

1 -0.23043560D+05 0.0000000 0.0000000 151.869 342.418 EM

207.714

2 -0.76541815D+04 ************ 0.6678386 157.799 339.381 EM

204.820

3 -0.75741796D+04 80.0018218 0.0104520 165.202 336.733 EM

200.065

4 -0.75152685D+04 58.9111783 0.0077779 172.905 332.390 EM

196.705

5 -0.74753630D+04 39.9054295 0.0053099 180.992 326.677 EM

194.330

6 -0.74536672D+04 21.6957907 0.0029023 188.762 322.006 EM

191.232

7 -0.74431027D+04 10.5645833 0.0014174 195.276 319.356 EM

187.369

8 -0.74383348D+04 4.7678343 0.0006406 200.534 318.105 EM

183.361

9 -0.74360557D+04 2.2790977 0.0003064 204.828 317.623 EM

179.549

10 -0.74348013D+04 1.2543935 0.0001687 208.402 317.586 EM

176.012

11 -0.74339908D+04 0.8104965 0.0001090 211.428 317.835 EM

172.736

TECHNICAL 8 OUTPUT FOR STARTING VALUE SET 193

ITER LOGLIKELIHOOD ABS CHANGE REL CHANGE CLASS COUNTS ALGORITHM

1 -0.25676584D+05 0.0000000 0.0000000 3.001 441.194 EM

257.805

2 -0.76358062D+04 ************ 0.7026160 3.053 431.454 EM

267.492

3 -0.75437075D+04 92.0986108 0.0120614 3.196 417.647 EM

281.157

4 -0.75287582D+04 14.9493319 0.0019817 3.377 404.084 EM

294.539

5 -0.75230320D+04 5.7261820 0.0007606 3.571 391.481 EM

306.948

6 -0.75188380D+04 4.1940512 0.0005575 3.762 380.280 EM

317.957

7 -0.75156442D+04 3.1937408 0.0004248 3.937 370.688 EM

327.375

8 -0.75133347D+04 2.3095264 0.0003073 4.086 362.722 EM

335.192

9 -0.75117541D+04 1.5806109 0.0002104 4.213 356.261 EM

341.526

10 -0.75107180D+04 1.0361388 0.0001379 4.328 351.111 EM

346.561

11 -0.75100564D+04 0.6615267 0.0000881 4.440 347.056 EM

350.505

TECHNICAL 8 OUTPUT FOR STARTING VALUE SET 194

ITER LOGLIKELIHOOD ABS CHANGE REL CHANGE CLASS COUNTS ALGORITHM

1 -0.18933861D+05 0.0000000 0.0000000 48.691 605.971 EM

47.338

2 -0.76881859D+04 ************ 0.5939452 73.368 573.836 EM

54.796

3 -0.76192583D+04 68.9276638 0.0089654 96.815 535.357 EM

69.828

4 -0.75695646D+04 49.6937179 0.0065221 117.149 499.941 EM

84.910

5 -0.75368194D+04 32.7451408 0.0043259 133.756 469.897 EM

98.347

6 -0.75157811D+04 21.0383215 0.0027914 146.848 445.531 EM

109.621

7 -0.75028074D+04 12.9737105 0.0017262 156.847 426.378 EM

118.775

8 -0.74951170D+04 7.6903587 0.0010250 164.262 411.626 EM

126.112

9 -0.74906251D+04 4.4919698 0.0005993 169.619 400.359 EM

132.022

10 -0.74879678D+04 2.6572812 0.0003547 173.394 391.740 EM

136.866

11 -0.74863486D+04 1.6192058 0.0002162 175.971 385.098 EM

140.931

12 -0.74853288D+04 1.0197758 0.0001362 177.643 379.934 EM

144.423

13 -0.74846677D+04 0.6611401 0.0000883 178.632 375.884 EM

147.484

TECHNICAL 8 OUTPUT FOR STARTING VALUE SET 195

ITER LOGLIKELIHOOD ABS CHANGE REL CHANGE CLASS COUNTS ALGORITHM

1 -0.21069233D+05 0.0000000 0.0000000 19.798 660.171 EM

22.031

2 -0.77271267D+04 ************ 0.6332507 30.417 626.939 EM

44.644

3 -0.76378632D+04 89.2635683 0.0115520 46.514 584.466 EM

71.020

4 -0.75880166D+04 49.8465798 0.0065262 60.540 546.392 EM

95.068

5 -0.75581828D+04 29.8338209 0.0039317 73.104 513.240 EM

115.656

6 -0.75372157D+04 20.9671038 0.0027741 84.915 483.844 EM

133.241

7 -0.75206676D+04 16.5480962 0.0021955 96.492 457.228 EM

148.280

8 -0.75065305D+04 14.1370098 0.0018798 108.136 432.789 EM

161.075

9 -0.74938648D+04 12.6656998 0.0016873 120.070 410.265 EM

171.665

10 -0.74820005D+04 11.8643034 0.0015832 132.625 389.492 EM

179.883

11 -0.74701735D+04 11.8270700 0.0015807 146.015 370.482 EM

185.503

12 -0.74584516D+04 11.7218524 0.0015692 159.539 354.042 EM

188.419

13 -0.74488788D+04 9.5727859 0.0012835 171.714 341.367 EM

188.919

14 -0.74428647D+04 6.0141704 0.0008074 181.753 332.526 EM

187.721

15 -0.74394621D+04 3.4025402 0.0004572 189.761 326.690 EM

185.549

16 -0.74374307D+04 2.0314484 0.0002731 196.142 322.970 EM

182.888

17 -0.74360862D+04 1.3445034 0.0001808 201.286 320.703 EM

180.011

18 -0.74351118D+04 0.9744142 0.0001310 205.498 319.434 EM

177.068

TECHNICAL 8 OUTPUT FOR STARTING VALUE SET 196

ITER LOGLIKELIHOOD ABS CHANGE REL CHANGE CLASS COUNTS ALGORITHM

1 -0.19530014D+05 0.0000000 0.0000000 132.704 176.266 EM

393.030

2 -0.77427934D+04 ************ 0.6035439 129.804 187.097 EM

385.099

3 -0.76137100D+04 129.0833986 0.0166714 137.778 200.153 EM

364.069

4 -0.75474059D+04 66.3040372 0.0087085 142.465 214.861 EM

344.674

5 -0.75260328D+04 21.3730719 0.0028318 143.401 228.907 EM

329.692

6 -0.75174762D+04 8.5566097 0.0011369 142.838 241.696 EM

317.465

7 -0.75122870D+04 5.1892400 0.0006903 141.751 253.134 EM

307.115

8 -0.75086390D+04 3.6480202 0.0004856 140.428 263.264 EM

298.308

9 -0.75059821D+04 2.6568916 0.0003538 138.953 272.195 EM

290.853

10 -0.75040208D+04 1.9612647 0.0002613 137.357 280.083 EM

284.561

11 -0.75025314D+04 1.4894555 0.0001985 135.655 287.119 EM

279.226

12 -0.75013235D+04 1.2078960 0.0001610 133.863 293.512 EM

274.625

13 -0.75002366D+04 1.0868959 0.0001449 132.000 299.467 EM

270.533

14 -0.74991437D+04 1.0928395 0.0001457 130.103 305.162 EM

266.735

15 -0.74979581D+04 1.1856607 0.0001581 128.228 310.730 EM

263.042

16 -0.74966401D+04 1.3179390 0.0001758 126.451 316.250 EM

259.299

17 -0.74952010D+04 1.4390905 0.0001920 124.857 321.732 EM

255.410

18 -0.74936944D+04 1.5066100 0.0002010 123.523 327.133 EM

251.343

19 -0.74921965D+04 1.4979109 0.0001999 122.505 332.365 EM

247.130

20 -0.74907832D+04 1.4133012 0.0001886 121.833 337.317 EM

242.850

21 -0.74895138D+04 1.2694684 0.0001695 121.515 341.882 EM

238.603

22 -0.74884236D+04 1.0901847 0.0001456 121.544 345.973 EM

234.484

23 -0.74875239D+04 0.8996877 0.0001201 121.902 349.534 EM

230.564

TECHNICAL 8 OUTPUT FOR STARTING VALUE SET 197

ITER LOGLIKELIHOOD ABS CHANGE REL CHANGE CLASS COUNTS ALGORITHM

1 -0.22174637D+05 0.0000000 0.0000000 129.987 105.059 EM

466.955

2 -0.77517297D+04 ************ 0.6504236 137.637 98.554 EM

465.809

3 -0.76512187D+04 100.5109462 0.0129663 151.649 101.573 EM

448.779

4 -0.76112790D+04 39.9397189 0.0052200 166.482 105.490 EM

430.028

5 -0.75902793D+04 20.9996868 0.0027590 182.285 109.338 EM

410.378

6 -0.75685963D+04 21.6829931 0.0028567 199.003 114.784 EM

388.212

7 -0.75344921D+04 34.1042520 0.0045060 214.768 122.083 EM

365.149

8 -0.74879077D+04 46.5843553 0.0061828 225.868 127.854 EM

348.278

9 -0.74497238D+04 38.1839462 0.0050994 231.457 130.456 EM

340.087

10 -0.74341819D+04 15.5418856 0.0020862 234.084 130.905 EM

337.010

11 -0.74304891D+04 3.6927897 0.0004967 235.715 130.613 EM

335.672

12 -0.74296748D+04 0.8142997 0.0001096 236.934 130.097 EM

334.969

TECHNICAL 8 OUTPUT FOR STARTING VALUE SET 198

ITER LOGLIKELIHOOD ABS CHANGE REL CHANGE CLASS COUNTS ALGORITHM

1 -0.14055348D+05 0.0000000 0.0000000 670.182 0.000 EM

31.818

2 -0.77982263D+04 6257.1218266 0.4451773 654.288 0.000 EM

47.712

3 -0.77324982D+04 65.7281469 0.0084286 627.366 0.000 EM

74.634

4 -0.76854501D+04 47.0480336 0.0060845 598.635 0.001 EM

103.364

5 -0.76510914D+04 34.3586913 0.0044706 570.500 0.023 EM

131.476

6 -0.76249682D+04 26.1232235 0.0034143 543.531 0.509 EM

157.959

7 -0.76011848D+04 23.7833970 0.0031191 517.691 1.598 EM

182.712

8 -0.75817138D+04 19.4710625 0.0025616 493.111 2.064 EM

206.825

9 -0.75661672D+04 15.5465610 0.0020505 470.295 2.243 EM

229.462

10 -0.75537042D+04 12.4629852 0.0016472 449.576 2.367 EM

250.057

11 -0.75439089D+04 9.7953106 0.0012968 430.936 2.464 EM

268.601

12 -0.75361708D+04 7.7381221 0.0010257 414.257 2.539 EM

285.204

13 -0.75300283D+04 6.1424821 0.0008151 399.531 2.596 EM

299.873

14 -0.75252555D+04 4.7727879 0.0006338 386.835 2.638 EM

312.527

15 -0.75217211D+04 3.5343965 0.0004697 376.205 2.671 EM

323.124

16 -0.75192556D+04 2.4654986 0.0003278 367.557 2.698 EM

331.745

17 -0.75176298D+04 1.6257718 0.0002162 360.685 2.723 EM

338.592

18 -0.75166047D+04 1.0251281 0.0001364 355.319 2.745 EM

343.936

19 -0.75159786D+04 0.6260679 0.0000833 351.178 2.766 EM

348.056

TECHNICAL 8 OUTPUT FOR STARTING VALUE SET 199

ITER LOGLIKELIHOOD ABS CHANGE REL CHANGE CLASS COUNTS ALGORITHM

1 -0.20881694D+05 0.0000000 0.0000000 18.587 614.631 EM

68.782

2 -0.77223517D+04 ************ 0.6301856 15.169 591.512 EM

95.318

3 -0.76370739D+04 85.2778501 0.0110430 27.328 550.552 EM

124.119

4 -0.75657994D+04 71.2745122 0.0093327 41.721 512.193 EM

148.085

5 -0.75377332D+04 28.0661774 0.0037096 51.890 483.010 EM

167.099

6 -0.75226870D+04 15.0462002 0.0019961 60.386 458.342 EM

183.272

7 -0.75115083D+04 11.1787224 0.0014860 68.131 436.482 EM

197.388

8 -0.75027195D+04 8.7887981 0.0011700 75.478 417.009 EM

209.512

9 -0.74958975D+04 6.8219508 0.0009093 82.584 399.890 EM

219.526

10 -0.74907143D+04 5.1832798 0.0006915 89.515 385.091 EM

227.393

11 -0.74867603D+04 3.9539627 0.0005278 96.320 372.477 EM

233.203

12 -0.74836149D+04 3.1453915 0.0004201 103.068 361.819 EM

237.113

13 -0.74808969D+04 2.7179672 0.0003632 109.876 352.834 EM

239.290

14 -0.74782523D+04 2.6446520 0.0003535 116.916 345.197 EM

239.887

15 -0.74753162D+04 2.9360679 0.0003926 124.420 338.554 EM

239.026

16 -0.74716904D+04 3.6258369 0.0004850 132.638 332.565 EM

236.797

17 -0.74670264D+04 4.6639844 0.0006242 141.680 327.032 EM

233.288

18 -0.74613552D+04 5.6712015 0.0007595 151.272 322.083 EM

228.645

19 -0.74554354D+04 5.9198328 0.0007934 160.746 318.090 EM

223.164

20 -0.74502844D+04 5.1509667 0.0006909 169.476 315.240 EM

217.284

21 -0.74463009D+04 3.9834506 0.0005347 177.176 313.410 EM

211.414

22 -0.74433224D+04 2.9785698 0.0004000 183.829 312.370 EM

205.801

23 -0.74410670D+04 2.2553198 0.0003030 189.540 311.916 EM

200.543

24 -0.74393148D+04 1.7522871 0.0002355 194.448 311.896 EM

195.657

25 -0.74379185D+04 1.3962543 0.0001877 198.687 312.194 EM

191.120

26 -0.74367818D+04 1.1366922 0.0001528 202.375 312.722 EM

186.903

27 -0.74358400D+04 0.9418001 0.0001266 205.613 313.413 EM

182.974

TECHNICAL 8 OUTPUT FOR STARTING VALUE SET 200

ITER LOGLIKELIHOOD ABS CHANGE REL CHANGE CLASS COUNTS ALGORITHM

1 -0.20953553D+05 0.0000000 0.0000000 194.127 273.527 EM

234.346

2 -0.78035245D+04 ************ 0.6275799 185.648 283.467 EM

232.884

3 -0.76844124D+04 119.1121174 0.0152639 181.010 300.054 EM

220.936

4 -0.75841450D+04 100.2673980 0.0130482 174.108 316.760 EM

211.132

5 -0.75270808D+04 57.0642490 0.0075242 169.970 328.132 EM

203.898

6 -0.75047537D+04 22.3270410 0.0029662 169.521 335.499 EM

196.980

7 -0.74927667D+04 11.9869779 0.0015973 170.822 340.415 EM

190.763

8 -0.74870254D+04 5.7413614 0.0007663 172.454 343.891 EM

185.655

9 -0.74848093D+04 2.2161227 0.0002960 173.936 346.503 EM

181.560

10 -0.74839595D+04 0.8497464 0.0001135 175.245 348.542 EM

178.213

TECHNICAL 8 OUTPUT FOR STARTING VALUE SET 201

ITER LOGLIKELIHOOD ABS CHANGE REL CHANGE CLASS COUNTS ALGORITHM

1 -0.20378601D+05 0.0000000 0.0000000 91.477 588.943 EM

21.580

2 -0.77367398D+04 ************ 0.6203498 111.631 551.889 EM

38.480

3 -0.76013482D+04 135.3916443 0.0174998 143.034 501.131 EM

57.835

4 -0.75300995D+04 71.2486859 0.0093732 168.723 458.354 EM

74.923

5 -0.74861041D+04 43.9953504 0.0058426 190.716 422.560 EM

88.724

6 -0.74554912D+04 30.6129302 0.0040893 208.147 395.077 EM

98.777

7 -0.74398250D+04 15.6661492 0.0021013 220.492 375.932 EM

105.576

8 -0.74333133D+04 6.5117017 0.0008752 228.850 363.157 EM

109.993

9 -0.74307048D+04 2.6085391 0.0003509 234.417 354.796 EM

112.787

10 -0.74296598D+04 1.0450278 0.0001406 238.103 349.387 EM

114.510

11 -0.74292372D+04 0.4225444 0.0000569 240.549 345.914 EM

115.536

TECHNICAL 8 OUTPUT FOR STARTING VALUE SET 202

ITER LOGLIKELIHOOD ABS CHANGE REL CHANGE CLASS COUNTS ALGORITHM

1 -0.27303036D+05 0.0000000 0.0000000 122.030 160.394 EM

419.576

2 -0.76071122D+04 ************ 0.7213822 115.328 178.274 EM

408.398

3 -0.75266585D+04 80.4537603 0.0105761 115.895 188.894 EM

397.211

4 -0.74992942D+04 27.3642397 0.0036356 119.258 194.850 EM

387.892

5 -0.74895686D+04 9.7256763 0.0012969 122.668 198.538 EM

380.794

6 -0.74857292D+04 3.8393999 0.0005126 125.321 201.293 EM

375.386

7 -0.74840346D+04 1.6946004 0.0002264 127.211 203.603 EM

371.186

8 -0.74832006D+04 0.8339168 0.0001114 128.503 205.630 EM

367.866

TECHNICAL 8 OUTPUT FOR STARTING VALUE SET 203

ITER LOGLIKELIHOOD ABS CHANGE REL CHANGE CLASS COUNTS ALGORITHM

1 -0.17812687D+05 0.0000000 0.0000000 3.774 80.636 EM

617.590

2 -0.78147836D+04 9997.9034587 0.5612799 2.197 88.272 EM

611.530

3 -0.77644585D+04 50.3251424 0.0064397 4.544 107.076 EM

590.380

4 -0.76926939D+04 71.7645846 0.0092427 18.745 134.340 EM

548.915

5 -0.76007247D+04 91.9691898 0.0119554 31.672 163.585 EM

506.743

6 -0.75439028D+04 56.8218481 0.0074758 41.970 187.252 EM

472.777

7 -0.75194753D+04 24.4275300 0.0032380 50.339 205.169 EM

446.493

8 -0.75077028D+04 11.7725037 0.0015656 57.372 219.410 EM

425.218

9 -0.75001330D+04 7.5698387 0.0010083 63.531 231.073 EM

407.397

10 -0.74948588D+04 5.2741168 0.0007032 69.074 240.624 EM

392.302

11 -0.74911690D+04 3.6898230 0.0004923 74.143 248.362 EM

379.495

12 -0.74885749D+04 2.5941060 0.0003463 78.827 254.536 EM

368.637

13 -0.74867278D+04 1.8471376 0.0002467 83.195 259.358 EM

359.447

14 -0.74853880D+04 1.3398080 0.0001790 87.303 263.005 EM

351.692

15 -0.74843884D+04 0.9995762 0.0001335 91.201 265.625 EM

345.174

TECHNICAL 8 OUTPUT FOR STARTING VALUE SET 204

ITER LOGLIKELIHOOD ABS CHANGE REL CHANGE CLASS COUNTS ALGORITHM

1 -0.21187955D+05 0.0000000 0.0000000 170.292 480.437 EM

51.271

2 -0.76119073D+04 ************ 0.6407436 204.827 453.351 EM

43.822

3 -0.75374126D+04 74.4947655 0.0097866 222.189 429.515 EM

50.296

4 -0.75107027D+04 26.7098675 0.0035436 231.243 412.599 EM

58.158

5 -0.74989732D+04 11.7295295 0.0015617 236.676 400.765 EM

64.559

6 -0.74937949D+04 5.1782527 0.0006905 239.845 392.308 EM

69.848

7 -0.74912531D+04 2.5417928 0.0003392 241.406 386.111 EM

74.483

8 -0.74898151D+04 1.4379884 0.0001920 241.840 381.475 EM

78.684

9 -0.74889033D+04 0.9118353 0.0001217 241.490 377.950 EM

82.561

TECHNICAL 8 OUTPUT FOR STARTING VALUE SET 205

ITER LOGLIKELIHOOD ABS CHANGE REL CHANGE CLASS COUNTS ALGORITHM

1 -0.23849969D+05 0.0000000 0.0000000 147.867 455.910 EM

98.223

2 -0.75554284D+04 ************ 0.6832101 162.352 436.346 EM

103.302

3 -0.74899891D+04 65.4393533 0.0086612 180.182 409.766 EM

112.053

4 -0.74564899D+04 33.4991297 0.0044725 196.528 386.295 EM

119.177

5 -0.74400832D+04 16.4067679 0.0022003 209.073 368.874 EM

124.053

6 -0.74337650D+04 6.3181621 0.0008492 218.001 356.947 EM

127.052

7 -0.74313143D+04 2.4507456 0.0003297 224.265 349.016 EM

128.719

8 -0.74302788D+04 1.0354801 0.0001393 228.681 343.828 EM

129.491

9 -0.74298081D+04 0.4707356 0.0000634 231.836 340.484 EM

129.679

TECHNICAL 8 OUTPUT FOR STARTING VALUE SET 206

ITER LOGLIKELIHOOD ABS CHANGE REL CHANGE CLASS COUNTS ALGORITHM

1 -0.16389521D+05 0.0000000 0.0000000 18.405 614.051 EM

69.545

2 -0.77604491D+04 8629.0715742 0.5264993 38.037 590.990 EM

72.974

3 -0.76736666D+04 86.7825404 0.0111827 59.405 553.445 EM

89.150

4 -0.76022106D+04 71.4559676 0.0093118 78.967 515.197 EM

107.837

5 -0.75540846D+04 48.1259668 0.0063305 94.662 481.666 EM

125.672

6 -0.75254362D+04 28.6484579 0.0037924 106.884 453.954 EM

141.162

7 -0.75080590D+04 17.3771310 0.0023091 116.370 431.840 EM

153.790

8 -0.74976510D+04 10.4080559 0.0013863 123.608 414.679 EM

163.712

9 -0.74915701D+04 6.0808849 0.0008110 129.023 401.582 EM

171.395

10 -0.74880250D+04 3.5450916 0.0004732 133.012 391.618 EM

177.370

11 -0.74859104D+04 2.1145748 0.0002824 135.916 383.991 EM

182.093

12 -0.74846082D+04 1.3022670 0.0001740 137.999 378.098 EM

185.903

13 -0.74837839D+04 0.8242864 0.0001101 139.460 373.500 EM

189.040

TECHNICAL 8 OUTPUT FOR STARTING VALUE SET 207

ITER LOGLIKELIHOOD ABS CHANGE REL CHANGE CLASS COUNTS ALGORITHM

1 -0.24339243D+05 0.0000000 0.0000000 313.811 382.695 EM

5.494

2 -0.76170933D+04 ************ 0.6870448 324.976 373.195 EM

3.829

3 -0.75325160D+04 84.5773176 0.0111036 329.238 369.373 EM

3.389

4 -0.75176641D+04 14.8519168 0.0019717 331.301 367.486 EM

3.213

5 -0.75156793D+04 1.9848449 0.0002640 332.722 366.169 EM

3.109

6 -0.75153034D+04 0.3759023 0.0000500 333.832 365.120 EM

3.049

TECHNICAL 8 OUTPUT FOR STARTING VALUE SET 208

ITER LOGLIKELIHOOD ABS CHANGE REL CHANGE CLASS COUNTS ALGORITHM

1 -0.23032184D+05 0.0000000 0.0000000 17.823 500.293 EM

183.883

2 -0.76159643D+04 ************ 0.6693338 14.636 483.449 EM

203.914

3 -0.75709166D+04 45.0477402 0.0059149 15.023 461.486 EM

225.491

4 -0.75528049D+04 18.1116654 0.0023923 15.838 440.443 EM

245.719

5 -0.75401235D+04 12.6814250 0.0016790 17.175 420.912 EM

263.913

6 -0.75297219D+04 10.4015125 0.0013795 19.376 403.000 EM

279.624

7 -0.75215353D+04 8.1866222 0.0010872 22.283 387.102 EM

292.615

8 -0.75154491D+04 6.0862756 0.0008092 25.691 373.408 EM

302.901

9 -0.75109725D+04 4.4765114 0.0005956 29.527 361.823 EM

310.650

10 -0.75076086D+04 3.3639608 0.0004479 33.749 352.112 EM

316.139

11 -0.75050068D+04 2.6017356 0.0003465 38.305 344.011 EM

319.684

12 -0.75029367D+04 2.0701005 0.0002758 43.131 337.275 EM

321.594

13 -0.75012381D+04 1.6986635 0.0002264 48.177 331.684 EM

322.138

14 -0.74997896D+04 1.4484338 0.0001931 53.419 327.040 EM

321.541

15 -0.74984954D+04 1.2942181 0.0001726 58.857 323.162 EM

319.981

16 -0.74972789D+04 1.2165338 0.0001622 64.511 319.890 EM

317.598

17 -0.74960784D+04 1.2004478 0.0001601 70.423 317.082 EM

314.495

18 -0.74948420D+04 1.2364660 0.0001649 76.648 314.614 EM

310.738

19 -0.74935208D+04 1.3211668 0.0001763 83.258 312.377 EM

306.365

20 -0.74920638D+04 1.4570566 0.0001944 90.339 310.278 EM

301.383

21 -0.74904118D+04 1.6519679 0.0002205 97.998 308.228 EM

295.773

22 -0.74884949D+04 1.9169100 0.0002559 106.358 306.141 EM

289.501

23 -0.74862343D+04 2.2605977 0.0003019 115.552 303.925 EM

282.522

24 -0.74835556D+04 2.6787078 0.0003578 125.712 301.479 EM

274.809

25 -0.74804150D+04 3.1406081 0.0004197 136.926 298.694 EM

266.381

26 -0.74768260D+04 3.5889742 0.0004798 149.189 295.472 EM

257.339

27 -0.74728629D+04 3.9630643 0.0005300 162.380 291.757 EM

247.863

28 -0.74686354D+04 4.2275157 0.0005657 176.288 287.535 EM

238.177

29 -0.74642538D+04 4.3815653 0.0005867 190.673 282.833 EM

228.494

30 -0.74598059D+04 4.4479819 0.0005959 205.315 277.699 EM

218.987

31 -0.74553548D+04 4.4510167 0.0005967 220.017 272.205 EM

209.777

32 -0.74509635D+04 4.3913167 0.0005890 234.576 266.462 EM

200.962

33 -0.74467327D+04 4.2308055 0.0005678 248.721 260.639 EM

192.640

34 -0.74428238D+04 3.9089475 0.0005249 262.102 254.966 EM

184.932

35 -0.74394283D+04 3.3955115 0.0004562 274.334 249.706 EM

177.960

36 -0.74338220D+04 5.6063127 0.0007536 308.908 236.721 FS

156.371

37 -0.74321847D+04 1.6373007 0.0002203 315.484 234.930 FS

151.586

38 -0.74310104D+04 1.1742567 0.0001580 318.275 234.508 EM

149.217

39 -0.74306722D+04 0.3382077 0.0000455 320.765 234.196 EM

147.039

TECHNICAL 8 OUTPUT FOR STARTING VALUE SET 209

ITER LOGLIKELIHOOD ABS CHANGE REL CHANGE CLASS COUNTS ALGORITHM

1 -0.23843438D+05 0.0000000 0.0000000 535.243 153.745 EM

13.013

2 -0.76731527D+04 ************ 0.6781860 511.316 167.623 EM

23.061

3 -0.76019783D+04 71.1744146 0.0092758 483.698 182.332 EM

35.970

4 -0.75526348D+04 49.3435147 0.0064909 453.155 197.632 EM

51.214

5 -0.75026453D+04 49.9894880 0.0066188 421.707 214.315 EM

65.978

6 -0.74611474D+04 41.4979073 0.0055311 395.453 228.730 EM

77.817

7 -0.74409006D+04 20.2468057 0.0027136 377.119 238.652 EM

86.229

8 -0.74336053D+04 7.2953256 0.0009804 365.137 244.823 EM

92.041

9 -0.74309378D+04 2.6674559 0.0003588 357.489 248.420 EM

96.090

10 -0.74298964D+04 1.0414140 0.0001401 352.644 250.382 EM

98.973

11 -0.74294596D+04 0.4368145 0.0000588 349.562 251.351 EM

101.086

TECHNICAL 8 OUTPUT FOR STARTING VALUE SET 210

ITER LOGLIKELIHOOD ABS CHANGE REL CHANGE CLASS COUNTS ALGORITHM

1 -0.18529697D+05 0.0000000 0.0000000 0.790 252.354 EM

448.856

2 -0.76221483D+04 ************ 0.5886523 3.459 270.298 EM

428.242

3 -0.75319290D+04 90.2192478 0.0118365 8.161 282.583 EM

411.257

4 -0.75122548D+04 19.6741916 0.0026121 13.140 290.963 EM

397.898

5 -0.75023422D+04 9.9126182 0.0013195 17.740 297.040 EM

387.220

6 -0.74964888D+04 5.8534206 0.0007802 22.068 301.234 EM

378.698

7 -0.74926126D+04 3.8761938 0.0005171 26.203 303.934 EM

371.863

8 -0.74898796D+04 2.7330152 0.0003648 30.109 305.560 EM

366.331

9 -0.74878991D+04 1.9805097 0.0002644 33.761 306.423 EM

361.817

10 -0.74864345D+04 1.4646178 0.0001956 37.155 306.736 EM

358.109

11 -0.74853316D+04 1.1028820 0.0001473 40.302 306.653 EM

355.045

12 -0.74844872D+04 0.8444225 0.0001128 43.216 306.283 EM

352.501

TECHNICAL 8 OUTPUT FOR STARTING VALUE SET 211

ITER LOGLIKELIHOOD ABS CHANGE REL CHANGE CLASS COUNTS ALGORITHM

1 -0.21509398D+05 0.0000000 0.0000000 7.441 474.748 EM

219.811

2 -0.76563293D+04 ************ 0.6440473 7.239 447.494 EM

247.268

3 -0.75798894D+04 76.4398906 0.0099839 7.107 421.431 EM

273.461

4 -0.75437248D+04 36.1646307 0.0047711 6.640 401.175 EM

294.185

5 -0.75274018D+04 16.3229483 0.0021638 6.254 385.356 EM

310.390

6 -0.75189126D+04 8.4891741 0.0011278 5.997 372.770 EM

323.233

7 -0.75142030D+04 4.7096240 0.0006264 5.840 362.912 EM

333.248

8 -0.75116535D+04 2.5495395 0.0003393 5.750 355.359 EM

340.891

9 -0.75103090D+04 1.3445228 0.0001790 5.699 349.662 EM

346.640

10 -0.75096023D+04 0.7066228 0.0000941 5.672 345.397 EM

350.931

TECHNICAL 8 OUTPUT FOR STARTING VALUE SET 212

ITER LOGLIKELIHOOD ABS CHANGE REL CHANGE CLASS COUNTS ALGORITHM

1 -0.28079984D+05 0.0000000 0.0000000 134.768 208.317 EM

358.915

2 -0.75642282D+04 ************ 0.7306185 140.385 205.808 EM

355.806

3 -0.75062985D+04 57.9297248 0.0076584 142.705 204.493 EM

354.802

4 -0.74952925D+04 11.0060136 0.0014662 143.872 203.290 EM

354.838

5 -0.74895576D+04 5.7348541 0.0007651 144.418 202.498 EM

355.083

6 -0.74861494D+04 3.4082689 0.0004551 144.554 202.135 EM

355.311

7 -0.74842010D+04 1.9484083 0.0002603 144.392 202.148 EM

355.461

8 -0.74831520D+04 1.0489527 0.0001402 144.014 202.462 EM

355.524

9 -0.74826195D+04 0.5325219 0.0000712 143.489 202.999 EM

355.512

TECHNICAL 8 OUTPUT FOR STARTING VALUE SET 213

ITER LOGLIKELIHOOD ABS CHANGE REL CHANGE CLASS COUNTS ALGORITHM

1 -0.20313064D+05 0.0000000 0.0000000 34.506 230.952 EM

436.541

2 -0.77870239D+04 ************ 0.6166495 31.915 235.761 EM

434.324

3 -0.76238674D+04 163.1564578 0.0209524 38.413 247.961 EM

415.626

4 -0.75298302D+04 94.0372208 0.0123346 45.003 256.251 EM

400.746

5 -0.75085941D+04 21.2361289 0.0028203 51.022 260.908 EM

390.070

6 -0.74977452D+04 10.8488517 0.0014449 56.421 263.642 EM

381.937

7 -0.74902983D+04 7.4469167 0.0009932 60.788 265.897 EM

375.315

8 -0.74862062D+04 4.0920717 0.0005463 64.024 268.258 EM

369.718

9 -0.74841849D+04 2.0213224 0.0002700 66.413 270.607 EM

364.980

10 -0.74831060D+04 1.0789681 0.0001442 68.263 272.733 EM

361.004

11 -0.74824538D+04 0.6521302 0.0000871 69.769 274.544 EM

357.686

TECHNICAL 8 OUTPUT FOR STARTING VALUE SET 214

ITER LOGLIKELIHOOD ABS CHANGE REL CHANGE CLASS COUNTS ALGORITHM

1 -0.28407826D+05 0.0000000 0.0000000 115.910 531.083 EM

55.007

2 -0.75959482D+04 ************ 0.7326107 145.213 491.326 EM

65.461

3 -0.75250362D+04 70.9119600 0.0093355 171.952 450.657 EM

79.391

4 -0.74783201D+04 46.7160698 0.0062081 194.619 415.869 EM

91.512

5 -0.74499264D+04 28.3937374 0.0037968 211.464 390.065 EM

100.472

6 -0.74372989D+04 12.6274972 0.0016950 222.969 372.531 EM

106.500

7 -0.74322818D+04 5.0170665 0.0006746 230.624 360.983 EM

110.393

8 -0.74302861D+04 1.9957748 0.0002685 235.677 353.479 EM

112.845

9 -0.74294859D+04 0.8001860 0.0001077 239.010 348.638 EM

114.352

TECHNICAL 8 OUTPUT FOR STARTING VALUE SET 215

ITER LOGLIKELIHOOD ABS CHANGE REL CHANGE CLASS COUNTS ALGORITHM

1 -0.21650673D+05 0.0000000 0.0000000 36.458 272.164 EM

393.378

2 -0.76332409D+04 ************ 0.6474363 30.917 293.125 EM

377.959

3 -0.75375213D+04 95.7196370 0.0125398 30.483 309.530 EM

361.987

4 -0.75204581D+04 17.0631370 0.0022638 31.122 321.703 EM

349.175

5 -0.75130759D+04 7.3822132 0.0009816 33.426 331.033 EM

337.541

6 -0.75036979D+04 9.3780813 0.0012482 37.523 337.865 EM

326.613

7 -0.74924287D+04 11.2691529 0.0015018 42.240 341.951 EM

317.809

8 -0.74858241D+04 6.6046457 0.0008815 46.067 343.641 EM

312.292

9 -0.74836210D+04 2.2030885 0.0002943 48.962 343.991 EM

309.047

10 -0.74828693D+04 0.7516532 0.0001004 51.355 343.770 EM

306.875

TECHNICAL 8 OUTPUT FOR STARTING VALUE SET 216

ITER LOGLIKELIHOOD ABS CHANGE REL CHANGE CLASS COUNTS ALGORITHM

1 -0.18605560D+05 0.0000000 0.0000000 557.846 136.174 EM

7.979

2 -0.76464872D+04 ************ 0.5890214 524.228 165.669 EM

12.103

3 -0.75926940D+04 53.7932761 0.0070350 491.058 195.981 EM

14.961

4 -0.75621291D+04 30.5648627 0.0040256 461.273 223.326 EM

17.401

5 -0.75395765D+04 22.5526030 0.0029823 433.913 247.793 EM

20.294

6 -0.75201089D+04 19.4676546 0.0025821 408.693 269.137 EM

24.170

7 -0.75024250D+04 17.6838391 0.0023515 386.015 286.790 EM

29.196

8 -0.74867381D+04 15.6869023 0.0020909 366.436 300.519 EM

35.045

9 -0.74739274D+04 12.8107020 0.0017111 350.237 310.458 EM

41.305

10 -0.74640657D+04 9.8616865 0.0013195 337.123 317.183 EM

47.694

11 -0.74565268D+04 7.5389592 0.0010100 326.403 321.646 EM

53.950

12 -0.74507121D+04 5.8146948 0.0007798 317.382 324.785 EM

59.833

13 -0.74462422D+04 4.4698543 0.0005999 309.555 327.268 EM

65.177

14 -0.74428104D+04 3.4318484 0.0004609 302.617 329.436 EM

69.948

15 -0.74401334D+04 2.6769609 0.0003597 296.385 331.420 EM

74.195

16 -0.74379929D+04 2.1405538 0.0002877 290.742 333.271 EM

77.987

17 -0.74362413D+04 1.7515418 0.0002355 285.604 335.009 EM

81.387

18 -0.74347843D+04 1.4569617 0.0001959 280.918 336.638 EM

84.444

19 -0.74335634D+04 1.2209428 0.0001642 276.656 338.147 EM

87.197

20 -0.74325436D+04 1.0198501 0.0001372 272.807 339.515 EM

89.677

21 -0.74317037D+04 0.8398685 0.0001130 269.370 340.718 EM

91.911

TECHNICAL 8 OUTPUT FOR STARTING VALUE SET 217

ITER LOGLIKELIHOOD ABS CHANGE REL CHANGE CLASS COUNTS ALGORITHM

1 -0.17217568D+05 0.0000000 0.0000000 321.212 237.092 EM

143.696

2 -0.76204318D+04 9597.1366381 0.5574037 289.395 239.462 EM

173.143

3 -0.75513108D+04 69.1209681 0.0090705 274.842 232.444 EM

194.715

4 -0.75279975D+04 23.3133091 0.0030873 268.943 221.873 EM

211.185

5 -0.75122182D+04 15.7793151 0.0020961 267.396 211.184 EM

223.420

6 -0.74996260D+04 12.5922340 0.0016762 268.808 202.039 EM

231.153

7 -0.74888313D+04 10.7946159 0.0014394 272.562 195.245 EM

234.193

8 -0.74781885D+04 10.6428556 0.0014212 277.827 191.422 EM

232.751

9 -0.74666723D+04 11.5161557 0.0015400 283.768 190.696 EM

227.537

10 -0.74554607D+04 11.2115904 0.0015016 289.886 192.131 EM

219.982

11 -0.74470964D+04 8.3643056 0.0011219 295.589 194.571 EM

211.840

12 -0.74421616D+04 4.9348567 0.0006627 300.350 197.363 EM

204.287

13 -0.74393986D+04 2.7629728 0.0003713 304.085 200.223 EM

197.692

14 -0.74376863D+04 1.7123084 0.0002302 306.988 203.016 EM

191.996

15 -0.74364769D+04 1.2093548 0.0001626 309.295 205.677 EM

187.028

16 -0.74355412D+04 0.9357985 0.0001258 311.196 208.176 EM

182.627

TECHNICAL 8 OUTPUT FOR STARTING VALUE SET 218

ITER LOGLIKELIHOOD ABS CHANGE REL CHANGE CLASS COUNTS ALGORITHM

1 -0.22063821D+05 0.0000000 0.0000000 87.590 165.743 EM

448.667

2 -0.77338516D+04 ************ 0.6494781 122.204 151.519 EM

428.277

3 -0.76515625D+04 82.2890467 0.0106401 154.733 144.677 EM

402.590

4 -0.76061021D+04 45.4604181 0.0059413 185.311 140.617 EM

376.071

5 -0.75702062D+04 35.8958638 0.0047194 213.616 136.460 EM

351.924

6 -0.75447734D+04 25.4328120 0.0033596 238.191 131.349 EM

332.460

7 -0.75276999D+04 17.0734764 0.0022630 258.607 126.367 EM

317.026

8 -0.75162529D+04 11.4470098 0.0015207 275.113 122.559 EM

304.328

9 -0.75087747D+04 7.4781983 0.0009949 288.116 120.232 EM

293.652

10 -0.75040016D+04 4.7731385 0.0006357 298.139 119.264 EM

284.597

11 -0.75009378D+04 3.0637792 0.0004083 305.747 119.412 EM

276.841

12 -0.74989053D+04 2.0325237 0.0002710 311.459 120.435 EM

270.106

13 -0.74974853D+04 1.4200285 0.0001894 315.709 122.128 EM

264.162

14 -0.74964268D+04 1.0584103 0.0001412 318.845 124.329 EM

258.826

15 -0.74955797D+04 0.8471006 0.0001130 321.135 126.912 EM

253.954

TECHNICAL 8 OUTPUT FOR STARTING VALUE SET 219

ITER LOGLIKELIHOOD ABS CHANGE REL CHANGE CLASS COUNTS ALGORITHM

1 -0.19469870D+05 0.0000000 0.0000000 2.507 481.863 EM

217.630

2 -0.77785564D+04 ************ 0.6004824 7.759 462.389 EM

231.852

3 -0.76931753D+04 85.3810488 0.0109765 15.234 447.222 EM

239.544

4 -0.76501212D+04 43.0541614 0.0055964 20.036 437.324 EM

244.640

5 -0.76132690D+04 36.8522040 0.0048172 23.256 429.923 EM

248.821

6 -0.75708124D+04 42.4565645 0.0055767 25.070 419.732 EM

257.199

7 -0.75360122D+04 34.8002649 0.0045966 25.634 405.912 EM

270.454

8 -0.75180625D+04 17.9496394 0.0023818 25.482 391.030 EM

285.487

9 -0.75094211D+04 8.6414270 0.0011494 24.963 377.134 EM

299.903

10 -0.75042098D+04 5.2112971 0.0006940 24.223 365.187 EM

312.589

11 -0.75006250D+04 3.5848114 0.0004777 23.362 355.371 EM

323.266

12 -0.74980455D+04 2.5794365 0.0003439 22.485 347.488 EM

332.027

13 -0.74961718D+04 1.8737270 0.0002499 21.708 341.226 EM

339.066

14 -0.74948509D+04 1.3208669 0.0001762 21.143 336.282 EM

344.575

15 -0.74939688D+04 0.8821252 0.0001177 20.859 332.391 EM

348.750

TECHNICAL 8 OUTPUT FOR STARTING VALUE SET 220

ITER LOGLIKELIHOOD ABS CHANGE REL CHANGE CLASS COUNTS ALGORITHM

1 -0.20771747D+05 0.0000000 0.0000000 144.767 272.571 EM

284.662

2 -0.76028745D+04 ************ 0.6339800 151.423 254.382 EM

296.196

3 -0.75259709D+04 76.9035332 0.0101151 154.291 240.675 EM

307.034

4 -0.74967588D+04 29.2121241 0.0038815 153.038 234.089 EM

314.873

5 -0.74885279D+04 8.2309318 0.0010979 150.072 231.381 EM

320.546

6 -0.74858677D+04 2.6601513 0.0003552 146.746 230.373 EM

324.881

7 -0.74845829D+04 1.2848554 0.0001716 143.498 230.201 EM

328.301

8 -0.74838153D+04 0.7675142 0.0001025 140.450 230.493 EM

331.057

TECHNICAL 8 OUTPUT FOR STARTING VALUE SET 221

ITER LOGLIKELIHOOD ABS CHANGE REL CHANGE CLASS COUNTS ALGORITHM

1 -0.18706955D+05 0.0000000 0.0000000 416.468 203.379 EM

82.153

2 -0.75846071D+04 ************ 0.5945568 371.753 234.572 EM

95.675

3 -0.74903240D+04 94.2831618 0.0124309 340.626 253.917 EM

107.457

4 -0.74568241D+04 33.4998597 0.0044724 326.363 262.644 EM

112.993

5 -0.74426562D+04 14.1679323 0.0019000 321.750 265.072 EM

115.178

6 -0.74360435D+04 6.6126869 0.0008885 321.913 264.343 EM

115.744

7 -0.74328872D+04 3.1563154 0.0004245 324.007 262.298 EM

115.695

8 -0.74313042D+04 1.5829884 0.0002130 326.632 259.872 EM

115.495

9 -0.74304434D+04 0.8608130 0.0001158 329.187 257.503 EM

115.311

TECHNICAL 8 OUTPUT FOR STARTING VALUE SET 222

ITER LOGLIKELIHOOD ABS CHANGE REL CHANGE CLASS COUNTS ALGORITHM

1 -0.25026193D+05 0.0000000 0.0000000 265.730 291.894 EM

144.377

2 -0.76584506D+04 ************ 0.6939826 268.407 310.557 EM

123.037

3 -0.75325346D+04 125.9160056 0.0164414 262.446 324.799 EM

114.755

4 -0.75068492D+04 25.6854380 0.0034099 252.478 334.091 EM

115.431

5 -0.74960471D+04 10.8020419 0.0014390 242.848 340.583 EM

118.569

6 -0.74903177D+04 5.7294398 0.0007643 234.858 345.410 EM

121.732

7 -0.74876330D+04 2.6847281 0.0003584 228.435 349.108 EM

124.457

8 -0.74863773D+04 1.2556114 0.0001677 223.169 351.960 EM

126.872

9 -0.74856918D+04 0.6855507 0.0000916 218.718 354.157 EM

129.124

TECHNICAL 8 OUTPUT FOR STARTING VALUE SET 223

ITER LOGLIKELIHOOD ABS CHANGE REL CHANGE CLASS COUNTS ALGORITHM

1 -0.26319500D+05 0.0000000 0.0000000 378.691 282.975 EM

40.334

2 -0.77244038D+04 ************ 0.7065140 379.625 289.109 EM

33.267

3 -0.76554486D+04 68.9551534 0.0089269 377.840 292.822 EM

31.338

4 -0.75997022D+04 55.7464399 0.0072819 375.187 294.917 EM

31.896

5 -0.75461395D+04 53.5626771 0.0070480 369.127 298.631 EM

34.242

6 -0.75167849D+04 29.3545832 0.0038900 361.045 302.091 EM

38.865

7 -0.74993635D+04 17.4214351 0.0023177 352.422 304.035 EM

45.543

8 -0.74811234D+04 18.2401093 0.0024322 343.042 306.347 EM

52.611

9 -0.74643744D+04 16.7490184 0.0022388 332.685 310.874 EM

58.441

10 -0.74538320D+04 10.5423951 0.0014124 322.502 316.300 EM

63.197

11 -0.74476165D+04 6.2154519 0.0008339 313.430 321.028 EM

67.542

12 -0.74435018D+04 4.1147593 0.0005525 305.529 324.869 EM

71.602

13 -0.74405468D+04 2.9550155 0.0003970 298.606 328.037 EM

75.357

14 -0.74382903D+04 2.2564954 0.0003033 292.478 330.714 EM

78.808

15 -0.74364846D+04 1.8056543 0.0002428 287.000 333.030 EM

81.970

16 -0.74349957D+04 1.4889172 0.0002002 282.069 335.070 EM

84.862

17 -0.74337493D+04 1.2463913 0.0001676 277.620 336.881 EM

87.499

18 -0.74327042D+04 1.0450554 0.0001406 273.620 338.482 EM

89.899

19 -0.74318375D+04 0.8667157 0.0001166 270.051 339.872 EM

92.077

TECHNICAL 8 OUTPUT FOR STARTING VALUE SET 224

ITER LOGLIKELIHOOD ABS CHANGE REL CHANGE CLASS COUNTS ALGORITHM

1 -0.25151990D+05 0.0000000 0.0000000 35.681 534.060 EM

132.260

2 -0.77354959D+04 ************ 0.6924499 49.361 514.741 EM

137.899

3 -0.75855711D+04 149.9247905 0.0193814 61.931 480.855 EM

159.214

4 -0.75288476D+04 56.7234502 0.0074778 70.741 455.165 EM

176.094

5 -0.75117159D+04 17.1317024 0.0022755 78.446 433.670 EM

189.884

6 -0.75016648D+04 10.0510876 0.0013381 86.082 414.351 EM

201.567

7 -0.74940638D+04 7.6010171 0.0010132 93.830 397.074 EM

211.096

8 -0.74880947D+04 5.9691672 0.0007965 101.674 381.967 EM

218.359

9 -0.74832489D+04 4.8457552 0.0006471 109.638 368.976 EM

223.386

10 -0.74789812D+04 4.2676937 0.0005703 117.856 357.833 EM

226.311

11 -0.74747046D+04 4.2766385 0.0005718 126.572 348.133 EM

227.295

12 -0.74698292D+04 4.8753234 0.0006522 136.028 339.484 EM

226.488

13 -0.74639894D+04 5.8398003 0.0007818 146.187 331.767 EM

224.046

14 -0.74575547D+04 6.4347010 0.0008621 156.482 325.292 EM

220.226

15 -0.74516567D+04 5.8980289 0.0007909 166.121 320.412 EM

215.467

16 -0.74470754D+04 4.5813484 0.0006148 174.632 317.074 EM

210.294

17 -0.74437440D+04 3.3313255 0.0004473 181.931 314.961 EM

205.107

18 -0.74413021D+04 2.4419353 0.0003281 188.133 313.751 EM

200.115

19 -0.74394524D+04 1.8497073 0.0002486 193.405 313.196 EM

195.400

20 -0.74380041D+04 1.4482932 0.0001947 197.912 313.113 EM

190.976

21 -0.74368387D+04 1.1654401 0.0001567 201.797 313.369 EM

186.834

22 -0.74358803D+04 0.9583544 0.0001289 205.178 313.866 EM

182.956

TECHNICAL 8 OUTPUT FOR STARTING VALUE SET 225

ITER LOGLIKELIHOOD ABS CHANGE REL CHANGE CLASS COUNTS ALGORITHM

1 -0.19529039D+05 0.0000000 0.0000000 22.579 639.120 EM

40.300

2 -0.77416286D+04 ************ 0.6035837 43.416 600.681 EM

57.903

3 -0.76292028D+04 112.4257620 0.0145222 76.894 543.100 EM

82.006

4 -0.75504237D+04 78.7790789 0.0103260 108.336 490.965 EM

102.699

5 -0.75068714D+04 43.5523135 0.0057682 134.689 448.927 EM

118.383

6 -0.74783558D+04 28.5156748 0.0037986 157.627 414.478 EM

129.895

7 -0.74566948D+04 21.6609866 0.0028965 176.968 387.162 EM

137.870

8 -0.74432935D+04 13.4012279 0.0017972 191.858 367.302 EM

142.840

9 -0.74366647D+04 6.6288697 0.0008906 202.737 353.702 EM

145.561

10 -0.74335744D+04 3.0902528 0.0004155 210.605 344.638 EM

146.757

11 -0.74320841D+04 1.4902788 0.0002005 216.342 338.697 EM

146.961

12 -0.74313136D+04 0.7705443 0.0001037 220.596 334.873 EM

146.531

TECHNICAL 8 OUTPUT FOR STARTING VALUE SET 226

ITER LOGLIKELIHOOD ABS CHANGE REL CHANGE CLASS COUNTS ALGORITHM

1 -0.20900846D+05 0.0000000 0.0000000 507.555 38.136 EM

156.309

2 -0.77759726D+04 ************ 0.6279590 504.794 28.569 EM

168.637

3 -0.77137561D+04 62.2164174 0.0080011 492.661 25.459 EM

183.879

4 -0.76537663D+04 59.9898818 0.0077770 479.221 24.903 EM

197.877

5 -0.75957286D+04 58.0376741 0.0075829 461.223 26.640 EM

214.137

6 -0.75582350D+04 37.4936206 0.0049361 439.512 29.951 EM

232.537

7 -0.75374767D+04 20.7582545 0.0027464 417.559 34.327 EM

250.113

8 -0.75244005D+04 13.0762228 0.0017348 397.360 39.616 EM

265.024

9 -0.75150443D+04 9.3561556 0.0012434 379.747 45.695 EM

276.558

10 -0.75081702D+04 6.8741319 0.0009147 364.938 52.427 EM

284.635

11 -0.75031175D+04 5.0527394 0.0006730 352.771 59.677 EM

289.552

12 -0.74993256D+04 3.7918256 0.0005054 342.883 67.342 EM

291.775

13 -0.74963316D+04 2.9939910 0.0003992 334.849 75.371 EM

291.780

14 -0.74937813D+04 2.5503450 0.0003402 328.254 83.779 EM

289.968

15 -0.74914066D+04 2.3747269 0.0003169 322.726 92.640 EM

286.634

16 -0.74889988D+04 2.4077621 0.0003214 317.935 102.079 EM

281.986

17 -0.74863933D+04 2.6055309 0.0003479 313.591 112.246 EM

276.163

18 -0.74834690D+04 2.9242531 0.0003906 309.437 123.281 EM

269.282

19 -0.74801600D+04 3.3090067 0.0004422 305.256 135.266 EM

261.478

20 -0.74764659D+04 3.6941605 0.0004939 300.886 148.180 EM

252.934

21 -0.74724472D+04 4.0187038 0.0005375 296.228 161.892 EM

243.881

22 -0.74682015D+04 4.2456985 0.0005682 291.240 176.193 EM

234.567

23 -0.74638299D+04 4.3715751 0.0005854 285.923 190.858 EM

225.218

24 -0.74594117D+04 4.4181599 0.0005919 280.306 205.687 EM

216.008

25 -0.74550019D+04 4.4097996 0.0005912 274.436 220.503 EM

207.062

26 -0.74506558D+04 4.3461105 0.0005830 268.402 235.120 EM

198.478

27 -0.74464691D+04 4.1866747 0.0005619 262.355 249.287 EM

190.358

28 -0.74426003D+04 3.8688172 0.0005196 256.507 262.665 EM

182.829

29 -0.74392390D+04 3.3613495 0.0004516 251.104 274.881 EM

176.014

30 -0.74365282D+04 2.7107842 0.0003644 246.369 285.628 EM

170.004

31 -0.74344999D+04 2.0282430 0.0002727 242.440 294.743 EM

164.817

32 -0.74330744D+04 1.4255260 0.0001917 239.360 302.238 EM

160.402

33 -0.74321121D+04 0.9623314 0.0001295 237.079 308.259 EM

156.662

TECHNICAL 8 OUTPUT FOR STARTING VALUE SET 227

ITER LOGLIKELIHOOD ABS CHANGE REL CHANGE CLASS COUNTS ALGORITHM

1 -0.30416389D+05 0.0000000 0.0000000 288.858 310.209 EM

102.933

2 -0.76459003D+04 ************ 0.7486256 284.756 302.601 EM

114.643

3 -0.75643479D+04 81.5523469 0.0106662 275.346 301.680 EM

124.974

4 -0.75079907D+04 56.3572076 0.0074504 267.426 303.678 EM

130.896

5 -0.74621005D+04 45.8901744 0.0061122 262.313 307.589 EM

132.097

6 -0.74398846D+04 22.2159262 0.0029772 258.735 312.377 EM

130.888

7 -0.74333981D+04 6.4864943 0.0008719 255.768 316.997 EM

129.236

8 -0.74312828D+04 2.1152888 0.0002846 253.185 321.062 EM

127.753

9 -0.74303521D+04 0.9307196 0.0001252 251.003 324.473 EM

126.525

TECHNICAL 8 OUTPUT FOR STARTING VALUE SET 228

ITER LOGLIKELIHOOD ABS CHANGE REL CHANGE CLASS COUNTS ALGORITHM

1 -0.20741945D+05 0.0000000 0.0000000 101.237 479.604 EM

121.158

2 -0.76122672D+04 ************ 0.6330013 108.484 447.427 EM

146.089

3 -0.75584859D+04 53.7813454 0.0070651 117.812 418.802 EM

165.386

4 -0.75299713D+04 28.5145883 0.0037725 127.231 395.662 EM

179.107

5 -0.75133159D+04 16.6554029 0.0022119 135.982 377.247 EM

188.770

6 -0.75028928D+04 10.4230809 0.0013873 143.818 362.847 EM

195.335

7 -0.74961338D+04 6.7589946 0.0009009 150.891 351.835 EM

199.274

8 -0.74916350D+04 4.4987671 0.0006001 157.506 343.493 EM

201.001

9 -0.74883307D+04 3.3043068 0.0004411 163.975 337.051 EM

200.974

10 -0.74853408D+04 2.9899497 0.0003993 170.603 331.775 EM

199.623

11 -0.74819794D+04 3.3613931 0.0004491 177.725 326.997 EM

197.278

12 -0.74777754D+04 4.2039765 0.0005619 185.724 322.135 EM

194.141

13 -0.74725803D+04 5.1950644 0.0006947 194.974 316.729 EM

190.296

14 -0.74666853D+04 5.8949897 0.0007889 205.710 310.532 EM

185.758

15 -0.74606855D+04 5.9998062 0.0008035 217.884 303.561 EM

180.555

16 -0.74550618D+04 5.6237516 0.0007538 231.121 296.058 EM

174.821

17 -0.74499477D+04 5.1141135 0.0006860 244.853 288.344 EM

168.803

18 -0.74453261D+04 4.6215624 0.0006203 258.479 280.727 EM

162.794

19 -0.74412508D+04 4.0753200 0.0005474 271.427 273.487 EM

157.086

20 -0.74378468D+04 3.4040142 0.0004575 283.214 266.870 EM

151.916

21 -0.74351968D+04 2.6499537 0.0003563 293.508 261.061 EM

147.431

22 -0.74332688D+04 1.9279991 0.0002593 302.177 256.154 EM

143.668

23 -0.74319366D+04 1.3322704 0.0001792 309.267 252.157 EM

140.576

24 -0.74310444D+04 0.8921550 0.0001200 314.943 249.003 EM

138.054

TECHNICAL 8 OUTPUT FOR STARTING VALUE SET 229

ITER LOGLIKELIHOOD ABS CHANGE REL CHANGE CLASS COUNTS ALGORITHM

1 -0.25758048D+05 0.0000000 0.0000000 75.686 376.950 EM

249.364

2 -0.77286848D+04 ************ 0.6999507 91.244 379.216 EM

231.540

3 -0.76442014D+04 84.4834013 0.0109311 99.033 364.769 EM

238.198

4 -0.75892374D+04 54.9640398 0.0071903 101.129 345.859 EM

255.012

5 -0.75289977D+04 60.2396858 0.0079375 99.679 332.348 EM

269.973

6 -0.74935794D+04 35.4183303 0.0047043 97.485 326.538 EM

277.977

7 -0.74845083D+04 9.0710634 0.0012105 95.892 324.690 EM

281.418

8 -0.74826430D+04 1.8653608 0.0002492 94.750 324.326 EM

282.924

9 -0.74820688D+04 0.5741518 0.0000767 93.808 324.567 EM

283.624

TECHNICAL 8 OUTPUT FOR STARTING VALUE SET 230

ITER LOGLIKELIHOOD ABS CHANGE REL CHANGE CLASS COUNTS ALGORITHM

1 -0.15622323D+05 0.0000000 0.0000000 627.935 61.893 EM

12.172

2 -0.77934021D+04 7828.9205769 0.5011368 612.159 60.551 EM

29.290

3 -0.76688242D+04 124.5779159 0.0159850 564.251 86.066 EM

51.683

4 -0.75831751D+04 85.6490954 0.0111685 521.548 106.898 EM

73.553

5 -0.75448113D+04 38.3638222 0.0050591 486.555 123.508 EM

91.937

6 -0.75182084D+04 26.6029132 0.0035260 455.753 139.292 EM

106.955

7 -0.74944313D+04 23.7770722 0.0031626 426.843 156.042 EM

119.115

8 -0.74704994D+04 23.9319506 0.0031933 399.779 173.717 EM

128.503

9 -0.74503225D+04 20.1768294 0.0027009 377.457 189.524 EM

135.019

10 -0.74391529D+04 11.1696622 0.0014992 361.310 201.699 EM

138.991

11 -0.74342863D+04 4.8665844 0.0006542 350.326 210.566 EM

141.107

12 -0.74321593D+04 2.1269808 0.0002861 343.038 216.978 EM

141.984

13 -0.74311561D+04 1.0032282 0.0001350 338.286 221.658 EM

142.057

14 -0.74306374D+04 0.5186660 0.0000698 335.251 225.133 EM

141.617

TECHNICAL 8 OUTPUT FOR STARTING VALUE SET 231

ITER LOGLIKELIHOOD ABS CHANGE REL CHANGE CLASS COUNTS ALGORITHM

1 -0.20813004D+05 0.0000000 0.0000000 422.658 18.470 EM

260.872

2 -0.78330524D+04 ************ 0.6236462 415.350 16.375 EM

270.276

3 -0.76797839D+04 153.2684532 0.0195669 405.170 17.455 EM

279.375

4 -0.75550583D+04 124.7256568 0.0162408 395.086 22.695 EM

284.219

5 -0.75106274D+04 44.4308409 0.0058809 386.936 30.255 EM

284.810

6 -0.75000637D+04 10.5636778 0.0014065 381.109 36.521 EM

284.371

7 -0.74964619D+04 3.6018151 0.0004802 377.014 41.651 EM

283.335

8 -0.74945961D+04 1.8658195 0.0002489 374.099 46.216 EM

281.685

9 -0.74933549D+04 1.2412027 0.0001656 371.994 50.440 EM

279.566

10 -0.74924220D+04 0.9329239 0.0001245 370.455 54.410 EM

277.135

TECHNICAL 8 OUTPUT FOR STARTING VALUE SET 232

ITER LOGLIKELIHOOD ABS CHANGE REL CHANGE CLASS COUNTS ALGORITHM

1 -0.24676220D+05 0.0000000 0.0000000 55.595 507.470 EM

138.935

2 -0.75617784D+04 ************ 0.6935601 70.795 474.570 EM

156.635

3 -0.75214564D+04 40.3220436 0.0053323 83.540 446.680 EM

171.781

4 -0.75054129D+04 16.0434519 0.0021330 94.753 423.472 EM

183.775

5 -0.74946090D+04 10.8038962 0.0014395 105.340 403.288 EM

193.372

6 -0.74858412D+04 8.7678262 0.0011699 115.822 385.499 EM

200.678

7 -0.74780535D+04 7.7877046 0.0010403 126.593 369.793 EM

205.614

8 -0.74703778D+04 7.6757452 0.0010264 137.968 355.859 EM

208.172

9 -0.74622656D+04 8.1121430 0.0010859 149.817 343.717 EM

208.466

10 -0.74543645D+04 7.9011081 0.0010588 161.284 333.910 EM

206.806

11 -0.74480979D+04 6.2665765 0.0008407 171.469 326.760 EM

203.771

12 -0.74438363D+04 4.2616684 0.0005722 180.064 321.925 EM

200.012

13 -0.74410035D+04 2.8327544 0.0003806 187.185 318.821 EM

195.993

14 -0.74390264D+04 1.9770653 0.0002657 193.086 316.948 EM

191.967

15 -0.74375624D+04 1.4640744 0.0001968 198.011 315.940 EM

188.049

16 -0.74364244D+04 1.1379216 0.0001530 202.168 315.542 EM

184.289

17 -0.74355076D+04 0.9168263 0.0001233 205.720 315.572 EM

180.708

TECHNICAL 8 OUTPUT FOR STARTING VALUE SET 233

ITER LOGLIKELIHOOD ABS CHANGE REL CHANGE CLASS COUNTS ALGORITHM

1 -0.25095450D+05 0.0000000 0.0000000 41.383 277.056 EM

383.561

2 -0.78234331D+04 ************ 0.6882529 30.107 287.377 EM

384.516

3 -0.77538491D+04 69.5840027 0.0088943 26.796 291.596 EM

383.608

4 -0.77038468D+04 50.0022806 0.0064487 27.001 293.214 EM

381.785

5 -0.76620704D+04 41.7763321 0.0054228 30.635 292.959 EM

378.407

6 -0.76202873D+04 41.7831349 0.0054532 35.883 294.785 EM

371.332

7 -0.75710473D+04 49.2400534 0.0064617 40.907 300.445 EM

360.648

8 -0.75277981D+04 43.2491536 0.0057124 45.164 306.402 EM

350.433

9 -0.75090497D+04 18.7483701 0.0024906 49.344 310.523 EM

342.134

10 -0.75028309D+04 6.2188738 0.0008282 53.884 312.752 EM

335.364

11 -0.74999300D+04 2.9008272 0.0003866 58.823 313.594 EM

329.584

12 -0.74980432D+04 1.8868698 0.0002516 64.101 313.528 EM

324.372

13 -0.74965665D+04 1.4766137 0.0001969 69.692 312.897 EM

319.411

14 -0.74952364D+04 1.3301223 0.0001774 75.621 311.914 EM

314.466

15 -0.74939071D+04 1.3293170 0.0001774 81.944 310.696 EM

309.360

16 -0.74924855D+04 1.4215911 0.0001897 88.742 309.301 EM

303.957

17 -0.74908971D+04 1.5883906 0.0002120 96.112 307.746 EM

298.142

18 -0.74890680D+04 1.8291569 0.0002442 104.170 306.017 EM

291.813

19 -0.74869186D+04 2.1493901 0.0002870 113.045 304.077 EM

284.879

20 -0.74843703D+04 2.5482829 0.0003404 122.867 301.860 EM

277.273

21 -0.74813668D+04 3.0035420 0.0004013 133.737 299.285 EM

268.978

22 -0.74779025D+04 3.4642956 0.0004631 145.680 296.264 EM

260.056

23 -0.74740353D+04 3.8671611 0.0005171 158.606 292.738 EM

250.656

24 -0.74698687D+04 4.1666095 0.0005575 172.322 288.690 EM

240.988

25 -0.74655169D+04 4.3518102 0.0005826 186.589 284.140 EM

231.271

26 -0.74610762D+04 4.4406910 0.0005948 201.175 279.136 EM

221.689

27 -0.74566163D+04 4.4599015 0.0005978 215.879 273.744 EM

212.377

28 -0.74521969D+04 4.4193943 0.0005927 230.502 268.064 EM

203.434

29 -0.74479044D+04 4.2924395 0.0005760 244.797 262.247 EM

194.956

30 -0.74438836D+04 4.0208458 0.0005399 258.433 256.509 EM

187.058

31 -0.74403239D+04 3.5596938 0.0004782 271.027 251.109 EM

179.864

32 -0.74373915D+04 2.9324288 0.0003941 282.231 246.295 EM

173.474

33 -0.74351534D+04 2.2380883 0.0003009 291.832 242.242 EM

167.927

34 -0.74335564D+04 1.5970027 0.0002148 299.790 239.023 EM

163.187

35 -0.74324690D+04 1.0873842 0.0001463 306.218 236.617 EM

159.165

36 -0.74317428D+04 0.7261942 0.0000977 311.323 234.932 EM

155.745

TECHNICAL 8 OUTPUT FOR STARTING VALUE SET 234

ITER LOGLIKELIHOOD ABS CHANGE REL CHANGE CLASS COUNTS ALGORITHM

1 -0.31411787D+05 0.0000000 0.0000000 94.895 385.537 EM

221.567

2 -0.77170480D+04 ************ 0.7543264 143.454 372.281 EM

186.264

3 -0.76073357D+04 109.7123013 0.0142169 179.189 354.220 EM

168.591

4 -0.75482457D+04 59.0900796 0.0077675 202.451 337.010 EM

162.538

5 -0.75181716D+04 30.0740424 0.0039842 215.289 323.210 EM

163.501

6 -0.75070354D+04 11.1362000 0.0014812 222.124 312.626 EM

167.251

7 -0.75021291D+04 4.9063195 0.0006536 225.946 303.739 EM

172.315

8 -0.74982453D+04 3.8838198 0.0005177 227.904 295.222 EM

178.874

9 -0.74939392D+04 4.3060927 0.0005743 228.325 286.134 EM

187.541

10 -0.74886460D+04 5.2931771 0.0007063 227.370 275.799 EM

198.831

11 -0.74819807D+04 6.6652984 0.0008901 225.299 263.792 EM

212.909

12 -0.74736932D+04 8.2875134 0.0011077 222.561 250.091 EM

229.348

13 -0.74640159D+04 9.6772834 0.0012948 219.748 235.405 EM

246.847

14 -0.74542445D+04 9.7713672 0.0013091 217.416 221.188 EM

263.396

15 -0.74462827D+04 7.9618053 0.0010681 215.865 208.755 EM

277.381

16 -0.74409455D+04 5.3372219 0.0007168 215.108 198.558 EM

288.334

17 -0.74377172D+04 3.2283712 0.0004339 215.013 190.394 EM

296.593

18 -0.74357915D+04 1.9256354 0.0002589 215.418 183.841 EM

302.742

19 -0.74345917D+04 1.1998493 0.0001614 216.178 178.485 EM

307.337

20 -0.74337845D+04 0.8071260 0.0001086 217.177 174.003 EM

310.820

TECHNICAL 8 OUTPUT FOR STARTING VALUE SET 235

ITER LOGLIKELIHOOD ABS CHANGE REL CHANGE CLASS COUNTS ALGORITHM

1 -0.17481627D+05 0.0000000 0.0000000 304.125 383.522 EM

14.353

2 -0.75285827D+04 9953.0438238 0.5693431 308.907 378.480 EM

14.613

3 -0.75112768D+04 17.3059107 0.0022987 312.689 373.380 EM

15.931

4 -0.75089297D+04 2.3470396 0.0003125 315.165 369.417 EM

17.418

5 -0.75080422D+04 0.8875532 0.0001182 316.602 366.328 EM

19.070

TECHNICAL 8 OUTPUT FOR STARTING VALUE SET 236

ITER LOGLIKELIHOOD ABS CHANGE REL CHANGE CLASS COUNTS ALGORITHM

1 -0.22900854D+05 0.0000000 0.0000000 395.830 111.728 EM

194.442

2 -0.76713962D+04 ************ 0.6650170 386.936 108.169 EM

206.895

3 -0.75763164D+04 95.0798412 0.0123941 374.908 112.593 EM

214.499

4 -0.75452629D+04 31.0534422 0.0040988 365.026 117.883 EM

219.092

5 -0.75208074D+04 24.4555674 0.0032412 355.975 122.846 EM

223.180

6 -0.74945123D+04 26.2950586 0.0034963 345.579 125.977 EM

230.444

7 -0.74728958D+04 21.6165399 0.0028843 333.457 126.397 EM

242.146

8 -0.74595260D+04 13.3697986 0.0017891 321.179 124.990 EM

255.831

9 -0.74509009D+04 8.6251056 0.0011563 309.941 122.721 EM

269.338

10 -0.74446712D+04 6.2296387 0.0008361 300.011 120.212 EM

281.778

11 -0.74400674D+04 4.6038093 0.0006184 291.330 117.878 EM

292.792

12 -0.74367789D+04 3.2885264 0.0004420 283.821 115.967 EM

302.213

13 -0.74345117D+04 2.2671631 0.0003049 277.401 114.555 EM

310.044

14 -0.74329557D+04 1.5560586 0.0002093 271.963 113.606 EM

316.430

15 -0.74318589D+04 1.0967713 0.0001476 267.384 113.031 EM

321.585

16 -0.74310570D+04 0.8019197 0.0001079 263.543 112.729 EM

325.728

TECHNICAL 8 OUTPUT FOR STARTING VALUE SET 237

ITER LOGLIKELIHOOD ABS CHANGE REL CHANGE CLASS COUNTS ALGORITHM

1 -0.21128425D+05 0.0000000 0.0000000 38.019 565.274 EM

98.707

2 -0.77796610D+04 ************ 0.6317917 52.890 552.947 EM

96.163

3 -0.76473493D+04 132.3117656 0.0170074 75.528 517.601 EM

108.871

4 -0.75623475D+04 85.0017809 0.0111152 92.913 483.447 EM

125.640

5 -0.75267828D+04 35.5647027 0.0047029 105.588 455.488 EM

140.923

6 -0.75087578D+04 18.0249596 0.0023948 115.214 433.285 EM

153.501

7 -0.74982627D+04 10.4950835 0.0013977 122.535 416.035 EM

163.430

8 -0.74920809D+04 6.1818710 0.0008244 128.035 402.817 EM

171.148

9 -0.74884150D+04 3.6658899 0.0004893 132.118 392.708 EM

177.174

10 -0.74861900D+04 2.2249636 0.0002971 135.114 384.930 EM

181.956

11 -0.74848001D+04 1.3898702 0.0001857 137.283 378.890 EM

185.827

12 -0.74839116D+04 0.8885627 0.0001187 138.821 374.157 EM

189.022

TECHNICAL 8 OUTPUT FOR STARTING VALUE SET 238

ITER LOGLIKELIHOOD ABS CHANGE REL CHANGE CLASS COUNTS ALGORITHM

1 -0.15872091D+05 0.0000000 0.0000000 9.224 676.063 EM

16.714

2 -0.77966473D+04 8075.4433084 0.5087826 6.225 661.781 EM

33.994

3 -0.77457575D+04 50.8897997 0.0065271 5.501 637.305 EM

59.193

4 -0.77013534D+04 44.4040973 0.0057327 5.520 609.068 EM

87.412

5 -0.76647508D+04 36.6025598 0.0047527 5.897 580.239 EM

115.864

6 -0.76358808D+04 28.8700367 0.0037666 6.555 551.947 EM

143.498

7 -0.76118498D+04 24.0309479 0.0031471 7.616 524.604 EM

169.780

8 -0.75911137D+04 20.7360870 0.0027242 9.165 498.594 EM

194.240

9 -0.75733478D+04 17.7659110 0.0023404 10.859 474.709 EM

216.432

10 -0.75592769D+04 14.0708822 0.0018579 12.217 453.528 EM

236.255

11 -0.75488172D+04 10.4597311 0.0013837 13.231 434.867 EM

253.902

12 -0.75409179D+04 7.8993075 0.0010464 14.081 418.321 EM

269.598

13 -0.75347404D+04 6.1775043 0.0008192 14.884 403.638 EM

283.478

14 -0.75298502D+04 4.8902060 0.0006490 15.698 390.745 EM

295.557

15 -0.75260514D+04 3.7987889 0.0005045 16.550 379.643 EM

305.807

16 -0.75232163D+04 2.8351010 0.0003767 17.458 370.295 EM

314.248

17 -0.75211923D+04 2.0240178 0.0002690 18.438 362.574 EM

320.988

18 -0.75197955D+04 1.3967543 0.0001857 19.511 356.281 EM

326.208

19 -0.75188430D+04 0.9525225 0.0001267 20.700 351.183 EM

330.117

TECHNICAL 8 OUTPUT FOR STARTING VALUE SET 239

ITER LOGLIKELIHOOD ABS CHANGE REL CHANGE CLASS COUNTS ALGORITHM

1 -0.18309232D+05 0.0000000 0.0000000 1.949 37.369 EM

662.681

2 -0.78474522D+04 ************ 0.5713937 3.169 31.265 EM

667.566

3 -0.78289191D+04 18.5331482 0.0023617 10.948 31.873 EM

659.178

4 -0.77797769D+04 49.1422234 0.0062770 30.820 35.367 EM

635.813

5 -0.77303552D+04 49.4216935 0.0063526 50.623 43.199 EM

608.178

6 -0.76917795D+04 38.5756860 0.0049902 70.531 52.803 EM

578.666

7 -0.76620271D+04 29.7524129 0.0038681 91.209 60.304 EM

550.487

8 -0.76411023D+04 20.9248197 0.0027310 112.585 65.248 EM

524.167

9 -0.76233966D+04 17.7056466 0.0023172 134.452 68.070 EM

499.479

10 -0.76064852D+04 16.9114314 0.0022184 156.875 69.214 EM

475.910

11 -0.75888597D+04 17.6254385 0.0023172 180.167 69.015 EM

452.818

12 -0.75689944D+04 19.8653741 0.0026177 204.331 67.581 EM

430.089

13 -0.75478764D+04 21.1179911 0.0027901 227.685 64.963 EM

409.353

14 -0.75306342D+04 17.2421475 0.0022844 248.245 61.455 EM

392.300

15 -0.75191095D+04 11.5246805 0.0015304 265.348 57.593 EM

379.059

16 -0.75117284D+04 7.3811572 0.0009817 279.043 53.807 EM

369.150

17 -0.75070601D+04 4.6683278 0.0006215 289.692 50.273 EM

362.035

18 -0.75041073D+04 2.9527386 0.0003933 297.822 47.022 EM

357.157

19 -0.75021764D+04 1.9308846 0.0002573 303.964 44.025 EM

354.011

20 -0.75008280D+04 1.3484405 0.0001797 308.568 41.241 EM

352.191

21 -0.74997953D+04 1.0326952 0.0001377 311.983 38.623 EM

351.394

22 -0.74989124D+04 0.8829451 0.0001177 314.470 36.139 EM

351.392

TECHNICAL 8 OUTPUT FOR STARTING VALUE SET 240

ITER LOGLIKELIHOOD ABS CHANGE REL CHANGE CLASS COUNTS ALGORITHM

1 -0.20610263D+05 0.0000000 0.0000000 12.379 11.173 EM

678.448

2 -0.78491794D+04 ************ 0.6191616 11.710 5.149 EM

685.141

3 -0.78391385D+04 10.0409374 0.0012792 12.673 6.326 EM

683.001

4 -0.78141278D+04 25.0107006 0.0031905 14.422 21.407 EM

666.171

5 -0.77530292D+04 61.0985517 0.0078190 17.310 46.170 EM

638.520

6 -0.77044817D+04 48.5475737 0.0062618 21.760 72.037 EM

608.203

7 -0.76645183D+04 39.9633787 0.0051870 27.524 97.404 EM

577.072

8 -0.76315295D+04 32.9887883 0.0043041 34.323 121.649 EM

546.028

9 -0.76026203D+04 28.9091974 0.0037881 42.087 144.101 EM

515.812

10 -0.75766131D+04 26.0071688 0.0034208 50.833 163.933 EM

487.235

11 -0.75541441D+04 22.4690199 0.0029656 60.492 180.576 EM

460.932

12 -0.75363041D+04 17.8399671 0.0023616 70.598 194.008 EM

437.394

13 -0.75229616D+04 13.3425190 0.0017704 80.623 204.457 EM

416.919

14 -0.75133354D+04 9.6262340 0.0012796 90.233 212.149 EM

399.618

15 -0.75065933D+04 6.7420597 0.0008973 99.283 217.344 EM

385.373

16 -0.75019367D+04 4.6565811 0.0006203 107.771 220.386 EM

373.844

17 -0.74986597D+04 3.2770126 0.0004368 115.777 221.662 EM

364.561

18 -0.74962186D+04 2.4411048 0.0003255 123.407 221.555 EM

357.038

19 -0.74942450D+04 1.9736580 0.0002633 130.753 220.396 EM

350.852

20 -0.74924988D+04 1.7461209 0.0002330 137.892 218.448 EM

345.660

21 -0.74908027D+04 1.6961755 0.0002264 144.904 215.910 EM

341.186

22 -0.74889832D+04 1.8194315 0.0002429 151.891 212.927 EM

337.182

23 -0.74868298D+04 2.1534669 0.0002876 159.007 209.596 EM

333.397

24 -0.74840787D+04 2.7510246 0.0003674 166.486 205.965 EM

329.549

25 -0.74804575D+04 3.6212045 0.0004839 174.648 202.027 EM

325.325

26 -0.74758173D+04 4.6401971 0.0006203 183.859 197.721 EM

320.420

27 -0.74703053D+04 5.5119954 0.0007373 194.410 192.957 EM

314.633

28 -0.74644026D+04 5.9027162 0.0007902 206.384 187.660 EM

307.956

29 -0.74586640D+04 5.7385961 0.0007688 219.549 181.853 EM

300.598

30 -0.74533678D+04 5.2962834 0.0007101 233.429 175.690 EM

292.881

31 -0.74485255D+04 4.8422890 0.0006497 247.469 169.412 EM

285.119

32 -0.74441316D+04 4.3938289 0.0005899 261.131 163.285 EM

277.584

33 -0.74402806D+04 3.8509994 0.0005173 273.913 157.563 EM

270.524

34 -0.74371057D+04 3.1749455 0.0004267 285.397 152.446 EM

264.156

35 -0.74324344D+04 4.6712628 0.0006281 315.662 139.428 FS

246.909

36 -0.74310307D+04 1.4036899 0.0001889 321.840 136.965 FS

243.195

37 -0.74309224D+04 0.1083435 0.0000146 327.974 133.524 FS

240.502

TECHNICAL 8 OUTPUT FOR STARTING VALUE SET 241

ITER LOGLIKELIHOOD ABS CHANGE REL CHANGE CLASS COUNTS ALGORITHM

1 -0.16791268D+05 0.0000000 0.0000000 313.061 63.445 EM

325.494

2 -0.78011537D+04 8990.1148076 0.5354041 315.602 63.132 EM

323.266

3 -0.76864029D+04 114.7508150 0.0147095 316.287 67.572 EM

318.141

4 -0.76088863D+04 77.5166057 0.0100849 311.886 69.672 EM

320.442

5 -0.75412173D+04 67.6689668 0.0088934 307.502 69.606 EM

324.892

6 -0.74993873D+04 41.8299978 0.0055468 307.467 69.355 EM

325.178

7 -0.74864480D+04 12.9393004 0.0017254 310.133 69.662 EM

322.205

8 -0.74834104D+04 3.0375965 0.0004057 313.436 70.320 EM

318.244

9 -0.74824713D+04 0.9390994 0.0001255 316.536 71.076 EM

314.388

TECHNICAL 8 OUTPUT FOR STARTING VALUE SET 242

ITER LOGLIKELIHOOD ABS CHANGE REL CHANGE CLASS COUNTS ALGORITHM

1 -0.17681757D+05 0.0000000 0.0000000 47.614 621.221 EM

33.166

2 -0.77542237D+04 9927.5329834 0.5614563 62.348 613.904 EM

25.747

3 -0.77228108D+04 31.4129245 0.0040511 79.333 596.430 EM

26.237

4 -0.76987091D+04 24.1017330 0.0031208 97.369 574.667 EM

29.964

5 -0.76740491D+04 24.6599440 0.0032031 114.946 550.126 EM

36.928

6 -0.76464184D+04 27.6306754 0.0036005 130.762 522.828 EM

48.410

7 -0.76081656D+04 38.2528243 0.0050027 143.006 491.403 EM

67.591

8 -0.75544805D+04 53.6851024 0.0070562 151.469 460.478 EM

90.053

9 -0.75157805D+04 38.7000578 0.0051228 160.200 435.997 EM

105.802

10 -0.74994276D+04 16.3528588 0.0021758 168.241 417.942 EM

115.816

11 -0.74924492D+04 6.9784302 0.0009305 174.417 404.708 EM

122.875

12 -0.74889537D+04 3.4955062 0.0004665 178.781 394.884 EM

128.336

13 -0.74869913D+04 1.9624004 0.0002620 181.721 387.475 EM

132.804

14 -0.74858090D+04 1.1822343 0.0001579 183.598 381.805 EM

136.597

15 -0.74850613D+04 0.7477059 0.0000999 184.687 377.412 EM

139.901

TECHNICAL 8 OUTPUT FOR STARTING VALUE SET 243

ITER LOGLIKELIHOOD ABS CHANGE REL CHANGE CLASS COUNTS ALGORITHM

1 -0.21527824D+05 0.0000000 0.0000000 24.933 241.330 EM

435.737

2 -0.77436091D+04 ************ 0.6402976 35.925 242.049 EM

424.027

3 -0.76659904D+04 77.6187265 0.0100236 50.995 252.960 EM

398.046

4 -0.75966031D+04 69.3872781 0.0090513 63.041 270.006 EM

368.953

5 -0.75314636D+04 65.1395585 0.0085748 70.117 286.027 EM

345.855

6 -0.74979584D+04 33.5051625 0.0044487 73.289 297.318 EM

331.394

7 -0.74880897D+04 9.8687466 0.0013162 74.684 305.225 EM

322.091

8 -0.74846391D+04 3.4506061 0.0004608 75.452 311.135 EM

315.413

9 -0.74830129D+04 1.6261359 0.0002173 75.966 315.686 EM

310.348

10 -0.74821908D+04 0.8221434 0.0001099 76.357 319.238 EM

306.406

TECHNICAL 8 OUTPUT FOR STARTING VALUE SET 244

ITER LOGLIKELIHOOD ABS CHANGE REL CHANGE CLASS COUNTS ALGORITHM

1 -0.22460826D+05 0.0000000 0.0000000 323.951 45.328 EM

332.721

2 -0.76057327D+04 ************ 0.6613779 330.836 40.869 EM

330.295

3 -0.75317480D+04 73.9847345 0.0097275 331.911 43.426 EM

326.663

4 -0.75096414D+04 22.1066062 0.0029351 329.695 50.948 EM

321.357

5 -0.75021588D+04 7.4825769 0.0009964 326.085 61.347 EM

314.568

6 -0.74972805D+04 4.8783144 0.0006503 322.025 73.264 EM

306.711

7 -0.74930346D+04 4.2459056 0.0005663 317.867 85.983 EM

298.150

8 -0.74891288D+04 3.9058039 0.0005213 313.710 99.185 EM

289.105

9 -0.74853783D+04 3.7505195 0.0005008 309.536 112.767 EM

279.698

10 -0.74816121D+04 3.7661482 0.0005031 305.276 126.721 EM

270.004

11 -0.74777145D+04 3.8976416 0.0005210 300.849 141.045 EM

260.105

12 -0.74736419D+04 4.0726169 0.0005446 296.189 155.701 EM

250.110

13 -0.74694130D+04 4.2288060 0.0005658 291.254 170.604 EM

240.142

14 -0.74650802D+04 4.3328468 0.0005801 286.031 185.644 EM

230.324

15 -0.74606979D+04 4.3823289 0.0005870 280.528 200.713 EM

220.759

16 -0.74563093D+04 4.3885949 0.0005882 274.776 215.704 EM

211.520

17 -0.74519592D+04 4.3500416 0.0005834 268.841 230.489 EM

202.670

18 -0.74477281D+04 4.2311075 0.0005678 262.849 244.866 EM

194.285

19 -0.74437592D+04 3.9689098 0.0005329 256.994 258.535 EM

186.471

20 -0.74402413D+04 3.5179167 0.0004726 251.516 271.132 EM

179.352

21 -0.74373394D+04 2.9019446 0.0003900 246.648 282.327 EM

173.025

22 -0.74351203D+04 2.2190262 0.0002984 242.555 291.916 EM

167.529

23 -0.74335331D+04 1.5872765 0.0002135 239.304 299.865 EM

162.831

24 -0.74324494D+04 1.0836100 0.0001458 236.869 306.288 EM

158.843

25 -0.74317242D+04 0.7252451 0.0000976 235.159 311.390 EM

155.450

TECHNICAL 8 OUTPUT FOR STARTING VALUE SET 245

ITER LOGLIKELIHOOD ABS CHANGE REL CHANGE CLASS COUNTS ALGORITHM

1 -0.19634996D+05 0.0000000 0.0000000 76.536 41.472 EM

583.992

2 -0.78100963D+04 ************ 0.6022359 81.319 40.270 EM

580.411

3 -0.77174561D+04 92.6401874 0.0118616 88.772 59.252 EM

553.976

4 -0.76684714D+04 48.9847859 0.0063473 93.495 77.717 EM

530.788

5 -0.76505417D+04 17.9296461 0.0023381 96.359 95.312 EM

510.329

6 -0.76366633D+04 13.8784208 0.0018140 98.076 113.927 EM

489.997

7 -0.76229966D+04 13.6667423 0.0017896 98.854 133.484 EM

469.663

8 -0.76092485D+04 13.7480326 0.0018035 98.768 153.703 EM

449.529

9 -0.75949225D+04 14.3260506 0.0018827 97.852 174.719 EM

429.430

10 -0.75787491D+04 16.1734072 0.0021295 96.069 196.943 EM

408.988

11 -0.75596602D+04 19.0888711 0.0025187 93.372 219.876 EM

388.752

12 -0.75405011D+04 19.1591237 0.0025344 89.961 241.459 EM

370.580

13 -0.75261051D+04 14.3959955 0.0019092 86.385 260.188 EM

355.427

14 -0.75168124D+04 9.2927197 0.0012347 83.184 275.745 EM

343.071

15 -0.75109674D+04 5.8449345 0.0007776 80.616 288.333 EM

333.051

16 -0.75073049D+04 3.6625449 0.0004876 78.721 298.358 EM

324.921

17 -0.75050077D+04 2.2971938 0.0003060 77.444 306.281 EM

318.275

18 -0.75035530D+04 1.4546422 0.0001938 76.706 312.527 EM

312.767

19 -0.75026160D+04 0.9370223 0.0001249 76.430 317.451 EM

308.119

TECHNICAL 8 OUTPUT FOR STARTING VALUE SET 246

ITER LOGLIKELIHOOD ABS CHANGE REL CHANGE CLASS COUNTS ALGORITHM

1 -0.16042861D+05 0.0000000 0.0000000 161.036 5.868 EM

535.096

2 -0.77604624D+04 8282.3987456 0.5162669 174.384 4.580 EM

523.036

3 -0.76477751D+04 112.6872241 0.0145207 195.437 5.337 EM

501.225

4 -0.75869983D+04 60.7768574 0.0079470 215.766 11.213 EM

475.021

5 -0.75372269D+04 49.7713388 0.0065601 234.065 21.301 EM

446.634

6 -0.75141349D+04 23.0920336 0.0030637 248.802 28.871 EM

424.327

7 -0.75035658D+04 10.5691421 0.0014066 261.090 35.150 EM

405.760

8 -0.74970130D+04 6.5527509 0.0008733 271.462 40.540 EM

389.998

9 -0.74926500D+04 4.3629832 0.0005820 280.268 45.185 EM

376.547

10 -0.74896278D+04 3.0222311 0.0004034 287.778 49.196 EM

365.025

11 -0.74874804D+04 2.1474051 0.0002867 294.206 52.663 EM

355.131

12 -0.74859295D+04 1.5508950 0.0002071 299.719 55.659 EM

346.622

13 -0.74847949D+04 1.1345749 0.0001516 304.458 58.247 EM

339.295

14 -0.74839541D+04 0.8408019 0.0001123 308.544 60.484 EM

332.972

TECHNICAL 8 OUTPUT FOR STARTING VALUE SET 247

ITER LOGLIKELIHOOD ABS CHANGE REL CHANGE CLASS COUNTS ALGORITHM

1 -0.15588902D+05 0.0000000 0.0000000 36.739 513.456 EM

151.806

2 -0.76335580D+04 7955.3444867 0.5103210 26.148 499.285 EM

176.567

3 -0.75908121D+04 42.7458602 0.0055997 23.942 477.705 EM

200.353

4 -0.75706151D+04 20.1970659 0.0026607 23.545 455.992 EM

222.463

5 -0.75569993D+04 13.6157856 0.0017985 23.545 435.810 EM

242.646

6 -0.75468219D+04 10.1773553 0.0013467 23.763 417.200 EM

261.037

7 -0.75383793D+04 8.4426145 0.0011187 24.364 399.792 EM

277.844

8 -0.75300692D+04 8.3101154 0.0011024 25.788 383.035 EM

293.177

9 -0.75189439D+04 11.1253184 0.0014775 28.814 366.385 EM

306.801

10 -0.75034846D+04 15.4593088 0.0020560 33.373 350.999 EM

317.628

11 -0.74916591D+04 11.8254662 0.0015760 37.840 339.264 EM

324.896

12 -0.74865845D+04 5.0746525 0.0006774 41.447 331.100 EM

329.453

13 -0.74845771D+04 2.0073523 0.0002681 44.484 325.158 EM

332.357

14 -0.74836199D+04 0.9571866 0.0001279 47.191 320.544 EM

334.264

TECHNICAL 8 OUTPUT FOR STARTING VALUE SET 248

ITER LOGLIKELIHOOD ABS CHANGE REL CHANGE CLASS COUNTS ALGORITHM

1 -0.14071422D+05 0.0000000 0.0000000 138.783 74.769 EM

488.448

2 -0.77387827D+04 6332.6392148 0.4500355 147.031 100.644 EM

454.324

3 -0.75791454D+04 159.6372588 0.0206282 172.946 123.510 EM

405.544

4 -0.74810853D+04 98.0600864 0.0129381 193.873 135.474 EM

372.653

5 -0.74461721D+04 34.9131993 0.0046669 206.556 139.949 EM

355.495

6 -0.74362494D+04 9.9227726 0.0013326 214.703 141.458 EM

345.839

7 -0.74326424D+04 3.6069123 0.0004850 220.332 141.791 EM

339.877

8 -0.74311715D+04 1.4709277 0.0001979 224.365 141.523 EM

336.112

9 -0.74305329D+04 0.6385680 0.0000859 227.327 140.891 EM

333.781

TECHNICAL 8 OUTPUT FOR STARTING VALUE SET 249

ITER LOGLIKELIHOOD ABS CHANGE REL CHANGE CLASS COUNTS ALGORITHM

1 -0.14666439D+05 0.0000000 0.0000000 325.017 222.580 EM

154.403

2 -0.74854948D+04 7180.9442876 0.4896174 316.684 234.493 EM

150.822

3 -0.74650716D+04 20.4232161 0.0027284 309.129 244.512 EM

148.359

4 -0.74564229D+04 8.6486570 0.0011585 301.788 254.786 EM

145.426

5 -0.74496534D+04 6.7694920 0.0009079 294.385 265.751 EM

141.864

6 -0.74440583D+04 5.5950817 0.0007511 287.020 276.988 EM

137.992

7 -0.74395760D+04 4.4823584 0.0006021 279.967 287.800 EM

134.233

8 -0.74362030D+04 3.3729752 0.0004534 273.511 297.579 EM

130.910

9 -0.74338302D+04 2.3728086 0.0003191 267.842 305.984 EM

128.175

10 -0.74322350D+04 1.5952002 0.0002146 263.028 312.940 EM

126.032

11 -0.74311775D+04 1.0575211 0.0001423 259.045 318.556 EM

124.399

12 -0.74304704D+04 0.7070115 0.0000951 255.814 323.018 EM

123.168

TECHNICAL 8 OUTPUT FOR STARTING VALUE SET 250

ITER LOGLIKELIHOOD ABS CHANGE REL CHANGE CLASS COUNTS ALGORITHM

1 -0.21059062D+05 0.0000000 0.0000000 260.884 370.717 EM

70.399

2 -0.76792939D+04 ************ 0.6353449 254.876 342.341 EM

104.783

3 -0.76112068D+04 68.0870434 0.0088663 247.815 317.809 EM

136.376

4 -0.75737917D+04 37.4151407 0.0049158 240.151 298.890 EM

162.959

5 -0.75461623D+04 27.6293523 0.0036480 232.126 285.861 EM

184.013

6 -0.75215929D+04 24.5694594 0.0032559 224.584 278.659 EM

198.757

7 -0.74968921D+04 24.7007338 0.0032840 220.616 275.370 EM

206.014

8 -0.74752957D+04 21.5964863 0.0028807 223.462 272.886 EM

205.653

9 -0.74611758D+04 14.1198168 0.0018889 232.513 269.533 EM

199.954

10 -0.74526412D+04 8.5346774 0.0011439 244.707 265.259 EM

192.034

11 -0.74465563D+04 6.0848504 0.0008165 257.753 260.404 EM

183.843

12 -0.74417676D+04 4.7887019 0.0006431 270.404 255.370 EM

176.226

13 -0.74380440D+04 3.7236423 0.0005004 281.941 250.560 EM

169.499

14 -0.74353086D+04 2.7354068 0.0003678 291.960 246.301 EM

163.739

15 -0.74334253D+04 1.8832462 0.0002533 300.315 242.782 EM

158.902

16 -0.74321947D+04 1.2306243 0.0001656 307.069 240.055 EM

154.875

17 -0.74314116D+04 0.7831390 0.0001054 312.415 238.068 EM

151.518

TECHNICAL 8 OUTPUT FOR STARTING VALUE SET 251

ITER LOGLIKELIHOOD ABS CHANGE REL CHANGE CLASS COUNTS ALGORITHM

1 -0.23762534D+05 0.0000000 0.0000000 72.708 121.844 EM

507.448

2 -0.77204579D+04 ************ 0.6750996 82.678 126.686 EM

492.636

3 -0.76323950D+04 88.0629439 0.0114064 108.653 126.751 EM

466.596

4 -0.75566250D+04 75.7699646 0.0099274 132.971 127.347 EM

441.683

5 -0.75144701D+04 42.1548891 0.0055785 147.549 131.897 EM

422.554

6 -0.74984543D+04 16.0158014 0.0021313 156.387 137.452 EM

408.161

7 -0.74915400D+04 6.9143600 0.0009221 162.417 142.236 EM

397.347

8 -0.74880473D+04 3.4927127 0.0004662 166.941 145.928 EM

389.130

9 -0.74860710D+04 1.9762166 0.0002639 170.567 148.652 EM

382.781

10 -0.74848621D+04 1.2089874 0.0001615 173.601 150.603 EM

377.796

11 -0.74840863D+04 0.7757301 0.0001036 176.212 151.954 EM

373.834

TECHNICAL 8 OUTPUT FOR STARTING VALUE SET 252

ITER LOGLIKELIHOOD ABS CHANGE REL CHANGE CLASS COUNTS ALGORITHM

1 -0.21080682D+05 0.0000000 0.0000000 193.895 72.786 EM

435.319

2 -0.75219323D+04 ************ 0.6431836 208.544 79.790 EM

413.666

3 -0.74964954D+04 25.4369672 0.0033817 215.734 86.437 EM

399.830

4 -0.74906985D+04 5.7969025 0.0007733 219.505 91.943 EM

390.552

5 -0.74884926D+04 2.2058843 0.0002945 221.336 96.591 EM

384.073

6 -0.74873997D+04 1.0928897 0.0001459 221.947 100.659 EM

379.394

7 -0.74867526D+04 0.6471384 0.0000864 221.756 104.322 EM

375.923

TECHNICAL 8 OUTPUT FOR STARTING VALUE SET 253

ITER LOGLIKELIHOOD ABS CHANGE REL CHANGE CLASS COUNTS ALGORITHM

1 -0.18567227D+05 0.0000000 0.0000000 346.780 311.312 EM

43.907

2 -0.77382971D+04 ************ 0.5832282 345.394 301.824 EM

54.782

3 -0.76596617D+04 78.6354057 0.0101618 344.927 291.941 EM

65.132

4 -0.76101430D+04 49.5186350 0.0064649 341.710 285.683 EM

74.607

5 -0.75653672D+04 44.7757960 0.0058837 335.278 284.508 EM

82.213

6 -0.75135809D+04 51.7862837 0.0068452 324.360 291.182 EM

86.458

7 -0.74689893D+04 44.5916407 0.0059348 311.263 302.329 EM

88.408

8 -0.74454616D+04 23.5276656 0.0031500 299.842 312.450 EM

89.707

9 -0.74376554D+04 7.8062022 0.0010485 290.985 320.107 EM

90.908

10 -0.74350221D+04 2.6333336 0.0003541 284.116 325.648 EM

92.236

11 -0.74336048D+04 1.4172825 0.0001906 278.576 329.745 EM

93.679

12 -0.74325605D+04 1.0442975 0.0001405 273.944 332.896 EM

95.160

13 -0.74317166D+04 0.8439663 0.0001135 269.988 335.396 EM

96.616

TECHNICAL 8 OUTPUT FOR STARTING VALUE SET 254

ITER LOGLIKELIHOOD ABS CHANGE REL CHANGE CLASS COUNTS ALGORITHM

1 -0.23491968D+05 0.0000000 0.0000000 415.635 26.527 EM

259.838

2 -0.77670564D+04 ************ 0.6693740 412.358 41.268 EM

248.374

3 -0.76461665D+04 120.8898347 0.0155644 400.491 51.214 EM

250.295

4 -0.75821070D+04 64.0595514 0.0083780 386.154 56.379 EM

259.467

5 -0.75317659D+04 50.3410750 0.0066395 368.774 58.828 EM

274.398

6 -0.74931303D+04 38.6355690 0.0051297 350.039 61.336 EM

290.625

7 -0.74665636D+04 26.5667420 0.0035455 332.958 65.092 EM

303.950

8 -0.74515257D+04 15.0378604 0.0020140 319.252 69.076 EM

313.672

9 -0.74444654D+04 7.0602891 0.0009475 308.712 72.769 EM

320.520

10 -0.74408232D+04 3.6422079 0.0004893 300.426 76.208 EM

325.366

11 -0.74384264D+04 2.3968558 0.0003221 293.614 79.432 EM

328.954

12 -0.74365888D+04 1.8376152 0.0002470 287.785 82.439 EM

331.776

13 -0.74350880D+04 1.5007838 0.0002018 282.660 85.223 EM

334.117

14 -0.74338328D+04 1.2551725 0.0001688 278.093 87.785 EM

336.123

15 -0.74327782D+04 1.0546031 0.0001419 274.009 90.129 EM

337.862

16 -0.74319003D+04 0.8779359 0.0001181 270.373 92.266 EM

339.361

TECHNICAL 8 OUTPUT FOR STARTING VALUE SET 255

ITER LOGLIKELIHOOD ABS CHANGE REL CHANGE CLASS COUNTS ALGORITHM

1 -0.17900031D+05 0.0000000 0.0000000 428.055 227.307 EM

46.638

2 -0.75670266D+04 ************ 0.5772618 388.972 249.847 EM

63.181

3 -0.74951470D+04 71.8795559 0.0094990 361.426 265.635 EM

74.938

4 -0.74646520D+04 30.4949751 0.0040686 345.927 273.916 EM

82.157

5 -0.74493202D+04 15.3318255 0.0020539 337.801 277.068 EM

87.131

6 -0.74404876D+04 8.8325820 0.0011857 334.305 276.955 EM

90.739

7 -0.74357710D+04 4.7166654 0.0006339 333.629 275.009 EM

93.362

8 -0.74333737D+04 2.3972789 0.0003224 334.382 272.232 EM

95.386

9 -0.74320427D+04 1.3310042 0.0001791 335.714 269.217 EM

97.070

10 -0.74311850D+04 0.8576793 0.0001154 337.178 266.279 EM

98.542

TECHNICAL 8 OUTPUT FOR STARTING VALUE SET 256

ITER LOGLIKELIHOOD ABS CHANGE REL CHANGE CLASS COUNTS ALGORITHM

1 -0.20120929D+05 0.0000000 0.0000000 76.108 625.892 EM

0.000

2 -0.78609973D+04 ************ 0.6093124 70.959 631.041 EM

0.000

3 -0.78069629D+04 54.0344409 0.0068737 93.567 608.433 EM

0.000

4 -0.77375959D+04 69.3669866 0.0088853 135.677 566.323 EM

0.000

5 -0.76504478D+04 87.1481404 0.0112629 180.383 521.617 EM

0.000

6 -0.75953741D+04 55.0736829 0.0071988 216.914 485.084 EM

0.002

7 -0.75659730D+04 29.4011194 0.0038709 246.077 455.755 EM

0.168

8 -0.75412439D+04 24.7290476 0.0032685 268.987 428.836 EM

4.177

9 -0.75107411D+04 30.5028738 0.0040448 285.328 404.910 EM

11.762

10 -0.74883683D+04 22.3727876 0.0029788 295.366 384.869 EM

21.766

11 -0.74720507D+04 16.3175755 0.0021791 300.031 369.747 EM

32.222

12 -0.74610477D+04 11.0029965 0.0014726 300.968 358.574 EM

42.458

13 -0.74525743D+04 8.4734465 0.0011357 299.467 350.599 EM

51.934

14 -0.74461980D+04 6.3762109 0.0008556 296.397 345.541 EM

60.063

15 -0.74418177D+04 4.3803273 0.0005883 292.444 342.831 EM

66.725

16 -0.74388465D+04 2.9711922 0.0003993 288.134 341.672 EM

72.194

17 -0.74366796D+04 2.1669311 0.0002913 283.795 341.423 EM

76.782

18 -0.74349979D+04 1.6816876 0.0002261 279.612 341.681 EM

80.707

19 -0.74336541D+04 1.3438513 0.0001807 275.694 342.194 EM

84.112

20 -0.74325736D+04 1.0804457 0.0001453 272.105 342.803 EM

87.092

21 -0.74317126D+04 0.8610308 0.0001158 268.882 343.402 EM

89.715

TECHNICAL 8 OUTPUT FOR STARTING VALUE SET 257

ITER LOGLIKELIHOOD ABS CHANGE REL CHANGE CLASS COUNTS ALGORITHM

1 -0.24618192D+05 0.0000000 0.0000000 253.599 76.963 EM

371.438

2 -0.75573689D+04 ************ 0.6930169 280.014 65.266 EM

356.720

3 -0.75240251D+04 33.3438477 0.0044121 294.242 65.736 EM

342.022

4 -0.75108251D+04 13.1999686 0.0017544 300.833 73.076 EM

328.091

5 -0.75021313D+04 8.6938463 0.0011575 302.511 84.844 EM

314.645

6 -0.74948943D+04 7.2369933 0.0009647 301.212 99.341 EM

301.448

7 -0.74882789D+04 6.6153680 0.0008826 298.170 115.416 EM

288.414

8 -0.74820944D+04 6.1845359 0.0008259 294.114 132.299 EM

275.587

9 -0.74763079D+04 5.7864801 0.0007734 289.463 149.441 EM

263.096

10 -0.74709068D+04 5.4010445 0.0007224 284.447 166.463 EM

251.090

11 -0.74658410D+04 5.0658444 0.0006781 279.176 183.158 EM

239.665

12 -0.74610265D+04 4.8145041 0.0006449 273.700 199.444 EM

228.856

13 -0.74563876D+04 4.6388407 0.0006217 268.052 215.290 EM

218.658

14 -0.74518982D+04 4.4894282 0.0006021 262.298 230.632 EM

209.070

15 -0.74476099D+04 4.2883018 0.0005755 256.565 245.312 EM

200.123

16 -0.74436561D+04 3.9537750 0.0005309 251.049 259.064 EM

191.887

17 -0.74402115D+04 3.4446308 0.0004628 245.980 271.569 EM

184.451

18 -0.74374140D+04 2.7975059 0.0003760 241.570 282.550 EM

177.880

19 -0.74352992D+04 2.1147770 0.0002843 237.953 291.866 EM

172.181

20 -0.74337936D+04 1.5056686 0.0002025 235.166 299.535 EM

167.299

21 -0.74327609D+04 1.0326744 0.0001389 233.160 305.707 EM

163.133

22 -0.74320588D+04 0.7020544 0.0000945 231.833 310.603 EM

159.565

TECHNICAL 8 OUTPUT FOR STARTING VALUE SET 258

ITER LOGLIKELIHOOD ABS CHANGE REL CHANGE CLASS COUNTS ALGORITHM

1 -0.27712645D+05 0.0000000 0.0000000 588.749 66.533 EM

46.718

2 -0.76684939D+04 ************ 0.7232854 541.712 92.692 EM

67.596

3 -0.75595203D+04 108.9735113 0.0142105 486.970 126.645 EM

88.384

4 -0.75019052D+04 57.6151355 0.0076215 442.898 154.657 EM

104.445

5 -0.74685248D+04 33.3804509 0.0044496 408.729 177.492 EM

115.779

6 -0.74482105D+04 20.3142799 0.0027200 383.721 194.928 EM

123.351

7 -0.74379997D+04 10.2107425 0.0013709 366.455 207.465 EM

128.081

8 -0.74334094D+04 4.5903782 0.0006172 354.877 216.299 EM

130.824

9 -0.74313655D+04 2.0438847 0.0002750 347.230 222.527 EM

132.243

10 -0.74304296D+04 0.9358338 0.0001259 342.243 226.960 EM

132.797

TECHNICAL 8 OUTPUT FOR STARTING VALUE SET 259

ITER LOGLIKELIHOOD ABS CHANGE REL CHANGE CLASS COUNTS ALGORITHM

1 -0.17539315D+05 0.0000000 0.0000000 46.278 8.262 EM

647.460

2 -0.78212061D+04 9718.1091430 0.5540757 53.965 5.104 EM

642.931

3 -0.77507458D+04 70.4602425 0.0090089 72.739 4.544 EM

624.717

4 -0.77201578D+04 30.5879903 0.0039465 91.883 5.168 EM

604.949

5 -0.76980150D+04 22.1428144 0.0028682 113.451 6.374 EM

582.175

6 -0.76754660D+04 22.5490442 0.0029292 137.111 8.075 EM

556.814

7 -0.76513225D+04 24.1435012 0.0031455 162.319 10.251 EM

529.430

8 -0.76238169D+04 27.5055477 0.0035949 189.286 12.705 EM

500.009

9 -0.75905953D+04 33.2216687 0.0043576 217.095 15.298 EM

469.608

10 -0.75566879D+04 33.9073376 0.0044670 241.962 17.997 EM

442.041

11 -0.75328654D+04 23.8224871 0.0031525 261.691 20.690 EM

419.619

12 -0.75196689D+04 13.1965064 0.0017519 276.516 23.168 EM

402.315

13 -0.75128187D+04 6.8502491 0.0009110 287.168 25.595 EM

389.237

14 -0.75091353D+04 3.6833971 0.0004903 294.638 28.092 EM

379.271

15 -0.75069318D+04 2.2034516 0.0002934 299.845 30.717 EM

371.438

16 -0.75054094D+04 1.5223881 0.0002028 303.477 33.505 EM

365.018

17 -0.75042136D+04 1.1958818 0.0001593 306.001 36.474 EM

359.525

18 -0.75031859D+04 1.0276882 0.0001369 307.734 39.631 EM

354.635

19 -0.75022500D+04 0.9358926 0.0001247 308.885 42.983 EM

350.132

TECHNICAL 8 OUTPUT FOR STARTING VALUE SET 260

ITER LOGLIKELIHOOD ABS CHANGE REL CHANGE CLASS COUNTS ALGORITHM

1 -0.18456975D+05 0.0000000 0.0000000 245.665 444.571 EM

11.763

2 -0.75643761D+04 ************ 0.5901616 263.382 413.451 EM

25.167

3 -0.74800809D+04 84.2951486 0.0111437 275.068 388.444 EM

38.488

4 -0.74565302D+04 23.5507370 0.0031485 280.320 371.481 EM

50.199

5 -0.74458213D+04 10.7088866 0.0014362 281.376 360.697 EM

59.927

6 -0.74398788D+04 5.9425060 0.0007981 279.982 354.410 EM

67.608

7 -0.74365852D+04 3.2935635 0.0004427 277.403 350.961 EM

73.636

8 -0.74345301D+04 2.0550990 0.0002763 274.413 349.083 EM

78.503

9 -0.74331071D+04 1.4230051 0.0001914 271.412 348.053 EM

82.535

10 -0.74320741D+04 1.0330447 0.0001390 268.592 347.471 EM

85.937

11 -0.74313108D+04 0.7632447 0.0001027 266.039 347.114 EM

88.847

TECHNICAL 8 OUTPUT FOR STARTING VALUE SET 261

ITER LOGLIKELIHOOD ABS CHANGE REL CHANGE CLASS COUNTS ALGORITHM

1 -0.25050479D+05 0.0000000 0.0000000 146.055 150.200 EM

405.745

2 -0.76587952D+04 ************ 0.6942655 147.587 162.124 EM

392.290

3 -0.75382099D+04 120.5853171 0.0157447 142.955 178.371 EM

380.674

4 -0.74950586D+04 43.1512540 0.0057243 141.004 185.610 EM

375.387

5 -0.74868231D+04 8.2355337 0.0010988 141.390 188.343 EM

372.267

6 -0.74847878D+04 2.0352451 0.0002718 142.246 189.895 EM

369.859

7 -0.74838352D+04 0.9526483 0.0001273 142.978 191.226 EM

367.796

TECHNICAL 8 OUTPUT FOR STARTING VALUE SET 262

ITER LOGLIKELIHOOD ABS CHANGE REL CHANGE CLASS COUNTS ALGORITHM

1 -0.19434299D+05 0.0000000 0.0000000 632.193 48.804 EM

21.003

2 -0.77186031D+04 ************ 0.6028360 604.435 62.625 EM

34.940

3 -0.76591850D+04 59.4180998 0.0076980 571.208 86.809 EM

43.983

4 -0.76216565D+04 37.5284775 0.0048998 538.973 113.032 EM

49.995

5 -0.75931171D+04 28.5393945 0.0037445 507.998 139.394 EM

54.608

6 -0.75683229D+04 24.7941480 0.0032653 477.987 165.579 EM

58.434

7 -0.75453865D+04 22.9364638 0.0030306 448.714 191.561 EM

61.725

8 -0.75235659D+04 21.8205527 0.0028919 420.471 216.881 EM

64.648

9 -0.75031582D+04 20.4077488 0.0027125 394.300 240.435 EM

67.265

10 -0.74852251D+04 17.9330921 0.0023901 371.363 261.086 EM

69.551

11 -0.74706364D+04 14.5886907 0.0019490 352.185 278.218 EM

71.596

12 -0.74595109D+04 11.1254783 0.0014892 336.533 291.844 EM

73.623

13 -0.74514344D+04 8.0764793 0.0010827 323.781 302.421 EM

75.799

14 -0.74457886D+04 5.6458679 0.0007577 313.276 310.607 EM

78.117

15 -0.74419066D+04 3.8819403 0.0005214 304.495 317.005 EM

80.500

16 -0.74391712D+04 2.7354644 0.0003676 297.047 322.067 EM

82.885

17 -0.74371329D+04 2.0382851 0.0002740 290.634 326.136 EM

85.229

18 -0.74355265D+04 1.6063390 0.0002160 285.031 329.474 EM

87.495

19 -0.74342095D+04 1.3170398 0.0001771 280.080 332.267 EM

89.653

20 -0.74331071D+04 1.1023818 0.0001483 275.678 334.637 EM

91.685

21 -0.74321814D+04 0.9257275 0.0001245 271.766 336.657 EM

93.578

TECHNICAL 8 OUTPUT FOR STARTING VALUE SET 263

ITER LOGLIKELIHOOD ABS CHANGE REL CHANGE CLASS COUNTS ALGORITHM

1 -0.19123866D+05 0.0000000 0.0000000 11.481 380.162 EM

310.356

2 -0.76074287D+04 ************ 0.6022024 6.470 367.212 EM

328.317

3 -0.75529248D+04 54.5038932 0.0071646 5.441 358.982 EM

337.577

4 -0.75379521D+04 14.9726563 0.0019824 5.621 353.679 EM

342.700

5 -0.75284680D+04 9.4841458 0.0012582 8.037 349.590 EM

344.374

6 -0.75122609D+04 16.2070206 0.0021528 16.877 345.715 EM

339.408

7 -0.74873352D+04 24.9256968 0.0033180 28.214 339.881 EM

333.905

8 -0.74707882D+04 16.5470321 0.0022100 37.782 332.078 EM

332.140

9 -0.74607596D+04 10.0286111 0.0013424 46.069 324.131 EM

331.800

10 -0.74537386D+04 7.0209858 0.0009411 53.462 316.701 EM

331.836

11 -0.74484214D+04 5.3171972 0.0007134 60.030 309.825 EM

332.145

12 -0.74443603D+04 4.0611274 0.0005452 65.769 303.425 EM

332.806

13 -0.74412562D+04 3.1041129 0.0004170 70.754 297.468 EM

333.778

14 -0.74388333D+04 2.4228280 0.0003256 75.109 291.940 EM

334.951

15 -0.74368890D+04 1.9443839 0.0002614 78.944 286.826 EM

336.230

16 -0.74352938D+04 1.5951152 0.0002145 82.346 282.112 EM

337.542

17 -0.74339694D+04 1.3244243 0.0001781 85.379 277.791 EM

338.830

18 -0.74328689D+04 1.1004917 0.0001480 88.090 273.866 EM

340.044

19 -0.74319642D+04 0.9047618 0.0001217 90.517 270.343 EM

341.140

TECHNICAL 8 OUTPUT FOR STARTING VALUE SET 264

ITER LOGLIKELIHOOD ABS CHANGE REL CHANGE CLASS COUNTS ALGORITHM

1 -0.14525270D+05 0.0000000 0.0000000 676.147 25.553 EM

0.300

2 -0.78484949D+04 6676.7752853 0.4596662 677.459 24.088 EM

0.453

3 -0.78400756D+04 8.4192590 0.0010727 674.970 26.464 EM

0.566

4 -0.78361319D+04 3.9436661 0.0005030 671.039 30.314 EM

0.647

5 -0.78245051D+04 11.6268021 0.0014837 662.749 38.549 EM

0.702

6 -0.78010328D+04 23.4723824 0.0029999 651.874 49.377 EM

0.750

7 -0.77865789D+04 14.4538755 0.0018528 642.598 58.592 EM

0.809

8 -0.77810178D+04 5.5610450 0.0007142 634.968 66.162 EM

0.870

9 -0.77779678D+04 3.0500510 0.0003920 628.206 72.871 EM

0.922

10 -0.77756209D+04 2.3468800 0.0003017 621.677 79.359 EM

0.964

11 -0.77732197D+04 2.4012000 0.0003088 614.778 86.222 EM

1.000

12 -0.77697067D+04 3.5129592 0.0004519 606.424 94.541 EM

1.035

13 -0.77615459D+04 8.1608379 0.0010503 593.696 107.224 EM

1.080

14 -0.77356963D+04 25.8495749 0.0033305 571.109 129.724 EM

1.168

15 -0.76758314D+04 59.8649707 0.0077388 539.477 161.124 EM

1.399

16 -0.76132610D+04 62.5703463 0.0081516 508.207 191.870 EM

1.923

17 -0.75792672D+04 33.9937645 0.0044651 481.474 217.908 EM

2.618

18 -0.75615804D+04 17.6868892 0.0023336 458.724 240.050 EM

3.226

19 -0.75498699D+04 11.7104631 0.0015487 438.923 259.282 EM

3.795

20 -0.75411778D+04 8.6921011 0.0011513 421.515 276.197 EM

4.288

21 -0.75345595D+04 6.6182715 0.0008776 406.255 291.068 EM

4.677

22 -0.75294668D+04 5.0927668 0.0006759 393.033 303.944 EM

5.024

23 -0.75255836D+04 3.8831686 0.0005157 381.802 314.838 EM

5.361

24 -0.75227245D+04 2.8591189 0.0003799 372.480 323.843 EM

5.676

25 -0.75207196D+04 2.0048907 0.0002665 364.905 331.143 EM

5.952

26 -0.75193780D+04 1.3415844 0.0001784 358.848 336.966 EM

6.185

27 -0.75185111D+04 0.8669267 0.0001153 354.061 341.556 EM

6.383

TECHNICAL 8 OUTPUT FOR STARTING VALUE SET 265

ITER LOGLIKELIHOOD ABS CHANGE REL CHANGE CLASS COUNTS ALGORITHM

1 -0.15672480D+05 0.0000000 0.0000000 228.952 77.165 EM

395.883

2 -0.75672228D+04 8105.2572617 0.5171649 244.243 68.280 EM

389.477

3 -0.75270602D+04 40.1626633 0.0053075 252.106 66.991 EM

382.903

4 -0.75168272D+04 10.2329927 0.0013595 255.126 69.016 EM

377.858

5 -0.75101484D+04 6.6788072 0.0008885 255.088 72.541 EM

374.371

6 -0.75025598D+04 7.5885230 0.0010104 253.330 76.664 EM

372.006

7 -0.74945929D+04 7.9669598 0.0010619 251.388 80.596 EM

370.017

8 -0.74884840D+04 6.1089246 0.0008151 250.493 83.707 EM

367.800

9 -0.74850568D+04 3.4271766 0.0004577 250.830 85.905 EM

365.265

10 -0.74833959D+04 1.6608703 0.0002219 251.917 87.450 EM

362.633

11 -0.74825518D+04 0.8441015 0.0001128 253.296 88.585 EM

360.118

TECHNICAL 8 OUTPUT FOR STARTING VALUE SET 266

ITER LOGLIKELIHOOD ABS CHANGE REL CHANGE CLASS COUNTS ALGORITHM

1 -0.18433399D+05 0.0000000 0.0000000 4.314 654.462 EM

43.225

2 -0.78306025D+04 ************ 0.5751949 4.485 650.760 EM

46.754

3 -0.77701303D+04 60.4722220 0.0077226 5.210 628.315 EM

68.475

4 -0.76969042D+04 73.2261234 0.0094241 6.747 599.390 EM

95.863

5 -0.76411094D+04 55.7947838 0.0072490 12.722 566.593 EM

122.686

6 -0.75879851D+04 53.1243547 0.0069524 23.982 530.885 EM

147.133

7 -0.75517589D+04 36.2261736 0.0047741 34.916 498.939 EM

168.145

8 -0.75310997D+04 20.6592037 0.0027357 44.243 471.553 EM

186.204

9 -0.75174296D+04 13.6701222 0.0018152 52.250 447.838 EM

201.911

10 -0.75074504D+04 9.9791846 0.0013275 59.352 427.175 EM

215.474

11 -0.75000830D+04 7.3673899 0.0009813 65.802 409.274 EM

226.924

12 -0.74947589D+04 5.3240952 0.0007099 71.742 393.910 EM

236.348

13 -0.74909615D+04 3.7974202 0.0005067 77.267 380.813 EM

243.920

14 -0.74882387D+04 2.7227420 0.0003635 82.453 369.699 EM

249.848

15 -0.74862493D+04 1.9894178 0.0002657 87.370 360.305 EM

254.326

16 -0.74847485D+04 1.5008419 0.0002005 92.078 352.401 EM

257.521

17 -0.74835566D+04 1.1918798 0.0001592 96.642 345.785 EM

259.572

18 -0.74825343D+04 1.0222868 0.0001366 101.132 340.276 EM

260.591

19 -0.74815648D+04 0.9694716 0.0001296 105.629 335.708 EM

260.663

TECHNICAL 8 OUTPUT FOR STARTING VALUE SET 267

ITER LOGLIKELIHOOD ABS CHANGE REL CHANGE CLASS COUNTS ALGORITHM

1 -0.20929455D+05 0.0000000 0.0000000 605.594 5.005 EM

91.401

2 -0.77714933D+04 ************ 0.6286815 593.972 4.212 EM

103.816

3 -0.76992041D+04 72.2891946 0.0093018 569.856 10.044 EM

122.100

4 -0.76387858D+04 60.4182911 0.0078473 537.704 23.478 EM

140.818

5 -0.75893505D+04 49.4353430 0.0064716 503.411 39.320 EM

159.269

6 -0.75458170D+04 43.5334996 0.0057361 468.182 55.621 EM

178.196

7 -0.75025610D+04 43.2559430 0.0057324 433.400 70.488 EM

198.111

8 -0.74641741D+04 38.3869004 0.0051165 403.913 82.376 EM

215.710

9 -0.74428367D+04 21.3374591 0.0028586 382.726 90.930 EM

228.345

10 -0.74343183D+04 8.5183703 0.0011445 368.583 96.790 EM

236.627

11 -0.74310667D+04 3.2515651 0.0004374 359.406 100.758 EM

241.837

12 -0.74298047D+04 1.2619885 0.0001698 353.515 103.460 EM

245.025

13 -0.74293056D+04 0.4991222 0.0000672 349.738 105.333 EM

246.928

TECHNICAL 8 OUTPUT FOR STARTING VALUE SET 268

ITER LOGLIKELIHOOD ABS CHANGE REL CHANGE CLASS COUNTS ALGORITHM

1 -0.25884270D+05 0.0000000 0.0000000 126.027 219.282 EM

356.692

2 -0.75575613D+04 ************ 0.7080249 115.724 225.112 EM

361.164

3 -0.75259193D+04 31.6419326 0.0041868 115.140 224.767 EM

362.092

4 -0.75117777D+04 14.1416841 0.0018791 117.615 222.286 EM

362.099

5 -0.74983595D+04 13.4181920 0.0017863 120.698 219.701 EM

361.601

6 -0.74882917D+04 10.0677237 0.0013427 122.758 218.538 EM

360.704

7 -0.74837678D+04 4.5239195 0.0006041 123.683 218.715 EM

359.602

8 -0.74824037D+04 1.3640949 0.0001823 123.975 219.539 EM

358.487

9 -0.74820307D+04 0.3730138 0.0000499 123.974 220.577 EM

357.449

TECHNICAL 8 OUTPUT FOR STARTING VALUE SET 269

ITER LOGLIKELIHOOD ABS CHANGE REL CHANGE CLASS COUNTS ALGORITHM

1 -0.12967566D+05 0.0000000 0.0000000 663.188 22.179 EM

16.632

2 -0.77772004D+04 5190.3651725 0.4002575 640.752 38.707 EM

22.541

3 -0.77090993D+04 68.1010695 0.0087565 610.902 57.953 EM

33.144

4 -0.76727820D+04 36.3172486 0.0047110 584.695 77.125 EM

40.180

5 -0.76517297D+04 21.0523433 0.0027438 559.969 97.254 EM

44.777

6 -0.76333046D+04 18.4250580 0.0024080 535.418 118.258 EM

48.323

7 -0.76150334D+04 18.2712857 0.0023936 510.730 139.754 EM

51.516

8 -0.75946405D+04 20.3928476 0.0026780 484.746 161.789 EM

55.465

9 -0.75651846D+04 29.4559512 0.0038785 454.721 184.584 EM

62.695

10 -0.75151553D+04 50.0292647 0.0066131 421.071 206.115 EM

74.813

11 -0.74642962D+04 50.8590867 0.0067675 392.874 222.279 EM

86.847

12 -0.74405349D+04 23.7612619 0.0031833 374.537 232.617 EM

94.846

13 -0.74329888D+04 7.5461738 0.0010142 362.974 239.106 EM

99.920

14 -0.74304777D+04 2.5110681 0.0003378 355.585 243.154 EM

103.261

15 -0.74295549D+04 0.9227571 0.0001242 350.857 245.627 EM

105.516

TECHNICAL 8 OUTPUT FOR STARTING VALUE SET 270

ITER LOGLIKELIHOOD ABS CHANGE REL CHANGE CLASS COUNTS ALGORITHM

1 -0.27499690D+05 0.0000000 0.0000000 90.299 519.902 EM

91.799

2 -0.76112043D+04 ************ 0.7232258 97.353 498.471 EM

106.176

3 -0.75486883D+04 62.5159691 0.0082137 106.566 472.030 EM

123.404

4 -0.75220980D+04 26.5903180 0.0035225 116.056 448.460 EM

137.484

5 -0.75047059D+04 17.3921468 0.0023121 126.500 426.381 EM

149.119

6 -0.74894820D+04 15.2239135 0.0020286 138.428 404.887 EM

158.685

7 -0.74743036D+04 15.1783154 0.0020266 152.049 383.929 EM

166.022

8 -0.74590878D+04 15.2158406 0.0020358 166.295 364.886 EM

170.820

9 -0.74470950D+04 11.9927711 0.0016078 179.015 349.883 EM

173.102

10 -0.74403089D+04 6.7860958 0.0009112 189.191 339.346 EM

173.463

11 -0.74369472D+04 3.3617711 0.0004518 197.041 332.343 EM

172.616

12 -0.74351742D+04 1.7729442 0.0002384 203.106 327.827 EM

171.066

13 -0.74341121D+04 1.0620831 0.0001428 207.864 325.011 EM

169.125

14 -0.74333936D+04 0.7185569 0.0000967 211.670 323.353 EM

166.977

TECHNICAL 8 OUTPUT FOR STARTING VALUE SET 271

ITER LOGLIKELIHOOD ABS CHANGE REL CHANGE CLASS COUNTS ALGORITHM

1 -0.17441488D+05 0.0000000 0.0000000 123.233 526.508 EM

52.259

2 -0.77355153D+04 9705.9726973 0.5564877 110.588 513.711 EM

77.700

3 -0.76425459D+04 92.9694006 0.0120185 116.922 481.804 EM

103.274

4 -0.75706222D+04 71.9236741 0.0094110 125.267 453.264 EM

123.469

5 -0.75276870D+04 42.9352120 0.0056713 131.629 432.253 EM

138.118

6 -0.75049097D+04 22.7772248 0.0030258 138.643 413.988 EM

149.368

7 -0.74879251D+04 16.9846868 0.0022631 148.002 395.695 EM

158.303

8 -0.74716588D+04 16.2663000 0.0021723 160.039 376.993 EM

164.968

9 -0.74558684D+04 15.7903362 0.0021134 173.069 359.818 EM

169.112

10 -0.74444635D+04 11.4049163 0.0015297 184.601 346.525 EM

170.874

11 -0.74386078D+04 5.8557336 0.0007866 193.741 337.330 EM

170.929

12 -0.74358503D+04 2.7574924 0.0003707 200.780 331.269 EM

169.951

13 -0.74344056D+04 1.4447105 0.0001943 206.232 327.390 EM

168.379

14 -0.74335279D+04 0.8776450 0.0001181 210.524 325.001 EM

166.475

TECHNICAL 8 OUTPUT FOR STARTING VALUE SET 272

ITER LOGLIKELIHOOD ABS CHANGE REL CHANGE CLASS COUNTS ALGORITHM

1 -0.22523819D+05 0.0000000 0.0000000 81.564 438.676 EM

181.760

2 -0.76717814D+04 ************ 0.6593925 76.510 404.123 EM

221.367

3 -0.75802136D+04 91.5678245 0.0119357 79.756 370.164 EM

252.080

4 -0.75383109D+04 41.9026787 0.0055279 84.169 342.718 EM

275.113

5 -0.75096740D+04 28.6369785 0.0037989 87.359 323.166 EM

291.475

6 -0.74932724D+04 16.4015739 0.0021841 88.574 310.722 EM

302.704

7 -0.74862295D+04 7.0429292 0.0009399 88.579 302.923 EM

310.498

8 -0.74834650D+04 2.7644104 0.0003693 88.138 297.751 EM

316.111

9 -0.74822850D+04 1.1800327 0.0001577 87.599 294.132 EM

320.269

10 -0.74817324D+04 0.5526293 0.0000739 87.077 291.515 EM

323.408

TECHNICAL 8 OUTPUT FOR STARTING VALUE SET 273

ITER LOGLIKELIHOOD ABS CHANGE REL CHANGE CLASS COUNTS ALGORITHM

1 -0.24000772D+05 0.0000000 0.0000000 10.442 494.868 EM

196.690

2 -0.76643683D+04 ************ 0.6806616 7.931 473.005 EM

221.064

3 -0.75721751D+04 92.1931902 0.0120288 7.011 449.805 EM

245.184

4 -0.75447049D+04 27.4702042 0.0036278 6.379 429.730 EM

265.892

5 -0.75326099D+04 12.0950012 0.0016031 5.954 412.080 EM

283.966

6 -0.75249516D+04 7.6583053 0.0010167 5.690 396.536 EM

299.774

7 -0.75194945D+04 5.4570711 0.0007252 5.540 383.177 EM

313.283

8 -0.75156379D+04 3.8565311 0.0005129 5.465 372.059 EM

324.477

9 -0.75130305D+04 2.6074573 0.0003469 5.436 363.071 EM

333.493

10 -0.75113477D+04 1.6827642 0.0002240 5.435 355.970 EM

340.596

11 -0.75103013D+04 1.0464744 0.0001393 5.450 350.446 EM

346.104

12 -0.75096671D+04 0.6341649 0.0000844 5.475 346.192 EM

350.333

TECHNICAL 8 OUTPUT FOR STARTING VALUE SET 274

ITER LOGLIKELIHOOD ABS CHANGE REL CHANGE CLASS COUNTS ALGORITHM

1 -0.20235542D+05 0.0000000 0.0000000 302.213 235.441 EM

164.346

2 -0.75551833D+04 ************ 0.6266380 293.930 226.556 EM

181.514

3 -0.74941347D+04 61.0485815 0.0080804 288.374 226.980 EM

186.646

4 -0.74689124D+04 25.2223179 0.0033656 283.208 234.432 EM

184.360

5 -0.74566171D+04 12.2952945 0.0016462 277.884 245.449 EM

178.666

6 -0.74490847D+04 7.5324650 0.0010102 272.368 257.647 EM

171.985

7 -0.74435154D+04 5.5692348 0.0007476 266.764 269.841 EM

165.395

8 -0.74391945D+04 4.3209055 0.0005805 261.304 281.320 EM

159.376

9 -0.74359542D+04 3.2403640 0.0004356 256.274 291.594 EM

154.133

10 -0.74336710D+04 2.2831748 0.0003070 251.900 300.384 EM

149.716

11 -0.74321549D+04 1.5160877 0.0002039 248.300 307.631 EM

146.069

12 -0.74311877D+04 0.9671531 0.0001301 245.477 313.444 EM

143.079

TECHNICAL 8 OUTPUT FOR STARTING VALUE SET 275

ITER LOGLIKELIHOOD ABS CHANGE REL CHANGE CLASS COUNTS ALGORITHM

1 -0.20692718D+05 0.0000000 0.0000000 0.625 591.769 EM

109.606

2 -0.78420262D+04 ************ 0.6210248 2.111 598.585 EM

101.304

3 -0.77295703D+04 112.4558882 0.0143402 2.679 571.212 EM

128.109

4 -0.76316925D+04 97.8778700 0.0126628 3.003 540.950 EM

158.047

5 -0.75944615D+04 37.2309939 0.0048785 3.222 515.039 EM

183.739

6 -0.75755213D+04 18.9402105 0.0024940 3.387 490.998 EM

207.615

7 -0.75605693D+04 14.9519488 0.0019737 3.527 468.522 EM

229.951

8 -0.75483925D+04 12.1768202 0.0016106 3.658 447.851 EM

250.491

9 -0.75386352D+04 9.7573174 0.0012926 3.786 429.051 EM

269.163

10 -0.75308171D+04 7.8181045 0.0010371 3.913 412.112 EM

285.975

11 -0.75245627D+04 6.2543547 0.0008305 4.036 397.099 EM

300.865

12 -0.75196823D+04 4.8804008 0.0006486 4.151 384.127 EM

313.722

13 -0.75160536D+04 3.6287143 0.0004826 4.257 373.249 EM

324.494

14 -0.75135046D+04 2.5489650 0.0003391 4.357 364.376 EM

333.267

15 -0.75118023D+04 1.7023564 0.0002266 4.455 357.298 EM

340.248

16 -0.75107055D+04 1.0967567 0.0001460 4.557 351.737 EM

345.706

17 -0.75100116D+04 0.6939614 0.0000924 4.669 347.411 EM

349.921

TECHNICAL 8 OUTPUT FOR STARTING VALUE SET 276

ITER LOGLIKELIHOOD ABS CHANGE REL CHANGE CLASS COUNTS ALGORITHM

1 -0.21754700D+05 0.0000000 0.0000000 370.996 247.806 EM

83.198

2 -0.75316615D+04 ************ 0.6537915 360.913 254.681 EM

86.406

3 -0.74620025D+04 69.6590207 0.0092488 349.124 261.263 EM

91.612

4 -0.74406149D+04 21.3876167 0.0028662 341.894 264.394 EM

95.712

5 -0.74344496D+04 6.1652667 0.0008286 338.521 264.888 EM

98.592

6 -0.74321778D+04 2.2717850 0.0003056 337.409 263.920 EM

100.671

7 -0.74310656D+04 1.1121925 0.0001496 337.462 262.282 EM

102.256

8 -0.74303982D+04 0.6674159 0.0000898 338.051 260.426 EM

103.523

TECHNICAL 8 OUTPUT FOR STARTING VALUE SET 277

ITER LOGLIKELIHOOD ABS CHANGE REL CHANGE CLASS COUNTS ALGORITHM

1 -0.20318017D+05 0.0000000 0.0000000 140.489 288.969 EM

272.542

2 -0.76644222D+04 ************ 0.6227770 147.176 287.548 EM

267.276

3 -0.75429479D+04 121.4742602 0.0158491 143.407 287.916 EM

270.677

4 -0.74987832D+04 44.1647620 0.0058551 137.359 292.193 EM

272.448

5 -0.74888098D+04 9.9733748 0.0013300 132.355 297.612 EM

272.034

6 -0.74858777D+04 2.9320766 0.0003915 128.392 302.520 EM

271.088

7 -0.74845617D+04 1.3160349 0.0001758 125.089 306.516 EM

270.394

8 -0.74838621D+04 0.6995499 0.0000935 122.218 309.671 EM

270.111

TECHNICAL 8 OUTPUT FOR STARTING VALUE SET 278

ITER LOGLIKELIHOOD ABS CHANGE REL CHANGE CLASS COUNTS ALGORITHM

1 -0.21549462D+05 0.0000000 0.0000000 16.898 385.925 EM

299.177

2 -0.76122018D+04 ************ 0.6467568 16.106 372.231 EM

313.664

3 -0.75257841D+04 86.4177162 0.0113525 14.985 361.001 EM

326.014

4 -0.75150911D+04 10.6930290 0.0014209 14.123 352.894 EM

334.983

5 -0.75121040D+04 2.9870398 0.0003975 13.744 346.576 EM

341.680

6 -0.75104432D+04 1.6608728 0.0002211 13.783 341.545 EM

346.672

7 -0.75095240D+04 0.9191120 0.0001224 14.156 337.516 EM

350.328

TECHNICAL 8 OUTPUT FOR STARTING VALUE SET 279

ITER LOGLIKELIHOOD ABS CHANGE REL CHANGE CLASS COUNTS ALGORITHM

1 -0.26420813D+05 0.0000000 0.0000000 10.921 594.662 EM

96.417

2 -0.76774951D+04 ************ 0.7094149 8.102 569.619 EM

124.279

3 -0.76225007D+04 54.9943235 0.0071631 13.722 536.971 EM

151.307

4 -0.75682831D+04 54.2176011 0.0071128 25.409 501.236 EM

175.355

5 -0.75353678D+04 32.9153784 0.0043491 34.782 472.159 EM

195.058

6 -0.75190986D+04 16.2692045 0.0021590 42.514 447.930 EM

211.557

7 -0.75083652D+04 10.7334063 0.0014275 49.252 427.200 EM

225.548

8 -0.75008653D+04 7.4998862 0.0009989 55.231 409.445 EM

237.324

9 -0.74956250D+04 5.2402904 0.0006986 60.568 394.285 EM

247.146

10 -0.74919679D+04 3.6570661 0.0004879 65.346 381.361 EM

255.293

11 -0.74894029D+04 2.5649641 0.0003424 69.633 370.339 EM

262.028

12 -0.74875916D+04 1.8113054 0.0002418 73.489 360.935 EM

267.576

13 -0.74863049D+04 1.2867632 0.0001719 76.965 352.909 EM

272.126

14 -0.74853856D+04 0.9192814 0.0001228 80.104 346.059 EM

275.837

TECHNICAL 8 OUTPUT FOR STARTING VALUE SET 280

ITER LOGLIKELIHOOD ABS CHANGE REL CHANGE CLASS COUNTS ALGORITHM

1 -0.22094406D+05 0.0000000 0.0000000 408.313 228.293 EM

65.395

2 -0.76270672D+04 ************ 0.6547965 394.642 233.867 EM

73.491

3 -0.75301389D+04 96.9283359 0.0127085 377.239 242.592 EM

82.169

4 -0.74755156D+04 54.6232765 0.0072540 362.017 250.798 EM

89.185

5 -0.74485542D+04 26.9614666 0.0036066 351.223 256.211 EM

94.566

6 -0.74377074D+04 10.8467155 0.0014562 344.796 258.819 EM

98.385

7 -0.74331851D+04 4.5223110 0.0006080 341.531 259.444 EM

101.025

8 -0.74312132D+04 1.9719677 0.0002653 340.162 258.930 EM

102.908

9 -0.74302847D+04 0.9284819 0.0001249 339.822 257.862 EM

104.316

TECHNICAL 8 OUTPUT FOR STARTING VALUE SET 281

ITER LOGLIKELIHOOD ABS CHANGE REL CHANGE CLASS COUNTS ALGORITHM

1 -0.16588766D+05 0.0000000 0.0000000 590.799 9.582 EM

101.619

2 -0.76563570D+04 8932.4092710 0.5384613 564.562 7.921 EM

129.518

3 -0.76204887D+04 35.8682517 0.0046848 537.479 8.557 EM

155.964

4 -0.75970102D+04 23.4785249 0.0030810 510.668 10.007 EM

181.325

5 -0.75757003D+04 21.3099535 0.0028050 484.140 12.078 EM

205.783

6 -0.75556498D+04 20.0504116 0.0026467 458.179 14.615 EM

229.205

7 -0.75371551D+04 18.4947544 0.0024478 433.109 17.745 EM

251.146

8 -0.75198736D+04 17.2815187 0.0022928 409.359 21.839 EM

270.801

9 -0.75034016D+04 16.4719794 0.0021905 387.577 27.055 EM

287.368

10 -0.74882928D+04 15.1088330 0.0020136 368.461 33.077 EM

300.462

11 -0.74756252D+04 12.6675805 0.0016917 352.418 39.530 EM

310.053

12 -0.74656205D+04 10.0046507 0.0013383 339.261 46.114 EM

316.625

13 -0.74578371D+04 7.7834323 0.0010426 328.400 52.547 EM

321.053

14 -0.74517815D+04 6.0555792 0.0008120 319.211 58.586 EM

324.203

15 -0.74471023D+04 4.6792553 0.0006279 311.223 64.072 EM

326.705

16 -0.74434989D+04 3.6033671 0.0004839 304.138 68.969 EM

328.893

17 -0.74406892D+04 2.8097155 0.0003775 297.777 73.324 EM

330.900

18 -0.74384485D+04 2.2406796 0.0003011 292.017 77.208 EM

332.775

19 -0.74366208D+04 1.8277354 0.0002457 286.775 80.687 EM

334.538

20 -0.74351037D+04 1.5170755 0.0002040 281.990 83.813 EM

336.196

21 -0.74338326D+04 1.2711115 0.0001710 277.632 86.627 EM

337.741

22 -0.74327683D+04 1.0642360 0.0001432 273.686 89.163 EM

339.151

23 -0.74318877D+04 0.8806427 0.0001185 270.151 91.447 EM

340.401

TECHNICAL 8 OUTPUT FOR STARTING VALUE SET 282

ITER LOGLIKELIHOOD ABS CHANGE REL CHANGE CLASS COUNTS ALGORITHM

1 -0.19574596D+05 0.0000000 0.0000000 22.195 665.376 EM

14.429

2 -0.78301942D+04 ************ 0.5999818 24.675 659.589 EM

17.736

3 -0.77586624D+04 71.5318330 0.0091354 48.368 629.145 EM

24.487

4 -0.76920788D+04 66.5835673 0.0085818 72.601 592.575 EM

36.824

5 -0.76379336D+04 54.1452777 0.0070391 96.210 552.786 EM

53.004

6 -0.75871517D+04 50.7818780 0.0066486 117.951 515.388 EM

68.662

7 -0.75496541D+04 37.4975280 0.0049422 136.600 482.945 EM

82.455

8 -0.75242708D+04 25.3833109 0.0033622 151.718 456.167 EM

94.115

9 -0.75081413D+04 16.1294909 0.0021437 163.434 434.878 EM

103.688

10 -0.74984477D+04 9.6936905 0.0012911 172.187 418.373 EM

111.440

11 -0.74927827D+04 5.6649404 0.0007555 178.523 405.746 EM

117.731

12 -0.74894623D+04 3.3204235 0.0004431 182.979 396.105 EM

122.916

13 -0.74874654D+04 1.9969033 0.0002666 186.004 388.706 EM

127.290

14 -0.74862212D+04 1.2441441 0.0001662 187.951 382.977 EM

131.072

15 -0.74854187D+04 0.8025664 0.0001072 189.087 378.501 EM

134.412

TECHNICAL 8 OUTPUT FOR STARTING VALUE SET 283

ITER LOGLIKELIHOOD ABS CHANGE REL CHANGE CLASS COUNTS ALGORITHM

1 -0.15345368D+05 0.0000000 0.0000000 40.504 197.417 EM

464.079

2 -0.76470695D+04 7698.2989998 0.5016692 41.119 228.303 EM

432.578

3 -0.75589387D+04 88.1307685 0.0115248 45.293 255.136 EM

401.571

4 -0.75249012D+04 34.0375034 0.0045029 50.526 275.792 EM

375.682

5 -0.75019413D+04 22.9598998 0.0030512 55.123 291.110 EM

355.767

6 -0.74900125D+04 11.9288055 0.0015901 58.190 301.802 EM

342.009

7 -0.74854890D+04 4.5234430 0.0006039 60.225 309.385 EM

332.390

8 -0.74836221D+04 1.8669859 0.0002494 61.824 314.994 EM

325.182

9 -0.74826948D+04 0.9272565 0.0001239 63.223 319.249 EM

319.528

TECHNICAL 8 OUTPUT FOR STARTING VALUE SET 284

ITER LOGLIKELIHOOD ABS CHANGE REL CHANGE CLASS COUNTS ALGORITHM

1 -0.21417405D+05 0.0000000 0.0000000 88.843 496.501 EM

116.655

2 -0.77081011D+04 ************ 0.6401011 113.105 475.029 EM

113.866

3 -0.76416823D+04 66.4187578 0.0086167 137.137 446.173 EM

118.689

4 -0.76111947D+04 30.4876403 0.0039897 159.260 420.821 EM

121.920

5 -0.75936715D+04 17.5231856 0.0023023 180.247 399.415 EM

122.338

6 -0.75787896D+04 14.8818883 0.0019598 201.345 380.168 EM

120.488

7 -0.75621076D+04 16.6819939 0.0022011 222.785 362.593 EM

116.622

8 -0.75447324D+04 17.3752238 0.0022977 243.135 347.903 EM

110.962

9 -0.75306953D+04 14.0370630 0.0018605 261.058 336.651 EM

104.291

10 -0.75208943D+04 9.8010247 0.0013015 276.150 328.175 EM

97.674

11 -0.75141645D+04 6.7297902 0.0008948 288.519 321.589 EM

91.892

12 -0.75095342D+04 4.6302714 0.0006162 298.481 316.220 EM

87.299

13 -0.75064158D+04 3.1184221 0.0004153 306.423 311.658 EM

83.919

14 -0.75043787D+04 2.0371236 0.0002714 312.718 307.675 EM

81.607

15 -0.75030730D+04 1.3056536 0.0001740 317.691 304.135 EM

80.173

16 -0.75022346D+04 0.8384428 0.0001117 321.612 300.936 EM

79.451

TECHNICAL 8 OUTPUT FOR STARTING VALUE SET 285

ITER LOGLIKELIHOOD ABS CHANGE REL CHANGE CLASS COUNTS ALGORITHM

1 -0.28201502D+05 0.0000000 0.0000000 42.257 508.692 EM

151.051

2 -0.76556097D+04 ************ 0.7285389 59.807 482.638 EM

159.555

3 -0.75422901D+04 113.3196128 0.0148022 72.259 455.909 EM

173.833

4 -0.75130821D+04 29.2079181 0.0038726 81.472 434.389 EM

186.139

5 -0.75010610D+04 12.0211693 0.0016000 90.241 414.612 EM

197.147

6 -0.74926737D+04 8.3872491 0.0011181 99.052 396.627 EM

206.321

7 -0.74860715D+04 6.6022691 0.0008812 107.932 380.816 EM

213.252

8 -0.74805089D+04 5.5625437 0.0007431 116.983 367.151 EM

217.866

9 -0.74752681D+04 5.2408148 0.0007006 126.452 355.281 EM

220.266

10 -0.74696235D+04 5.6445919 0.0007551 136.601 344.792 EM

220.606

11 -0.74631480D+04 6.4755281 0.0008669 147.359 335.575 EM

219.067

12 -0.74563153D+04 6.8326557 0.0009155 158.061 327.993 EM

215.946

13 -0.74503622D+04 5.9530724 0.0007984 167.882 322.374 EM

211.745

14 -0.74459378D+04 4.4244544 0.0005939 176.412 318.555 EM

207.033

15 -0.74428053D+04 3.1325378 0.0004207 183.643 316.127 EM

202.230

16 -0.74405367D+04 2.2685471 0.0003048 189.735 314.710 EM

197.555

17 -0.74388251D+04 1.7115661 0.0002300 194.885 314.015 EM

193.100

18 -0.74374850D+04 1.3400922 0.0001801 199.271 313.837 EM

188.892

19 -0.74364046D+04 1.0804127 0.0001453 203.043 314.026 EM

184.931

20 -0.74355137D+04 0.8908950 0.0001198 206.320 314.474 EM

181.206

TECHNICAL 8 OUTPUT FOR STARTING VALUE SET 286

ITER LOGLIKELIHOOD ABS CHANGE REL CHANGE CLASS COUNTS ALGORITHM

1 -0.19373261D+05 0.0000000 0.0000000 182.304 445.072 EM

74.624

2 -0.77127663D+04 ************ 0.6018860 170.920 430.595 EM

100.485

3 -0.76179380D+04 94.8282725 0.0122950 169.539 407.430 EM

125.031

4 -0.75744156D+04 43.5223565 0.0057131 167.416 389.439 EM

145.146

5 -0.75480622D+04 26.3533832 0.0034793 162.678 377.630 EM

161.692

6 -0.75271109D+04 20.9513294 0.0027757 157.447 369.092 EM

175.461

7 -0.75076360D+04 19.4749211 0.0025873 154.176 361.525 EM

186.299

8 -0.74913130D+04 16.3229880 0.0021742 153.957 354.149 EM

193.894

9 -0.74781469D+04 13.1660898 0.0017575 157.104 346.547 EM

198.349

10 -0.74662586D+04 11.8883565 0.0015897 163.548 338.492 EM

199.960

11 -0.74550511D+04 11.2074690 0.0015011 171.920 330.939 EM

199.140

12 -0.74465115D+04 8.5396031 0.0011455 180.243 325.169 EM

196.588

13 -0.74415816D+04 4.9299013 0.0006620 187.502 321.329 EM

193.169

14 -0.74388958D+04 2.6857418 0.0003609 193.577 318.938 EM

189.485

15 -0.74372428D+04 1.6530090 0.0002222 198.637 317.554 EM

185.809

16 -0.74360779D+04 1.1648830 0.0001566 202.881 316.873 EM

182.246

17 -0.74351818D+04 0.8961498 0.0001205 206.481 316.687 EM

178.832

TECHNICAL 8 OUTPUT FOR STARTING VALUE SET 287

ITER LOGLIKELIHOOD ABS CHANGE REL CHANGE CLASS COUNTS ALGORITHM

1 -0.18305335D+05 0.0000000 0.0000000 503.539 148.784 EM

49.676

2 -0.77211687D+04 ************ 0.5782012 487.862 140.085 EM

74.053

3 -0.76002350D+04 120.9336662 0.0156626 457.814 148.304 EM

95.881

4 -0.75467305D+04 53.5044740 0.0070398 428.619 162.446 EM

110.935

5 -0.75177596D+04 28.9709867 0.0038389 403.022 178.672 EM

120.306

6 -0.74998823D+04 17.8772511 0.0023780 381.437 195.146 EM

125.417

7 -0.74875232D+04 12.3590672 0.0016479 363.564 210.875 EM

127.561

8 -0.74778301D+04 9.6931528 0.0012946 348.722 225.597 EM

127.681

9 -0.74693024D+04 8.5276582 0.0011404 336.046 239.449 EM

126.505

10 -0.74613185D+04 7.9839738 0.0010689 324.762 252.675 EM

124.563

11 -0.74539010D+04 7.4174935 0.0009941 314.365 265.443 EM

122.192

12 -0.74473830D+04 6.5179109 0.0008744 304.669 277.678 EM

119.653

13 -0.74421018D+04 5.2812751 0.0007091 295.747 289.021 EM

117.232

14 -0.74381743D+04 3.9274667 0.0005277 287.767 299.048 EM

115.185

15 -0.74354414D+04 2.7329340 0.0003674 280.826 307.536 EM

113.639

16 -0.74335877D+04 1.8536655 0.0002493 274.904 314.506 EM

112.591

17 -0.74323086D+04 1.2791127 0.0001721 269.904 320.136 EM

111.960

18 -0.74313904D+04 0.9181733 0.0001235 265.705 324.654 EM

111.641

TECHNICAL 8 OUTPUT FOR STARTING VALUE SET 288

ITER LOGLIKELIHOOD ABS CHANGE REL CHANGE CLASS COUNTS ALGORITHM

1 -0.24332972D+05 0.0000000 0.0000000 108.810 330.420 EM

262.769

2 -0.77316799D+04 ************ 0.6822550 115.403 316.927 EM

269.669

3 -0.76408935D+04 90.7863877 0.0117421 119.234 316.611 EM

266.155

4 -0.75869831D+04 53.9104126 0.0070555 120.471 325.530 EM

255.999

5 -0.75330451D+04 53.9379532 0.0071093 121.130 332.025 EM

248.845

6 -0.74975190D+04 35.5261038 0.0047160 122.549 332.505 EM

246.946

7 -0.74844690D+04 13.0500616 0.0017406 125.258 330.167 EM

246.575

8 -0.74793789D+04 5.0900507 0.0006801 129.224 327.274 EM

245.502

9 -0.74756014D+04 3.7775148 0.0005051 134.420 324.482 EM

243.098

10 -0.74713900D+04 4.2113656 0.0005633 140.906 321.772 EM

239.322

11 -0.74661651D+04 5.2248965 0.0006993 148.625 319.046 EM

234.329

12 -0.74600507D+04 6.1144631 0.0008190 157.132 316.468 EM

228.400

13 -0.74539627D+04 6.0879588 0.0008161 165.663 314.382 EM

221.955

14 -0.74488863D+04 5.0764171 0.0006810 173.589 312.947 EM

215.464

15 -0.74450789D+04 3.8073921 0.0005111 180.627 312.098 EM

209.275

16 -0.74422914D+04 2.7874897 0.0003744 186.747 311.713 EM

203.540

17 -0.74402093D+04 2.0821163 0.0002798 192.031 311.689 EM

198.280

18 -0.74386031D+04 1.6062243 0.0002159 196.596 311.949 EM

193.455

19 -0.74373259D+04 1.2772063 0.0001717 200.559 312.429 EM

189.012

20 -0.74362849D+04 1.0409872 0.0001400 204.024 313.073 EM

184.903

21 -0.74354199D+04 0.8650182 0.0001163 207.077 313.837 EM

181.085

TECHNICAL 8 OUTPUT FOR STARTING VALUE SET 289

ITER LOGLIKELIHOOD ABS CHANGE REL CHANGE CLASS COUNTS ALGORITHM

1 -0.19062028D+05 0.0000000 0.0000000 323.411 137.548 EM

241.041

2 -0.75789779D+04 ************ 0.6024044 291.376 153.510 EM

257.114

3 -0.75042893D+04 74.6885877 0.0098547 278.433 158.279 EM

265.288

4 -0.74874608D+04 16.8284274 0.0022425 276.507 158.028 EM

267.465

5 -0.74821966D+04 5.2642285 0.0007031 278.905 156.974 EM

266.121

6 -0.74786208D+04 3.5758069 0.0004779 282.987 156.739 EM

262.274

7 -0.74746240D+04 3.9968264 0.0005344 287.596 157.993 EM

256.411

8 -0.74695100D+04 5.1139802 0.0006842 292.053 161.003 EM

248.944

9 -0.74632217D+04 6.2883278 0.0008419 295.993 165.602 EM

240.405

10 -0.74565613D+04 6.6603571 0.0008924 299.340 171.178 EM

231.482

11 -0.74507396D+04 5.8217384 0.0007808 302.125 177.017 EM

222.858

12 -0.74463341D+04 4.4054652 0.0005913 304.390 182.631 EM

214.979

13 -0.74431713D+04 3.1627859 0.0004247 306.221 187.799 EM

207.980

14 -0.74408676D+04 2.3036775 0.0003095 307.744 192.454 EM

201.802

15 -0.74391235D+04 1.7441846 0.0002344 309.073 196.610 EM

196.316

16 -0.74377521D+04 1.3713163 0.0001843 310.293 200.311 EM

191.396

17 -0.74366417D+04 1.1104672 0.0001493 311.452 203.613 EM

186.936

18 -0.74357226D+04 0.9190625 0.0001236 312.576 206.570 EM

182.855

TECHNICAL 8 OUTPUT FOR STARTING VALUE SET 290

ITER LOGLIKELIHOOD ABS CHANGE REL CHANGE CLASS COUNTS ALGORITHM

1 -0.25223324D+05 0.0000000 0.0000000 328.104 286.749 EM

87.146

2 -0.75971963D+04 ************ 0.6988027 317.758 292.383 EM

91.859

3 -0.74944456D+04 102.7506576 0.0135248 306.366 300.277 EM

95.357

4 -0.74510232D+04 43.4224021 0.0057939 296.224 309.496 EM

96.280

5 -0.74383351D+04 12.6881187 0.0017029 288.355 316.863 EM

96.782

6 -0.74349192D+04 3.4158909 0.0004592 282.053 322.499 EM

97.448

7 -0.74333505D+04 1.5687195 0.0002110 276.801 326.897 EM

98.302

8 -0.74322869D+04 1.0635580 0.0001431 272.326 330.403 EM

99.271

9 -0.74314667D+04 0.8202484 0.0001104 268.475 333.238 EM

100.286

TECHNICAL 8 OUTPUT FOR STARTING VALUE SET 291

ITER LOGLIKELIHOOD ABS CHANGE REL CHANGE CLASS COUNTS ALGORITHM

1 -0.16665277D+05 0.0000000 0.0000000 419.929 1.915 EM

280.157

2 -0.76220043D+04 9043.2729923 0.5426416 416.311 1.698 EM

283.991

3 -0.75521080D+04 69.8963587 0.0091703 405.750 2.260 EM

293.991

4 -0.75361985D+04 15.9094660 0.0021066 393.404 3.273 EM

305.323

5 -0.75295260D+04 6.6725216 0.0008854 381.825 4.148 EM

316.027

6 -0.75243450D+04 5.1809873 0.0006881 371.118 5.566 EM

325.317

7 -0.75183965D+04 5.9485053 0.0007906 360.334 8.717 EM

332.949

8 -0.75123027D+04 6.0938171 0.0008105 350.028 13.024 EM

338.948

9 -0.75076466D+04 4.6560385 0.0006198 340.920 17.469 EM

343.610

10 -0.75043425D+04 3.3040810 0.0004401 332.919 21.807 EM

347.274

11 -0.75018205D+04 2.5220666 0.0003361 325.700 26.099 EM

350.201

12 -0.74997689D+04 2.0515495 0.0002735 319.050 30.373 EM

352.577

13 -0.74980480D+04 1.7209199 0.0002295 312.859 34.602 EM

354.539

14 -0.74965884D+04 1.4596550 0.0001947 307.071 38.742 EM

356.187

15 -0.74953470D+04 1.2413889 0.0001656 301.651 42.758 EM

357.591

16 -0.74942897D+04 1.0572936 0.0001411 296.571 46.629 EM

358.800

17 -0.74933860D+04 0.9036627 0.0001206 291.804 50.348 EM

359.848

TECHNICAL 8 OUTPUT FOR STARTING VALUE SET 292

ITER LOGLIKELIHOOD ABS CHANGE REL CHANGE CLASS COUNTS ALGORITHM

1 -0.15689937D+05 0.0000000 0.0000000 641.674 17.123 EM

43.203

2 -0.76808731D+04 8009.0644067 0.5104587 603.179 32.777 EM

66.045

3 -0.76084997D+04 72.3734050 0.0094225 564.531 47.442 EM

90.027

4 -0.75723770D+04 36.1226287 0.0047477 530.445 59.939 EM

111.617

5 -0.75482375D+04 24.1395238 0.0031878 500.092 71.387 EM

130.521

6 -0.75302799D+04 17.9576611 0.0023791 472.816 82.269 EM

146.915

7 -0.75158110D+04 14.4688092 0.0019214 447.881 92.988 EM

161.132

8 -0.75033740D+04 12.4370658 0.0016548 424.900 103.791 EM

173.309

9 -0.74923670D+04 11.0069862 0.0014669 403.871 114.811 EM

183.318

10 -0.74823839D+04 9.9830926 0.0013324 384.826 126.254 EM

190.920

11 -0.74727546D+04 9.6293087 0.0012869 367.659 138.402 EM

195.939

12 -0.74629145D+04 9.8400862 0.0013168 352.551 151.100 EM

198.349

13 -0.74536614D+04 9.2531190 0.0012399 340.309 163.308 EM

198.383

14 -0.74467456D+04 6.9157730 0.0009278 331.406 173.952 EM

196.642

15 -0.74423994D+04 4.3462197 0.0005836 325.397 182.737 EM

193.866

16 -0.74397037D+04 2.6957289 0.0003622 321.521 189.874 EM

190.605

17 -0.74379064D+04 1.7972974 0.0002416 319.134 195.694 EM

187.173

18 -0.74366089D+04 1.2974396 0.0001744 317.779 200.491 EM

183.730

19 -0.74356127D+04 0.9962020 0.0001340 317.141 204.501 EM

180.359

TECHNICAL 8 OUTPUT FOR STARTING VALUE SET 293

ITER LOGLIKELIHOOD ABS CHANGE REL CHANGE CLASS COUNTS ALGORITHM

1 -0.18055469D+05 0.0000000 0.0000000 291.165 340.685 EM

70.149

2 -0.76555058D+04 ************ 0.5760007 280.073 334.954 EM

86.974

3 -0.76060954D+04 49.4104051 0.0064542 273.556 323.548 EM

104.896

4 -0.75808774D+04 25.2179994 0.0033155 272.575 308.561 EM

120.863

5 -0.75565943D+04 24.2830413 0.0032032 275.657 292.138 EM

134.205

6 -0.75258620D+04 30.7323242 0.0040670 281.421 277.289 EM

143.290

7 -0.74858374D+04 40.0246001 0.0053183 289.325 266.203 EM

146.472

8 -0.74507750D+04 35.0623844 0.0046838 298.557 258.575 EM

144.868

9 -0.74358631D+04 14.9118874 0.0020014 306.857 253.353 EM

141.789

10 -0.74318760D+04 3.9871389 0.0005362 313.446 249.591 EM

138.963

11 -0.74306570D+04 1.2189834 0.0001640 318.484 246.861 EM

136.655

12 -0.74301258D+04 0.5312015 0.0000715 322.322 244.902 EM

134.776

TECHNICAL 8 OUTPUT FOR STARTING VALUE SET 294

ITER LOGLIKELIHOOD ABS CHANGE REL CHANGE CLASS COUNTS ALGORITHM

1 -0.18789333D+05 0.0000000 0.0000000 6.397 645.683 EM

49.920

2 -0.78307612D+04 ************ 0.5832337 11.332 638.566 EM

52.102

3 -0.77633051D+04 67.4561011 0.0086142 32.985 606.858 EM

62.156

4 -0.76731925D+04 90.1125334 0.0116075 56.274 566.103 EM

79.622

5 -0.76076896D+04 65.5028765 0.0085366 76.676 526.186 EM

99.138

6 -0.75614747D+04 46.2149321 0.0060748 93.261 491.053 EM

117.686

7 -0.75312477D+04 30.2269987 0.0039975 106.468 461.757 EM

133.775

8 -0.75119883D+04 19.2594119 0.0025573 116.850 438.207 EM

146.943

9 -0.75001673D+04 11.8209999 0.0015736 124.842 419.808 EM

157.351

10 -0.74931551D+04 7.0122255 0.0009349 130.861 405.698 EM

165.442

11 -0.74890380D+04 4.1171074 0.0005494 135.319 394.939 EM

171.742

12 -0.74865801D+04 2.4578170 0.0003282 138.578 386.703 EM

176.720

13 -0.74850688D+04 1.5113307 0.0002019 140.926 380.340 EM

180.734

14 -0.74841136D+04 0.9552028 0.0001276 142.583 375.380 EM

184.037

TECHNICAL 8 OUTPUT FOR STARTING VALUE SET 295

ITER LOGLIKELIHOOD ABS CHANGE REL CHANGE CLASS COUNTS ALGORITHM

1 -0.21910258D+05 0.0000000 0.0000000 359.841 150.334 EM

191.825

2 -0.76263852D+04 ************ 0.6519263 338.872 138.388 EM

224.740

3 -0.75515139D+04 74.8712579 0.0098174 321.785 135.217 EM

244.999

4 -0.75224662D+04 29.0476855 0.0038466 310.190 136.152 EM

255.658

5 -0.75052448D+04 17.2214167 0.0022893 302.453 140.298 EM

259.249

6 -0.74927343D+04 12.5105335 0.0016669 296.994 147.688 EM

257.318

7 -0.74833095D+04 9.4247722 0.0012579 292.669 157.883 EM

251.448

8 -0.74759380D+04 7.3715288 0.0009851 288.687 170.177 EM

243.136

9 -0.74697056D+04 6.2323390 0.0008337 284.545 183.890 EM

233.564

10 -0.74640593D+04 5.6463167 0.0007559 279.996 198.436 EM

223.568

11 -0.74587541D+04 5.3052425 0.0007108 274.974 213.364 EM

213.662

12 -0.74537145D+04 5.0395891 0.0006757 269.538 228.321 EM

204.140

13 -0.74489613D+04 4.7531732 0.0006377 263.842 242.979 EM

195.179

14 -0.74445933D+04 4.3679844 0.0005864 258.118 256.977 EM

186.906

15 -0.74407656D+04 3.8277187 0.0005142 252.648 269.922 EM

179.430

16 -0.74376276D+04 3.1379755 0.0004217 247.711 281.458 EM

172.832

17 -0.74352424D+04 2.3851919 0.0003207 243.511 291.353 EM

167.136

18 -0.74335521D+04 1.6903527 0.0002273 240.145 299.556 EM

162.299

19 -0.74324137D+04 1.1384129 0.0001531 237.605 306.174 EM

158.221

20 -0.74316644D+04 0.7492707 0.0001008 235.806 311.417 EM

154.777

TECHNICAL 8 OUTPUT FOR STARTING VALUE SET 296

ITER LOGLIKELIHOOD ABS CHANGE REL CHANGE CLASS COUNTS ALGORITHM

1 -0.14188768D+05 0.0000000 0.0000000 163.033 503.300 EM

35.667

2 -0.77827475D+04 6406.0204585 0.4514853 138.318 500.128 EM

63.554

3 -0.76461714D+04 136.5761588 0.0175486 140.474 474.799 EM

86.727

4 -0.75650703D+04 81.1010838 0.0106068 165.560 440.370 EM

96.070

5 -0.75198659D+04 45.2044439 0.0059754 198.003 406.865 EM

97.132

6 -0.74913380D+04 28.5278860 0.0037937 228.904 377.311 EM

95.785

7 -0.74706095D+04 20.7285115 0.0027670 254.603 353.187 EM

94.210

8 -0.74568726D+04 13.7368136 0.0018388 274.524 334.434 EM

93.042

9 -0.74483763D+04 8.4962985 0.0011394 289.585 319.940 EM

92.475

10 -0.74430712D+04 5.3051524 0.0007123 300.949 308.545 EM

92.506

11 -0.74396143D+04 3.4569279 0.0004644 309.593 299.384 EM

93.023

12 -0.74372302D+04 2.3840248 0.0003205 316.265 291.851 EM

93.884

13 -0.74354796D+04 1.7506688 0.0002354 321.514 285.522 EM

94.964

14 -0.74341189D+04 1.3606444 0.0001830 325.733 280.106 EM

96.161

15 -0.74330169D+04 1.1019862 0.0001482 329.192 275.406 EM

97.403

16 -0.74321049D+04 0.9120639 0.0001227 332.065 271.297 EM

98.638

TECHNICAL 8 OUTPUT FOR STARTING VALUE SET 297

ITER LOGLIKELIHOOD ABS CHANGE REL CHANGE CLASS COUNTS ALGORITHM

1 -0.15190005D+05 0.0000000 0.0000000 1.487 694.011 EM

6.502

2 -0.78330772D+04 7356.9274072 0.4843269 9.107 684.909 EM

7.984

3 -0.77724856D+04 60.5916711 0.0077354 22.631 658.406 EM

20.963

4 -0.76932023D+04 79.2832352 0.0102005 38.062 620.655 EM

43.283

5 -0.76311531D+04 62.0491927 0.0080655 53.497 581.336 EM

67.166

6 -0.75880176D+04 43.1355254 0.0056526 67.881 544.756 EM

89.363

7 -0.75584186D+04 29.5989739 0.0039008 81.240 511.783 EM

108.976

8 -0.75364205D+04 21.9981127 0.0029104 94.045 481.969 EM

125.986

9 -0.75185800D+04 17.8404615 0.0023672 106.702 454.748 EM

140.550

10 -0.75030752D+04 15.5048627 0.0020622 119.523 429.639 EM

152.838

11 -0.74887128D+04 14.3624261 0.0019142 132.941 406.208 EM

162.851

12 -0.74743971D+04 14.3156714 0.0019116 147.331 384.267 EM

170.402

13 -0.74600775D+04 14.3195606 0.0019158 161.984 364.768 EM

175.248

14 -0.74484555D+04 11.6220648 0.0015579 175.082 349.454 EM

177.464

15 -0.74415274D+04 6.9280298 0.0009301 185.650 338.678 EM

177.672

16 -0.74379357D+04 3.5917697 0.0004827 193.863 331.515 EM

176.622

17 -0.74359816D+04 1.9540912 0.0002627 200.245 326.900 EM

174.855

18 -0.74347863D+04 1.1952765 0.0001607 205.276 324.024 EM

172.700

19 -0.74339673D+04 0.8189488 0.0001102 209.316 322.332 EM

170.352

TECHNICAL 8 OUTPUT FOR STARTING VALUE SET 298

ITER LOGLIKELIHOOD ABS CHANGE REL CHANGE CLASS COUNTS ALGORITHM

1 -0.26694144D+05 0.0000000 0.0000000 109.378 579.779 EM

12.843

2 -0.76528818D+04 ************ 0.7133123 139.186 537.682 EM

25.132

3 -0.75720064D+04 80.8754265 0.0105680 168.621 490.647 EM

42.733

4 -0.75133090D+04 58.6973396 0.0077519 195.076 447.695 EM

59.229

5 -0.74696961D+04 43.6129181 0.0058048 216.055 412.971 EM

72.974

6 -0.74457171D+04 23.9789775 0.0032102 230.339 388.578 EM

83.083

7 -0.74356961D+04 10.0210810 0.0013459 239.349 372.554 EM

90.097

8 -0.74317478D+04 3.9483027 0.0005310 244.786 362.292 EM

94.923

9 -0.74301894D+04 1.5583112 0.0002097 247.942 355.768 EM

98.290

10 -0.74295584D+04 0.6310871 0.0000849 249.692 351.607 EM

100.701

TECHNICAL 8 OUTPUT FOR STARTING VALUE SET 299

ITER LOGLIKELIHOOD ABS CHANGE REL CHANGE CLASS COUNTS ALGORITHM

1 -0.17382909D+05 0.0000000 0.0000000 689.497 11.028 EM

1.475

2 -0.78208748D+04 9562.0337713 0.5500825 681.961 18.973 EM

1.066

3 -0.77959824D+04 24.8924343 0.0031828 673.870 27.160 EM

0.970

4 -0.77872442D+04 8.7381565 0.0011209 666.136 34.909 EM

0.955

5 -0.77788326D+04 8.4116182 0.0010802 655.752 45.278 EM

0.971

6 -0.77592749D+04 19.5576866 0.0025142 637.636 63.342 EM

1.022

7 -0.77138277D+04 45.4471767 0.0058571 609.758 91.076 EM

1.167

8 -0.76637103D+04 50.1174342 0.0064971 579.141 121.364 EM

1.495

9 -0.76300321D+04 33.6781797 0.0043945 549.790 150.184 EM

2.026

10 -0.76055091D+04 24.5229899 0.0032140 522.155 177.213 EM

2.632

11 -0.75855522D+04 19.9569278 0.0026240 496.565 202.251 EM

3.184

12 -0.75693209D+04 16.2313415 0.0021398 473.372 224.919 EM

3.708

13 -0.75566098D+04 12.7110602 0.0016793 452.707 245.105 EM

4.187

14 -0.75469145D+04 9.6953413 0.0012830 434.386 263.054 EM

4.560

15 -0.75394334D+04 7.4811090 0.0009913 418.103 279.036 EM

4.860

16 -0.75335072D+04 5.9262016 0.0007860 403.682 293.178 EM

5.140

17 -0.75287926D+04 4.7145376 0.0006258 391.091 305.491 EM

5.419

18 -0.75251358D+04 3.6567919 0.0004857 380.339 315.972 EM

5.689

19 -0.75224284D+04 2.7073980 0.0003598 371.378 324.687 EM

5.935

20 -0.75205273D+04 1.9011695 0.0002527 364.069 331.781 EM

6.150

21 -0.75192535D+04 1.2737953 0.0001694 358.207 337.455 EM

6.338

22 -0.75184301D+04 0.8233459 0.0001095 353.564 341.932 EM

6.504

TECHNICAL 8 OUTPUT FOR STARTING VALUE SET 300

ITER LOGLIKELIHOOD ABS CHANGE REL CHANGE CLASS COUNTS ALGORITHM

1 -0.18683952D+05 0.0000000 0.0000000 441.175 203.537 EM

57.288

2 -0.76019187D+04 ************ 0.5931311 416.940 214.285 EM

70.776

3 -0.75164156D+04 85.5031445 0.0112476 387.764 238.359 EM

75.878

4 -0.74836930D+04 32.7225488 0.0043535 363.204 261.087 EM

77.708

5 -0.74667764D+04 16.9166683 0.0022605 343.861 279.200 EM

78.938

6 -0.74557803D+04 10.9960396 0.0014727 328.795 292.926 EM

80.279

7 -0.74482489D+04 7.5313802 0.0010101 316.804 303.366 EM

81.831

8 -0.74431501D+04 5.0988482 0.0006846 306.993 311.469 EM

83.538

9 -0.74397429D+04 3.4072400 0.0004578 298.805 317.846 EM

85.349

10 -0.74373880D+04 2.3549065 0.0003165 291.863 322.913 EM

87.225

11 -0.74356477D+04 1.7402655 0.0002340 285.889 326.989 EM

89.122

12 -0.74342777D+04 1.3699605 0.0001842 280.681 330.323 EM

90.996

13 -0.74331546D+04 1.1231114 0.0001511 276.100 333.092 EM

92.809

14 -0.74322189D+04 0.9356812 0.0001259 272.058 335.410 EM

94.532

TECHNICAL 8 OUTPUT FOR STARTING VALUE SET 301

ITER LOGLIKELIHOOD ABS CHANGE REL CHANGE CLASS COUNTS ALGORITHM

1 -0.21484652D+05 0.0000000 0.0000000 3.775 540.091 EM

158.134

2 -0.76132805D+04 ************ 0.6456410 4.405 512.699 EM

184.896

3 -0.75748322D+04 38.4482532 0.0050502 4.540 487.476 EM

209.983

4 -0.75583371D+04 16.4951769 0.0021776 4.558 464.819 EM

232.623

5 -0.75465345D+04 11.8025799 0.0015615 4.560 444.440 EM

253.000

6 -0.75372692D+04 9.2652492 0.0012277 4.574 426.084 EM

271.342

7 -0.75298270D+04 7.4422539 0.0009874 4.603 409.605 EM

287.792

8 -0.75238325D+04 5.9944608 0.0007961 4.649 395.014 EM

302.338

9 -0.75191283D+04 4.7042030 0.0006252 4.708 382.405 EM

314.887

10 -0.75156185D+04 3.5097906 0.0004668 4.780 371.821 EM

325.399

11 -0.75131492D+04 2.4693199 0.0003286 4.863 363.179 EM

333.959

12 -0.75115001D+04 1.6491194 0.0002195 4.956 356.274 EM

340.770

13 -0.75104403D+04 1.0598251 0.0001411 5.056 350.845 EM

346.099

14 -0.75097762D+04 0.6641021 0.0000884 5.157 346.619 EM

350.224

TECHNICAL 8 OUTPUT FOR STARTING VALUE SET 302

ITER LOGLIKELIHOOD ABS CHANGE REL CHANGE CLASS COUNTS ALGORITHM

1 -0.22943977D+05 0.0000000 0.0000000 331.348 150.825 EM

219.826

2 -0.76008961D+04 ************ 0.6687193 310.638 149.798 EM

241.564

3 -0.75345665D+04 66.3296617 0.0087266 292.014 151.590 EM

258.396

4 -0.75148498D+04 19.7166894 0.0026168 279.238 151.510 EM

271.252

5 -0.75067678D+04 8.0819757 0.0010755 270.597 149.867 EM

281.536

6 -0.75030614D+04 3.7064014 0.0004937 264.298 147.555 EM

290.148

7 -0.75009261D+04 2.1353237 0.0002846 259.283 145.027 EM

297.690

8 -0.74992858D+04 1.6403075 0.0002187 254.959 142.473 EM

304.568

9 -0.74977325D+04 1.5532494 0.0002071 250.958 140.000 EM

311.042

10 -0.74961261D+04 1.6064523 0.0002143 247.041 137.704 EM

317.255

11 -0.74944548D+04 1.6712380 0.0002229 243.072 135.676 EM

323.252

12 -0.74927744D+04 1.6803946 0.0002242 239.008 133.987 EM

329.004

13 -0.74911652D+04 1.6092239 0.0002148 234.882 132.686 EM

334.432

14 -0.74897015D+04 1.4637655 0.0001954 230.772 131.790 EM

339.438

15 -0.74884346D+04 1.2668168 0.0001691 226.772 131.297 EM

343.931

16 -0.74873881D+04 1.0465774 0.0001398 222.964 131.189 EM

347.847

17 -0.74865584D+04 0.8296215 0.0001108 219.401 131.438 EM

351.162

TECHNICAL 8 OUTPUT FOR STARTING VALUE SET 303

ITER LOGLIKELIHOOD ABS CHANGE REL CHANGE CLASS COUNTS ALGORITHM

1 -0.21724281D+05 0.0000000 0.0000000 46.291 450.332 EM

205.377

2 -0.77031449D+04 ************ 0.6454131 40.224 448.571 EM

213.205

3 -0.76346502D+04 68.4947071 0.0088918 41.306 435.974 EM

224.720

4 -0.75865347D+04 48.1155067 0.0063023 43.032 417.694 EM

241.274

5 -0.75439447D+04 42.5899565 0.0056139 44.780 398.156 EM

259.064

6 -0.75187633D+04 25.1813711 0.0033380 46.913 381.381 EM

273.706

7 -0.75082061D+04 10.5572599 0.0014041 49.514 368.108 EM

284.378

8 -0.75036482D+04 4.5578178 0.0006070 52.516 357.764 EM

291.720

9 -0.75012837D+04 2.3645069 0.0003151 55.887 349.444 EM

296.669

10 -0.74997348D+04 1.5489624 0.0002065 59.617 342.408 EM

299.975

11 -0.74984991D+04 1.2356535 0.0001648 63.716 336.146 EM

302.138

12 -0.74973750D+04 1.1240971 0.0001499 68.201 330.318 EM

303.481

13 -0.74962660D+04 1.1089947 0.0001479 73.099 324.687 EM

304.214

14 -0.74951144D+04 1.1516729 0.0001536 78.443 319.083 EM

304.474

15 -0.74938753D+04 1.2391063 0.0001653 84.282 313.368 EM

304.350

16 -0.74925047D+04 1.3705694 0.0001829 90.676 307.427 EM

303.897

17 -0.74909519D+04 1.5527462 0.0002072 97.703 301.153 EM

303.143

18 -0.74891548D+04 1.7971578 0.0002399 105.460 294.445 EM

302.095

19 -0.74870387D+04 2.1160461 0.0002825 114.059 287.205 EM

300.736

20 -0.74845250D+04 2.5137527 0.0003357 123.620 279.357 EM

299.023

21 -0.74815527D+04 2.9723126 0.0003971 134.243 270.869 EM

296.888

22 -0.74781107D+04 3.4419432 0.0004601 145.960 261.784 EM

294.256

23 -0.74742547D+04 3.8560556 0.0005156 158.695 252.239 EM

291.066

24 -0.74700890D+04 4.1656635 0.0005573 172.260 242.438 EM

287.302

25 -0.74657298D+04 4.3591964 0.0005836 186.416 232.597 EM

282.987

26 -0.74612754D+04 4.4544246 0.0005966 200.927 222.901 EM

278.173

27 -0.74567977D+04 4.4776428 0.0006001 215.585 213.485 EM

272.931

28 -0.74523588D+04 4.4388962 0.0005953 230.184 204.449 EM

267.367

29 -0.74480466D+04 4.3122306 0.0005786 244.473 195.889 EM

261.639

30 -0.74440063D+04 4.0403176 0.0005425 258.115 187.916 EM

255.969

31 -0.74404280D+04 3.5783355 0.0004807 270.723 180.656 EM

250.621

32 -0.74374787D+04 2.9492334 0.0003964 281.947 174.205 EM

245.847

33 -0.74352267D+04 2.2519906 0.0003028 291.569 168.603 EM

241.828

34 -0.74336192D+04 1.6075234 0.0002162 299.547 163.814 EM

238.639

35 -0.74325242D+04 1.0949878 0.0001473 305.992 159.749 EM

236.259

36 -0.74317924D+04 0.7317832 0.0000985 311.110 156.291 EM

234.599

TECHNICAL 8 OUTPUT FOR STARTING VALUE SET 304

ITER LOGLIKELIHOOD ABS CHANGE REL CHANGE CLASS COUNTS ALGORITHM

1 -0.28924655D+05 0.0000000 0.0000000 392.371 29.978 EM

279.651

2 -0.76051537D+04 ************ 0.7370702 380.075 38.111 EM

283.814

3 -0.75197802D+04 85.3734156 0.0112257 362.117 45.776 EM

294.107

4 -0.75057808D+04 13.9994867 0.0018617 346.180 51.333 EM

304.487

5 -0.75008140D+04 4.9667320 0.0006617 332.816 55.436 EM

313.748

6 -0.74979916D+04 2.8224390 0.0003763 321.583 58.725 EM

321.692

7 -0.74960713D+04 1.9202509 0.0002561 311.996 61.543 EM

328.461

8 -0.74946117D+04 1.4596372 0.0001947 303.669 64.075 EM

334.256

9 -0.74934262D+04 1.1855196 0.0001582 296.319 66.437 EM

339.244

10 -0.74924323D+04 0.9938962 0.0001326 289.747 68.704 EM

343.549

TECHNICAL 8 OUTPUT FOR STARTING VALUE SET 305

ITER LOGLIKELIHOOD ABS CHANGE REL CHANGE CLASS COUNTS ALGORITHM

1 -0.18493774D+05 0.0000000 0.0000000 41.724 196.768 EM

463.507

2 -0.77256731D+04 ************ 0.5822555 49.304 203.734 EM

448.961

3 -0.76458271D+04 79.8460639 0.0103352 60.644 212.839 EM

428.517

4 -0.76016092D+04 44.2178612 0.0057833 68.103 223.078 EM

410.820

5 -0.75738761D+04 27.7331005 0.0036483 71.310 236.527 EM

394.164

6 -0.75484142D+04 25.4619126 0.0033618 71.410 252.853 EM

377.737

7 -0.75267015D+04 21.7127319 0.0028765 69.368 268.660 EM

363.972

8 -0.75146229D+04 12.0785204 0.0016048 66.348 282.178 EM

353.473

9 -0.75087776D+04 5.8453615 0.0007779 63.212 293.131 EM

345.657

10 -0.75056456D+04 3.1319512 0.0004171 60.315 301.735 EM

339.950

11 -0.75038081D+04 1.8374904 0.0002448 57.734 308.383 EM

335.883

12 -0.75026738D+04 1.1343321 0.0001512 55.443 313.474 EM

333.083

13 -0.75019451D+04 0.7287145 0.0000971 53.388 317.349 EM

331.263

TECHNICAL 8 OUTPUT FOR STARTING VALUE SET 306

ITER LOGLIKELIHOOD ABS CHANGE REL CHANGE CLASS COUNTS ALGORITHM

1 -0.20551229D+05 0.0000000 0.0000000 29.877 488.988 EM

183.135

2 -0.76149187D+04 ************ 0.6294665 28.417 476.608 EM

196.975

3 -0.75692823D+04 45.6363931 0.0059930 34.966 456.256 EM

210.778

4 -0.75482910D+04 20.9912784 0.0027732 45.416 434.604 EM

221.980

5 -0.75322934D+04 15.9976310 0.0021194 59.037 413.215 EM

229.748

6 -0.75180226D+04 14.2707634 0.0018946 74.877 393.264 EM

233.859

7 -0.75058105D+04 12.2121169 0.0016244 91.673 375.715 EM

234.612

8 -0.74960307D+04 9.7798329 0.0013030 108.682 360.744 EM

232.574

9 -0.74881254D+04 7.9052879 0.0010546 125.763 347.882 EM

228.355

10 -0.74813050D+04 6.8203889 0.0009108 142.949 336.508 EM

222.544

11 -0.74750677D+04 6.2372508 0.0008337 160.202 326.128 EM

215.670

12 -0.74691979D+04 5.8698138 0.0007853 177.394 316.428 EM

208.178

13 -0.74636397D+04 5.5581881 0.0007441 194.347 307.235 EM

200.417

14 -0.74583918D+04 5.2479675 0.0007031 210.898 298.461 EM

192.640

15 -0.74534527D+04 4.9390625 0.0006622 226.912 290.062 EM

185.026

16 -0.74488255D+04 4.6271519 0.0006208 242.251 282.041 EM

177.709

17 -0.74445581D+04 4.2674575 0.0005729 256.719 274.460 EM

170.821

18 -0.74407650D+04 3.7930994 0.0005095 270.052 267.446 EM

164.502

19 -0.74375855D+04 3.1795286 0.0004273 281.967 261.156 EM

158.876

20 -0.74351004D+04 2.4850780 0.0003341 292.264 255.721 EM

154.015

21 -0.74332838D+04 1.8165619 0.0002443 300.882 251.206 EM

149.913

22 -0.74320241D+04 1.2596756 0.0001695 307.904 247.597 EM

146.499

23 -0.74311796D+04 0.8445408 0.0001136 313.513 244.821 EM

143.666

TECHNICAL 8 OUTPUT FOR STARTING VALUE SET 307

ITER LOGLIKELIHOOD ABS CHANGE REL CHANGE CLASS COUNTS ALGORITHM

1 -0.24401234D+05 0.0000000 0.0000000 51.091 394.442 EM

256.467

2 -0.75928671D+04 ************ 0.6888327 48.225 385.016 EM

268.758

3 -0.75297855D+04 63.0815718 0.0083080 50.384 368.600 EM

283.016

4 -0.75027060D+04 27.0794797 0.0035963 53.469 353.282 EM

295.250

5 -0.74910232D+04 11.6828246 0.0015571 56.015 341.305 EM

304.681

6 -0.74861935D+04 4.8296510 0.0006447 58.025 332.148 EM

311.827

7 -0.74839792D+04 2.2143340 0.0002958 59.736 325.005 EM

317.259

8 -0.74828559D+04 1.1233505 0.0001501 61.265 319.341 EM

321.394

9 -0.74822464D+04 0.6094808 0.0000815 62.656 314.791 EM

324.553

TECHNICAL 8 OUTPUT FOR STARTING VALUE SET 308

ITER LOGLIKELIHOOD ABS CHANGE REL CHANGE CLASS COUNTS ALGORITHM

1 -0.27424493D+05 0.0000000 0.0000000 316.139 301.896 EM

83.965

2 -0.76489299D+04 ************ 0.7210913 300.883 305.251 EM

95.866

3 -0.75911699D+04 57.7600164 0.0075514 285.192 309.553 EM

107.255

4 -0.75535429D+04 37.6270076 0.0049567 270.470 314.946 EM

116.584

5 -0.75108271D+04 42.7158214 0.0056551 260.322 319.065 EM

122.614

6 -0.74650721D+04 45.7549151 0.0060919 255.451 321.693 EM

124.855

7 -0.74390749D+04 25.9972481 0.0034825 253.064 324.105 EM

124.830

8 -0.74320338D+04 7.0410896 0.0009465 251.361 326.407 EM

124.232

9 -0.74303363D+04 1.6975503 0.0002284 249.899 328.490 EM

123.612

10 -0.74297518D+04 0.5844467 0.0000787 248.648 330.303 EM

123.049

TECHNICAL 8 OUTPUT FOR STARTING VALUE SET 309

ITER LOGLIKELIHOOD ABS CHANGE REL CHANGE CLASS COUNTS ALGORITHM

1 -0.16350848D+05 0.0000000 0.0000000 435.374 104.573 EM

162.053

2 -0.76667534D+04 8684.0947453 0.5311097 407.710 133.545 EM

160.744

3 -0.75737636D+04 92.9898655 0.0121290 377.872 158.476 EM

165.652

4 -0.75361160D+04 37.6475934 0.0049708 352.859 177.875 EM

171.266

5 -0.75117310D+04 24.3849431 0.0032357 331.446 191.003 EM

179.551

6 -0.74870148D+04 24.7162196 0.0032903 314.736 197.357 EM

189.906

7 -0.74634933D+04 23.5215307 0.0031416 305.612 197.216 EM

199.172

8 -0.74478351D+04 15.6581374 0.0020980 303.847 192.808 EM

205.345

9 -0.74401959D+04 7.6392227 0.0010257 305.707 187.094 EM

209.199

10 -0.74366108D+04 3.5851682 0.0004819 308.462 181.663 EM

211.875

11 -0.74348051D+04 1.8056803 0.0002428 311.100 176.910 EM

213.990

12 -0.74337950D+04 1.0100232 0.0001359 313.380 172.798 EM

215.822

13 -0.74331450D+04 0.6500554 0.0000874 315.305 169.199 EM

217.496

TECHNICAL 8 OUTPUT FOR STARTING VALUE SET 310

ITER LOGLIKELIHOOD ABS CHANGE REL CHANGE CLASS COUNTS ALGORITHM

1 -0.29591823D+05 0.0000000 0.0000000 80.100 564.452 EM

57.448

2 -0.75972039D+04 ************ 0.7432668 108.848 517.345 EM

75.808

3 -0.75340104D+04 63.1934792 0.0083180 134.617 472.206 EM

95.177

4 -0.74946862D+04 39.3242233 0.0052196 157.546 434.327 EM

110.126

5 -0.74665310D+04 28.1551680 0.0037567 177.976 403.347 EM

120.677

6 -0.74473880D+04 19.1430777 0.0025639 194.353 379.961 EM

127.686

7 -0.74376736D+04 9.7143508 0.0013044 206.380 363.634 EM

131.986

8 -0.74333894D+04 4.2841767 0.0005760 214.960 352.645 EM

134.395

9 -0.74314852D+04 1.9042010 0.0002562 221.072 345.378 EM

135.550

10 -0.74305965D+04 0.8887622 0.0001196 225.472 340.639 EM

135.889

TECHNICAL 8 OUTPUT FOR STARTING VALUE SET 311

ITER LOGLIKELIHOOD ABS CHANGE REL CHANGE CLASS COUNTS ALGORITHM

1 -0.22481586D+05 0.0000000 0.0000000 645.498 1.708 EM

54.795

2 -0.78601514D+04 ************ 0.6503738 652.616 8.814 EM

40.569

3 -0.77938672D+04 66.2842522 0.0084329 634.960 23.670 EM

43.370

4 -0.77191498D+04 74.7173749 0.0095867 596.429 39.371 EM

66.200

5 -0.76176519D+04 101.4978641 0.0131488 553.995 54.079 EM

93.926

6 -0.75671604D+04 50.4915454 0.0066282 520.118 66.733 EM

115.149

7 -0.75428779D+04 24.2824537 0.0032089 491.006 78.128 EM

132.866

8 -0.75256543D+04 17.2235809 0.0022834 464.687 89.097 EM

148.216

9 -0.75114824D+04 14.1719465 0.0018832 440.345 100.096 EM

161.559

10 -0.74990431D+04 12.4392538 0.0016560 417.783 111.315 EM

172.902

11 -0.74877931D+04 11.2500445 0.0015002 397.040 122.927 EM

182.033

12 -0.74771439D+04 10.6491661 0.0014222 378.084 135.223 EM

188.694

13 -0.74664104D+04 10.7335827 0.0014355 361.040 148.249 EM

192.711

14 -0.74559867D+04 10.4236823 0.0013961 346.743 161.116 EM

194.141

15 -0.74478234D+04 8.1633161 0.0010949 336.036 172.518 EM

193.446

16 -0.74427087D+04 5.1146712 0.0006867 328.689 181.926 EM

191.385

17 -0.74396798D+04 3.0289035 0.0004070 323.887 189.507 EM

188.606

18 -0.74377625D+04 1.9172840 0.0002577 320.870 195.622 EM

185.508

19 -0.74364318D+04 1.3306735 0.0001789 319.082 200.612 EM

182.306

20 -0.74354360D+04 0.9957996 0.0001339 318.144 204.742 EM

179.114

TECHNICAL 8 OUTPUT FOR STARTING VALUE SET 312

ITER LOGLIKELIHOOD ABS CHANGE REL CHANGE CLASS COUNTS ALGORITHM

1 -0.22899942D+05 0.0000000 0.0000000 320.922 170.051 EM

211.028

2 -0.76336665D+04 ************ 0.6666513 325.708 158.641 EM

217.651

3 -0.75699108D+04 63.7556855 0.0083519 325.037 143.693 EM

233.270

4 -0.75331168D+04 36.7940217 0.0048606 320.619 135.843 EM

245.538

5 -0.75140931D+04 19.0236491 0.0025253 312.744 138.253 EM

251.003

6 -0.75020351D+04 12.0580146 0.0016047 302.792 149.244 EM

249.963

7 -0.74901090D+04 11.9261143 0.0015897 291.754 167.040 EM

243.206

8 -0.74766105D+04 13.4984895 0.0018022 280.400 189.281 EM

232.319

9 -0.74631940D+04 13.4164456 0.0017945 269.624 212.533 EM

219.844

10 -0.74526359D+04 10.5581532 0.0014147 260.126 233.963 EM

207.911

11 -0.74455523D+04 7.0836312 0.0009505 252.153 252.426 EM

197.421

12 -0.74408735D+04 4.6787524 0.0006284 245.663 267.853 EM

188.484

13 -0.74376529D+04 3.2205652 0.0004328 240.528 280.518 EM

180.955

14 -0.74354109D+04 2.2420027 0.0003014 236.605 290.752 EM

174.643

15 -0.74338788D+04 1.5321282 0.0002061 233.746 298.895 EM

169.359

16 -0.74328503D+04 1.0285472 0.0001384 231.786 305.294 EM

164.920

17 -0.74321575D+04 0.6927902 0.0000932 230.556 310.286 EM

161.159

TECHNICAL 8 OUTPUT FOR STARTING VALUE SET 313

ITER LOGLIKELIHOOD ABS CHANGE REL CHANGE CLASS COUNTS ALGORITHM

1 -0.16999342D+05 0.0000000 0.0000000 117.391 501.678 EM

82.931

2 -0.76647629D+04 9334.5786406 0.5491141 123.098 479.987 EM

98.915

3 -0.75539977D+04 110.7652450 0.0144512 129.613 453.438 EM

118.948

4 -0.75136786D+04 40.3190400 0.0053374 137.557 432.321 EM

132.122

5 -0.74992451D+04 14.4335428 0.0019210 144.783 415.799 EM

141.418

6 -0.74923500D+04 6.8950720 0.0009194 150.539 403.143 EM

148.318

7 -0.74886397D+04 3.7102870 0.0004952 154.850 393.510 EM

153.640

8 -0.74865107D+04 2.1290377 0.0002843 157.962 386.138 EM

157.900

9 -0.74852202D+04 1.2905286 0.0001724 160.139 380.437 EM

161.425

10 -0.74844036D+04 0.8165211 0.0001091 161.600 375.981 EM

164.419

TECHNICAL 8 OUTPUT FOR STARTING VALUE SET 314

ITER LOGLIKELIHOOD ABS CHANGE REL CHANGE CLASS COUNTS ALGORITHM

1 -0.15697506D+05 0.0000000 0.0000000 69.204 569.398 EM

63.399

2 -0.77663060D+04 7931.1999820 0.5052522 58.848 558.472 EM

84.681

3 -0.76442530D+04 122.0529958 0.0157157 69.898 528.920 EM

103.183

4 -0.75925116D+04 51.7414574 0.0067687 89.292 498.686 EM

114.021

5 -0.75647026D+04 27.8089334 0.0036627 111.429 469.477 EM

121.094

6 -0.75419575D+04 22.7451064 0.0030067 135.317 441.071 EM

125.611

7 -0.75207526D+04 21.2049462 0.0028116 160.143 413.842 EM

128.015

8 -0.75015460D+04 19.2065571 0.0025538 184.312 389.057 EM

128.631

9 -0.74856433D+04 15.9026876 0.0021199 206.536 367.701 EM

127.763

10 -0.74730942D+04 12.5491008 0.0016764 226.497 349.720 EM

125.782

11 -0.74629906D+04 10.1036088 0.0013520 244.427 334.453 EM

123.120

12 -0.74546164D+04 8.3742442 0.0011221 260.573 321.240 EM

120.188

13 -0.74477538D+04 6.8625830 0.0009206 274.987 309.675 EM

117.338

14 -0.74424183D+04 5.3354633 0.0007164 287.558 299.589 EM

114.853

15 -0.74385384D+04 3.8799734 0.0005213 298.188 290.909 EM

112.903

16 -0.74358489D+04 2.6894713 0.0003616 306.935 283.538 EM

111.526

17 -0.74339999D+04 1.8490058 0.0002487 314.010 277.327 EM

110.663

18 -0.74326924D+04 1.3075251 0.0001759 319.692 272.102 EM

110.206

19 -0.74317272D+04 0.9651258 0.0001298 324.253 267.702 EM

110.045

TECHNICAL 8 OUTPUT FOR STARTING VALUE SET 315

ITER LOGLIKELIHOOD ABS CHANGE REL CHANGE CLASS COUNTS ALGORITHM

1 -0.19118168D+05 0.0000000 0.0000000 38.080 183.017 EM

480.903

2 -0.75803715D+04 ************ 0.6034991 37.589 211.413 EM

452.998

3 -0.75407550D+04 39.6164941 0.0052262 38.403 235.384 EM

428.213

4 -0.75240896D+04 16.6653601 0.0022100 39.545 253.885 EM

408.570

5 -0.75153050D+04 8.7846876 0.0011675 40.855 267.691 EM

393.454

6 -0.75103480D+04 4.9569762 0.0006596 42.379 277.847 EM

381.774

7 -0.75072670D+04 3.0809762 0.0004102 44.217 285.336 EM

372.447

8 -0.75050937D+04 2.1733355 0.0002895 46.430 290.916 EM

364.654

9 -0.75033924D+04 1.7012210 0.0002267 49.032 295.114 EM

357.854

10 -0.75019708D+04 1.4216291 0.0001895 52.010 298.282 EM

351.708

11 -0.75007301D+04 1.2406903 0.0001654 55.348 300.650 EM

346.001

12 -0.74996034D+04 1.1267306 0.0001502 59.043 302.378 EM

340.580

13 -0.74985366D+04 1.0667512 0.0001422 63.101 303.577 EM

335.322

14 -0.74974851D+04 1.0515141 0.0001402 67.541 304.333 EM

330.126

15 -0.74964113D+04 1.0738714 0.0001432 72.387 304.712 EM

324.901

16 -0.74952813D+04 1.1299669 0.0001507 77.674 304.764 EM

319.562

17 -0.74940613D+04 1.2200228 0.0001628 83.446 304.524 EM

314.030

18 -0.74927128D+04 1.3484315 0.0001799 89.763 304.014 EM

308.223

19 -0.74911889D+04 1.5239786 0.0002034 96.699 303.243 EM

302.058

20 -0.74894299D+04 1.7589150 0.0002348 104.347 302.205 EM

295.448

21 -0.74873635D+04 2.0664723 0.0002759 112.819 300.876 EM

288.305

22 -0.74849103D+04 2.4531668 0.0003276 122.234 299.211 EM

280.554

23 -0.74820050D+04 2.9052665 0.0003881 132.698 297.143 EM

272.159

24 -0.74786277D+04 3.3772945 0.0004514 144.258 294.590 EM

263.152

25 -0.74748249D+04 3.8028754 0.0005085 156.852 291.489 EM

253.659

26 -0.74706960D+04 4.1288711 0.0005524 170.309 287.814 EM

243.878

27 -0.74663578D+04 4.3382413 0.0005807 184.391 283.582 EM

234.027

28 -0.74619120D+04 4.4458038 0.0005954 198.862 278.840 EM

224.298

29 -0.74574338D+04 4.4781315 0.0006001 213.508 273.659 EM

214.833

30 -0.74529849D+04 4.4489213 0.0005966 228.128 268.137 EM

205.735

31 -0.74486471D+04 4.3378274 0.0005820 242.478 262.423 EM

197.099

32 -0.74445569D+04 4.0901390 0.0005491 256.234 256.732 EM

189.034

33 -0.74409014D+04 3.6555730 0.0004910 269.011 251.325 EM

181.663

34 -0.74378559D+04 3.0454229 0.0004093 280.448 246.459 EM

175.093

35 -0.74355058D+04 2.3501269 0.0003160 290.303 242.328 EM

169.369

36 -0.74322745D+04 3.2312615 0.0004346 314.207 234.081 FS

153.712

37 -0.74312638D+04 1.0107723 0.0001360 319.258 234.050 FS

148.692

38 -0.74306435D+04 0.6203068 0.0000835 321.355 233.967 EM

146.678

TECHNICAL 8 OUTPUT FOR STARTING VALUE SET 316

ITER LOGLIKELIHOOD ABS CHANGE REL CHANGE CLASS COUNTS ALGORITHM

1 -0.19228629D+05 0.0000000 0.0000000 501.585 148.900 EM

51.516

2 -0.76847364D+04 ************ 0.6003492 480.833 147.096 EM

74.071

3 -0.75996277D+04 85.1087012 0.0110750 452.638 155.229 EM

94.133

4 -0.75560124D+04 43.6153595 0.0057391 425.708 165.097 EM

111.195

5 -0.75281173D+04 27.8950466 0.0036918 402.371 175.075 EM

124.554

6 -0.75094647D+04 18.6526397 0.0024777 382.949 185.073 EM

133.979

7 -0.74970929D+04 12.3718167 0.0016475 367.034 195.018 EM

139.948

8 -0.74884110D+04 8.6818394 0.0011580 354.062 204.854 EM

143.083

9 -0.74814487D+04 6.9623281 0.0009297 343.289 214.722 EM

143.989

10 -0.74748567D+04 6.5920090 0.0008811 333.878 224.868 EM

143.254

11 -0.74679571D+04 6.8995995 0.0009230 325.097 235.507 EM

141.396

12 -0.74607766D+04 7.1805222 0.0009615 316.461 246.755 EM

138.784

13 -0.74537981D+04 6.9784448 0.0009354 307.780 258.576 EM

135.644

14 -0.74475473D+04 6.2508254 0.0008386 299.144 270.659 EM

132.197

15 -0.74423501D+04 5.1972071 0.0006978 290.845 282.392 EM

128.762

16 -0.74383280D+04 4.0221085 0.0005404 283.206 293.128 EM

125.666

17 -0.74354236D+04 2.9043656 0.0003905 276.449 302.443 EM

123.109

18 -0.74334256D+04 1.9980464 0.0002687 270.651 310.210 EM

121.139

19 -0.74320699D+04 1.3557028 0.0001824 265.780 316.524 EM

119.696

20 -0.74311364D+04 0.9334248 0.0001256 261.744 321.580 EM

118.676

TECHNICAL 8 OUTPUT FOR STARTING VALUE SET 317

ITER LOGLIKELIHOOD ABS CHANGE REL CHANGE CLASS COUNTS ALGORITHM

1 -0.17708429D+05 0.0000000 0.0000000 173.463 111.170 EM

417.367

2 -0.75873404D+04 ************ 0.5715407 200.492 102.707 EM

398.801

3 -0.75273094D+04 60.0309693 0.0079120 209.997 112.508 EM

379.495

4 -0.75098540D+04 17.4554600 0.0023190 211.119 126.160 EM

364.721

5 -0.75021251D+04 7.7289191 0.0010292 208.434 139.479 EM

354.086

6 -0.74974213D+04 4.7037365 0.0006270 203.989 151.341 EM

346.670

7 -0.74940923D+04 3.3289918 0.0004440 198.826 161.411 EM

341.763

8 -0.74916242D+04 2.4681287 0.0003293 193.528 169.663 EM

338.809

9 -0.74897648D+04 1.8594229 0.0002482 188.399 176.268 EM

337.334

10 -0.74883332D+04 1.4315877 0.0001911 183.568 181.500 EM

336.932

11 -0.74871951D+04 1.1380658 0.0001520 179.076 185.649 EM

337.275

12 -0.74862621D+04 0.9330605 0.0001246 174.920 188.971 EM

338.109

TECHNICAL 8 OUTPUT FOR STARTING VALUE SET 318

ITER LOGLIKELIHOOD ABS CHANGE REL CHANGE CLASS COUNTS ALGORITHM

1 -0.29144379D+05 0.0000000 0.0000000 288.111 168.931 EM

244.957

2 -0.76254880D+04 ************ 0.7383548 284.568 199.877 EM

217.555

3 -0.75522018D+04 73.2862775 0.0096107 275.809 225.363 EM

200.827

4 -0.75259472D+04 26.2545982 0.0034764 264.033 245.766 EM

192.201

5 -0.75130700D+04 12.8771185 0.0017110 251.546 261.874 EM

188.580

6 -0.75054048D+04 7.6651852 0.0010202 239.585 274.554 EM

187.861

7 -0.75003121D+04 5.0927565 0.0006785 228.659 284.376 EM

188.966

8 -0.74968169D+04 3.4952122 0.0004660 218.862 291.782 EM

191.356

9 -0.74943812D+04 2.4356886 0.0003249 210.098 297.194 EM

194.708

10 -0.74926322D+04 1.7490172 0.0002334 202.207 301.012 EM

198.781

11 -0.74913142D+04 1.3179322 0.0001759 195.030 303.589 EM

203.381

12 -0.74902645D+04 1.0497820 0.0001401 188.439 305.219 EM

208.342

13 -0.74893853D+04 0.8792028 0.0001174 182.338 306.128 EM

213.534

TECHNICAL 8 OUTPUT FOR STARTING VALUE SET 319

ITER LOGLIKELIHOOD ABS CHANGE REL CHANGE CLASS COUNTS ALGORITHM

1 -0.23954562D+05 0.0000000 0.0000000 48.943 354.510 EM

298.547

2 -0.75373275D+04 ************ 0.6853490 48.605 357.765 EM

295.630

3 -0.74989558D+04 38.3717023 0.0050909 50.518 353.394 EM

298.087

4 -0.74906461D+04 8.3097380 0.0011081 52.726 347.678 EM

301.596

5 -0.74872146D+04 3.4314784 0.0004581 55.011 341.854 EM

305.135

6 -0.74854053D+04 1.8092400 0.0002416 57.242 336.289 EM

308.469

7 -0.74842780D+04 1.1273324 0.0001506 59.331 331.122 EM

311.547

8 -0.74835038D+04 0.7742376 0.0001034 61.232 326.405 EM

314.363

TECHNICAL 8 OUTPUT FOR STARTING VALUE SET 320

ITER LOGLIKELIHOOD ABS CHANGE REL CHANGE CLASS COUNTS ALGORITHM

1 -0.23133265D+05 0.0000000 0.0000000 17.383 142.964 EM

541.653

2 -0.78188031D+04 ************ 0.6620104 24.190 149.242 EM

528.569

3 -0.76716859D+04 147.1172273 0.0188158 40.777 170.470 EM

490.753
[truncated: 3,643,409 more chars]
