# Supplementary material for: Beliefs and misperceptions about naloxone and overdose among U.S. laypersons: a cross-sectional study
Source: BMC Public Health. 2022 May 10;22:924. doi: 10.1186/s12889-022-13298-3 (PMC9086153; doi:10.1186/s12889-022-13298-3)
Supplement: Supplementary file 4 — Additional file 4. (PDF 182 kb) [file 12889_2022_13298_MOESM4_ESM.pdf]

```
  ___  ____  ____  ____  ____(R)
 /__    /   ____/   /   ____/
___/   /   /___/   /   /___/
  Statistics/Data analysis
```

```
  ___  ____  ____  ____  ____ ©
 /__    /   ____/   /   ____/      17.0
___/   /   /___/   /   /___/       SE—Standard Edition
```

Statistics and Data Science

Copyright 1985-2021 StataCorp LLC
StataCorp
4905 Lakeway Drive
College Station, Texas 77845 USA
800-STATA-PC   https://www.stata.com
979-696-4600   stata@stata.com

```
Stata license: 98-user network, expiring 30 Jun 2022
Serial number: 401709300463
  Licensed to: IUAnyWare
               Indiana University

Notes:
      1. Unicode is supported; see help unicode_advice.
      2. Maximum number of variables is set to 5,000; see help set_maxvar.
      3. New update available; type -update all-

1 . use "\\Client\C$\Users\lgolzarr\Indiana University\O365-BCC - Documents\Projects\Ag
  > ley, Jon\Naloxone misinformation\Stata\Data for analysis.dta"

2 . mlogit class c.trust c.age i.gender i.ethnicity b1.race b5.education i.residence c.
  > religious c.political i.party i.profession i.rxtraining i.naloxtraining i.idu i.inj
  > ection i.receivednalox i.witnessod, rrr base(1)

Iteration 0:   log likelihood = -698.64539
Iteration 1:   log likelihood =  -582.6754
Iteration 2:   log likelihood = -571.83125
Iteration 3:   log likelihood = -571.15973
Iteration 4:   log likelihood = -571.02166
Iteration 5:   log likelihood = -570.98872
Iteration 6:   log likelihood = -570.98171
Iteration 7:   log likelihood = -570.98024
Iteration 8:   log likelihood = -570.97991
Iteration 9:   log likelihood = -570.97983
Iteration 10:  log likelihood = -570.97982

Multinomial logistic regression                         Number of obs =    700
                                                        LR chi2(82)   = 255.33
                                                        Prob > chi2   = 0.0000
Log likelihood = -570.97982                             Pseudo R2     = 0.1827
```

class	RRR	Std. err.	z	P>|z|	[95% conf.	interval]
**1**	(base outcome)					
**2**						
trust	.3609185	.0757759	-4.85	0.000	.2391647	.5446549
age	.9921738	.0085444	-0.91	0.362	.9755676	1.009063
gender						
2	1.3612	.2951061	1.42	0.155	.8899853	2.081906
3	.4027659	.3190297	-1.15	0.251	.0852743	1.902335
4	2.442798	3.731545	0.58	0.559	.1223539	48.77051
2.ethnicity	.6305056	.2669236	-1.09	0.276	.2750009	1.445586
race						
0	.5282985	.2666214	-1.26	0.206	.1964697	1.420572
2	1.27568	.4057094	0.77	0.444	.6839598	2.379319
3	1157519	1.28e+09	0.01	0.990	0	.
4	1.025643	.3904055	0.07	0.947	.486397	2.162726

6	1.404744	1.714343	0.28	0.781	.1284679	15.3603
education						
2	.6686964	.6399132	-0.42	0.674	.1024864	4.363064
3	1.442054	.4960592	1.06	0.287	.7348056	2.830027
4	1.185252	.3026689	0.67	0.506	.7185318	1.95513
6	.7967092	.2537062	-0.71	0.475	.4268163	1.487164
7	.9360198	.6098179	-0.10	0.919	.2610516	3.356168
residence						
2	1.006802	.2948308	0.02	0.982	.5671256	1.787348
3	1.914739	.5746886	2.16	0.030	1.063238	3.44817
4	.8867274	.2788308	-0.38	0.702	.4787739	1.64229
5	1.435828	.496026	1.05	0.295	.7295302	2.82593
6	.8221758	.387965	-0.41	0.678	.3260642	2.073129
7	.3992697	.4149378	-0.88	0.377	.0520789	3.061053
religious	1.049418	.041838	1.21	0.226	.9705393	1.134708
political	1.413267	.1021498	4.79	0.000	1.226592	1.628352
party						
2	1.303079	.6153383	0.56	0.575	.5164368	3.287941
3	1.585723	.6820132	1.07	0.284	.6825413	3.684052
profession						
2	.7859477	.5527153	-0.34	0.732	.1980563	3.118879
3	1.245839	1.62533	0.17	0.866	.0966002	16.0674
4	1.258722	1.360188	0.21	0.831	.1513952	10.46519
5	1.015028	.6151886	0.02	0.980	.3094433	3.329467
6	.7019932	.3131693	-0.79	0.428	.2928192	1.682931
7	.8793878	.408339	-0.28	0.782	.35394	2.184899
2.rxtraining	1.14242	.3344594	0.45	0.649	.6436129	2.027809
2.naloxtraining	1.91839	.6873246	1.82	0.069	.950532	3.871748
idu						
2	.9509568	.7178201	-0.07	0.947	.2165907	4.175242
3	796933.7	2.27e+09	0.00	0.996	0	.
4	.4128153	.5837374	-0.63	0.532	.0258299	6.597642
5	5.86e-08	.0000971	-0.01	0.992	0	.
2.injection	2.036713	5.490202	0.26	0.792	.0103375	401.2777
2.receivednalox	3.197681	4.11665	0.90	0.367	.2564544	39.87127
2.witnessod	.9596751	.376946	-0.10	0.917	.4444113	2.072351
_cons	5.09027	8.457366	0.98	0.327	.1961052	132.1273
**3**						
trust	.2113512	.0576348	-5.70	0.000	.1238468	.3606822
age	.9846189	.0123821	-1.23	0.218	.960647	1.009189
gender						
2	1.75618	.5239705	1.89	0.059	.9786021	3.151606
3	.8266614	1.008871	-0.16	0.876	.0755975	9.03957
4	3.15e-06	.0075272	-0.01	0.996	0	.
2.ethnicity	.243506	.1231445	-2.79	0.005	.0903743	.6561066
race						
0	.6538068	.4763234	-0.58	0.560	.1567893	2.726356
2	1.035863	.4555041	0.08	0.936	.4375223	2.452474
3	355315.4	3.94e+08	0.01	0.991	0	.
4	1.09005	.5779463	0.16	0.871	.3856008	3.081448
6	1.832594	2.496246	0.44	0.657	.1269439	26.45578
education						
2	.9251456	1.112945	-0.06	0.948	.0875427	9.776884
3	3.317923	1.426286	2.79	0.005	1.428754	7.705043

4	1.881281	.6580259	1.81	0.071	.9478162	3.734075
6	1.344312	.6366414	0.62	0.532	.5313577	3.401053
7	.6142551	.7164626	-0.42	0.676	.0624473	6.042045
residence						
2	1.279092	.5271989	0.60	0.550	.5702496	2.869053
3	1.757263	.746759	1.33	0.185	.7640361	4.04166
4	1.088376	.4820612	0.19	0.848	.4568423	2.592933
5	1.251425	.6154465	0.46	0.648	.4772946	3.281125
6	1.021596	.6695802	0.03	0.974	.2827353	3.691292
7	1.117413	1.415855	0.09	0.930	.0932548	13.38926
religious	1.006772	.0537433	0.13	0.899	.9067604	1.117815
political	1.62075	.1536991	5.09	0.000	1.345845	1.951809
party						
2	2.25316	1.339752	1.37	0.172	.7025206	7.226453
3	1.386382	.73326	0.62	0.537	.4916778	3.909174
profession						
2	.5890983	.5763839	-0.54	0.589	.086568	4.008833
3	2.475669	3.72513	0.60	0.547	.129686	47.25985
4	1.32e-06	.0017166	-0.01	0.992	0	.
5	.3978505	.362465	-1.01	0.312	.0667154	2.372543
6	.542291	.3259651	-1.02	0.309	.1669494	1.76149
7	.4508508	.2863839	-1.25	0.210	.1298204	1.565751
2.rxtraining	1.016131	.4029566	0.04	0.968	.467087	2.210558
2.naloxtraining	3.369855	1.829785	2.24	0.025	1.162573	9.767919
idu						
2	4.793539	3.992289	1.88	0.060	.9369895	24.52323
3	.063122	327.4287	-0.00	1.000	0	.
4	6.78e-07	.0011377	-0.01	0.993	0	.
5	3.48e-07	.000882	-0.01	0.995	0	.
2.injection	8.27e-07	.0011081	-0.01	0.992	0	.
2.receivednalox	2558593	3.99e+09	0.01	0.992	0	.
2.witnessod	1.141903	.65273	0.23	0.816	.3724509	3.50098
_cons	9.63e-06	.0150164	-0.01	0.994	0	.

Note: **_cons** estimates baseline relative risk for each outcome.
Note: 4 observations completely determined. Standard errors questionable.

```
3 . mlogit class c.trust c.age i.gender i.ethnicity b1.race b5.education i.residence c.
  > religious c.political i.party i.profession i.rxtraining i.naloxtraining i.idu i.inj
  > ection i.receivednalox i.witnessod, rrr base(2)

  Iteration 0:   log likelihood = -698.64539
  Iteration 1:   log likelihood =  -582.6754
  Iteration 2:   log likelihood = -571.83125
  Iteration 3:   log likelihood = -571.15973
  Iteration 4:   log likelihood = -571.02166
  Iteration 5:   log likelihood = -570.98872
  Iteration 6:   log likelihood = -570.98171
  Iteration 7:   log likelihood = -570.98024
  Iteration 8:   log likelihood = -570.97991
  Iteration 9:   log likelihood = -570.97983
  Iteration 10:  log likelihood = -570.97982

  Multinomial logistic regression                       Number of obs =    700
                                                        LR chi2(82)   = 255.33
                                                        Prob > chi2   = 0.0000
  Log likelihood = -570.97982                           Pseudo R2     = 0.1827
```

class	RRR	Std. err.	z	P>|z|	[95% conf.	interval]
**1**						
trust	2.770708	.5817181	4.85	0.000	1.836025	4.18122
age	1.007888	.0086797	0.91	0.362	.9910187	1.025044
gender						
2	.7346457	.1592701	-1.42	0.155	.480329	1.123614
3	2.482832	1.966644	1.15	0.251	.5256697	11.72686
4	.4093666	.625336	-0.58	0.559	.0205042	8.173011
2.ethnicity	1.586029	.6714429	1.09	0.276	.6917611	3.636352
race						
0	1.892869	.9552921	1.26	0.206	.7039417	5.089844
2	.7838959	.2493055	-0.77	0.444	.4202884	1.462074
3	8.64e-07	.0009586	-0.01	0.990	0	.
4	.9749982	.3711279	-0.07	0.947	.4623794	2.055934
6	.7118735	.8687672	-0.28	0.781	.0651029	7.784044
education						
2	1.495447	1.431077	0.42	0.674	.2291967	9.757389
3	.6934554	.2385452	-1.06	0.287	.3533536	1.360904
4	.8437021	.2154498	-0.67	0.506	.5114748	1.391727
6	1.255163	.3996975	0.71	0.475	.6724209	2.342929
7	1.068353	.6960334	0.10	0.919	.2979588	3.83066
residence						
2	.9932438	.2908604	-0.02	0.982	.5594882	1.763278
3	.5222644	.1567521	-2.16	0.030	.2900089	.9405232
4	1.127742	.3546177	0.38	0.702	.6089059	2.088669
5	.6964624	.2406023	-1.05	0.295	.3538659	1.370745
6	1.216285	.5739357	0.41	0.678	.4823627	3.066881
7	2.504573	2.602858	0.88	0.377	.326685	19.20163
religious	.9529088	.0379904	-1.21	0.226	.8812838	1.030355
political	.7075804	.0511433	-4.79	0.000	.6141178	.8152672
party						
2	.7674134	.3623871	-0.56	0.575	.3041417	1.936346
3	.6306272	.2712302	-1.07	0.284	.2714403	1.465113
profession						
2	1.272349	.8947758	0.34	0.732	.320628	5.049069
3	.802672	1.047172	-0.17	0.866	.0622378	10.35194
4	.7944569	.8584986	-0.21	0.831	.0955549	6.605229
5	.9851948	.5971075	-0.02	0.980	.3003484	3.23161
6	1.424515	.635497	0.79	0.428	.5942015	3.415077
7	1.137155	.5280316	0.28	0.782	.4576872	2.825338
2.rxtraining	.8753347	.2562664	-0.45	0.649	.4931432	1.553729
2.naloxtraining	.5212704	.1867618	-1.82	0.069	.2582813	1.052042
idu						
2	1.051572	.7937689	0.07	0.947	.2395071	4.617003
3	1.25e-06	.0035702	-0.00	0.996	0	.
4	2.422391	3.425357	0.63	0.532	.1515693	38.7148
5	1.71e+07	2.83e+10	0.01	0.992	0	.
2.injection	.4909873	1.323515	-0.26	0.792	.002492	96.73541
2.receivednalox	.3127266	.4025999	-0.90	0.367	.0250807	3.899328
2.witnessod	1.042019	.4092895	0.10	0.917	.4825436	2.250168
_cons	.1964532	.3264025	-0.98	0.327	.0075685	5.099305
**2**	(base outcome)					

**3**						
trust	.5855926	.1278939	-2.45	0.014	.381674	.8984598
age	.9923855	.0109546	-0.69	0.489	.9711455	1.01409
gender						
2	1.29017	.3298126	1.00	0.319	.781718	2.129334
3	2.052461	2.5779	0.57	0.567	.1750501	24.0651
4	1.29e-06	.0030814	-0.01	0.995	0	.
2.ethnicity	.3862076	.1560343	-2.35	0.019	.1749529	.8525512
race						
0	1.237571	.8689352	0.30	0.761	.3125476	4.900314
2	.8120089	.2992668	-0.57	0.572	.3943206	1.672138
3	.3069628	.3984359	-0.91	0.363	.0241119	3.907871
4	1.062797	.5017882	0.13	0.897	.4212738	2.681242
6	1.304575	1.219556	0.28	0.776	.2088045	8.150761
education						
2	1.383506	1.362576	0.33	0.742	.2007506	9.534661
3	2.300832	.7978051	2.40	0.016	1.166095	4.539792
4	1.58724	.4815897	1.52	0.128	.8757435	2.876792
6	1.687331	.733829	1.20	0.229	.7194629	3.957239
7	.6562416	.7295458	-0.38	0.705	.0742644	5.79892
residence						
2	1.27045	.4694124	0.65	0.517	.6158165	2.620981
3	.9177559	.3352974	-0.23	0.814	.4484821	1.878059
4	1.227407	.4838967	0.52	0.603	.566772	2.658085
5	.8715702	.36932	-0.32	0.746	.3798511	1.999822
6	1.242552	.7200413	0.37	0.708	.3990757	3.868778
7	2.798643	2.936377	0.98	0.327	.3579766	21.87966
religious	.959362	.0421814	-0.94	0.345	.8801501	1.045703
political	1.146811	.0881166	1.78	0.075	.9864813	1.333199
party						
2	1.729106	.8049176	1.18	0.239	.6943491	4.305912
3	.8742899	.3443031	-0.34	0.733	.404059	1.89176
profession						
2	.7495388	.6489218	-0.33	0.739	.1373589	4.090078
3	1.987151	2.077013	0.66	0.511	.2561747	15.41435
4	1.05e-06	.0013638	-0.01	0.992	0	.
5	.3919603	.318515	-1.15	0.249	.0797147	1.927285
6	.7725019	.403593	-0.49	0.621	.2774534	2.150845
7	.5126871	.2842279	-1.21	0.228	.1729641	1.519668
2.rxtraining	.8894551	.3054795	-0.34	0.733	.4537144	1.743675
2.naloxtraining	1.756606	.8595004	1.15	0.250	.6732629	4.583148
idu						
2	5.040753	3.717417	2.19	0.028	1.18784	21.3911
3	7.92e-08	.0003435	-0.00	0.997	0	.
4	1.64e-06	.0027559	-0.01	0.994	0	.
5	5.937413	17974.16	0.00	1.000	0	.
2.injection	4.06e-07	.0005441	-0.01	0.991	0	.
2.receivednalox	800140.2	1.25e+09	0.01	0.993	0	.
2.witnessod	1.189885	.6068375	0.34	0.733	.4379203	3.233069
_cons	1.89e-06	.00295	-0.01	0.993	0	.

Note: **_cons** estimates baseline relative risk for each outcome.
Note: 4 observations completely determined. Standard errors questionable.

4 .
